# Characterization of Carboxylic Acid Reductases as Enzymes in the Toolbox for Synthetic Chemistry

**DOI:** 10.1002/cctc.201601249

**Published:** 2017-02-14

**Authors:** William Finnigan, Adam Thomas, Holly Cromar, Ben Gough, Radka Snajdrova, Joseph P. Adams, Jennifer A. Littlechild, Nicholas J. Harmer

**Affiliations:** ^1^ Biosciences, College of Life and Environmental SciencesUniversity of Exeter, Stocker RoadDevonExeterEX4 4QDUK; ^2^ Synthetic Chemistry, AC-API Chem-UK, GlaxoSmithKline R&D LtdMedicines Research CentreGunnels Wood RoadStevenageHertfordshireSG1 2NYUK

**Keywords:** biocatalysis, carboxylic acids, enzymes, green chemistry, reduction

## Abstract

Carboxylic acid reductase enzymes (CARs) meet the demand in synthetic chemistry for a green and regiospecific route to aldehydes from their respective carboxylic acids. However, relatively few of these enzymes have been characterized. A sequence alignment with members of the ANL (Acyl‐CoA synthetase/ NRPS adenylation domain/Luciferase) superfamily of enzymes shed light on CAR functional dynamics. Four unstudied enzymes were selected by using a phylogenetic analysis of known and hypothetical CARs, and for the first time, a thorough biochemical characterization was performed. Kinetic analysis of these enzymes with various substrates shows that they have a broad but similar substrate specificity. Electron‐rich acids are favored, which suggests that the first step in the proposed reaction mechanism, attack by the carboxylate on the α‐phosphate of adenosine triphosphate (ATP), is the step that determines the substrate specificity and reaction kinetics. The effects of pH and temperature provide a clear operational window for the use of these CARs, whereas an investigation of product inhibition by NADP^+^, adenosine monophosphate, and pyrophosphate indicates that the binding of substrates at the adenylation domain is ordered with ATP binding first. This study consolidates CARs as important and exciting enzymes in the toolbox for sustainable chemistry and provides specifications for their use as a biocatalyst.

## Introduction

Green chemistry” is of increasing global importance, driven by the need to balance sustainable and efficient resource utilization with the demands and increasing consumption of an increasing population.^1^ Biological solutions to chemistry challenges are a critical component to meet this demand. The use of isolated enzymes and cell‐based systems that produce negligible dangerous waste, often with higher yields, offers an alternative to traditional chemical processes. In some cases, biological alternatives are more rapid and cost‐effective than their chemical counterpart.^2^ Despite these potential advantages, enzymes are still underused in chemistry. An expansion of the toolbox of available enzymes is essential for the successful development of new synthetic routes and sustainable manufacturing processes.^3^

An important opportunity ripe for exploitation is synthetic routes based on organic acids. These compounds have a long history of production by fermentation.^4^ Indeed, multiple carboxylic acids were identified as “Top Value Added Chemicals From Biomass”,^5^ many of which are now produced industrially. The reduction products of these organic acids, especially optically pure aldehydes and alcohols, are essential building blocks for use in the chemical, pharmaceutical, and food industries.^6^ However, chemical methods for the reduction of carboxylic acids are limited and require chemicals such as lithium aluminum hydride and sodium borohydride in stoichiometric amounts.^7^

Two enzyme classes are able to reduce organic acids to aldehydes, and the biocatalytic reductions possible by organisms that harbor them have been reviewed.^7^ The aldehyde oxidoreductases (AORs) oxidize organic aldehydes reversibly to their respective acids. The oxidized product is more thermodynamically favorable, and so the equilibrium tends towards this product. Therefore, AORs are more useful for syntheses that require the oxidation of aldehydes.^8^, ^9^ In contrast, carboxylic acid reductases (CARs) catalyze the reduction of a carboxylic acid to an aldehyde at the expense of adenosine triphosphate (ATP) and NADPH to produce adenosine monophosphate (AMP), pyrophosphate (PP_i_), and NADP^+^ as byproducts.^6^ The reduction of carboxylic acids into aldehydes by using CARs has been confirmed previously in a number of studies by using GC–MS. Products other than the aldehyde have not been detected.^10^, ^11^ The hydrolysis of ATP makes the reduction of acids to aldehydes by CARs strongly thermodynamically favorable, which makes their use an attractive “green chemical” route to aldehyde production.^7^ This synthesis can be coupled to other enzymes, such as an alcohol dehydrogenase, to provide a complete route to the alcohol product.^10^

Indeed, CARs have been employed in a number of synthetic pathways. These include the production of the flavor vanillin by yeast^12^ and a synthetic pathway for the production of propane in *Escherichia coli*.^13^ These examples both highlight the potential of CARs as part of a toolbox for the synthesis of fine chemicals from non‐oil‐based chemical precursors.^5^

**Figure 1:**
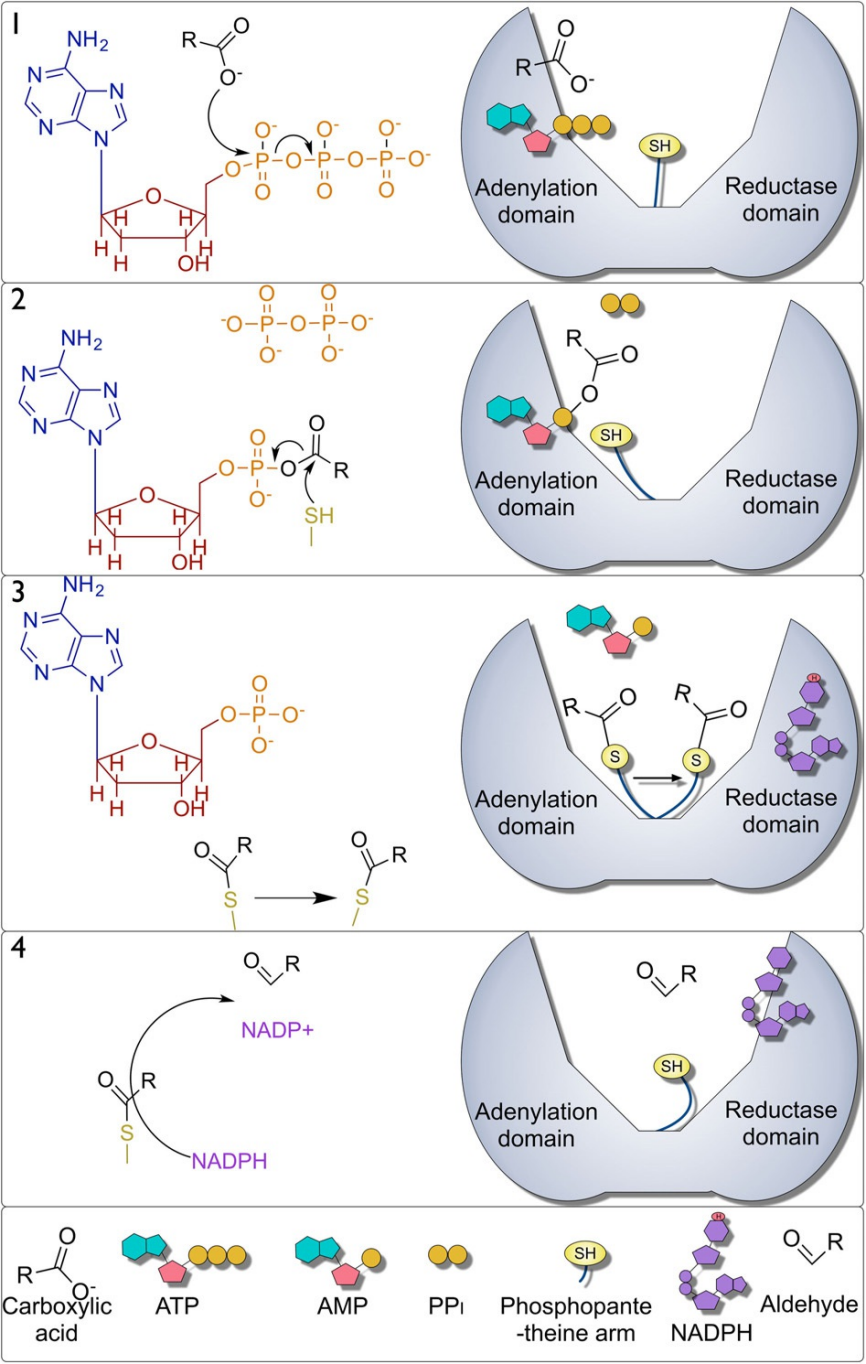
Proposed mechanism of CAR enzymes. 1) ATP and a carboxylic acid enter the adenylation domain in which a phosphoester intermediate is formed with the release of pyrophosphate in the process. 2) The thiol of the phosphopantetheine arm attacks the carbonyl carbon atom of this intermediate nucleophilically to form a thioester intermediate with the phosphopantetheine arm and release AMP. 3) The phosphopantetheine arm transfers to the reduction domain in which 4) the thioester bond is reduced by NADPH to release an aldehyde product, which regenerates the phosphopantetheine thiol group and produces NADP^+^.

CARs are relatively large, multidomain enzymes of around 130 kDa. They feature an N‐terminal adenylation domain, a C‐terminal thioester reductase domain that likely adopts a Rossmann fold, and a central phosphopantetheine binding domain (Figure 1).^14^ A phosphopantetheine arm must be attached covalently to a conserved serine in this central domain through the action of a phosphopantetheine transferase to produce an active enzyme.^15^ Fungal α‐aminoadipate reductases, which are responsible for the reduction of α‐aminoadipate to α‐aminoadipate semialdehyde in lysine biosynthesis, share this domain architecture and also require the loading of a central phosphopantetheine prosthetic group.^16^ However, these enzymes have a different substrate specificity from CARs.^11^

Phosphopantetheine arms are most commonly associated with acyl carrier proteins in which they maintain an acyl chain in a energetically active thioester bond, and the length and flexibility of the arm allows access to spatially distinct active sites.^17^ In CARs, the phosphopantetheine arm is believed to act in much the same way and shuttles an attached acyl chain between the N‐ and C‐terminal domains.^15^

The proposed mechanism of CAR enzymes has four main steps (Figure 1). In the first two steps, the relatively unreactive carboxylic acid is activated to form a thioester with the phosphopantetheine arm at the N‐terminal adenylation domain, in a mechanism possibly similar to that of the ANL (Acyl‐CoA synthetase/ NRPS adenylation domain/Luciferase) superfamily of adenylating enzymes such as long‐chain fatty acid CoA ligases.^18^, ^19^ (1) ATP and a carboxylic acid enter the active site of the adenylation domain in which the α‐phosphate of ATP is attacked by an O atom from the carboxylic acid to form an AMP‐acyl phosphoester with the release of pyrophosphate.^19^ (2) The thiol group of the phosphopantetheine arm can then attack the carbonyl carbon atom of the AMP‐acyl phosphoester intermediate nucleophilically to release AMP and to form an acyl thioester with the phosphopantetheine arm. (3) The phosphopantetheine arm transfers to the C‐terminal reductase domain in which (4) the thioester is reduced by NADPH, the aldehyde and NADP^+^ are released, and the thiol of the phosphopantetheine arm is regenerated in the process.^7^

Relatively few CARs have been explored to date. CARs were first described in *Neurospora crassa* as an aryl aldehyde: NADP^+^ oxidoreductase.^20^ Recently, this CAR has been further characterized.^21^ Subsequently, CARs were characterized from *Nocardia asteroides* JCM 3016^22^ and later *Nocardia iowensis* 23 (niCAR) when they were reclassified as carboxylic acid reductases.^23^ The *Nocardia asteroides* JCM 3016 CAR was characterized by comparing the relative activity of this enzyme towards various aromatic substrates (1 mm concentration). This CAR was reported to prefer benzoates and aliphatic acids that were substituted with a phenyl group in the 3‐position. No reaction of this CAR with simple aliphatic acids has been reported. The optimum pH for the activity of this enzyme was pH 7.5, and the optimum temperature for activity was 40 °C.^22^

The relative activities of the niCAR against various aromatic substrates have also been reported. The highest activity was achieved with indole‐5‐carboxylic acid, which was the most activated carboxylic acid tested. Substrates with benzoates in the 2‐position or ring‐deactivating groups showed no or very low levels of activity. The reduction of racemic ibuprofen by whole *Nocardia iowensis* cells gave a enantiomeric excess (*ee*) of 61.2 %, which has been attributed to enantioselectivity by niCAR based on kinetic data for its reduction of (*S*)‐(+)‐ and (*R*)‐(−)‐ibuprofen enantiomers.^23^ The requirement for the presence of a phosphopantetheine transferase for the loading of a phosphopantetheine group onto the CAR enzyme was shown for niCAR^23^ and is presumed to be the case for all the CAR enzymes.

A CAR from *Mycobacterium marinum* has also been described, and its application for the reduction of fatty acids to fatty alcohols has been explored. This CAR is active against C_2_–C_18_ fatty acids.^10^ CARs have also been reported in the fungi *Syncephalastrum racemosum* 24 and *Trametes versicolor*.^25^

Recently the characterization of CARs from *Nocardia iowensis*, *Nocardia brasiliensis*, *Mycobacterium marinum*, and *Mycobacterium smegmatis* showed that CARs prefer substrates in which the carboxylic acid was the only polar or charged group, which gives a useful insight into the substrate specificity of these enzymes. Moreover, a model was developed for the prediction of CAR reactivity using this and previous CAR data.^11^ Notably, the CAR characterized by Moura et al. is distinct from msCAR used in this study.

Here, we have produced a detailed phylogeny of the CARs and identified four previously undescribed CARs for further study that are broadly spread across this phylogeny. With the addition of niCAR for comparison to earlier work, a thorough biochemical characterization was performed on each. We investigated the effects of temperature and pH to identify suitable conditions for the use of CARs in biocatalytic reactions. We further performed a full kinetic analysis on a range of aromatic and aliphatic substrates with these CARs to look for potential differences in their substrate specificity and to examine the effects of various functional groups on their kinetic parameters. Finally, we describe potential issues of product inhibition with the CAR enzymes. Our investigation provides a more thorough description of the factors to be considered if the CAR enzymes are used in industrial biocatalysis.

## Results

### Alignment and phylogenetic analysis

CAR adenylation domains were aligned with a firefly luciferase, a fatty acyl‐CoA ligase and a reductase domain from a nonribosomal peptide synthetase, all from the ANL superfamily (Figure S1). CARs share ∼20 % sequence homology with other ANL superfamily members. Members of the ANL superfamily catalyze the initial adenylation of a carboxylic acid to form an acyl‐AMP intermediate, which is followed generally by the formation of a thioester. The family name is based on three of its subfamilies: acyl‐CoA synthetases, the nonribosomal peptide synthase (NRPS) adenylation domain, and the Luciferase enzyme.^18^ Previous alignment and crystallography studies have identified 10 motifs that are conserved within the superfamily. Of these 10, five are conserved strongly within the CARs, which include the active site ppxTSGSTGxPK, rGxTE, and TGD motifs (in which p=aliphatic and r=aromatic residues). These motifs are considered as a “signature” of the ANL superfamily and are involved in the hydrolysis of ATP.^26^ The remaining five motifs are also present albeit with a lower conservation.

A total of 48 unique sequences that show homology to known CAR proteins were gathered by using pBLAST or mined directly from GenBank by raw text searches (Figure S2). All sequences identified were solely from the subclass *actinobacteridae*. Within this subclass, sequences were obtained from the families *Streptomycetaceae* and *Corynebacterinae*.

**Figure 2:**
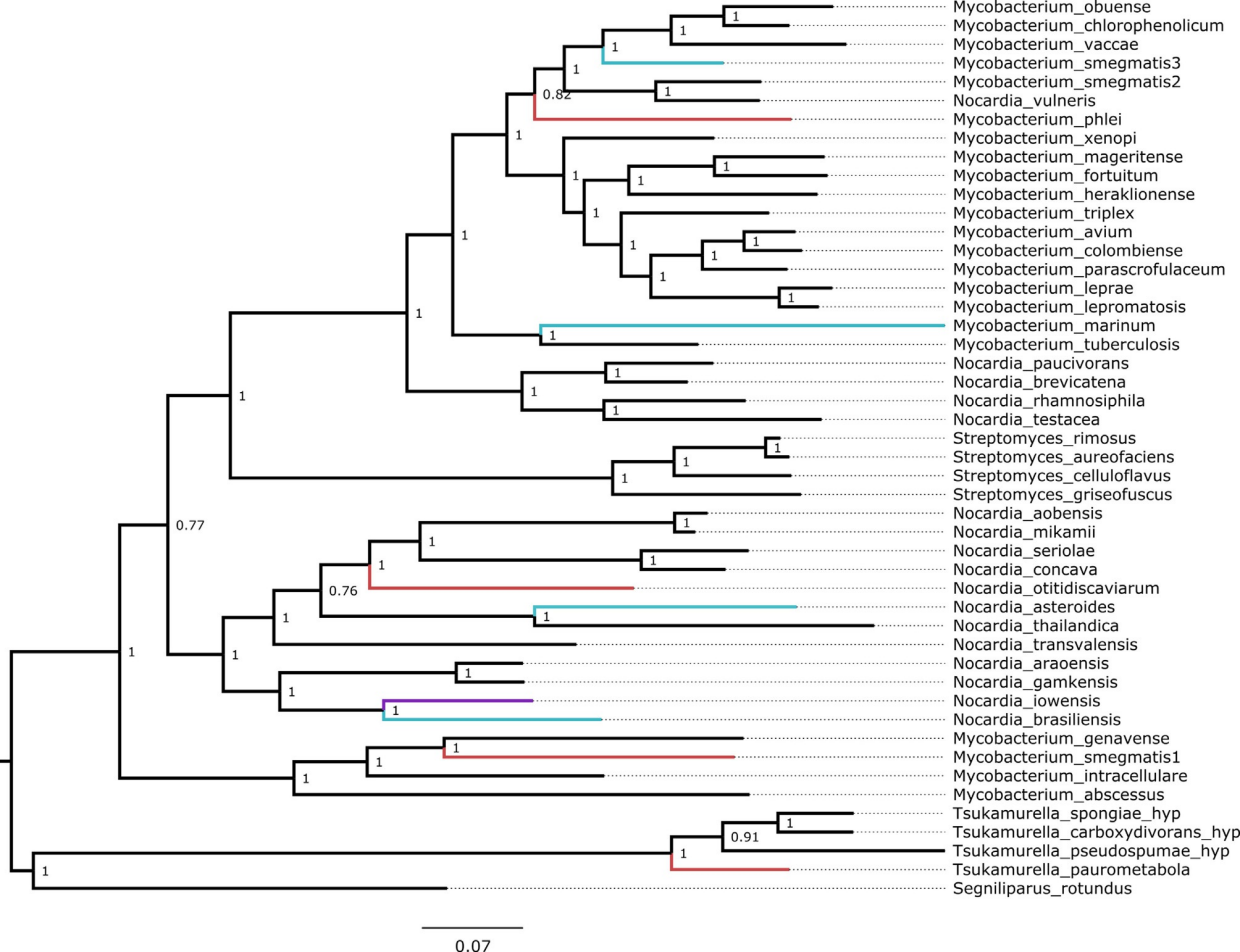
Phylogenetic tree of CAR enzymes. A midpoint rooted phylogeny of a masked alignment of 48 carboxylic acid reductase sequences retrieved from GenBank. The phylogeny was constructed by using MrBayes and visualized in FigTree. Node labels represent Bayesian posterior probabilities that describe node reliability (in which 1 is unequivocal) computed by using MrBayes. Colored branches represent CARs that have been studied: Blue—in previous research, Red—in this study, Purple—both in this study and previously.

A masked multiple sequence alignment of the dataset was shown to be best fit to the Whelan and Goldman model of amino acid substitution with a discrete γ distribution of mutation rates and an assumed presence of invariant sites (WAG+I+G). This model was implemented into a Bayesian phylogenetic reconstruction (Figure 2). According to 16S data, the *Streptomycetaceae* are thought to have evolved before the *Corynebacterinae*. However, rooting the tree on the streptomycetes has poor parsimony as numerous gene loss events would have had to have occurred for this to be the case.^27^ Instead, as an outgroup was unobtainable for this dataset, we opted to root the tree on its midpoint. The tree is extremely well supported: all nodes possess a confidence score of >0.75 and only four of 46 biologically relevant nodes are scored at below the highest possible confidence score of 1.

To better understand how CAR functionality differs across clades, we selected sequences for characterization from a range of host organisms that broadly cover distinct areas of the phylogenetic tree.

### Expression and purification

CAR enzymes (Table 1) were expressed in *E. coli* and purified from the cell lysates by nickel affinity chromatography followed by gel filtration to obtain a high level of purity (Figure S3). The optimum conditions for the expression of *Mycobacterium phlei* CAR (mpCAR) in *E. coli* in lysogeny broth (LB) medium were determined to be induction at OD_600_ 0.6 with 150 μm isopropyl β‐d‐1‐thiogalactopyranoside (IPTG), followed by incubation for approximately 18 h at 20 °C with orbital shaking at 225 rpm (data not shown). Similar conditions were assumed to be suitable for the expression of the other CAR enzymes, and indeed all CARs were well expressed. CARs were coexpressed with the Sfp phosphopantetheinyl transferase from *Bacillus subtilis* on a separate plasmid as the loading of a phosphopantetheine group onto CAR enzymes is essential for activity.^15^

**Table 1:** CARs chosen for this study.

Abbreviation	Source	NCBI Reference:
mpCAR	*Mycobacterium phlei*	WP_003889896.1
msCAR	*Mycobacterium smegmatis*	AFP42026.1
niCAR	*Nocardia iowensis*	Q6RKB1.1
noCAR	*Nocardia otitidiscaviarum*	WP_029928026.1
tpCAR	*Tsukamurella paurometabola*	WP_013126039.1

Five CARs were chosen for a thorough biochemical characterization from a range of host organisms that contain putative CARs. niCAR has been characterized previously and was chosen for comparison.^11^, ^23^ CAR abbreviations were chosen to reflect their source. NCBI ascension numbers are shown to access the protein sequences. The sequence identities of these five orthologues are provided in Table S1.

### Kinetic characterization of CAR enzymes

**Figure 3:**
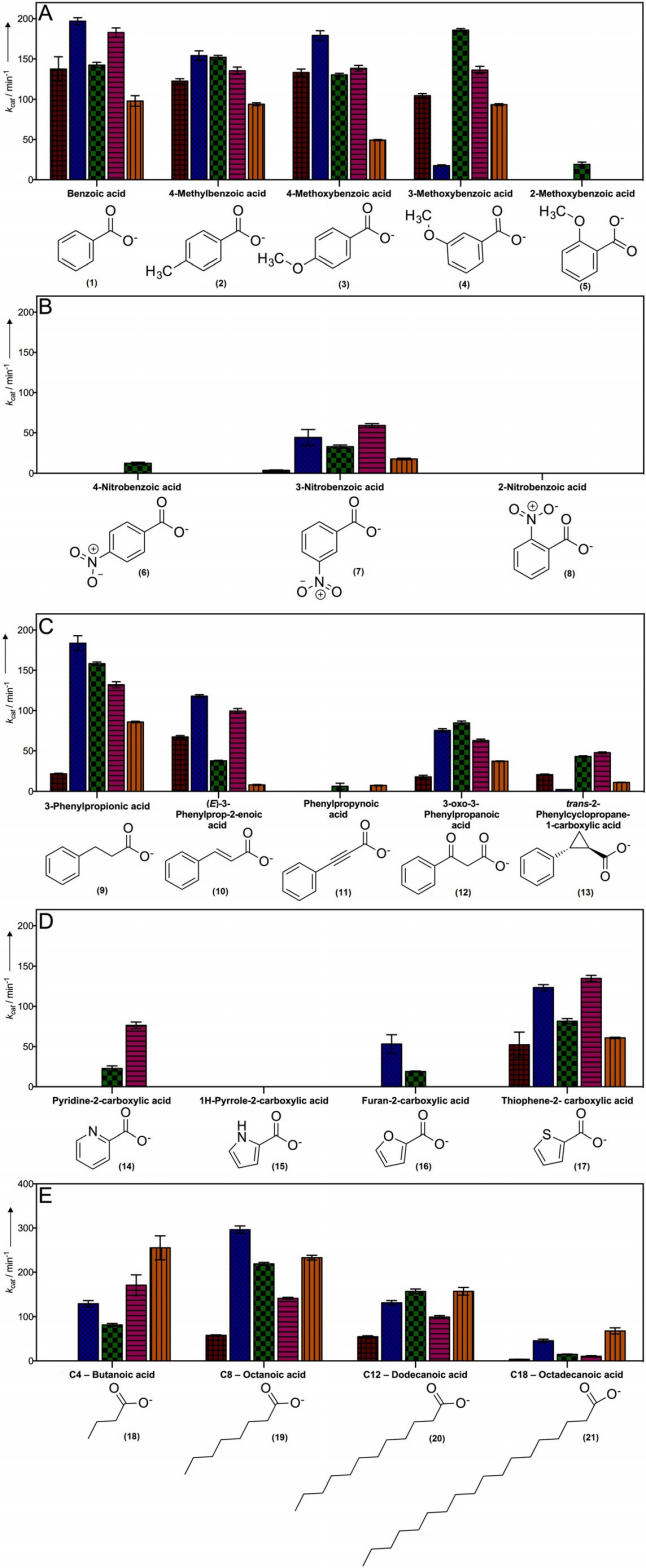
CAR activity for various benzoic acid derivatives, heterocycles, and fatty acids. The *k*_cat_ [min^−1^] determined for each enzyme against each substrate (mpCAR, msCAR, tpCAR, noCAR, and niCAR). Below each substrate is its chemical structure. Error bars show the standard error. A) Benzoic acid and derivatives with electron‐donating groups. B) Derivatives with an electron‐withdrawing groups. C) Derivatives with various substituents between the carboxylate group and benzene ring. D) Heterocycles that contain either an oxygen, sulfur, or nitrogen atom. E) C_4_–C_18_ fatty acids.

The CAR enzymes were characterized in terms of their substrate specificity towards a range of aromatic carboxylic acids, a range of aliphatic unsaturated fatty acids, and the cofactors ATP and NADPH. A list of the substrates and their chemical structures is given in Figure 3 and Figure S4. For each CAR, an initial assay at a high substrate concentration (5 mm) was performed to identify compounds for which the CAR had activity. For those compounds for which activity was detected, a full kinetic analysis was performed (Figures S5–S9). All the CARs that were tested showed similar Michaelis constant (*K*_m_) values for NADPH and ATP. For NADPH *K*_m_=24–36 μm, whereas for ATP *K*_M_=64–84 μm. These values are well within the physiological ranges for these cofactors and in good agreement with previous studies.^6^, ^10^, ^23^ The production of benzaldehyde and 4‐methylbenzaldehyde from the derivative acids was confirmed by using HPLC, and no other products were observed. NADPH consumption was also confirmed as a good measure of aldehyde production (Figures S10 and S11).

### Effect of electron density on aryl‐substituted carboxylic acid substrates

All of the enzymes that we tested showed a strong activity against the classical CAR substrate benzoic acid (**1**; Figure 3 A and Table 2), in line with all CARs studied previously.^6^, ^10^, ^11^ A series of substituents with various electronic configurations was tested (**2**–**5**; Figure 3 A and Table 2). Compounds with systems that were more electron‐rich generally exhibited a decreased *K*_m_ to give an increased catalytic efficiency compared to benzoic acid. A minimal activity was detected with 2‐methoxybenzoic acid.

**Table 2:** CAR activity against benzoic acid and its derivatives with electron‐donating and ‐withdrawing groups.

		Benzoic acid (**1**)	4‐Methylbenzoic acid (**2**)	4‐Methoxybenzoic acid (**3**)	3‐Methoxybenzoic acid (**4**)	2‐Methoxybenzoic acid (**5**)	4‐Nitrobenzoic acid (**6**)	3‐Nitrobenzoic acid (**7**)	2‐Nitrobenzoic acid (**8**)
Hammett		0	−0.17	−0.27	0.12	–	0.71	0.78	–
*σ* constants^28^									
mpCAR	*k*_cat_ [min^−1^]	140±20*	122±3	132±4	104±3	NA	NA	3.7±0.5	NA
	*K*_m_ [mm]	20±4*	3.7±0.2	2.8±0.2	3.0±0.2	NA	NA	0.3±0.1	NA
	*k*_cat_/*K*_m_ [min^−1^ mm ^−1^]	7±1*	33±2	48±5	35±3	NA	NA	11±4	NA
msCAR	*k*_cat_ [min^−1^]	197±4	154±6	179±6	18±1*	NA	NA	40±10	NA
	*K*_m_ [mm]	3.4±0.2	0.16±0.02	0.19±0.02	12±1*	NA	NA	0.5±0.2	NA
	*k*_cat_/*K*_m_ [min^−1^ mm ^−1^]	57±4	900±100	930±80	1.4±0.2*	NA	NA	100±50	NA
tpCAR	*k*_cat_ [min^−1^]	142±3	152±2	130±2	186±2	19±3	13±1	33±2	NA
	*K*_m_ [mm]	2.0±0.1	0.69±0.03	0.45±0.02	0.56±0.02	9±3	0.6±0.1	0.7±0.1	NA
	*k*_cat_/*K*_m_ [min^−1^ mm ^−1^]	72±6	220±10	290±10	334±10	2.2±0.7	22±6	44±8	NA
noCAR	*k*_cat_ [min^−1^]	183±6	135±5	138±4	136±5	NA	NA	59±2	NA
	*K*_m_ [mm]	2.1±0.2	1.2±0.2	1.1±0.1	0.9±0.1	NA	NA	2.5±0.3	NA
	*k*_cat_/*K*_m_ [min^−1^ mm ^−1^]	89.1±8	110±20	130±10	150±10	NA	NA	24±3	NA
niCAR	*k*_cat_ [min^−1^]	98±7	94±2	49±1	93±1	NA	NA	18±1	NA
	*K*_m_ [mm]	0.9±0.1	1.0±0.1	0.25±0.01	0.68±0.03	NA	NA	5.6±0.7	NA
	*k*_cat_/*K*_m_ [min^−1^ mm ^−1^]	103±9	97±6	200±10	137±6	NA	NA	3.2±0.4	NA

NA: no activity was detected with that substrate. *: *K*_m_ was unusually large and substrate concentrations could not reach a high enough concentration to be able to determine the kinetic constants accurately. Errors represent the standard error. No Hammett constants are shown for substrates substituted in the 2‐position as steric effects cannot be accounted for properly.

In contrast, the incorporation of an electron‐withdrawing nitro group in the benzene ring (**6**–**8**; Figure 3 B and Table 2) resulted in a large decrease in the turnover number of the CARs, which in most cases inhibited the activity altogether. Again, there was no detectable activity with a nitro group in the 2‐position, whereas with the substituent in the *para* position, only tpCAR showed a low level of activity. However, all the CARs tested were active against 3‐nitrobenzoic acid with a lower *k*_cat_ than that with benzoic acid. Absorbance at OD_340 nm_ by nitro compounds was shown not to interfere with the assay (Figure S12).

### Effect of the aromatic unit on catalytic activity

3‐Phenylpropionic acid (**9**; Figure 3 C and Table 3) has a carboxylate group not conjugated to the aryl ring that is extended away from the aryl ring by two carbon atoms to give the carboxylate group a greater flexibility. If tested with the CARs, this change caused a decreased *K*_m_ with a similar or slightly lower *k*_cat_. (*E*)‐3‐Phenylprop‐2‐enoic acid (**10**; Figure 3 C and Table 3), which is a conjugated system, was expected to have activity between that of 3‐phenylpropionic acid and the model compound benzoic acid. The CAR activity against (*E*)‐3‐phenylprop‐2‐enoic acid showed a substantially decreased *k*_cat_ compared to that of 3‐phenylpropionic acid or benzoic acid, with a further slight decrease of *K*_m_.

**Table 3:** CAR activity against benzoic acid derivatives with the carboxylic acid group extended from the ring.

		3‐Phenylpropionic	(*E*)‐3‐Phenylprop‐2‐enoic	Phenylpropynoic	3‐Oxo‐3‐phenylpropanoic	*trans*‐2‐Phenylcyclopropane‐1‐
		acid (**9**)	acid (**10**)	acid (**11**)	acid (**12**)	carboxylic acid (**13**)
mpCAR	*k*_cat_ [min^−1^]	21.5±0.7	67±2	NA	18±2	20±1
	*K*_m_ [mm]	3.0±0.3	0.3±0.02	NA	3.8±0.8	1.8±0.2
	*k*_cat_/*K*_m_ [min^−1^ mm ^−1^]	7.2±0.7	240±2	NA	5±1	12±1
msCAR	*k*_cat_ [min^−1^]	184±9	118±2	NA	75±2	2.2±0.1
	*K*_m_ [mm]	0.16±0.02	0.075±0.004	NA	0.27±0.02	0.006±0.0001
	*k*_cat_/*K*_m_ [min^−1^ mm ^−1^]	1200±200	1600±500	NA	280±20	380±20
tpCAR	*k*_cat_ [min^−1^]	158±2	38±1	6±4	85±2	43±1
	*K*_m_ [mm]	0.32±0.01	0.310±0.002	0.09±0.02	0.55±0.04	0.061±0.005
	*k*_cat_/*K*_m_ [min^−1^ mm ^−1^]	500±20	120±2	70±40	150±10	700±60
noCAR	*k*_cat_ [min^−1^]	140±4	105±3	NA	63±2	48±1
	*K*_m_ [mm]	2.7±0.2	0.72±0.07	NA	0.29±0.03	1±0.1
	*k*_cat_/*K*_m_ [min^−1^ mm ^−1^]	52±4	147±15	NA	210±20	46±3
niCAR	*k*_cat_ [min^−1^]	85.8±0.9	7.7±0.7	7±0.4	37.1±0.5	10.8±0.3
	*K*_m_ [mm]	0.97±0.03	0.05±0.02	1.3±0.2	0.39±0.02	0.21±0.02
	*k*_cat_/*K*_m_ [min^−1^ mm ^−1^]	88±3	170±70	5±1	94±4	51±5

NA: no activity was detected with that substrate. Errors represent the standard error.

The cognate compound with a triple bond (phenylpropynoic acid, **11**; Figure 3 C and Table 3) showed a very low or no detectable activity in the CAR reaction.

Two other compounds were tested: firstly, the β‐keto acid 3‐oxo‐3‐phenylpropanoic acid (**12**; Figure 3 C and Table 3) showed an increase in *K*_m_ with mpCAR, msCAR, and tpCAR but a decrease in *K*_m_ with noCAR and niCAR in comparison to 3‐phenylpropionic acid. However, in all cases, *k*_cat_ was decreased compared to that of 3‐phenylpropionic acid or benzoic acid as it was for (*E*)‐3‐phenylprop‐2‐enoic acid. Finally, *trans*‐2‐phenylcyclopropane‐1‐carboxylic acid (**13**; Figure 3 C and Table 3) features a cyclopropane ring between the benzene ring and carboxylate group. For all the CARs this modification resulted in much lower *K*_m_ values and a much lower *k*_cat_ compared to that of 3‐phenylpropionic acid or benzoic acid.

### Heterocycles

Heterocycles that contain N, O, or S were tested (**14**–**17**; Figure 3 D and Table 4). Generally, weak activity was observed with decreasing *K*_m_ values with the increasing heteroatom size. If there was activity, *k*_cat_ was generally lower than that observed with benzoic acid as the substrate. If no activity was detected, it is possible that the *K*_m_ was outside the range of detection of the assay.

**Table 4:** CAR activity against heterocycles and fatty acids.

		Pyridine‐2‐	1*H*‐Pyrrole‐2‐	Furan‐2‐	Thiophene‐2‐	Butanoic	Octanoic	Dodecanoic	Octadecanoic
		carboxylic acid	carboxylic acid	carboxylic acid	carboxylic acid	acid	acid	acid	acid
		(**14**)	(**15**)	(**16**)	(**17**)	(**18**)	(**19**)	(**20**)	(**21**)
mpCAR	*k*_cat_ [min^−1^]	NA	NA	NA	50±20*	NA	58±1	55±2	3.7±0.3
	*K*_m_ [mm]	NA	NA	NA	50±20*	NA	2.0±0.1	0.09±0.01	0.09±0.02
	*k*_cat_/*K*_m_ [min^−1^ mm ^−1^]	NA	NA	NA	1.1±0.6*	NA	29±2	600±70	39±9
msCAR	*k*_cat_ [min^−1^]	NA	NA	50±10	123±4	129±7*	296±8	131±5	46±4
	*K*_m_ [mm]	NA	NA	13±4	3.3±0.3	7.9±0.8*	0.1±0.01	0.05±0.01	0.6±0.09
	*k*_cat_/*K*_m_ [min^−1^ mm ^−1^]	NA	NA	4±2	37±3	16±2*	3000±300	2700±400	80±10
tpCAR	*k*_cat_ [min^−1^]	23±3	NA	19±1	82±3	82±3*	219±3	157±5	15±1
	*K*_m_ [mm]	24±7	NA	4.7±0.5	3.3±0.3	5.0±0.4*	0.2±0.01	0.04±0.01	0.12±0.03
	*k*_cat_/*K*_m_ [min^−1^ mm ^−1^]	0.9±0.3	NA	4.0±0.4	25±3	17±2*	1140±50	3600±400	120±30
noCAR	*k*_cat_ [min^−1^]	76±4	NA	NA	135±4	170±20*	141±2	99±3	11±1
	*K*_m_ [mm]	20±2	NA	NA	2.6±0.2	50±8*	0.2±0.01	0.04±0.01	0.02±0.01
	*k*_cat_/*K*_m_ [min^−1^ mm ^−1^]	3.9±0.4	NA	NA	52±4	3.4±0.7*	750±30	2500±300	500±300
**niCAR**	*k*_cat_ [min^−1^]	NA	NA	NA	60.8±0.9	260±30*	233±5	157±9	68±7
	*K*_m_ [mm]	NA	NA	NA	1.00±0.05	32±4*	0.2±0.01	0.02±0.01	0.7±0.1
	*k*_cat_/*K*_m_ [min^−1^ mm ^−1^]	NA	NA	NA	58±3	8±1*	1350±90	7000±2000	100±20

NA: no activity was detected with that substrate. *: *K*_m_ was unusually large and substrate concentrations could not reach a high enough concentration to determine kinetic constants accurately. Errors represent the standard error.

### Fatty acids

All the CARs showed a very high catalytic efficiency for C_8_–C_12_ fatty acids (**18**–**21**) with low *K*_m_ values compared to that of benzoic acid (Figure 3 E and Table 4). Octadecanoic acid (**21**), which had a C_18_ carbon chain, showed a similarly low *K*_m_ but a greatly reduced *k*_cat_. All CARs except mpCAR were active against butanoic acid (**18**; Figure 3 E and Table 4) but with a very large *K*_m_, in most cases too large to characterize accurately. In general, mpCAR was much less efficient with fatty acids than the other CAR enzymes.

### Effect of pH

**Figure 4:**
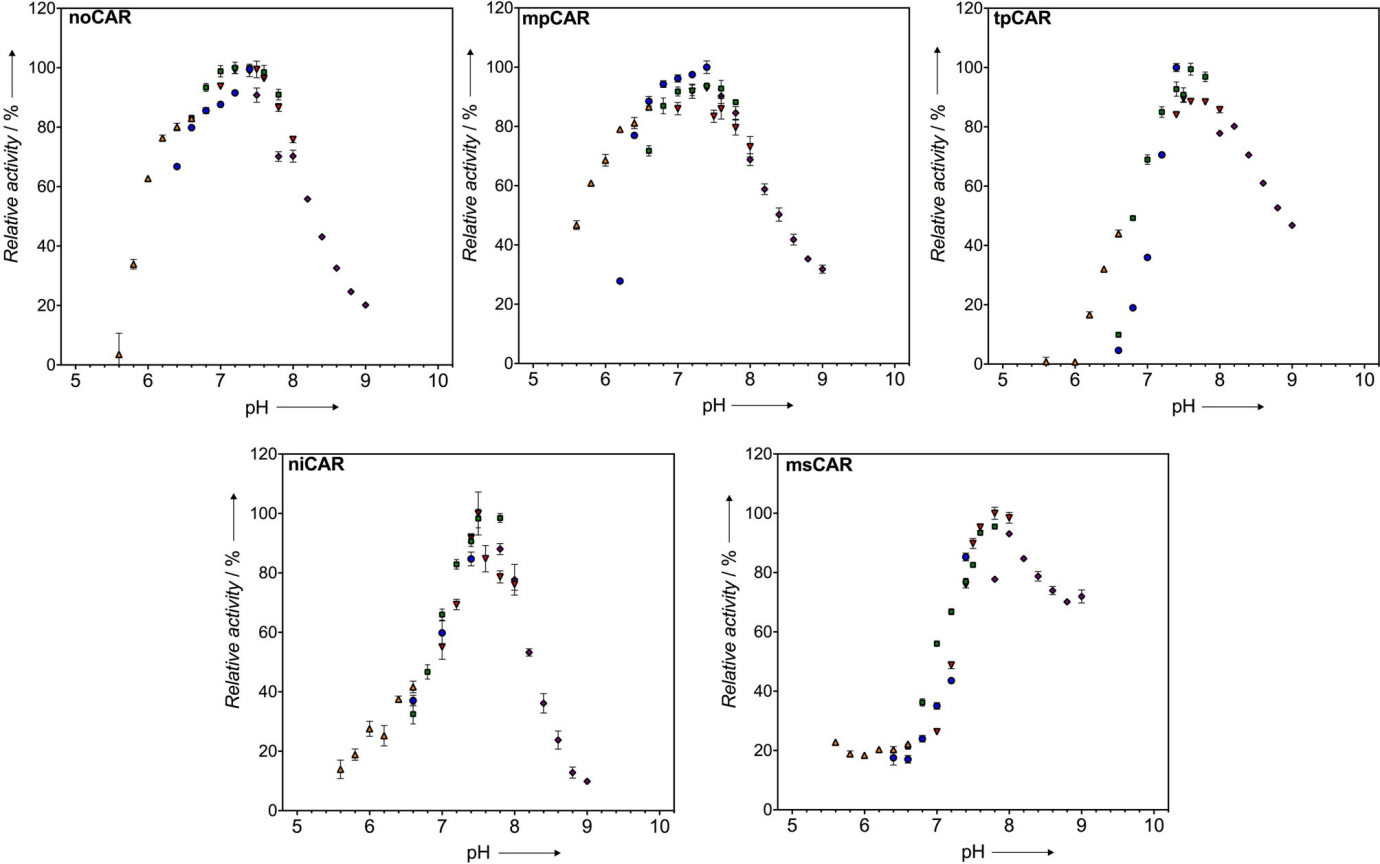
Activity of CAR enzymes in response to pH. Overlapping buffers were used to cover a range of pH 5.6–9.0 in intervals of 0.2 (MES‐NaOH, PIPES‐NaOH, MOPS‐NaOH, HEPES‐NaOH, Tris‐HCl). Activity against 4‐methylbenzoic acid **2** is shown relative to the highest activity at 100 %. Errors bars show the combined standard deviation of three measurements and three blank measurements (with no enzyme) at each pH value.

The activity of an enzyme at different pH values is an important consideration for an industrial enzyme. Therefore, the effect of pH on the CAR activity was examined by measuring activity against benzoic acid at different pH values. mpCAR, niCAR, noCAR, and tpCAR all showed an optimum activity at pH 7.5, whereas msCAR showed an optimum activity at pH 7.8 (Figure 4). Both niCAR and tpCAR showed a sharp peak of activity around pH 7.5 that decreased quickly as the pH moved away from this point. In contrast, mpCAR and noCAR show a slightly broader optimum around pH 7.0–7.6. msCAR behaves very differently from the other CARs. At more acidic pH values (pH 5.5–6.8) it shows very low activity at which the other CARs are more active. However, it is also active at more alkaline pH values at which the other CARs are less active.

### Effect of temperature

**Figure 5:**
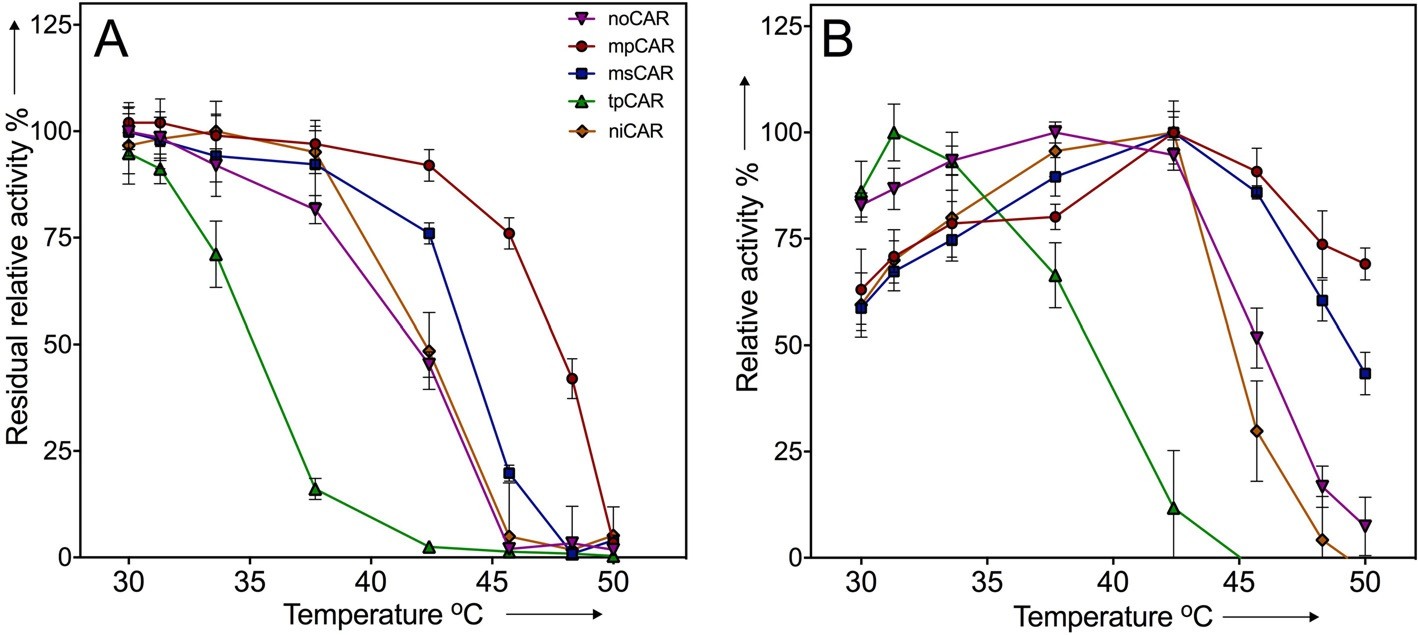
Effect of temperature on CAR enzymes. A) Thermostability of CAR enzymes. The residual activity of CAR enzymes against 4‐methylbenzoic acid after a 30 min incubation at different temperatures. Activity is shown relative to a control sample kept at 4 °C, and the errors bars show the standard deviation of three measurements. B) Activity of CAR enzymes at different temperatures. Activity is relative to the fastest rate at 100 %. Error bars show the combined standard deviation of three measurements and three blank measurements (with no substrate) at each temperature.

The thermostability of the CAR enzymes was investigated by incubating them at various temperatures for 30 min and measuring the residual activity against 4‐methylbenzoic acid relative to a control kept on ice. tpCAR was the least thermostable CAR tested and was completely inactive after 30 min at 42 °C (Figure 5 A). In contrast, mpCAR, a CAR from the moderate thermophile *M. phlei*, retains 92 % of its activity following the same incubation at 42 °C. mpCAR was able to retain residual activity up to 50 °C, which made it the most thermostable CAR identified to date. Both niCAR and noCAR showed intermediate thermostability and were denatured at temperatures beyond 44 °C, whereas msCAR is marginally more thermostable and is able to retain some activity until 47 °C.

The activity at different temperatures was tested in a 10 min reaction. The more thermostable CARs, mpCAR, msCAR, and niCAR all showed an optimum activity of 42 °C (Figure 5 B). Activity decreased past this temperature to various degrees relative to the thermostability of each enzyme. noCAR showed a slightly lower optimum at 38 °C, whereas that of tpCAR was only 31 °C.

The half‐life and degradation constant (*K*_D_) at 30 °C were calculated by measuring the activity at various time points over 120 h. The data were fitted to a one‐phase decay equation by nonlinear least‐squares regression. mpCAR, a CAR from a moderate thermophile, showed by far the longest half‐life at 30 °C of 123.2 h (Table 5). In contrast, tpCAR has a much shorter half‐life of only 25.0 h. The half‐lives of msCAR, niCAR, and noCAR fell between these extremes at 53.7, 42.9, and 35.3 h, respectively. The total turnover numbers (TTN) for the three best substrates were calculated as *k*_cat_/*K*_D_ (Table 5).

**Table 5:** Half‐life and degradation constants (*K*_D_) of CAR enzymes incubated at 30 °C.

Enzyme	Half‐life	*K*_D_	TTN of	TTN of 4‐methyl‐	TTN of 4‐methoxy‐
	[h]	[h^−1^]	benzoic acid	benzoic acid	benzoic acid
mpCAR	123.2	0.0056±0.004	30 000±20 000	20 000±20 000	20 000±20 000
msCAR	53.7	0.013±0.001	15 000±1000	10 000±1000	14 000±1000
niCAR	25	0.28±0.002	350±30	336±8	175±4
noCAR	35.3	0.02±0.002	9000±1000	6800±700	6900±700
tpCAR	42.9	0.016±0.002	9000±1000	6000±1000	8000±1000

The half‐life, *K*_D_, and TTN for the three best substrates of CAR enzymes calculated from activity after incubation at 30 °C over time, fitted to *Y*=*Y*_0_×e^−K*X^. Standard error for *K*_D_ is shown. TTN was calculated as *k*_cat_/*K*_D_, and the combined error is shown.

### Product inhibition

mpCAR was tested for product inhibition with AMP, NADP^+^, and PP_i_. NADP^+^ showed competitive inhibition with NADPH with an inhibition constant (*K*_I_) of (143±8) μm (Figure S13), and AMP was a competitive inhibitor of ATP with *K*_I_=(8200±900) μm (Figure S14). PP_i_ showed a mixed inhibition with ATP with *K*_I_=(220±50) μm and mechanism parameter (α)=(2.5±1.4). Surprisingly, PP_i_ also showed competitive inhibition with 4‐methylbenzoic acid with *K*_I_=(340±40) μm (Figures S15 and S16).

## Discussion

The CAR enzymes offer an excellent opportunity for green chemistry: they offer the opportunity to reduce carboxylic acids selectively to aldehydes without the use of harsh reducing agents. CARs also have a clear advantage over other enzymes that are able to perform this reaction in that the reduced product is thermodynamically favored because of the hydrolysis of ATP. Although a few CARs from different species have been identified previous studies and their activity against diverse acids has been demonstrated, none of these studies has provided a detailed, kinetic comparison of diverse CARs. Therefore, we aimed to characterize some example CARs from across the known CAR family thoroughly, together with the best‐characterized CAR from *N. iowensis*. Our aim was to demonstrate the similarities and differences between these CARs, learn more about the CAR mechanism, and highlight the potential of these enzymes for biocatalysis.

### Effect of electron‐donating or ‐withdrawing groups

Typically, the reduction of carboxylic acids to aldehydes involves a transfer of a “hydride” to the carbonyl unit. Therefore, we expected initially that electron‐withdrawing groups, which make this carbon atom more electrophilic, would be preferred substrates. However, our observation was that, contrary to our expectation, electron‐donating groups were preferred substrates (Figure 3 and Table 2). The addition of electron‐donating groups to benzoic acid resulted in a reduction in *K*_m_ and so an increase in the catalytic efficiency. We reasoned that these groups would drive electrons into the π system to make the first step of the reaction (attack by the negatively charged carboxylate group on the α‐phosphate of ATP) more favorable. As two of the other steps (2 and 4) involve nucleophilic attacks on the acid group carbon atom of the carboxyl group (which should favor electron‐withdrawing groups), this suggests strongly that the first step in the reaction has the greatest impact on substrate specificity and selectivity. It is possible that the reduced *K*_m_ with electron‐donating substituents is a consequence of the more ready formation of the acyl‐AMP intermediate, although very detailed studies of the kinetics of this individual step would be required to confirm this. Indeed, previous studies of NRPSs have shown that the adenylation reaction is a rate‐limiting step.^29^ In long‐chain fatty acid ligases, the acyl‐AMP intermediate has been shown to be unable to leave the active site,^19^ so the addition of a group that likely improves the formation of this intermediate might be expected to cause a lower *K*_M_ and greater catalytic efficiency. Moreover, if benzyl‐AMP was used as a substrate with a CAR from *Nocardia asteroides* it showed a *K*_m_ of 70 nm, compared to *K*_M_=260 nm for benzoic acid, which suggests that this intermediate binds more tightly to the enzyme.^23^ Furthermore, the phosphopantetheine binding and C‐terminal reductase domains show a high sequence identity to that of other ANL superfamily members that process very different substrates. For example, a NRPS from *Mycobacterium intracellulare*, WP_014382786.1, has an average of 58 % identity to the CARs shown in Figure 2 for this C‐terminal region. This suggests strongly that substrate specificity must be determined in the adenylation domain, likely at the formation of the first intermediate.

In the 3‐position (**4**), the methoxy group has no resonance effect on the carboxylic acid and so is actually slightly electron‐withdrawing by induction, as indicated by the Hammett *σ* constant (Table 2). In many of the CARs, the *k*_cat_ of benzoic acids substituted in the 3‐position shows a small reduction compared to that substituted in the 4‐position, and msCAR showed a greatly reduced activity. However, these are still good substrates for most of the CARs. It is likely that there are further interactions between the substrate and the active site binding pocket and that electronic effects alone cannot account for all differences in activity.

Very low or no activity was found with the methoxy‐substituted benzoic acid in the 2‐position (**5**). This suggests that there is a steric interference by the methoxy group on the binding of the nearby carboxylate group to the relevant area of the active site. This effect has been reported for other CARs examined to date with other substituents in the 2‐position. However, in some cases there is activity but at a low level.^22^, ^23^ No structure of a CAR enzyme has yet been described and this would be highly beneficial to understand the effects of groups in the 2‐position.

All substrates with an electron‐withdrawing group (**6**–**8**) showed much lower *k*_cat_ values than benzoic acid, and in most cases inhibited the activity all together. These groups should increase the propensity of the carbonyl carbon atom to nucleophilic attack in steps (2) and (4) of the reaction. Therefore, this suggests strongly again that these two steps are of limited relevance for substrate specificity. Only the nitro substituent in the 3‐position (**7**) showed activity with all the CAR enzymes, likely as in this position the electron‐withdrawing group has no resonance effect on the carboxylate group. As is the case with the methoxy group, it is possible that a nitro substituent in the 2‐position (**8**) inhibits activity because of steric hindrance caused by its close proximity to the carboxylate group.

Previously, it has been reported that benzoic acids substituted in the 2‐position are poor substrates for niCAR, in good agreement with our data.^23^ However, very low activity was observed with **8** previously, which we did not detect. Substrates with the addition of electron‐donating groups to benzoic acid were shown previously to be good substrates for niCAR in agreement with our results. The activity of niCAR with electron‐withdrawing chloro‐ and bromo‐substituted benzoic acids in the 3‐position supports our reasoning that in the 3‐position the absence of a resonance effect allows a better activity with these substrates than that in the other positions.^23^

### Modification of the aromatic ring and unit on CAR activity

Generally, The CARs showed less activity towards heterocycles than a benzene ring. They showed a preference for heterocycles that contained a larger heteroatom or that had a less aromatic nature. In substrate **17**, the lone pairs of electrons in the sulfur atom are more dispersed and less available for bonding, which could result in the lower *K*_m_. In contrast, the nitrogen atom in substrates **14** or **15** has lone pairs that are more available for bonding, which may result in the very large *K*_m_ values and lack of activity observed. Substrate **16**, which has an oxygen atom in the heterocycle, sits between these substrates in both respects.

The extension of the carboxylate group away from the aryl group in **9** disrupts its influence on the carboxylic acid to make a less sterically rigid substrate. This difference seems to have made the carboxylic acid group more accessible as the *K*_m_ is much lower than that of substrate **1** in most cases. In contrast, the inclusion of a double bond in substrate **10**, should withdraw electrons from the carboxylic acid group. This would be beneficial for nucleophilic attack on the carbonyl in steps (2) and (4) of the reaction but detrimental to the initial attack by the oxygen atom of the carboxylate group on ATP. The result is a significant decrease in *k*_cat_ compared to that of substrate **1**. The double bond also makes the molecule more rigid in an apparently favorable conformation as the *K*_m_ is even smaller than that of **9**. If a triple bond is added to the substrate (**11**), the molecule is very rigid and flat with a more electron‐deficient carboxylic acid group. These effects together reduced activity in nearly all the CARs. The presence of a β‐ketone group on the β‐carbon atom of substrate **9** will have a similar effect to the inclusion of a double bond in **10**, which results in the very weak withdrawal of electrons from the carboxylic acid group. The ketone group (**12**) had mixed effects on the *K*_m_ for the various CARs, which suggests that different interactions take place with the ketone group within the active sites of the enzymes. These observations agree with the hypothesis that the first step of the proposed reaction mechanism is rate limiting.

### Fatty acids

Fatty acids are interesting substrates as fatty alcohols can be used as biofuels and in detergents, surfactants, and polymers.^7^ As was observed for the CAR from *Mycobacterium marinum* (mmCAR), most of the CARs tested were active against C_4_–C_18_ fatty acids, and we observed similar kinetics to that obtained previously.^10^ The catalytic efficiency with substrate **18** (C_4_) was very poor, primarily because of the large *K*_m_ values for this substrate, which suggests that it might be too small to make the necessary interactions in the active site of the adenylation domain. However, larger fatty acids showed much lower *K*_m_ values and high turnover numbers to result in catalytic efficiencies higher than that of any of the aromatic substrates tested in many cases. As the acyl chain length increased past substrate **19** (C_8_), *k*_cat_ decreased and reached a low residual level for substrate **21** (C_18_). Both niCAR and msCAR showed a higher turnover number with substrate **21** than the other CARs, which suggests that these enzymes might be better suited to larger substrates. Recently, two other CARs, in addition to niCAR and mmCAR, have been shown to have activity against ethanoic, butanoic, 2‐methyl butanoic, and 2‐oxobutanoic acids, which highlights that CARs can accept small fatty acids and that they can tolerate the addition of groups such as a methyl or carbonyl on the α‐carbon atom. However, 2‐aminobutanoic acid was tested but showed no activity.^11^

### Effects of pH and temperature

The operating pH and temperature range of an enzyme is an important consideration for a potential biocatalyst. Stability at extremes of pH and in solvents are characteristics often found in thermostable proteins as the mechanisms that stabilize these proteins against high temperature can also stabilize against these other conditions. We observed an optimum pH of 7.5 for four of the five CARs tested with a general tolerance to acidic pH, consistent with data reported previously on the activity of other CARs.^22^ In particular, both mpCAR and noCAR were able to tolerate pH 6 with only a small loss of activity (whereas other CARs showed a much narrower optimum). In contrast, msCAR is clearly better suited to more alkaline pH values (Figure 4). Therefore, this offers a CAR suitable for use in biocatalysis in conjunction with other enzymes that favor a similar alkaline pH.

mpCAR showed by far the best thermostability of any characterized CAR (Figure 5 and Table 5). We also observed that it shows a much lower catalytic efficiency in general than the other CARs at 30 °C (Tables 3–4). Possibly, there has been a trade‐off between the rigidity of the enzyme (which provides thermostability) and flexibility to allow a broader substrate range. Notably, the rate enhancement in mpCAR at its optimum temperature compared to that at 30 °C was a little greater than that for other CARs (Figure 5 B). In contrast, tpCAR shows very low thermostability (Figure 5) but is active with many of the substrates that the other CARs could not turn over (e.g., **5**, **6**, **11**, **14**). A possible compromise enzyme is msCAR, which shows the next best thermostability and has a generally good catalytic efficiency. If an enzyme is chosen for industrial use, the lifespan of the enzyme is an important consideration. The TTN can be calculated as a measure of how effective an enzyme will be over its lifetime, which we have demonstrated with three of the best CAR substrates (Table 5). In this respect, the most thermostable CARs have a clear advantage in that the TTN of these enzymes will be much greater.^30^ We observed that the lifespan of the enzyme at 30 °C (Table 5) mirrored the thermostability of the enzymes exactly (Figure 5), which suggests that a test of thermostability will be a good predictor of lifespan for CARs.

The CAR enzymes in this study show only moderate thermostability. To date, no CAR enzymes have been identified in any thermophilic organisms. A thermostable CAR enzyme would be attractive for use industrially as this enzyme would likely be resistant to other denaturing forces, such as extremes of pH or organic solvent, and likely offer a higher TTN for use in in vitro reactions.

### Product inhibition and reaction mechanism

mpCAR was inhibited by most of its reaction products, and we assume that the other CARs share this inhibition. It is unsurprising that NADP^+^ acts as a competitive inhibitor of NADPH (Figure S13) as NADP^+^ is likely also able to bind to the Rossmann fold of the reductase domain. AMP acts as a competitive inhibitor against ATP (Figure S14), likely because they are very similar molecules. AMP is also a competitive inhibitor of ATP in long‐chain fatty acid CoA synthetases, in which the adenylation domain shows a significant homology to the CAR adenylation domain.^31^

**Figure 6:**
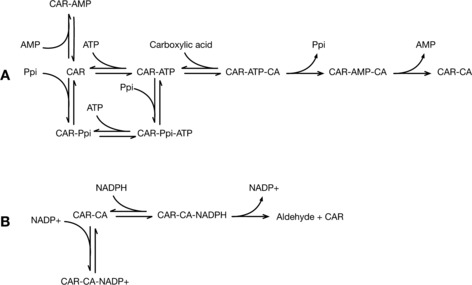
Model for the binding of substrates and inhibitors to the CAR enzyme. A) Binding and release of substrates, products, and inhibitors in the adenylation domain. The final result is the formation of a thioester intermediate with the phosphopantetheine arm, represented by CAR‐CA. B) The phosphopantetheine arm can then transfer CA to the reduction domain, in which it is reduced by NADPH to release the aldehyde product.

PP_i_ showed mixed inhibition against ATP but competitive inhibition against 4‐methylbenzoic acid (Figures S15 and S16). This pattern of inhibition is characteristic for ordered sequential bisubstrate reactions.^32^ This indicates that ATP is the first to bind to the adenylation domain and is then followed by a carboxylic acid. Long‐chain fatty acid CoA synthetases show the same ordered binding of these substrates.^19^ Therefore, we propose a model for the ordered binding of substrates and inhibitors to the CAR enzyme based on these results (Figure 6). Interestingly, although PP_i_ is a product of ATP, its activity as an inhibitor shows that it binds preferentially to the carboxylic acid binding site. Therefore, CARs might need to be combined with other enzymes such as phosphite dehydrogenase^33^ or inorganic pyrophosphatase^29^ to overcome product inhibition in an industrial process. Indeed the in vitro turnover of niCAR was improved by the addition of an inorganic pyrophosphatase enzyme.^34^

### CAR phylogeny and insight into the adenylation step

Here we have provided the first glimpse of CAR evolution within the *Actinomycetes*. From the phylogeny, we hypothesize that the CARs may have propagated through the *Nocardia* and *Mycobacteria* by a series of early horizontal transfer events. This is most apparent in *M. smegmatis*, which possesses three CAR paralogues that cluster in two distinct Mycobacterial clades. Additionally, it is apparent that a large amount of change has occurred within the *Tsukamurella*. This could reflect the slightly more promiscuous substrate range of tpCAR.

We presented evidence that the adenylation domain of the CARs belongs to the ANL superfamily of enzymes because of the presence of conserved hallmark motifs. Similarly, both NRPSs and the acyl‐CoA synthetases use an acyl group to form a thioester between a substrate and a pantetheine thiol, which supports this interpretation. Furthermore, the NPRSs mobilize their substrate following the thiolation of a phosphopantetheine arm bound to a *holo*‐acyl carrier protein domain. Parallels can be drawn between both the above reactions and the proposed mechanism of CAR activity presented in Figure 1. This offers the opportunity to exploit the extensive studies on the ANL superfamily to gain an insight into the finer details of the mechanism of carboxylic acid reduction employed by CARs.

In particular, ANL superfamily members are further partitioned into two subdomains: a large (∼450 aa) N‐terminal domain and a small (∼100 aa) C‐terminal domain, connected by a flexible linker. Crystal structures show that the substrate binding pocket is formed by the N‐ and C‐domain interface. Substrate adenylation proceeds in a two‐step manner, in which the active site undergoes large conformational changes because of a ∼140° rotation of the C‐terminal domain following the formation of an acyl‐bound intermediate and the release of PP_i_. Within NRPSs and acyl‐CoA synthetases, the second domain architecture facilitates the thiolation of the phosphopantetheine. Lysines that are required within each active site are positioned on opposing faces of the C‐terminal domain and are conserved within the CARs (Figure S1).^35^ This suggests that the CARs also undergo characteristic ANL superfamily domain alteration between steps (1) and (2) (Figure 1) to catalytically isolate the adenylation and thioester‐forming reactions.^18^

## Conclusions

Carboxylic acid reductases (CARs) are proposed as a useful tool for biocatalysis. This study has demonstrated that, across the entire extant phylogeny of CARs, similar substrates are preferred by this family of enzymes, and some enzymes are more promiscuous than others. In particular, our detailed kinetic analysis of CARs suggests strongly that the first step in the proposed reaction mechanism, during which an adenosine monophosphate (AMP) carboxylic acid phosphoester intermediate is formed with the release of pyrophosphate (PP_i_), is critical to determine suitable substrates. Consequently, the addition of groups that donate electrons, which makes the oxygen atom of the carboxylic acid more electronegative, will be better substrates; and aliphatic acids are preferred strongly to aromatic acids. This study also highlighted that, similar to that of other members of the ANL (Acyl‐CoA synthetase/NRPS adenylation domain/Luciferase) superfamily to which the CARs belong, this first step is an ordered sequential Bi Bi (2 reactants, 2 products) reaction, in which adenosine triphosphate is bound before the carboxylic acid. Of particular relevance to biocatalysis is that all of the byproducts of the reaction (PP_i_, AMP, and NADP^+^) appear to be inhibitors: for the use of CARs in vitro there is a need to remove or regenerate these. These data further validate CARs as a useful tool for new biocatalytic reactions and highlight their potential if integrated with other enzymes in vitro for the efficient reduction of carboxylic acids to aldehydes.

## Experimental Section

### Alignments and phylogeny construction

Unless specified, all algorithms were performed under default settings. We retrieved 48 sequences by homology search in BLAST to the *N. iowensis* CAR. Alignments were performed using the MUSCLE plug‐in within Geneious version 9.1 (http://www.geneious.com).^36^ Sequence masking was conducted with the Gblocks algorithm within the Phylogeny.fr online tool (http://www.phylogeny.fr).^37^ ProtTest (version 3.4)^38^ analysis of the aligned dataset was performed in the command line. MrBayes (version 3.2.6)^39^ was run in the command line as follows: the amino acid substitution model was fixed to WAG with a γ‐distributed rate variation across a proportion of invariable sites and eight γ categories. The analysis was run for 1 000 000 MCMCMC generations, and every 100 generations were sampled with two parallel runs and four chains (which contained one heated chain of temperature 0.2) with a burn‐in of 25 %. Trees were visualized, midpoint rooted, and modified in FigTree version 1.4 (http://tree.bio.ed.ac.uk/software/figtree/).

A more complete list of 124 CAR homologues was retrieved (Figures S17 and S18). However, a reduced set of sequences was used as this allowed the construction of a more reliable phylogeny.

### Expression and purification

CAR genes (except niCAR) were cloned into expression vectors pNIC28‐Bsa4^28^ or obtained from Prozomix, cloned into pET28a (Novagen). A pET plasmid for the expression of niCAR was obtained from Andrew Hill (University of Manchester). All contained a N‐terminal 6x histidine tag.^28^ Full sequences for all vectors are supplied as Supporting Information. Vectors were transformed into BL21 (DE3) *E. coli* along with a pCDF‐Duet1 vector that contained a phosphopantetheine transferase from *Bacillus subtilis* for its coexpression with the CARs. Expression was performed in LB medium with the addition of 50 μg μL^−1^ each of kanamycin and spectinomycin. Cells were grown to approximately 0.6 OD_600 nm_ at 37 °C with shaking at 225 rpm, at which point IPTG was added to a concentration of 150 μm and the temperature was decreased to 20 °C for protein expression overnight. Cells were harvested by centrifuging and resuspended in 25 mm Tris‐HCl pH 8.0 and 0.5 m NaCl. The cell lysate was prepared by sonication on ice followed by centrifugation to remove the insoluble fraction.

CARs were purified from the cell lysate by using a 1 mL His‐Trap FF crude column (GE Healthcare) using an elution gradient from 10 to 250 mm imidazole in 25 mm Tris‐HCl pH 8.0 and 0.5 m NaCl. The purified sample was then applied to a Superdex 200 HiLoad 16/60 gel filtration column (GE Healthcare) and eluted in 25 mm HEPES, pH 7.5, 0.1 m NaCl at 1.0 mL min^−1^. Eluted fractions were analyzed by using SDS‐PAGE before they were pooled and concentrated to approximately 2 mg mL^−1^. To calculate the protein concentration from OD_280 nm_, an extinction coefficient and molecular weight for each enzyme was calculated by using the ExPaSy ProtParam tool (Figure S19). Yields of approximately 2–10 mg purified protein per liter of culture were obtained, from 2–4 L of culture prepared per batch. Single‐use aliquots of protein were stored at −80 °C.

### Standard enzyme assay

Unless otherwise specified, assays were performed in 100 mm Tris‐HCl pH 7.5 prepared at 30 °C, 1 mm ATP, 0.25 mm NADPH, 10 mm MgCl_2_, 2–6 μg of purified CAR enzyme and 5 mm carboxylic acid substrate in a total volume of 200 μL. Carboxylic acid substrates were prepared in DMSO at 500 mm. The oxidation of NADPH was used to monitor the reactions by measuring the absorbance of NADPH at *λ*=340 nm. Reactions were performed in triplicate in a 96‐well microtiter plate by using a Tecan M200 plate reader at 30 °C over 5 or 10 min after a 5 min preincubation at 30 °C. If convenient, an EpMotion 7050 (Eppendorf) liquid handling robot was used to set up the assays.

### Kinetic analysis of substrate specificity

Kinetic analysis was performed by picking eight appropriate substrate concentrations around an approximate *K*_m_ value for each substrate and measuring initial rates as described previously. Rates were fitted to the Michaelis–Menten equation by nonlinear least‐squares regression by using GraphPad Prism 5.0. To calculate constants for ATP and NADPH, 5 mm (*E*)‐3‐phenylprop‐2‐enoic acid was used as the carboxylic acid substrate, except for niCAR for which 5 mm 4‐methylbenzoic acid was used.

### pH vs. activity

Buffers were prepared and titrated to the correct pH using NaOH or HCl at 30 °C to cover pH values in intervals of 0.2. The buffers 50 mm MES pH 5.6–6.6, 50 mm PIPES pH 6.4–7.4, 50 mm MOPS pH 6.6–7.8, 50 mm HEPES pH 7.0–8.0, and 50 mm Tris pH 7.8–9.0 were used. Reactions were performed as standard with 1 mm ATP, 0.25 mm NADPH, 10 mm MgCl_2_, 2–6 μg of purified CAR enzyme, and 5 mm 4‐methylbenzoic acid. Blanks that contained no enzyme were used to subtract a blank rate at each pH value. Initial rates were calculated as the relative activity against the fastest result at 100 %.

### Thermostability

A solution that contained 2 μg of purified enzyme, 0.25 mm NADPH, 1 mm ATP, 10 mm MgCl_2_, and 100 mm Tris‐HCl pH 7.5 was incubated across the temperature gradient of a Biorad thermocycler from 30 to 50 °C for 30 min. The sample was cooled and assayed for CAR activity against 4‐methylbenzoic acid and compared to a control sample that remained on ice.

### Degradation at 30 °C

We used 2 mL samples at 2 mg mL^−1^ in 25 mm HEPES, pH 7.5, 0.1 m NaCl that were incubated at 30 °C over 120 h. At specified time intervals, samples were taken and assayed for enzyme activity against 4‐methylbenzoic acid. Rates were calculated relative to the first reading at 100 % and fitted to a model of first‐order thermal deactivation by using the equation *Y*=*Y*_0_×e^−K* *X*^ in which *Y* is the relative activity and *X* is the time [h].

### Temperature vs. activity

Tris‐HCl pH 7.5 (100 mm) was prepared at assay temperatures between 30 and 50 °C. Assays were performed as for the thermostability experiment using the temperature gradient of a Biorad thermocycler from 30 to 50 °C over the course of 10 min, before the sample was cooled rapidly on ice with the addition of 10 mm NaOH. A blank reaction with no substrate was used to calculate the NADPH used in the reaction. Activity was calculated relative to the maximum rate at 100 %.

### Product inhibition

Potential inhibitors were titrated across a broad range of concentrations to determine whether inhibition occurred and to give an idea of an approximate *K*_I_. Kinetic analysis then was performed as described above using substrates that each inhibitor was likely competitive against with the addition of the inhibitors at a range of concentrations based around the approximate *K*_I_. Data were fitted by using GraphPad Prism 5.0 by nonlinear least‐squares regression to different models of enzyme inhibition. The model with the best fit for the data was used to determine the mode of inhibition. If inhibition was not competitive, additional analysis was performed with other substrates.

## Supporting information

As a service to our authors and readers, this journal provides supporting information supplied by the authors. Such materials are peer reviewed and may be re‐organized for online delivery, but are not copy‐edited or typeset. Technical support issues arising from supporting information (other than missing files) should be addressed to the authors.

Supplementary
