# Supplementary material for: Characterization of Carboxylic Acid Reductases as Enzymes in the Toolbox for Synthetic Chemistry
Source: ChemCatChem. 2017 Feb 14;9(6):1005–17. doi: 10.1002/cctc.201601249 (PMC5396282; doi:10.1002/cctc.201601249)
Supplement: Supplementary file 1 — Supplementary [file CCTC-9-1005-s001.pdf]

Heterogeneous & Homogeneous & Bio- & Nano-
CHEMCATCHEM
CATALYSIS

Supporting Information

Characterization of Carboxylic Acid Reductases as Enzymes in the Toolbox for Synthetic Chemistry

William Finnigan,[a] Adam Thomas,[a] Holly Cromar,[a] Ben Gough,[a] Radka Snajdrova,[b] Joseph P. Adams,[b] Jennifer A. Littlechild,[a] and Nicholas J. Harmer*[a]

cctc_201601249_sm_miscellaneous_information.pdf

Supporting Information

	tpCAR	msCAR	niCAR	noCAR	mpCAR
tpCAR	100	48	49	51	47
msCAR	48	100	59	57	57
niCAR	49	59	100	65	63
noCAR	51	57	65	100	59
mpCAR	47	57	63	59	100

Supplementary table 1: Sequence identity of CAR orthologues in this study. Pairwise sequence identities were calculated using BLAST.

WILEY-VCH

```
CLUSTAL multiple sequence alignment by MUSCLE (3.8)

Conserved motifs between adenylating domains of Actinomycete CARs, FadD13 and NPRS
p=medium aliphatic amino acid. r=aromatic amino acid. x=any amino acid.

Firefly_Luciferase|BAL46512.1       ---------------------MEDAKNIKKGPAPFYPLEDGTAGEQLHKAMK--------
FadD13|NP_217605.1                  --------------------------------------------------MKNIGWMLRQ
NRPS|WP_014382786.1                 ----------------------------------------------------SIPALFAA
Tsukamurella_paurometabola          ---MSIET------------------VQNGVPAEGSVPPADQQTERLPQVIARIFAQFAD
Mycobacterium_smegmatis1            ---MTSDVHDEQSTRRIAELYATDPEFAAAAPLPAVVDAAHKPGLRLAEILQTLFTGYGD
Mycobacterium_genavense             ---MTSDNRDERTARRVAELFNNDPQFRAAAPLPEVIEAACAPGLRLTEVLARLVEGYAD
Mycobacterium_marinum               MSITCVDTRAQRSARRIEQLYSTDAQFAAARPSTAVGIAISKSGLGLPQIIQTVMDGYPQ
Nocardia_asteroides                 ---MTVEVDADRLADRIRALYAQDAQIRAATPIPEAHARVTTPGTPLARIVSTVMTAYAD
Nocardia_paucivorans                ---MSVDTRESRLERRIAELYATDEQFAAARPDVAVTAEVERAGRRSARVVHAVAKGYAH
Mycobacterium_fortuitum             ---MSFDTRDEQLATRIADLTATDPQFAAAIPSDTVTASVDVPGLLLPEIVQRVLEGYAE
Mycobacterium_smegmatis2            ---MTIETREDRFNRRIDHLFETDPQFAAARPDEAISAAAADPELRLPAAVKQILAGYAD
Mycobacterium_vaccae                ---MSTDTREGRLARRIADLFATDPQFAAAVPDETVAAAVEEHAAHLPDIMRTVLDGYAD
Mycobacterium_obuense               ---MPTDTREERLAHRIDDLSATDTQFAAALPDEAIAEAIEDPQLRLPQIIATVLDGYAD
Nocardia_seriolae                   ---MVEDTARAEIHRRIAERVLADEQVRVAMPLPEVSEAARQPGLGLARAVEVLMVGYAE
Nocardia_otitidiscaviarum           ---MLDDARAERRERRIADALADD-QVREAAADAAVSESVRRVEVRLARIVDAVMSGYGD
Nocardia_iowensis                   ---MAVDSPDERLQRRIAQLFAEDEQVKAARPLEAVSAAVSAPGMRLAQIAATVMAGYAD
Nocardia_transvalensis              ---MEITDAQAQLIRRATELIEGDEQVRAALPDEAVAKAVQAPGLGLASVVATIMEGYAD

                                                   p(S/T)rx(E/Q)p N-terminal helix cap (A1)
Firefly_Luciferase|BAL46512.1       RYALVPGTIAFTDAH-----------IEVNITYAEYFEMSVRLAEAMKRYGLN-TNHRIV
FadD13|NP_217605.1                  RATVSPRLQAYVEPS-----------TDVRMTYAQMNALANRCADVLTALGIA-KGDRVA
NRPS|WP_014382786.1                 QVARGPGAVAITCGG-------------RSFTYRHLYEATNRLAHLLVERGAG-PGQRVA
Tsukamurella_paurometabola          RPAFATREAGPGTP-------------YATVSYREIWRRVTALVASWQSE-VA-PGDFVA
Mycobacterium_smegmatis1            RPALGYRARELATDE-GGRTVTRLLPRFDTLTYAQVWSRVQAVAAALRHNPIY-PGDAVA
Mycobacterium_genavense             RPALGERVRELVTDA-DGRTVLRLRPRFETISYRDVWDRVRAIATAWSSDPVT-AGDVVA
Mycobacterium_marinum               RPALGQRATRVVTDPNTGRSSAQLLAEFETITYRELWNRTNALTNAFAAEALADRGQRVC
Nocardia_asteroides                 RPALGVRRTELVVEA--GRATRRLLPEFELLTYGEVWERARALAASWYAEGLA-AGEFVA
Nocardia_paucivorans                RPALGQRAVDYITDPRTGRTSMELLPRYETLTYREVWERAGAIASALAGNPVR-AGDRVC
Mycobacterium_fortuitum             RPALGERALEFVADPATGRTTARLLPRFDTISYGQVWDRVRALAAALHASGVA-AGDRVA
Mycobacterium_smegmatis2            RPALGKRAVEFVTDE-EGRTTAKLLPRFDTITYRQLAGRIQAVTNAWHNHPVN-AGDRVA
Mycobacterium_vaccae                RPALARRAVRFVEDA-TGRTVAELLPHFETITYAELAHRIHGVTAALTD--VH-PGDRVA
Mycobacterium_obuense               RPALGQRAVRLVADPHTERTEAQLLPHFDTITYGELSTRIHHLLTALTD--VD-PGDRVA
Nocardia_seriolae                   RPAIGERAAEIVTGA-DGRRIRRLLPEYRTITYAELWSRAGAIAAAWQHDPLR-AGDFLC
Nocardia_otitidiscaviarum           RAALAWRRSELVDGA------VRLLPEYSTMTYRELWRQAGAVAAEWGADPVR-AEDFVC
Nocardia_iowensis                   RPAAGQRAFELNTDDATGRTSLRLLPRFETITYRELWQRVGEVAAAWHHDPLR-AGDFVA
Nocardia_transvalensis              RPAAGRRAVEFVADD-SGRRHARLLPRYDTITYGELWERVRALMAAWHHDPTR-AGDFVA

                 structural element (R/K/F)pGp poorly conserved with ANL superfamily (A2)
Firefly_Luciferase|BAL46512.1       VCSENSLQFFMPVLGALFIGVAVAPANDIYNERELLNSMNISQPTVVFVSKKGLQKILNV
FadD13|NP_217605.1                  LLMPNSVEFCCLFYGAAKLGAVAVPINTRLAAPEVSFILSDSGSKVVIYGAPSAP-----
NRPS|WP_014382786.1                 VAVPRSAEAIVAILAVLKTGAAYVPIDPSVPAARVQFVLGDAAPIAAVTTAEV-------
Tsukamurella_paurometabola          ILGFTSSDFVTVDLATTLLGAPNVPLQAGAPAARIATILDETRPKILAVSADQVDLAQEA
Mycobacterium_smegmatis1            TIGFASPDYLTLDLVCAYLGLVSVPLQHNAPVSRLAPILAEVEPRILTVSAEYLDLAVES
Mycobacterium_genavense             TVGFSSADYLVVDLVCAYLGLVTVPLQHNAPPARLRPIIEECEPKIVAVSAEYLDLAAES
Mycobacterium_marinum               VLGFASIDYATIDLALMLLGAVSVPLPTNAARAQLCHIVSETQPSLIASSTENLPDAISL
Nocardia_asteroides                 TLGFTGADYTVLDLATIHLGAVAVPLQAGASATQLRSILDETAPRVLAVDTANLAVALDV
Nocardia_paucivorans                ILGFASVDYTTIDMALTRLAAVSVPLQTGAPAERLRPIIAETEPTVLAASIDHLDDAVTA
Mycobacterium_fortuitum             ILGFTSADYTVIDTALGQIGAVSVPLQTSSSPEALAPIVTETEPRVIAASVDHLADAVEL
Mycobacterium_smegmatis2            ILGFTSVDYTTIDIALLELGAVSVPLQTSAPVAQLQPIVAETEPKVIASSVDFLADAVAL
Mycobacterium_vaccae                LLGFTSVDYTTVDMALSMLGAVLVPLQTSAPLSTLRPIIAETEPVLIASSVDTLDDAVAL
Mycobacterium_obuense               ILGFTSVDYTVIDTTLVLRGAVSVPLQTSAPAATLRPIVAETEPVVFAASVDHLSDAVDL
Nocardia_seriolae                   VLGFGSGDFAALEIAAIRQGLVTVPLQANAAAAQWRSIIEETGARTLAVSLELLDSALDV
Nocardia_otitidiscaviarum           TLGFTSPDYTVVDLALMRLAAVAVPLQASASVAQWRSIMAETEPRMLAASAETLPAAVEA
Nocardia_iowensis                   LLGFTSIDYATLDLADIHLGAVTVPLQASAAVSQLIAILTETSPRLLASTPEHLDAAVEC
Nocardia_transvalensis              ILGFTGIDYTVVDLACACLGAVSVPLQAGASLAQLTPIAAETEPRVLATDIEQLGAGVDL


Firefly_Luciferase|BAL46512.1       QKKLPIIQKIIIMDSKTDYQGFQSMYTFVTSHL--PPGFNEYDFVPESFDRDKTI-----
FadD13|NP_217605.1                  ------------------------VIDAIRAQADPPGTVTDWIGADSLAERLRSA-AADE
NRPS|WP_014382786.1                 ---------------RPQLGGF-------------AGQIIDIDDPAVVRQPATGL-PVPS
Tsukamurella_paurometabola          LAESAATPRVVVFD----------------------GERDGYEGIEADILSGSAL-PAPE
Mycobacterium_smegmatis1            VRDVNSVSQLVVFDHHPEVDDHRDALARAREQL--AGKGIAVTTLDAIADEGAGL-PAEP
Mycobacterium_genavense             ALTSTSLRQLMVFDYRAEVDEQRENFEQTRVRLQGSGTRVAVTTVDEVVARGRRL-PAVA
Mycobacterium_marinum               VLSHRAPHRVVVFDYRPELDAHREALEAARARL--AAIPVTVETLTAIIARGRTVRPAEA
Nocardia_asteroides                 VLAGAAPRALVVFDHHADDDNDREVLAAARARLRAANSPIVLSTVAEVIDRGRLA-DPAP
Nocardia_paucivorans                VLAGHLPARLIVFDYHPRVDDQREAFAAATEKLAEAGGPVVIETLAEVIDRGTAL-PPVP
Mycobacterium_fortuitum             ALTAHAPAQLVVFDHHPEIDDHREAVASAAERITAAGASIAVDTLAGLLDRGSNL-PAPE
```

```
Mycobacterium_smegmatis2          VESGPAPSRLVVFDYSHEVDDQREAFEAAKGKL--AGTGVVVETITDALDRGRSL-ADAP
Mycobacterium_vaccae              ALDAPDAARLVVFDHRAEVDDHRDALTSATARLRAAGSPLEIETLAEVIARGSTM-PARE
Mycobacterium_obuense             VADAESVGRLIVFDYRAEVDDHRDAIADARARLADAGRSIEIVTLSEVLAHGATL-PAAQ
Nocardia_seriolae                 VLDGSPVTSIVVFDFEPEEDRQAEILVGARDRIAASGSTITLESLAAVLERGATL-PAVP
Nocardia_otitidiscaviarum         VLGGFAPRRVLVFDYRPELEAHRSAVDSARERLAEVG--CTVATVADAVDRGANL-P-AP
Nocardia_iowensis                 LLAGTTPERLVVFDYHPEDDDQRAAFESARRRLADAGSLVIVETLDAVRARGRDL-PAAP
Nocardia_transvalensis            VLSGDSVRSVVVFDYYAEDDDHRAALESARARL--ADSPVTVDTLDEVLARGRDL-PAAP
```

Phosphate binding loop ppx(S/T)(S/T/G)G(S/T)TGxPK (A3)

```
Firefly_Luciferase|BAL46512.1     ----------ALIMNSSGSTGLPKGVALPHRTA---CVRFSHARDPI-FGNQIIPDTAIL
FadD13|NP_217605.1                PAVECGGDDNLFIMYTSGTTGHPKGVVHTHESVHSAASSWASTIDVR-YRDRLLLP----
NRPS|WP_014382786.1               A------DSIAYIIYTSGTTGTPKGVAVTHSNV---TQLLESIDAQL------DVGQVWT
Tsukamurella_paurometabola        FFAEPGTDPLVTLIYTSGSTGTPKGAMYTEQLV---RDAWLKVDSIV---DIDMPAESLL
Mycobacterium_smegmatis1          IYTADHDQRLAMILYTSGSTGAPKGAMYTEAMV---ARLWTMSFITG-----DPTPVINV
Mycobacterium_genavense           ACADGDDQRLAMIMYTSGSTGTPKGAMYTERTV---TTVWTTMRFLA-----PGLPVINA
Mycobacterium_marinum             DCGAQSADAPALLIYTSGSTGAPKGVVYTRNRV---ADFWRTSKAEVEATEQRTAPSITL
Nocardia_asteroides               LVPAPQDDPLAMLIYTSGSTGTPKGAMYTDRLV---AAGWQPAR---------PVAVLNV
Nocardia_paucivorans              LAPAEEPDSLALLIYTSGSTGAPKGAMITDRMV---AEHWRAATAER-WGQRGTEPSIVL
Mycobacterium_fortuitum           APKADGSDPLALLIYTSGSTGAPKGAMYLQSAV---AKFWRRNSKAW---LGPVSSAINL
Mycobacterium_smegmatis2          LYVPDEADPLTLLIYTSGSTGTPKGAMYPESKT---ATMWQAGSKAR-WDETGVMPSITL
Mycobacterium_vaccae              QF-SPDADTLMLLIYTSGSTGAPKGAMYTERLV---ATTWRRSSRSF-WGDHGPLPSITL
Mycobacterium_obuense             PFTSPDDDPLLLLIYTSGSTGAPKGAMYPERLI---TNAWRRSGRSA-WGGEQTTPSITL
Nocardia_seriolae                 LHVPADEDEVALLIYTSGSTGTPKGAIYPHRLV---TGMWLGPNV-------IPAPVMNF
Nocardia_otitidiscaviarum         LRIPSDRERLALLIYTSGSTGAPKGAMYTDRLV---AGLWLSANEIR-------VPALTM
Nocardia_iowensis                 LFVDTDDDPLALLIYTSGSTGTPKGAMYTNRLA---ATMWQGNSMLQ---GNSQRVGINL
Nocardia_transvalensis            LHTDGDEDELSLLIYTSGSTGTPKGAIYPARLL---TRMWRSGGD-------RPMPVLGF
```

possible active site aromatic residue (A4)

```
Firefly_Luciferase|BAL46512.1     SVVPFHHGFGMFTTLGYLICGFRVVLMYRFEEELFLRSLQDYKIQSALLVPTLFSFF---
FadD13|NP_217605.1                --LPMFHVAALTTVIFSAMRGVTLISMPQFDATKVWSLIVEERVCIGGAVPAILNFMRQ-
NRPS|WP_014382786.1               QCHSLAFDFSVWEVFGALLHGGRLVVVPDDVVRSPDDLRALLVREQVSVLSQTPSAFY--
Tsukamurella_paurometabola        HFLPMSHMYGRNWLIAGLASGGTGYFAGASDMSTLFDDLAAARPTAIGLVPRVCELIHQR
Mycobacterium_smegmatis1          NFMPLNHLGGRIPISTAVQNGGTSYFVPESDMSTLFEDLALVRPTELGLVPRVADMLYQH
Mycobacterium_genavense           NFMPLNHLGGRLPLASAFLSGGTSYFVPESDLSTLFEDWALVRPTEAAMVPRVVEMLYQH
Mycobacterium_marinum             NFMPMSHANGRQVLYGTLSNGGTAYFTARSDLSTLFDDLALVRPTELGFPPRIWDMLLER
Nocardia_asteroides               NFLPMSHIAARLTLNGVLARGGTAYFTAAADMSTLFEDIALVRPTEIFLVPRVCDMLLHR
Nocardia_paucivorans              GFMPMSHILGRAICWMALGSGGTVYFAAKSDLSTLLDDLALVRPTQLTFVPRVWEMLFQR
Mycobacterium_fortuitum           SFMPMSHVMGRGILYASLAAGGTCYFAARSDLSTLLEDLALTRPTELNFVPRVWEMIHSE
Mycobacterium_smegmatis2          NFMPMSHVMGRGILCSTLASGGTAYFAARSDLSTFLEDLALVRPTQLNFVPRIWDMLFQE
Mycobacterium_vaccae              NFLPMSHVMGRGLLYATLGAGGTAYFAAKSDLSTFLEDLALVRPTQLSFVPRIWDIIFAE
Mycobacterium_obuense             NFMPMSHMMGRGVLYGTLGAGGTAYFAARSDLSTFLDDLALVRPTQLSFVPRIWDTIAAE
Nocardia_seriolae                 CYMPLSHVAGRMVLSGTFARGGTAYFAASSDMSTLFEDIALVRPTEVFFVPRVCDMLFQR
Nocardia_otitidiscaviarum         NYMPLSHIAGRMSLYGTLMRGGTAYFAAASDMSTLLDDFGLARPTELFLVPRVCELLHQR
Nocardia_iowensis                 NYMPMSHIAGRISLFGVLARGGTAYFAAKSDMSTLFEDIGLVRPTEIFFVPRVCDMVFQR
Nocardia_transvalensis            SYMPMSHVAGRGSLISALSLGGTVYFAARSDMSTLFDDITLCRPTMVFFVPRVCDMVFQR
```

```
Firefly_Luciferase|BAL46512.1     ----------------AKSTLIDKYDLSNLHEIASGG---------APLSKEV-GEAVAK
FadD13|NP_217605.1                ------------VPEFAELDAP------DFRYFITGG---------APMPEALIKIYAAK
NRPS|WP_014382786.1               ------------ALQSADALAPEVGEQLKLQTVVFGGEALE--------PHRL-STWLHH
Tsukamurella_paurometabola        YLA---------VEADTDAET----ARVELRDRVLGGRLQAAMCGSAALSSEL-QTFMEW
Mycobacterium_smegmatis1          HLATVDRL----VTQGADELTAEKQAGAELREQVLGGRVITGFVSTAPLAAEM-RAFLDI
Mycobacterium_genavense           YRGAVDRG----IAEGADPATAEHDAATEMREQVLGGRVLGGFVGSAPLATEM-KAFLDS
Mycobacterium_marinum             FGREVDRRLRDGTAEGADPGALKARVAADLRQVLLGGRYALAMMGSAPISEQM-KASVES
Nocardia_asteroides               FRREVDRR----ADAGVDPEVLAEEVRGELRERVLGGRLLTVLCGSAPIAPEL-RRFVES
Nocardia_paucivorans              FQSEMYRR----AADGREQAEVAAEVTAELRRNLLGGRFLSATTGSAPMSAEM-KVWVED
Mycobacterium_fortuitum           YQSRVDQR----LAEGRDREAVEAEVLAEVRDKVLGGRFVAAMTGSAPISAEL-KTWTQD
Mycobacterium_smegmatis2          YQSRLDNR----RAEGSE-DRAEAAVLEEVRTQLLGGRFVSALTGSAPISAEM-KSWVED
Mycobacterium_vaccae              VAKELERR----TADAAD-------VLADLRQSLLGGRYVSAMTGSAPLSAEM-ESFVEQ
Mycobacterium_obuense             VAKEVDRR----PDDLAD-------VYADLRQSLLGGRHVMAMSGSAPLSPEM-RTFVED
Nocardia_seriolae                 CQSEVQRR----TAAGESVEDADAAVKTALREEFLGGRLVRVMVGSAPVSAEM-KEFMRS
Nocardia_otitidiscaviarum         YQSELDRR----VVAGEDAETAATNVKAELRERVLGGRYLTALSGSAPLAAEM-KTFMES
Nocardia_iowensis                 YQSELDRR----SVAGADLDTLDREVKADLRQNYLGGRFLVAVVGSAPLAAEM-KTFMES
Nocardia_transvalensis            YRSEVDRR----LAAGGDREQVEREVKTELREHFLGGRFLLALVGSAPLSPEM-RAFMES
```

active site and mg^{2+} binding a(G/W)x(A/T)E (A5)

```
Firefly_Luciferase|BAL46512.1     RFHLPGIRQGYGLTETT--SAILITPEGDDKPG-AVGKVVPFFEAKVVDLDTG---KTLG
FadD13|NP_217605.1                NIE---VVQGYALTESCGGGTLLLSEDALRKAG-SAGRATMFT--DVAVRGDDGVIREHG
NRPS|WP_014382786.1               HPGLPRMINMYGITET---TVHASFREILRGDVDNNVSPIGVPLGNLAFFVLDGWLRPVP
Tsukamurella_paurometabola        LLGID-IQIGYGSTEA---GGVIRDGVVVRPPV-TEYKLIDVP--ELGYFVTD---SPHP
Mycobacterium_smegmatis1          TLGAH-IVDGYGLTET---GAVTRDGVIVRPPV-IDYKLIDVP--ELGYFSTD---KPYP
Mycobacterium_genavense           MLDAH-ITDGYGLTET---GMLTRDNVVNRNRV-IDYKLIDVP--ELGYFLTD---RPYP
Mycobacterium_marinum             LLDLD-VMEGYGSTEA---GTVIINNEVQRPQV-IDYKLVDVA--ELGYFLTD---RPYP
Nocardia_asteroides               VLRLR-LHDGYGSTET---GGVIFDTKVMRPPV-LDYKLVDVP--ELGYFSTD---KPYP
Nocardia_paucivorans              LLDLP-LRDGYGSTEA---GSITLDGRVRRPPV-IDYKLVDVP--ELGYFRTD---RPYP
Mycobacterium_fortuitum           MLGIH-LLEGYGSTEA---GMALFDGVVQRPPV-IDYKLVDVP--DLGYFGTD---QPHP
```

```
Mycobacterium_smegmatis2            LLDMH-LLEGYGSTEA---GAVFIDGQIQRPPV-IDYKLVDVP--DLGYFATD---RPYP
Mycobacterium_vaccae                LLDMH-LIDGYGSTEA---GAVLVDGQIQRPPV-IDYKLVDVP--DLGYFSTD---RPHP
Mycobacterium_obuense               LIDIH-LTDGYGSTEA---GAVFVDGQVQRPPV-IDYKLVDVP--DLGYFTTD---RPHP
Nocardia_seriolae                   VMGQP-VIDGYGSTEA---GGILIDNEIRRPPV-IDYKLADVP--ELGYFSTD---KPHP
Nocardia_otitidiscaviarum           LLDDE-LHDGYGSTEA---GSVLLDNRIKRPPV-LDYRLVDVP--ELGYFRTD---KPHP
Nocardia_iowensis                   VLDLP-LHDGYGSTEA---GSVLLDNQIQRPPV-LDYKLVDVP--ELGYFRTD---RPHP
Nocardia_transvalensis              VLEIG-MFDGYGATET---GGVLLNNELQRPPV-LDYRLEDVP--ELGYFGTD---KPYP
```

Distorted beta sheet GEx_{10-14}GY (A6) ATP binding (S/T)GD (A7)

```
Firefly_Luciferase|BAL46512.1       VNQRGELCVRGPMIMSGYVNNPEATNALIDKDGW-------LHSGDIAYWDEDEHFFIVD
FadD13|NP_217605.1                  ---EGEVVIKSDILLKEYWNRPEATRDAFD-NGW-------FRTGDI-GEIDDEGYLYIK
NRPS|WP_014382786.1                 VGVVGELYVAGGGLATGYVGRPGLSATRFVACPFGAPGARMYRTGDLVRWSADGQLEYIG
Tsukamurella_paurometabola          ---RGELLVKSTQLIPGYYNSDK---RIRDDEGF-------YRTGDVMAELGPDRLEYVD
Mycobacterium_smegmatis1            ---RGELLVRSQTLTPGYYKRPEVTASVFDRDGY-------YHTGDVMAETAPDHLVYVD
Mycobacterium_genavense             ---RGELLVKTDTMTPGYYKRPEVTAQVFDEDGF-------YKTGDVMAEIEPDHLVYVD
Mycobacterium_marinum               ---RGELLVKTRTLFSGYYRDPEDGAQVFDPDGF-------YRTGDIMAQVGPDRLAYLD
Nocardia_asteroides                 ---RGELLLKTTTMISGYYRRPEVTAQVFDEDGF-------CRTGDVVAELGPDRVAYVD
Nocardia_paucivorans                ---RGELAVRSESLTPGYYRRPDVTAQVFDADGY-------YHTGDIFAEVAPDELVYVD
Mycobacterium_fortuitum             ---RGELLIKTENLFPGYYKRPEVTASVFDEDGF-------YRTGDVVAEIGPDQLRYVD
Mycobacterium_smegmatis2            ---RGELLVKSEQMFPGYYKRPEITAEMFDEDGY-------YRTGDIVAELGPDHLEYLD
Mycobacterium_vaccae                ---RGELLVRSETLFPGYYKRPDITAEMFDEDGY-------YRTGDIVAETAPDRLTYLD
Mycobacterium_obuense               ---RGELLVKSETLFPGYYKRPEVTAEMFDPDGY-------YRTGDVVAETGPDQLVYLD
Nocardia_seriolae                   ---RGELLVKSTQQIPGYFKRPDVTAEIFDADGF-------YRTGDIVAEVRPDHLVFVD
Nocardia_otitidiscaviarum           ---RGELLLKTESMFPGYYKRPEITAEMFDADGF-------YRTGDVVAELGPEQLVYVD
Nocardia_iowensis                   ---RGELLLKAETTIPGYYKRPEVTAEIFDEDGF-------YKTGDIVAELEHDRLVYVD
Nocardia_transvalensis              ---RGELLLKSETLVPGYYKRPELNAEIFDADGF-------YRTGDVMAEIGRDRLVYVD
```

Hinge domain Rx(D/K)x_6G modified to Rx_4Kx_3G in CARs (A8)

```
Firefly_Luciferase|BAL46512.1       RLKSLIKY-KGYQVAPAELESILLQHPNIFDAGVAGLPDDDAGELPAAVVVLEHG-----
FadD13|NP_217605.1                  DRLKDMIISGGENVYPAEIESVIIGVPGVSEVAVIGLPDEKWGEIAAAIVVAD-------
NRPS|WP_014382786.1                 RADEQVKI-RGYRIELGEIRAALADVEGVEQAAVLVREDRAGEKRLVGYV----------
Tsukamurella_paurometabola          RRSNVIKLAQGEFVPIAQLEAIYAAGPDVHQIFLYGTSERSY--LIGVVVPAPGPDGET-
Mycobacterium_smegmatis1            RRNNVLKLAQGEFVAVANLEAVFSGAALVRQIFVYGNSERSF--LLAVVVPTPEALEQD-
Mycobacterium_genavense             RRNNVLKLAQGEFVAVANLESIYAGAPLVRQIFVYGNSERSN--LLAVIVPTPEALAEGN
Mycobacterium_marinum               RRNNVLKLSQGEFVAVSRLEAIFANSPLVRQIFVYANGARAY--PLAVVVPTQDAQSRHG
Nocardia_asteroides                 RRNNVLKLSQGEFVTVSRLEAVFAGADLVRQIYVYGSSERAY--LLAVIVPTEAALAGP-
Nocardia_paucivorans                RRSFVLKLSQGEFVTVSKLEAVFARSPLVRQIYVYGNSTRSY--LLAVVVPTEEAQARGD
Mycobacterium_fortuitum             RRNNVLKLAQGEFVTLAKLEAVFGNSPLVQQIYVYGNSAQPY--LLAVVVPTDPSVS---
Mycobacterium_smegmatis2            RRNNVLKLSQGEFVTVSKLEAVFGDSPLVRQIYVYGNSARSY--LLAVVVPTEEALSRWD
Mycobacterium_vaccae                RRNNVLKLSQGEFVTVSKLEAVFGDSPLIHQIYVYGNSARPY--LLAVVVPTDAALAR--
Mycobacterium_obuense               RRNNVQKLSQGEFVTVSKLEAVFGDSPRVRQIYVYGNSARSY--LLAVVVPTEDVLGR--
Nocardia_seriolae                   RRNNVLKLSQGEFVAVSKLEAVYATSPLIAQIFVHGSGERSH--LLAVIVPTAAARALA-
Nocardia_otitidiscaviarum           RRNNVLKLSQGEFVTVAALEAVYATSPLIRQIFVYGSSERAY--LLAVVVPTDAVLALP-
Nocardia_iowensis                   RRNNVLKLSQGEFVTVAHLEAVFASSPLIRQIFIYGSSERSY--LLAVIVPTDDALRGRD
Nocardia_transvalensis              RRNNVLKLSQGEFVAVSKLEALYTSSPLVRQIYVYGSSERAY--LLAVIVPTDDAVTAPE
```

Helix associated with adenylation (LPxYM(IV)P (A9)

adenylate forming motif Px_4GKrx(R/K) poorly conserved apart from second active site lysine (A10)

```
Firefly_Luciferase|BAL46512.1       ----KTMTEKEIVDYVASQVTTAKKLRGGVVFVDEVPKGLTGKL---DARKIREILIKAK
FadD13|NP_217605.1                  ----QNEVSEQQIVEYCGTRLARYKLPKKVIFAEAIPRNPTGK----ILK---TVLREQY
NRPS|WP_014382786.1                 ----TGAADPSEIRARLGRRLPTYMVPAAVVVLDVLPLTVNGKL---DTRALPAPE----
Tsukamurella_paurometabola          DAQTRTRVLDGLAAIARENDLAAYEVPRDVLI-ERDPFSQENGLRSGIGKLVRPALIARY
Mycobacterium_smegmatis1            PAALKAALADSLQRTARDAELQSYEVPADFIV-ETEPFSAANGLLSGVGKLLRPNLKDRY
Mycobacterium_genavense             SPALKTAIHQSLRQTAAAAQLQSYELPVDFIL-ETKPFTDENGLLSGLGKQLRPRLKERY
Mycobacterium_marinum               RAELKAELHTSLHRVAMSAGLAPYEIPRDFIV-ETTPFTPQNGLLTAIHKLARPHLTQRY
Nocardia_asteroides                 AASLRAELGASLQRAAVAAELEPYEIPRDFLI-ESVPFSVDNGLLSGVSKLLRPALKQRY
Nocardia_paucivorans                VEALKPLIGRSLRQVAKTAGLQSYEIPRDLIV-DTTPFTLRNGLLTGSRKLARPKLEEHY
Mycobacterium_fortuitum             ----KEAIAESLQEVAREADLQSYEIPRDFIV-ETTPFSLENGLLTGIRKLAWPKLKAHY
Mycobacterium_smegmatis2            GDELKSRISDSLQDAARAAGLQSYEIPRDFLV-ETTPFTLENGLLTGIRKLARPKLKAHY
Mycobacterium_vaccae                -EDVKTAVAESLQDVARAADLQSYEIPRDFLI-ETTPFTLENGLLTGIRKLARPALKERY
Mycobacterium_obuense               -DDAKALVAESLQNVARAAGLQSYEIPRDFLI-EPTPFTLENGLLTGIRKLARPRLKEHY
Nocardia_seriolae                   PAERTAAIAESLRQIARDAELESYEIPRDFIV-EDEPFTQENGLLSGIAKLLRPKLRERY
Nocardia_otitidiscaviarum           AARARAEVSESLQRIAKESGLRPYEIPRDLII-ESEPFTIDNGLLSGIGKLLRPKLKEHY
Nocardia_iowensis                   TATLKSALAESIQRIAKDANLQPYEIPRDFLI-ETEPFTIANGLLSGIAKLLRPNLKERY
Nocardia_transvalensis              PAELKAAIGESLQRVARDAELESYEIPRDFLV-EPEAFSMANGLLSGIGKLLRPKLKQRY
```

```
Firefly_Luciferase|BAL46512.1       KGGKSKL----------------------------------------------------
FadD13|NP_217605.1                  SATVPK-----------------------------------------------------
NRPS|WP_014382786.1                 -------YSDVD----------RYRAPASAIEEILAGIYAQVLGAERVGV--DDSFFD
Tsukamurella_paurometabola          GDRLHDLYAQADTRQREGLRALDASG---PIIDTVLGAAALTLGADIADFDADTRFGD
Mycobacterium_smegmatis1            GQRLEQMYADIAATQANQLRELRRAAATQPVIDTLTQAAATILGT-GSEVASDAHFTD
Mycobacterium_genavense             GEQLERLYSEIAAAQVDEIRVLREAAADRPAVETLAGACRALLGTSGADS--ESHFTD
Mycobacterium_marinum               GARLELLYTELADSQTRRLHRLRQTGGRLPALETIRRAAGALLGTETTEPRPEAHFKD
Nocardia_asteroides                 GARLDALYDEVVREQEAELERLRREAPMLPVDEVVARAALAVLGCAAADLRPSARFGE
Nocardia_paucivorans                GPRLEQLYTELAEAGTDELRALRRDGADRPVLETVRRAVSALLGTTDGAPPPEAHFTD
```

```
Mycobacterium_fortuitum              GERLEQLYAELAETQAAELRELRSASADAPVVETVSRAAGALLGAAASDLGPDAHFTD
Mycobacterium_smegmatis2             GERLEQLYTDLAEGQANELRELRRNGADRPVVETVSRAAVALLGASVTDLRSDAHFTD
Mycobacterium_vaccae                 GDRLEALYAELAEGQTDELRELRRSGAERPVQETVLRAAAALLGAAATDLQPDAHFTD
Mycobacterium_obuense                GEQLEALYAELADGQADEMRTLRADGANRPMLETVGRAAAALLGTAATDVQPDAHFTD
Nocardia_seriolae                    GARLEQMYDEQAQRQRDELATLRREAAELPVLETVCRAARAVLG--GTQPPPDAHFTD
Nocardia_otitidiscaviarum            GERLEQLYAELAEQREDELTALRRGAHDRPILDTVTRAAGAVLDLTAGEVSPDAHFAD
Nocardia_iowensis                    GAQLEQMYTDLATGQADELLALRREAADLPVLETVSRAAKAMLGVASADMRPDAHFTD
Nocardia_transvalensis               GDRLEERYDELSREQQDELTALRTAAADLPVLETVSRAAKALLGCATTDLRPDAHFAD
```

Supplementary Figure 1 - MUSCLE Alignment between *Mycobacteria, Tsukamurella* and *Nocardia* CARs, Firefly Luciferase from *Photinus pyralis*, a long chain fatty acid CoA ligase (FadD13) from *Mycobacterium tuberculosis*, and an adenylating subunit from a non-ribosomal peptide synthase from *Mycobacterium intracellulare* suggests the CARs are a member of the ANL enzyme superfamily. Confidently conserved domains that confer the hallmark functions of the ANL family are highlighted blue. Potentially, or poorly conserved domains are highlighted yellow. ANL hallmark motifs were identified according to Gulick 2009 and Marahiel *et al.,* 1997. Accession numbers for the CARs are found in Supplementary Figure 2.

```
>Mycobacterium_marinum|ACC41375.1
MSITCVDTRAQRSARRIEQLYSTDAQFAAARPSTAVGIAISKSGLGLPQIIQTVMDGYPQRPALGQRATRVVTDPNTGRSSAQLLAEFETITYRELWNRTNALTNAFAAEALADRGQRV
CVLGFASIDYATIDLALMLLGAVSVPLPTNAARAQLCHIVSETQPSLIASSTENLPDAISLVLSHRAPHRVVVFDYRPELDAHREALEAARARLAAIPVTVETLTAIIARGRTVRPAEA
DCGAQSADAPALLIYTSGSTGAPKGVVYTRNRVADFWRTSKAEVEATEQRTAPSITLNFMPMSHANGRQVLYGTLSNGGTAYFTARSDLSTLFDDLALVRPTELGFPPRIWDMLLERFG
REVDRRLRDGTAEGADPGALKARVAADLRQVLLGGRYALAMMGSAPISEQMKASVESLLDLDVMEGYGSTEAGTVIINNEVQRPQVIDYKLVDVAELGYFLTDRPYPRGELLVKTRTLF
SGYYRDPEDGAQVFDPDGFYRTGDIMAQVGPDRLAYLDRRNNVLKLSQGEFVAVSRLEAIFANSPLVRQIFVYANGARAYPLAVVVPTQDAQSRHGRAELKAELHTSLHRVAMSAGLAP
YEIPRDFIVETTPFTPQNGLLTAIHKLARPHLTQRYGARLELLYTELADSQTRRLHRLRQTGGRLPALETIRRAAGALLGTETTEPRPEAHFKDLGGDSVSAVTFSNLLHDIYGFDVPV
GVILGPATDLRALASHVESRRGAGWSGPSFASVHVPRATSVHAGDLKLAKFLDTKTLAAATSLPAADARARTVLLTGATGFLGRYLVLEWLRRLRAVGGKLICLVRAASDEQARVRLDT
AFDSGDPQLPEHFRQLAVDRLEVLAGDKSEPGLGLDGPTWQRLADTVDLIVDPATLVNHVLSYRQLFAPNVAGTAELLRLALTTKRKPYAYVSTVSVANQIEPSAFTEDADIREISRTR
TIDDSFANGYTTSKWASEVLLREAHDLCGLPVTVFRCDMILADTSYAGQLNLADTFTRLMLSVAATGIAPASFYRLGPDGKRQPAHFDGLPVEFIAEAVATLGARRHDGFQVHHVANPH
HDGVGLDEYVDWLVDAGCPIRRIPDYDEWLSRFETALHALPDRKRRHSLLPLLQNYREPAEPIRGGIAPAPRFRGAVRQAKIGRDNDIPHVGPAIIAKYASDLQLLGLA

>Nocardia_iowensis|Q6RKB1.1
MAVDSPDERLQRRIAQLFAEDEQVKAARPLEAVSAAVSAPGMRLAQIAATVMAGYADRPAAGQRAFELNTDDATGRTSLRLLPRFETITYRELWQRVGEVAAAWHHDPLRAGDFVALLG
FTSIDYATLDLADIHLGAVTVPLQASAAVSQLIAILTETSPRLLASTPEHLDAAVECLLAGTTPERLVVFDYHPEDDDQRAAFESARRRLADAGSLVIVETLDAVRARGRDLPAAPLFV
DTDDDPLALLIYTSGSTGTPKGAMYTNRLAATMWQGNSMLQGNSQRVGINLNYMPMSHIAGRISLFGVLARGGTAYFAAKSDMSTLFEDIGLVRPTEIFFVPRVCDMVFQRYQSELDRR
SVAGADLDTLDREVKADLRQNYLGGRFLVAVVGSAPLAAEMKTFMESVLDLPLHDGYGSTEAGSVLLDNQIQRPPVLDYKLVDVPELGYFRTDRPHPRGELLLKAETTIPGYYKRPEVT
AEIFDEDGFYKTGDIVAELEHDRLVYVDRRNNVLKLSQGEFVTVAHLEAVFASSPLIRQIFIYGSSERSYLLAVIVPTDDALRGRDTATLKSALAESIQRIAKDANLQPYEIPRDFLIE
TEPFTIANGLLSGIAKLLRPNLKERYGAQLEQMYTDLATGQADELLALRREAADLPVLETVSRAAKAMLGVASADMRPDAHFTDLGGDSLSALSFSNLLHEIFGVEVPVGVVVSPANEL
RDLANYIEAERNSGAKRPTFTSVHGGGSEIRAADLTLDKFIDARTLAAADSIPHAPVPAQTVLLTGANGYLGRFLCLEWLERLDKTGGTLICVVRGSDAAAARKRLDSAFDSGDPGLLE
HYQQLAARTLEVLAGDIGDPNLGLDDATWQRLAETVDLIVHPAALVNHVLPYTQLFGPNVVGTAEIVRLAITARRKPVTYLSTVGVADQVDPAEYQEDSDVREMSAVRVVRESYANGYG
NSKWAGEVLLREAHDLCGLPVAVFRSDMILAHSRYAGQLNVQDVFTRLILSLVATGIAPYSFYRTDADGNRQRAHYDGLPADFTAAAITALGIQATEGFRTYDVLNPYDDGISLDEFVD
WLVESGHPIQRITDYSDWFHRFETAIRALPEKQRQASVLPLLDAYRNPCPAVRGAILPAKEFQAAVQTAKIGPEQDIPHLSAPLIDKYVSDLELLQLL

>Mycobacterium_phlei|WP_003889896.1
MASESRDVRLQRRIAELYDTDPQFAAARPDEAVARAVNAPGLTLSQVIRTVLDNYADRPALGYRAVEFAADPASARTVARLLPRFDTITYRELGERIDATTAALGHDGLRPGERVAILG
FSSVDYTTIDIAAFNLGAVSVPLQTSAPPSQLRPMTAETEPAVIAASVDFLDDALELIRTGHAPRRLVVFDFHPEIDDHRDALAAATTGLADTAMTVETLDSLLTRGRTLPAPTGYRDR
DDDLALLIYTSGSTGAPKGAMYQRRMVTNMWRRATTAIGGKEAAPWLTLNFMPMSHVMGRGILSTTLCSGGTAYFAARSDLSTLLEDLALVRPTQLTFVPRIWEMIFQEYQRDVARRPE
AEVLADLREHRLGGRFLAAMTGSAPMSPEMTAFAESVLDLHLVDGYGSTEAGSIVLDGQVLRPPVLDYKLVDVPELGYFSTDRPYPRGELLVKTELMFPGYYKRPDITAEVFDEDGYYR
TGDIVAELGPDRLAYVDRRNNVLKLSQGEFVTVSKLEAAFAASPLVRQIYIYGNSAHPYLLAVVVPTEDALTRYDAATLKTAISESLQDVGRAAGLQSYEIPRDFLVETTPFTLENGLL
TGLRKLARPKLKEHYGERLEQLYTELADGQAEELKELRTHGAQQPTLTTVSRAATALLGTASAEVRPDAHFTDLGGDSLSALTFGNLLGEIYAVEVPVGVIVSPANDLAAIADYIDTAR
RPGDGRPTFAGVHGDDAAEVHARDLTLDRFLDDATLAAATALPGPAPEIRTVLLTGATGFLGRYLALEWLERMAMVGGTLICLVRGRDDAAARARLDQIFDSGDPELLRHYRELADRHL
EVIAGDKSDADLGLDRRTWQRLADTVDLIVDPAALVNHVLPYRELFGPNVVGTAELIRLALTGRLKPYLYTSTIAVGAGIAPGQFTEDADIRQISATRTLDDSYANGYATSKWAGEVLL
REAHDLCGLPVAVFRCDMILADTSYAGQLNVPDMFTRLILSLVATGIAPLSFYELDAAGHRQRAHYDGLPVEFVAEAVSALGLDVAGGFATYHVMNPYDDGIGLDEFVDWLTGAGYPIE
HVNDYGTWFQRFETAIRGLPERQRQASLLPLLHSYQRPQPPIRGSAAPTDRFRSAVQDAKIGPDKDIPHITPEVIVKYVTDLRLLGLL

>Tsukamurella_paurometabola|WP_013126039.1
MSIETVQNGVPAEGSVPPADQQTERLPQVIARIFAQFADRPAFATREAGPGTPYATVSYREIWRRVTALVASWQSEVAPGDFVAILGFTSSDFVTVDLATTLLGAPNVPLQAGAPAARI
ATILDETRPKILAVSADQVDLAQEALAESAATPRVVVFDGERDGYEGIEADILSGSALPAPEFFAEPGTDPLVTLIYTSGSTGTPKGAMYTEQLVRDAWLKVDSIVDIDMPAESLLHFL
PMSHMYGRNWLIAGLASGGTGYFAGASDMSTLFDDLAAARPTAIGLVPRVCELIHQRYLAVEADTDAETARVELRDRVLGGRLQAAMCGSAALSSELQTFMEWLLGIDIQIGYGSTEAG
GVIRDGVVVRPPVTEYKLIDVPELGYFVTDSPHPRGELLVKSTQLIPGYYNSDKRIRDDEGFYRTGDVMAELGPDRLEYVDRRSNVIKLAQGEFVPIAQLEAIYAAGPDVHQIFLYGTS
ERSYLIGVVVPAPGPDGETDAQTRTRVLDGLAAIARENDLAAYEVPRDVLIERDPFSQENGLRSGIGKLVRPALIARYGDRLHDLYAQADTRQREGLRALDASGPIIDTVLGAAALTLG
ADIADFDADTRFGDLGGDSLSALSLATTLEGLYDVPVPVQTIVGPTATLGGVARHIEKARSGGVAAPTADSVHGVGASVARATDLTLEKFIDPELLALAPTLPAATGEPNTVLLTGSTG
YLGRFLLLDWLRRVAPHGGTVIALVRGADADDARRRVTAAIGDSDPDLTQEFTSLAEHHLHVIAGDFGSPALGLDDATWSDLAGRVDHVVHCGALVNHVLPYDQLFGPNVVATGEVVRL
ALTTRRKSVDYVSTVAVVPQDDGRVLVEDDDVRELGAERRIGDAYANGYAVSKWAGEVLLHEAADLADLPVRVFRSDMILAHSRFHGQFNEVDQFTRLLLSIAETGLAPASFYTPDPSG
HRPHYDGLPVDFTAEAITTLSAAGRSGYRTFHVLNANDDGVSLDSFVDWIAASGRSIERIDDYDTWFARFEQALQQLPDEARQRSVLPLLHAVREPAPAAGTSALSVDRFRGAVRETGV
GPGDIPVLDRALIEKYLRDFETAGWL

>Streptomyces_rimosus|WP_030643740.1
MYATDPQFRDAAPLDSVTEAIRRPGLPLADLVATVMEGYADRPALGERATEPVTDPDTGRTTLRLLERFDTITYGELWERVGAVASEWRHHPGHAVDRGDFVALLGPTSAEYAVVDLAC
VRSGAVSVPLQAGASAEHLAPIVAQTGPRLLAVDMAHLDVALQLAAEAPSLGRIVVLGHRPDITTHREGLDSARDRLAAQGRGVTVDTLASVIERGRDLPPLPRCPEGTTPDALSSLIY
TSGSTGTPKGAMYTERLVRQFWVDFVPGQGVRPSIVLNYMPLSHMMGRGVLFGTLAKGGIAYFVASSDLSTLFEDLSLTRPTEFIMVPRISDMLFQRYQAELARRSDTGAEAPGATADQ
AEHVQEDVQAELQAELRKELREKTLGGRLLWAVSASAPLSAEMTAFVENCLHVKLFNGYGSTEAGIVSLDGRVVRPPVTDHKLADVPELGYFRTDSPHPRGELLIKSDRLFSGYFQRPD
ATAQVFDEDGFYRTGDIMARTGPDTLVYVDRRSNVLKLSQGEFVATSRLEALFIGSPFVRQVFVYGNSTRAYLLAVIVPTQDALDRAGEDTQRLRSILRESLQRLATEAGLNAYEIPRD
FLIETEPFSQQNGLLSGVRKLLRPALTKRYGERLEALYTELAERGTDELQALRQAGPSQPIPETVLRAARALLGHRQGDVKPDTHFLELGGDSLSALSFSQLLKEIFHVDVPVDVLINP
VNTLRQVADHIENALAGHRRPTADSVHGPGAKRLLASDLKLDAFLDTGTLAKTGRAAGPLPEARTVLLTGANGYLGRFLCLEWLERVAERGGTLVCVVRGSTDEAARARLDAAFDSGDA
ELLRHYRELAAEHLEVVAGDIGETDLGLGKETWQRLADTVDLIVHPAALVNHVLPYDQQFGPNVLGTAELIRLALTYRVKQFTYLSTVAVVFGAEAAADETADIRTACAVRDLGGGYAD
GYAAGKWAGEVLLREAHETYGLPVAVFRSNMILAHRRYRGQLNIPDVFTRLLLSLLATGIAPGSFYAGGAGADTGSGHYDGLPVDFTARAVAALGDGTREGYRTFNVVNPHEDGISLDT
FVDWLTAAGHPLTRIHDYDAWLDRFETAMRGLPDRQRQHSLLPLLHAFTKPEEPLPGSALPAQRFRAAVRAAALDGEADIPHLSQDLITKYVADLRAQHLL
```

Supporting information

>Streptomyces_celluloflavus|WP_052859397.1
MYATDPQFRGAAPLDAVTEAIRRPGLPLADLVDTVMEAYADRPALGERATEPVTDPVTGRTTLRLLARFDTITYGELWERVGALAAEWHHHPDHAVGPGDFVAVLGHPSAEYTVVDLAC
VRSAAVSVPLQAGASAQQLAPIVAQTGPRLLAVDMAHLEVALRIAADAPSPVRIVVFGHRPEVTAHREQLDSARDRLAAQGRGVTLDTLAAGIERGRSLPPAPRSREGSTADALSALIY
TSGSTGTPKGAMYTERLVTRFWVDFVPGQEIRPSIVLNYLPLSHVMGRGALYGTLAKGGTAYFVASSDLSTLFEDLSLVRPTEFLMVPRICDMLFQRYQSELIWWNAAGGDAAEQADRV
KEELREKTLGGRLLWAVSASAPLSAEMSAFVESCFQVRMLDGYGSTEAGVVSLDGRLLRPPVTDHKLADVPELGYFRTDSPHPRGELLIKTDRLVPGYFRRPDATAQVFDEDGFYHTGD
IMARTGPDELVYVDRRSHVLKLSQGEFVAVSRLEALFTGSPVVRQIFVYGNSARAYLLAVIVPTQDALDRVGGDTRLLGPALRESLQLLATEAGLNSYEIPRDFLIETEPFSQRNGLLS
GVRKLLRTALTKHYGERLEALYTELTERETDELRALRQAGPSRPVGETVCRAARALLGHRQGDLMPGTRFLELGGDSLSALSFSQLLKEIYDVDVPVDVVINPVNTLQQVADHIEKALA
SGHRRPTAESVHGPGATRLVAGDLTLNAFFDTEPLAQADRPADPLPQARTVLLTGANGYLGRFLCLEWLERVAERGGTLVCLVRGGTDELARTRLDAAFDSGDPGLLRRYHDLAAEHLE
VVAGDVGETGLGLGEETWQRLADTVDLIVHPAALVNHVLPYDQLFGPNVLGTAELIRLAVTSRVKQFTFLSTVAVVFGYEAAADETADIRTACATRDLDSGYADGYAAGKWAGEVLLRE
AHDRFGLPVAVFRSNLILAHPRYRGQLNIPDVFTRLVLSLLATGIAPGSFYARGTGEGGGHYDGLPVDFTARAVASLGDDAREGYRTFNVVNPHEDGISLDTFVDWLVAAGHPLARIHD
YDEWLHRFETALRGLPDGRRQHSLLPLLHAFARPQEPLPGSALPADRFRAAVRAAAFNEENDIPCLSRDLITKYVTDLRAEGLL

>Nocardia_paucivorans|WP_040789375.1
MSVDTRESRLERRIAELYATDEQFAAARPDVAVTAEVERAGRRSARVVHAVAKGYAHRPALGQRAVDYITDPRTGRTSMELLPRYETLTYREVWERAGAIASALAGNPVRAGDRVCILG
FASVDYTTIDMALTRLAAVSVPLQTGAPAERLRPIIAETEPTVLAASIDHLDDAVTAVLAGHLPARLIVFDYHPRVDDQREAFAAATEKLAEAGGPVVIETLAEVIDRGTALPPVPLAP
AEEPDSLALLIYTSGSTGAPKGAMITDRMVAEHWRAATAERWGQRGTEPSIVLGFMPMSHILGRAICWMALGSGGTVYFAAKSDLSTLLDDLALVRPTQLTFVPRVWEMLFQRFQSEMY
RRAADGREQAEVAAEVTAELRRNLLGGRFLSATTGSAPMSAEMKVWVEDLLDLPLRDGYGSTEAGSITLDGRVRRPPVIDYKLVDVPELGYFRTDRPYPRGELAVRSESLTPGYYRRPD
VTAQVFDADGYYHTGDIFAEVAPDELVYVDRRSFVLKLSQGEFVTVSKLEAVFARSPLVRQIYVYGNSTRSYLLAVVVPTEEAQARGDVEALKPLIGRSLRQVAKTAGLQSYEIPRDLI
VDTTPFTLRNGLLTGSRKLARPKLEEHYGPRLEQLYTELAEAGTDELRALRRDGADRPVLETVRRAVSALLGTTDGAPPPEAHFTDLGGDSLSALTFANLLRDIFDVDVPVGVIVGPAT
DLRALAEYIDRQRGGTERPTFAAVHGAGATELRARDLTLDKFLDPATLAAAPSLPGPSSTARTVLLTGATGFLGRYLALEWLRRMSFVDGKLICLVRAEDDATARQRLDRTFSAGDPRL
FTHYREAAAGHLEVLAGDKSEPELGLDHTTWQRLADTVDLIVDPAALVNHVLPYSQLFGPNVVGTAELIRLALTTKQKPYTYVSTIGVSAQIDPSAFTEDADIRTIGPIRALDDSYANG
YSNSKWAGEVLLREAHDLCGLSVAVFRCDLILADTEYAGQLNVPDMFTRLMLSLLATGIAPGSFYELDSAGRRQRAHYDGLPVGFVSEAIAALGAQPAAEFRTYHVMNPYDDDISLDAC
VDWLIDAGHPIERIPDYETWLRRFETALRALPDRQRRHSVLPLLHSYRQPQRPVRGSIAPTERFHAAVREAKIGPDKDIPHVTREIIGKYATDLGLLGLL

>Nocardia_otitidiscaviarum|WP_029928026.1
MLDDARAERRERRIADALADDQVREAAADAAVSESVRRVEVRLARIVDAVMSGYGDRAALAWRRSELVDGAVRLLPEYSTMTYRELWRQAGAVAAEWGADPVRAEDFVCTLGFTSPDYT
VVDLALMRLAAVAVPLQASASVAQWRSIMAETEPRMLAASAETLPAAVEAVLGGFAPRRVLVFDYRPELEAHRSAVDSARERLAEVGCTVATVADAVDRGANLPAPLRIPSDRERLALL
IYTSGSTGAPKGAMYTDRLVAGLWLSANEIRVPALTMNYMPLSHIAGRMSLYGTLMRGGTAYFAAASDMSTLLDDFGLARPTELFLVPRVCELLHQRYQSELDRRVVAGEDAETAATNV
KAELRERVLGGRYLTALSGSAPLAAEMKTFMESLLDDELHDGYGSTEAGSVLLDNRIKRPPVLDYRLVDVPELGYFRTDKPHPRGELLLKTESMFPGYYKRPEITAEMFDADGFYRTGD
VVAELGPEQLVYVDRRNNVLKLSQGEFVTVAALEAVYATSPLIRQIFVYGSSERAYLLAVVVPTDAVLALPAARARAEVSESLQRIAKESGLRPYEIPRDLIIESEPFTIDNGLLSGIG
KLLRPKLKEHYGERLEQLYAELAEQREDELTALRRGAHDRPILDTVTRAAGAVLDLTAGEVSPDAHFADLGGDSLSALSFSTLLRDIFGVEVPVGFIVGPATDLARIAEYLVSERDSGS
RPTAATVHGDDGLLRADDLALEAFLDPATLDAAAHLPSALEPPRTVLLTGANGYLGRFLALEWLQRLDVSGGTLICLIRGSDADSARRRLDAVFATGDPELEAHYRELAERRLRVLPGD
IGEPNLGLREQDWRDLAETVDLIVHPAALVNHVLPYAQLFGPNVVGTAEVIRLALTSRLKPVTYLSTVAVSAGIDPETFTEDGDIREISPVRRLDDGYANGYGNSKWAGEVLLRNAHDR
FGLPVAVFRSDMILAHSRYAGQLNVPDMFTRLLLSVLATGLAPGSFHDAHGERHRAHYDGLPADFTAAAVTTLGSRVTSGYETYDVLNPHDDGISLDTFVDWLIEAGHPIDRIDDYAEW
FARFDTALRALPEHQRQHSLLPLLHAYRRPTPPLHGVALPAKHFRAAVQQAKLGPDGDIPHVTRELIEKYASDLRLLGLI

>Tsukamurella_pulmonis_hyp|KXP09115.1|now_T.spongiae
MQSSDPVVTGSATGEAGAPAERLPQVIARVFERFADRPAFATRDGGPRAPYVTVSYGEIWQRVTALAAAWQSELAPGDFVAILGFTSADFVTVDLATTLLGAPNVPLQAGAPAARIAAI
LDETRPKIFAVSADQAALAEQALAESSATPRVVVFDGEHAGYEGIEADVLAGRALPDPEFFAPEPDADPLVTLIYTSGSTGTPKGAMYTEKLVTDAWLKVDSIVDYDLPSESLLHFLPM
SHMYGRNWLIAGLASGGTGYFAGASDMSTLFDDLAAARPTAIGLVPRVCELVHQRFLTLEAETDTETARVELREHVLGGRLQAAMCGSAALSAELQTFMEWLLGIEIQIGYGSTEAGGV
LRDGEIVRPPVTEYKLIDVPELGYFVTDSPHPRGELLVKSTQLIPGYYKSDKRIRDDEGFYRTGDVMAELAPDRLEYVDRRSNVIKLAQGEFVPIAQLEATYAAGPDVHQIFLYGTSER
SYLLAVVVPAPGPDGETDAQARTRVLDGLAAIARDQDLAGYELPRDVIIEREPFSQENGLRSGIGKLVRPALNARYGDELAALYAAAEDRRRAGLRDLDADGSVTETVVRAAALTLGAL
PEELDAATRFADLGGDSLSALSLATTLEGLYEVPVPVQTIVGPTATLGGIVEHIEAARAGALSAPTAASIHGADAQVARASDLHLDRFVDPELLAAAPSIPAPHGEPSTVLVTGATGYL
GRFLLLEWLRRVAPHEGAVVALVRGADADDARRRVLDAIGTSDDALTAEFAELAERHLEVVVGDFGAPSLGLDAPTWDRLAERVDHVVHCGAMVNHVLPYDQLFGPNVVGTAEIARLAI
TVRRKSIDYVSTVAVVPQDDGRLLVEDDDVRVAGAERRIGADAYANGYAVSKWAGEVLLHEASELAGLPVRVFRSDMILAHSRFRGQYNPVDQFTRLLLSIAETGLAPASFAADPTGPR
PHYDGLPVDFTAEAIVTLGAAGREGFRTFHVLNVDSDGAGLDDFVDWIAEDRPIERIADYGEWFARFEAALQALPAEDRQRSVLPLLHSFAHPTPNGGGVALTADRFREAVREANVGPG
DIPGLDRALIERYLDGFTAAGWLA

>Tsukamurella_sunchonensis_hyp|KXP00736.1|now_T.pseudospumae
MLLMSVEIVESTEPNTAAGAASGAGGSPGHLADVITSAFTRFADRPAFATRDGGPDGPYRGTTYGQVWHRVTALTAAWREELQPGDFVAVLGFTSPDFVVVDLATTLLGAPNVPLQAGA
PAARIAAILDETAPKILAVSAAQLPLARAAVAESGTDPRLVVFDGAAPGATTLDDEISRGAALPVPEPYRAAEPDGDRLVTLIYTSGSTGTPKGAMYTEHLVVHAWLKVESIIDDDIPT
GVLLHFLPMSHMYGRNWLIAGLAAGGVGYFASAADMSSLFDDLAEARPTAIALVPRVCELVHQRFLALEAETDAESARTYLRDEVLGGNIAAAVCGSAALSADLQAFMEQLLGVSIQIG
YGSTEAGGVIVDGVVSRPPVIDYKLIDVPELGYLTTDLPHPRGELLVKSSQLIPGYYRSDKQVLDEDGYYRTGDVMAELGPDRLEYVDRRSNVIKLAQGEFVPIAQLEATYSANPAVHQ
IFLHGTSERSYLLAVVVPAPGPADETDEQARGRVLDALATVARENELAGYELPRDVIIEREPFSAENGLRSGIGKLVRPALTTRYGDRLADLYAAAEERRRDGLRGLDADGPVADTVVR
AAALTLGVLPEELDGGTRFVDLGGDSLSALSLATTLEGLYDLPVPVQTIVGPTATLAGVVAHIEAARAGAVQAPTAASIHGTGASIARAADLRLDRFIDADLLAAAPSKPGPHGEPKTV
LLTGATGYLGRFLLLEWLRRVALSDGTVVALVRGTDADDARRRVFDAIGTADAALTEEFARLAAGHLEVVPGDFGSPSLGLDDGAWERLTHTVDHVVHCGALVNHVLPYDQLFGPNVVA
TAEIVRLAVTDRRKSVDYVSTVAVVPQDDGRLLVEDDDVRVLGAERRIGAEAYANGYAVSKWAGEVLLREASDLAGLPVRVFRSDMILAHSRFRGQYNPVDQFTRLLLSIAETGLAPQS
FYEPDGSGRSPHYDGLPVDFTAESIVTLGSAAREGFRTFHVLNVNDDAVSLDSFVDWIAQERTVERIADYDEWLERFERALRALPDADRQRSVLPLLHSFARRSPAGAGGALTADRFRE
AVHAENVGPGDIPHLDRALVDRYLRGFEDAGWLSSEGSV

>Tsukamurella_tyrosinosolvens_hyp|KXP05362.1|now_T.carboxydivorans
MSIETVWGDDPAITGAAAAGTADGRAERLPQVIARIFARYADRPAFATRDGGPRAPYATVTYGEVWRRVTALAAAWRSELEPGDFVAILGFTSADFVTVDLATTLLGAPNVPLQAGAPA
ARIAAILDETRPKVFAVSADQVGLAEQALAESAATPRVVVFDGEHAGYEGIEADVLAGGVLPAPEFYAPEPGTDPLVTLIYTSGSTGTPKGAMYTEQLVSDAWLKVDSIVDIDLPSESL
LHFLPMSHMYGRNWLIAGLASGGTGYFAGASDMSTLFDDLAAARPTALGLVPRVCELIHQRFLAVEAETDTETARAELRDRVLGGRLQAAMCGSAALSAELQTFMEWLLGVEIQIGYGS
TEAGGVLRDGAIVRPPVTEYKLVDVPELGYFVTDSPHPRGELLVKSTQLIPGYYNSDKRIRDEEGFYHTGDVMAELAPDRLEYVDRRSNVIKLAQGEFVPIAQLEATYAAGPEVHQIFL
YGTSERSYLLGVVVPAPGPDGESDAAARVRVLDGLAAIARENGLASYEVPRDVIIEREPFSQENGLRSGIGKLVRPALTARYGAELEALYAAAEERRRDGLRALDADGSVTETVVRAAA
LTLGVLPEELDEGTRFLDLGGDSLSALSLATTLEGIYDLPVPVQAIVGPTATLRGVIAHIEEARAGGVQAPTSASIHGPDAEVARASDLRLDRFIDPALLAAAPSLPAPHGEPSTVLLT
GGTGYLGRFLLLEWMRRVAAHGGTVVALVRGADADDARRRVFGAIGTADPALTEEFTALAEGHLEVVVGDFGAPSLGLDTATWEALAERVDHVVHCGAMVNHVLPYDQLFGPNVVGTAE
IARLALTVRRKSIDYVSTVAVVPQDDGRLLVEDDDVRVAGAERRIGADAYANGYAVSKWAGEVLLREAADLADLPVRVFRSNMILAHSRFRGQYNPVDQFTRLLLSIAETGLAPASFSA
DPTGPRAHYDGLPVDFTAEAITRLGAAGRAGFRTFHVLNVAEGGAGLDDFVDWIAEDRPIERIADYAEWLARFEAALRALPAEDRHRSVLPLLHSFARPAETGAGAALTADRFREAVRE
ENVGPGDVPHLDRALIERYLDGFAATGWLA

>Mycobacterium_smegmatis1|AFP42026.1
MTSDVHDEQSTRRIAELYATDPEFAAAAPLPAVVDAAHKPGLRLAEILQTLFTGYGDRPALGYRARELATDEGGRTVTRLLPRFDTLTYAQVWSRVQAVAAALRHNPIYPGDAVATIGF
ASPDYLTLDLVCAYLGLVSVPLQHNAPVSRLAPILAEVEPRILTVSAEYLDLAVESVRDVNSVSQLVVFDHHPEVDDHRDALARAREQLAGKGIAVTTLDAIADEGAGLPAEPIYTADH
DQRLAMILYTSGSTGAPKGAMYTEAMVARLWTMSFITGDPTPVINVNFMPLNHLGGRIPISTAVQNGGTSYFVPESDMSTLFEDLALVRPTELGLVPRVADMLYQHHLATVDRLVTQGA
DELTAEKQAGAELREQVLGGRVITGFVSTAPLAAEMRAFLDITLGAHIVDGYGLTETGAVTRDGVIVRPPVIDYKLIDVPELGYFSTDKPYPRGELLVRSQTLTPGYYKRPEVTASVFD
RDGYYHTGDVMAETAPDHLVYVDRRNNVLKLAQGEFVAVANLEAVFSGAALVRQIFVYGNSERSFLLAVVVPTPEALEQDPAALKAALADSLQRTARDAELQSYEVPADFIVETEPFSA
ANGLLSGVGKLLRPNLKDRYGQRLEQMYADIAATQANQLRELRRAAATQPVIDTLTQAAATILGTGSEVASDAHFTDLGGDSLSALTLSNLLSDFFGFEVPVGTIVNPATNLAQLAQHI
EAQRTAGDRRPSFTTVHGADATEIRASELTLDKFIDAETLRAAPGLPKVTTEPRTVLLSGANGWLGRFLTLQWLERLAPVGGTLITIVRGRDDAAARARLTQAYDTDPELSRRFAELAD

RHLRVVAGDIGDPNLGLTPEIWHRLAAEVDLVVHPAALVNHVLPYRQLFGPNVVGTAEVIKLALTERIKPVTYLSTVSVAMGIPDFEEDGDIRTVSPVRPLDGGYANGYGNSKWAGEVL
LREAHDLCGLPVATFRSDMILAHPRYRGQVNVPDMFTRLLLSLLITGVAPRSFYIGDGERPRAHYPGLTVDFVAEAVTTLGAQQREGYVSYDVMNPHDDGISLDVFVDWLIRAGHPIDR
VDDYDDWVRRFETALTALPEKRRAQTVLPLLHAFRAPQAPLRGAPEPTEVFHAAVRTAKVGPGDIPHLDEALIDKYIRDLREFGLI

>Mycobacterium_genavense|WP_025734970.1
MTSDNRDERTARRVAELFNNDPQFRAAAPLPEVIEAACAPGLRLTEVLARLVEGYADRPALGERVRELVTDADGRTVLRLRPRFETISYRDVWDRVRAIATAWSSDPVTAGDVVATVGF
SSADYLVVDLVCAYLGLVTVPLQHNAPPARLRPIIEECEPKIVAVSAEYLDLAAESALTSTSLRQLMVFDYRAEVDEQRENFEQTRVRLQGSGTRVAVTTVDEVVARGRRLPAVAACAD
GDDQRLAMIMYTSGSTGTPKGAMYTERTVTTVWTTMRFLAPGLPVINANFMPLNHLGGRLPLASAFLSGGTSYFVPESDLSTLFEDWALVRPTEAAMVPRVVEMLYQHYRGAVDRGIAE
GADPATAEHDAATEMREQVLGGRVLGGFVGSAPLATEMKAFLDSMLDAHITDGYGLTETGMLTRDNVVNRNRVIDYKLIDVPELGYFLTDRPYPRGELLVKTDTMTPGYYKRPEVTAQV
FDEDGFYKTGDVMAEIEPDHLVYVDRRNNVLKLAQGEFVAVANLESIYAGAPLVRQIFVYGNSERSNLLAVIVPTPEALAEGNSPALKTAIHQSLRQTAAAAQLQSYELPVDFILETKP
FTDENGLLSGLGKQLRPRLKERYGEQLERLYSEIAAAQVDEIRVLREAAADRPAVETLAGACRALLGTSGADSESHFTDLGGDSLSALTLSRLLEDIFGVEVPVAVITSPANNIAKIAE
YIDVQRGAGIRRPGFSTVHGHGATCIAAADLTLDKFIDAQTLCTAPSLPHAGGAAHTVLLTGANGWLGRFLTLEWLDRLAERGGKLVTIVRGRDVDEARARLEKAFDSGDPELLSRFRE
LAATHLEVLAGDIGEENLGLSPATWHRLAETVDLIVHPAALVNHVLPYDQLFGPNVVGTAEIIRLAITSQIKPVTYLSTIAVASTVPPGQFQEDGDIRRVSPDRPLNGDYANGYANSKW
AGEVLLREANDLCGLPVAVFRSDMILAHTRYRGQLNVPDMFTRLIFSLLVTGIAPHSFYEHDGVGSRARAHYDGLPVDFIAEAITTIGSRIRKGYTSFDVMNPYDDGVSLDVFVDWLIR
AGNKIQRIPDYDEWLARFQTALTGLPERQRQQSVLPLLHAFHRPEKATRGACAPTEVFRAAVRADEIGPDKDIPHISAELIDKYADDLRQLALL

>Mycobacterium_intracellulare|WP_014386080.1
MTGDVKRERVAARIRELAATDEQFRNAQPDLSLQQAARQPGLRLPQILELFVEGYADRPAVGWRAKTLSTDPATGRTTTRLLPRFDTMTYRELWADVRAIAAAWRHDPVSPGDFVATVG
FASAEYLTLDLVCGYLGLVAVPLQHNTTPSRLRPIVDEVEPSILAAGVGYLDLAVEAASGSSSLRRLVVFDYQPEVDEQREALQRAQATLAAAGAAVTIETLDEIIERGRALPPEPMYT
GDTDQRLAMIMYTSGSTGLPKGAMYTEQMLAKVWTNELMPDFADTPVFNVNFMPLNHLGGRIPLSTAFQAGGTSYFVPESDLSTLFDDWNLVRPTEMGLVPRVAEMLYQRYQSAVDRLV
ASGADAGSAEARARAELREQVLGGRIVTAFCGTAPLAAEMRAFVETCLDVHVLDGYGLTEVGMVTKDGRMTRPPVLDYKLIDVPELGYFHTDKPYPRGELLVKSLTATPGYFKRPDVTA
NAFDPDGYYRTGDVMAELEPDRLAYVDRRNNVLKLAQGEFVAVARLEAVFASAPLIRQIFVYGNSERPYLLAVVVPTADAAERTDPEGLKAAVAESLRQSAQLAELQSYEVPVDFIVET
EPFSEDNGLLSGVGKLLRPKLKERYADRLEQLYAELAENRVTELRALREGADKHPVVFTLTRAAEALLGVAGGPPAPDALFIELGGDSLSALTFSNLLRDIFDVDVPVGMITGPATDLG
QLAEYVESERKSGSRRPTFATVHGRDAAEVRAAELTLDKFIDATTLAAAPNLPRATGTPHTVLLTGANGYLGRFLALEWLERLAETGGKLVSIVRATDTATAGKRLEAVFDSGDPQLLE
RFRTLAAEHLEVIVGDIGEPNLGLDQSTWQRLAQSVDLIVHPAALVNHVLPYDQLFGPNVVGTAELIRLAITTRIKPVTYLSTVAVAMTVDPGEFAEDGDIRAVSAVRPIDDSYANGYA
NSKWAGEVLLREAHDLCGLPVAVFRSDMILAHSRYAGQLNVPDAFTRLMFSLLTTGIAPTTFYRTDEHGNRAVAHYDGLPADFVAEAVTTLGEQMAAECRSYDVMNPHDDGVSLDVFVD
WLIAAGHDIRRIEDYDEWLGRFTTALRALPDKQRQHSVLPLLDAYREPAAPLRGAPAPTDVFRHAVRTAKIGADEDIPHLSAALIDKYVADLRLLGLV

>Nocardia_brasiliensis|AFU02004.1
MFAEDEQVKAAVPDQEVVEAIRAPGLRLAQIMATVMERYADRPAVGQRASEPVTESGRTTFRLLPEFETLTYRELWARVRAVAAAWHGDAERPLRAGDFVALLGFAGIDYGTLDLANIH
LGLVTVPLQSGATAPQLAAILAETTPRVLAATPDHLDIAVELLTGGASPERLVVFDYRPADDDHRAALESARRRLSDAGSAVVVETLDAVRARGSELPAAPLFVPAADEDPLALLIYTS
GSTGTPKGAMYTERLNRTTWLSGAKGVGLTLGYMPMSHIAGRASFAGVLARGGTVYFTARSDMSTLFEDLALVRPTEMFFVPRVCDMIFQRYQAELSRRAPAAAASPELEQELKTELRL
SAVGDRLLGAIAGSAPLSAEMREFMESLLDLELHDGYGSTEAGIGVLQDNIVQRPPVIDYKLVDVPELGYFRTDQPHPRGELLLKTEGMIPGYFRRPEVTAEIFDEDGFYRTGDIVAEL
EPDRLIYLDRRNNVLKLAQGEFVTVAHLEAVFATSPLIRQIYIYGNSERSFLLAVIVPTADALADGVTDALNTALTESLRQLAKEAGLQSYELPREFLVETEPFTVENGLLSGIAKLLR
PKLKEHYGERLEQLYRDIEANRNDELIELRRTAAELPVLETVTRAARSMLGLAASELRPDAHFTDLGGDSLSALSFSTLLQDMLEVEVPVGVIVSPANSLADLAKYIEAERHSGVRRPS
LISVHGPGTEIRAADLTLDKFIDERTLAAAKAVPAAPAQAQTVLLTGANGYLGRFLCLEWLQRLDQTGGTLVCIVRGTDAAAARKRLDAVFDSGDPELLDHYRKLAAEHLEVLAGDIGD
PNLGLDEATWQRLAATVDLIVHPAALVNHVLPYSQLFGPNVVGTAEIIRLAITERRKPVTYLSTVAVAAQVDPAGFDEERDIREMSAVRSIDAGYANGYGNSKWAGEVLLREAHDLCGL
PVAVFRSDMILAHSKYVGQLNVPDVFTRLILSLALTGIAPYSFYGTDSAGQRRRAHYDGLPADFVAEAITTLGARAESGFHTYDVWNPYDDGISLDEFVDWLGDFGVPIQRIDDYDEWF
RRFETAIRALPEKQRDASLLPLLDAHRRPLRAVRGSLLPAKNFQAAVQSARIGPDQDIPHLSPQLIDKYVTDLRHLGLL

>Mycobacterium_smegmatis2|WP_011728718.1
MTIETREDRFNRRIDHLFETDPQFAAARPDEAISAAAADPELRLPAAVKQILAGYADRPALGKRAVEFVTDEEGRTTAKLLPRFDTITYRQLAGRIQAVTNAWHNHPVNAGDRVAILGF
TSVDYTTIDIALLELGAVSVPLQTSAPVAQLQPIVAETEPKVIASSVDFLADAVALVESGPAPSRLVVFDYSHEVDDQREAFEAAKGKLAGTGVVVETITDALDRGRSLADAPLYVPDE
ADPLTLLIYTSGSTGTPKGAMYPESKTATMWQAGSKARWDETGVMPSITLNFMPMSHVMGRGILCSTLASGGTAYFAARSDLSTFLEDLALVRPTQLNFVPRIWDMLFQEYQSRLDNRR
AEGSEDRAEAAVLEEVRTQLLGGRFVSALTGSAPISAEMKSWVEDLLDMHLLEGYGSTEAGAVFIDGQIQRPPVIDYKLVDVPDLGYFATDRPYPRGELLVKSEQMFPGYYKRPEITAE
MFDEDGYYRTGDIVAELGPDHLEYLDRRNNVLKLSQGEFVTVSKLEAVFGDSPLVRQIYVYGNSARSYLLAVVVPTEEALSRWDGDELKSRISDSLQDAARAAGLQSYEIPRDFLVETT
PFTLENGLLTGIRKLARPKLKAHYGERLEQLYTDLAEGQANELRELRRNGADRPVVETVSRAAVALLGASVTDLRSDAHFTDLGGDSLSALSFSNLLHEIFDVDVPVGVIVSPATDLAG
VAAYIEGELRGSKRPTYASVHGRDATEVRARDLALGKFIDAKTLSAAPGLPRSGTEIRTVLLTGATGFLGRYLALEWLERMDLVDGKVICLVRARSDDEARARLDATFDTGDATLLEHY
RALAADHLEVIAGDKGEADLGLDHDTWQRLADTVDLIVDPAALVNHVLPYSQMFGPNALGTAELIRIALTTTIKPYVYVSTIGVGQGISPEAFVEDADIREISATRRVDDSYANGYGNS
KWAGEVLLREAHDWCGLPVSVFRCDMILADTTYSGQLNLPDMFTRLMLSLVATGIAPGSFYELDADGNRQRAHYDGLPVEFIAEAISTIGSQVTDGFETFHVMNPYDDGIGLDEYVDWL
IEAGYPVHRVDDYATWLSRFETALRALPERQRQASLLPLLHNYQQPSPPVCGAMAPTDRFRAAVQDAKIGPDKDIPHVTADVIVKYISNLQMLGLL

>Mycobacterium_smegmatis3|WP_015306631.1
MSTVSREERLARRISDLYATDQQFADARPSEAVAHAIESPALRLPQIIQTVIDGYAERPALGQRAVEFVTDPTTGRTSAALLPRFDTITYRELSERVDAVATALTQNPVRPGDRVAILG
FTSIDYTTVDMALLRAGAVSVPLQTSAPVAQLRPIAAETEPVAILSSVDFLDDAVELMLTGHLPERLVVFDYHAEVDDHREALASATARLAETPVVVETLAEVLARGNALPAHPAFDAG
DDNLALLIYTSGSTGTPKGAMYLAKAVANSWRRSSMAMWGNEGATPSITLNFMPMSHMMGRGILYATLGAGGTAYFVARSDLSTFFDDLSLVRPTQLSFVPRIWDMVFAEYQSEVDRRS
ADGGDRWAVEADVLADLRQNLLGGRFISAMTGSAPISSEMRTFVESLLDIHLTDGYGSTEAGAVFVDGQVQRPPVIDYKLVDVPDLGYFSTDRPYPRGELLVKSETMFPGYYKRPEITA
DVFDADGYYRTGDVVAELGPDQLQYLDRRNNVLKLSQGEFVTVAKLEAVFVDSPLIRQIFVYGNSARSYLLAVIVPTEEALARHDAAELKQLITESLQDVAKATGLQSYEIPRDFIIET
TPFTLENGLLTGIRKLARPKLKEFYGERLEQLYTDLADSQANELRELRQHGADRPVLETISRAAGALLGAAASELQPDAHFTDLGGDSLSALTFANLLHEIFEIDVPVGVIVSPANDLA
ALAAYIEAERQPGSKRPTFASVHGRDATEVYANDLTLDKFIDAKTLAAASSLPGPSSEVRTVLLTGATGFLGRYLALEWLERMDMVDGKVIALVRAKDDDAARERLDKTFDSGDPELLR
HYHELAADHLEVIAGDKGEANLGLDQQTWQRLADTVDLIVDPAALVNHVLPYSQLFGPNALGTAELIRIALTTKQKPFVYVSTIGVGAGIEPGQFTEDGDVRVYSATRKVDDTYANGYG
NSKWAGEVLLREAHDLGLPVSVFRCDMILADTTYAGQLNLPDMFTRLMLSLVATGIAPDSFYEVDANGNRQRAHYDGLPVEFIAEAISTLGAQVVDGFETYHVMNPYDDGIGLDEYVDW
LIEAGFPVERVGDYTTWLQRFDTAVRALPERQRQASLLPLLHNYQRPETPIRGSIAPTDRFRAAVQDAKIGPDKDIPHVTRDVIVKYITDLQLLGLL

>Mycobacterium_abscessus|WP_005099942.1
MTAGTAARVAKLFESDPQFRAAMPDPAVMDSLLAPGLRLSQVLHALLSGYAERPVMGFRSRESVVDTATGRTVDRLLPAFETITYGQLLEDISAILAEWQHGPMGAGDFIATIGFSSPD
YVTLDLATLMNGSVSIPLQHNTSVVQLRMMLEETSPRLVAASADCLDLAVEAAVGLTDLRRVVVFDYRAATDDHREKLATARERLHAAGMDVVVEPLAEVIGRGRDLPEPVLYTAGDDQ
RTALIMYTSGSTGAPKGAMFTEWTVTRFWSSGAAPNRDTPIINVNFLPLNHLAGRVGLLTAFIPGGTCYFVPESDLSTLFEDWQLARPTHMGVVPRVVDMLFQHYQTRVDALMAGGTDV
DTADRLAKTELREDVLGGRVVAGMLATAPLSPEMKAFLESSLDFHLLDLYGLTEVGGVFRDGKISRPPVLDYKLVDVPELGYYTTDKPHPRGELLVKSATATPGYYKRPDVTAEVFDAD
GYYRTGDVMAEVAPDQLVYVDRRNNVIKLAQGEFVAVANLETVYVGAPLVRQIFVYGNSERAYLLAVVVPTEEALRAPDPVELKNSIRESLQRTARSNHLHSYELPADFIIETTPFTIE
SGMLAAVGKPIRPKMIEHYGDRLEQLYVDLAEARVQELRQLRDTAQQRPVLDTVTEAAQALLGMSADAVRPDHHFIDLGGDSLSALTFSNLLRDLFDVEVPVGVITGPAADLRKLAAYI
QHEREHSTATAASVHGLDTTVISAAELTLDKFIDAETLYNASQLDVPAGTVATVLLTGANGYLGRFLCLEWLQRLSQTDGQLICLVRGDNDDQALARLVAAYGDTDRTLLEEFHTLARR
HLRVVAADIAQPRFGVDDATWEQLARDVDKIVHPAALVNHVLPYNQLFGPNVFGTAEVIRLALTTRIKPVTYLSTMAVAMTVPDFDEDGDIRTVSPTRHIDPGYANGYANSKWAGEVLL
REAHDLCGLPVSVFRSDMILTHRRYSGQLNVTDAFTRMLLSLVLTGIAPRSFYQGDGSGARPRAHYEGLPVDFVAEAITSLGLSSSEGFRSYDVMNPHDDGISVDTFVDWLVEDGHSID
IIDNYDEWLSRFETALRGLPDEQRRASVLPLLDAYRVPGNPRRAAATPNHVFRKAVQESNIGGDADIPQIDRALIAKYIADLRAHGLL

>Nocardia_brevicatena|WP_040834023.1
MSVDTRESRLERRIAELYATDEQFAAARPDEAVTAAIEQSGLRAARVAQTVMEGYAHRPAVGQRAVEFVTDPGTGRTSTELLPRFETLTYREVWDRAGAIATALTGDPVRPGDRVCVLG
FTSVDYTIIDMALMRLAAVAVPLQTGASAERLRPIIAETEPTVLAASIDYLDDAVELVLTGHAPTRLIVFDYRPRVDDQREAFAAATAKLADAAGPVIVETLADVVDRGTGLPPVPVPT
TDEPDSLALLVYTSGSTGAPKGAMITDGMMAEYWRASTSERWGQRSAEPSIVLSFMPMSHILGRAVLYMALGRGGTVYFAAKSDLSTLLDDLASVRPTQLTFVPRIWEMLFQRFQSEMY
RRASDGGDRATLEAEVTADLRRDLLGGRFLSATTGSAPMSTEMKAWVETFLDLHLRDGYGSTEAGSITLDGHVRRPPVVDYKLVDVPELGYFRTDRPYPRGELAVVSQSLTPGYYKRPD

VTAEVFDADGFYHTGDIFAEVGPDQLVYVDRRSFVLKLSQGEFVTVSKLEAVFAESPLVRQIYVYGNSTRSYLLAVVVPTEDAQARGDIEALKPLIGESLRKVAKTAGLQSYEIPRDLI
LDTTPFTMENGLLTGSRKLARPKLEEYYGPRLEQLYTELAEAETHELRTLRHSGAGRPVLETVSRAAGTLLGAAAGVLRPEAHFTDLGGDSLSALTFANLLRDIFDVDVPVGVIVGPAT
DLRALAAYIEQRRGGTERPTFTAVHGDGATELHARDLRLDKFLDTQTLEAAPNLPGPSPKVRTVLLTGATGFLGRYLALEWLQRMALVGGRLICLVRAKDDATARERSDNTFAGSDPRL
LAHYREPAAEHLEVLAGDKGEADLGLDRATWQRLADTVDLIVDPAALVNHVLPYSQLFGPNVVGTAELIRLALTTKQKPYTYVSTIGVSDQIDPSVFTEDADIRAIGPTRAIDDSYANG
YSNSKWAGEVLLREANDLCGLPVAVFRCDLILADTEYAGQLNVPDMFTRLMLSLLATGIAPGSFYELGPDGRRRRAHYDGLPVGFVAEAISTLGAQAAPGFRTYHVMNPYDDDISLDTY
VDWLIDAGHPIERIPDYGTWLRRFETAVRALPDRQRRHSLLPLLHSYRQPQRPIRGSIAPTERFRAAVQEAKIGPDKDIPHVTREIILEYATDLRQLGLL

>Nocardia_rhamnosiphila|WP_030520721.1
MSIETRETRLEHRIAELYRTDEQFAAARPDPAVTERAGKPGLRAFEIARTVMEGYADRPALGQRAIEYVTDERTGRTAAKLRPEFETVTYREVWDRAGAIAAALTADGVRPGDRVCLLG
FTSVDYTVIDVALTRMAAVAVPLPTSSATARLLPIVAETEPVVIAASIDRLADAVELALTGHSPARLIAFDHHPRDDDDNAIATAARARLAEADGTVVAETLAEVVARGAVLPPVPVPV
TEADSLALLIYTSGSTGAPKGAMLTERLVADHWRAASSEKWGQRGTEPAIGLGFMPMSHIMGRAILYMTLGRGGTVFFAATSDLSTLLDDLALVRPTQLSFVPRIWEMLFHRFEGEVAR
RGGDGADRTALEAEVAADLRRNLLGGRYLAATTGSAPISAEMRAWVESFLDMHVVDGYGATETGSIAVDGRVRRPPVSHYELVDVPELGYFRTDRPHPRGELVVRSDTLVPGYYKRPEV
TARVFDADGAYHTGDIFAEVGPDQLVYVDRRSFVLKLSQGEFVTVSKVEAVFAESPLVRQIYVYGNSTRSYLVAVVVPTAAARERDDSHVLKQLISESLQQVAKSAGLQSYEIPRDFLL
EYQRFTPENGLLTGIRKLARPALKEHYGPRLEQLYTELAEAEAEELRAVRHGAGDRPTIETVVRAAAALLGAATGEVRPDAHFTDLGGDSLSALTFGQLLREIFGVEVPVGFLIGPTAD
LRAVAHYIDEQRAGARRPTFAAVHGAEATEVHAGDLTLDKFLDEHTLAQAPHLPGPSATVRTVLLTGATGFLGRYLALEWLERMARAGGTLICLVRATDDTAARSRLDETFDSGDPVLT
ARYRELAAGHLEVIAGDKGRADLGLDHDTWQRLADTVDVIVDPAALVNHLLPYTELFGPNVVGTAELIRLALTTKQKPYTYTSTIAVGDQVDPANFTEDADIRVISPHRGIDDRYANGY
GNSKWAGEVLLREAADLCGLPVAVFRCDMILADTGYAGQLNVPDMFTRLMLSLEATGTAPRSFYELDPDGRPQRAHYDGLPVGFIAEAIVSLGARAGTGFRTYHVMNPYDDGISLDTYV
DWLIEAGHPIHRIPDYDSWLRRFETAVRALPERQRRYSLLPLLDSYRKPQRAVRGSIAPTERFRAAVRHAEIGADRDIPHVTPEIIVKYTTDLHLLGLL

>Nocardia_testacea|WP_039829215.1
MSIDTRESPLEHRIAELYRTDRQFAAARPDPAVTARAEKQGARPVEVAETVMQGYARRPAVGQRAIEFVTDPQTGRTSVALLPRFDTLTYGEVWDRAGALAAALSGDPVRPGDRVCVLG
FSSVDYTLVDLALVRLGAVAVPLQTSAPVTQLRSVVAETEPTVLAASIDHLDVAVDLVRTGPGAARLIVFDYHPQADDQRAAFEAVVARLSAAGSPVRVDDLSEVIAHGATLAPPPVPP
PAETDPPALLIYTSGSTGAPKGAMITERLVADQWRAATSERWGQPATEPAIALGFMPMSHIMGRAILYMTLARGGTVFFAARSDLSTLLDDLALVRPTQLVFVPRIWEMLYQRFRGEVQ
RRTAAGADPATVEEEVRTELREKLLGGRFLAATTGSAPISRELRTWVETLLGFHLVDGYGSTEAGSIAVDGRIRRPPVLDYELVDVPELGYFHTDRPYPRGELVIRSEALIPGYYKRPD
ATAEVFDADGAYHTGDIFAEVGTDELVYLERRGFVLKLSQGEFVAVSTVEAALAESPLVRQVYVYGNSTRSYPLAVVVPTAEAIEQGDIEALRPLLAESLRQVAKTAGLQSYEIPRDLL
VETRPFTPENGLLTGIGKLARPALKQRYGPRLEQLYADLAAAEAEELRALHRSGDRPTVDTVVRAAGALLGSATGDLRPDAHFTELGGDSLSALTFAQLLREIFDIEVPVGTIIGPATD
LRALADYIDGQRRGGHRPTFASVHGAGATEVYARDLTLDQFIDAGTLAAAPGLPEPSAPARTVLLTGATGFLGRYLVLEWLRRLNHSGGTLICPVRAPDDAAARARLDEVFETGDPALS
ARYRESAAGHLEVIAGDKSAPRLGTTRAVWERLADTVDVIVDPAALVNHMLPYRELFGPNVVGTAELIRLALTTRQKPYTYISTIGVSDQIDRAAFTEDADIRVISPRRAIDGGYANGY
SNSKWAGEVLLREAADLCGLPIAVFRCDMILADTEYSGQLNLPDMFTRLVLSVEATGTAPGSFYELDAEGRRPRAHYDGLSVGFIAEAVATLGARPGSGFRTYHVMNPYDDGISLDTYV
DWLIDAGHPIERISGYDDWLQRMETAVRALPDRQRRYSLLPLLDNYRKPQQAVRGSIAPTERFRDAVRHAEIGAEKDIPHVTPDIIVKYTTDLERLGLL

>Mycobacterium_tuberculosis|WP_003413409.1
MSINDQRLTRRVEDLYASDAQFAAASPNEAITQAIDQPGVALPQLIRMVMEGYADRPALGQRALRFVTDPDSGRTMVELLPRFETITYRELWARAGTLATALSAPAIRPGDRVCVLGFN
SVDYTTIDIALIRLGAVSVPLQTSAPVTGLRPIVTETEPTMIATSIDNLGDAVEVLAGHAPARLVVFDYHGKVDTHREAVEAARARLAGSVTIDTLAELIERGRALPATPIADSADDAL
ALLIYTSGSTGAPKGAMYRESQVMSFWRKSSGWFEPSGYPSITLNFMPMSHVGGRQVLYGTLSNGGTAYFVAKSDLSTLFEDLALVRPTELCFVPRIWDMVFAEFHSEVDRRLVDGADR
AALEAQVKAELRENVLGGRFVMALTGSAPISAEMTAWVESLLDVHLVEGYGSTEAGMVLNDGMVRRPAVIDYKLVDVPELGYFGTDQPYPRGELLVKTQTMFPGYYQRPDVTAEVFDPD
GFYRTGDIMAKVGPDQFVYLDRRNNVLKLSQGEFIAVSKLEAVFGDSPLVRQIFIYGNSARAYPLAVVVPSGDALSRHGIENLKPVISESLQEVARAAGLQSYEIPRDFIIETTPFTLE
NGLLTGIRKLARPQLKKFYGERLERLYTELADSQSNELRELRQSGPDAPVLPTLCRAAAALLGSTAADVRPDAHFADLGGDSLSALSLANLLHEIFGVDVPVGVIVSPASDLRALADHI
EAARTGVRRPSFASIHGRSATEVHASDLTLDKFIDAATLAAAPNLPAPSAQVRTVLLTGATGFLGRYLALEWLDRMDLVNGKLICLVRARSDEEAQARLDATFDSGDPYLVRHYRELGA
GRLEVLAGDKGEADLGLDRVTWQRLADTVDLIVDPAALVNHVLPYSQLFGPNAAGTAELLRLALTGKRKPYIYTSTIAVGEQIPPEAFTEDADIRAISPTRRIDDSYANGYANSKWAGE
VLLREAHEQCGLPVTVFRCDMILADTSYTGQLNLPDMFTRLMLSLAATGIAPGSFYELDAHGNRQRAHYDGLPVEFVAEAICTLGTHSPDRFVTYHVMNPYDDGIGLDEFVDWLNSSGC
TIQRIADYGEWLQRFETSLRALPDRQRHASLLPLLHNYREPAKPICGSIAPTDQFRAAVQEAKIGPDKDIPHLTAAIIAKYISNLRLLGLL

>Mycobacterium_avium|WP_024637302.1
MSTATHDERLDRRVHELIATDPQFAAAQPDPAITAALEQPGLRLPQIIRTVLDGYADRPALGQRVVEFVTDAKTGRTSAQLLPRFETITYGEVAQRVSALGRALSDDAVHPGDRVCVLG
FNSVDYATIDMALGAIGAVSVPLQTSAAISSLQPIVAETEPTLIASSVNQLSDAVQLITGAQAPTRLVVFDYHPQVDDQREAVQDAAARLSGTGVAVQTLAELLERGKDLPAVAEPPAD
EDSLALLIYTSGSTGAPKGAMYPQSNVGKMWRRGSKNWFGESAASITLNFMPMSHVMGRSILYGTLGNGGTAYFAARSDLSTLLEDLELVRPTELNFVPRIWETLYGEFQRQVERRLSE
AGERRAVEAEVLAEQRQYLLGGRFTFAMTGSAPISPELRNWVESLLEMHLMDGYGSTEAGMVLFDGEIQRPPVVDYKLVDVPDLGYFSTDRPHPRGELLLRTENMFPGYYKRAETTAGV
FDEDGYYRTGDVFAEIAPDRLVYVDRRNNVLKLAQGEFVTLAKLEAVFGNSPLIRQIYVYGNSAQPYLLAVVVPTEEALASGDPETLKPKIADSLQQVAKEAGLQSYEVPRDFIIETTP
FSLENGLLTGIRKLAWPKLKQHYGERLEQMYADLAAGQADELAELRRNGAQAPVLQTVSRAAGAMLGSAASDLSPDAHFTDLGGDSLSALTFGNLLREIFDVDVPVGVIVSPANDLAAI
ASYIEAERQGSKRPTFASVHGRDATVVRAADLTLDKFLDADTLAAAPNLPKPATEVRTVLLTGATGFLGRYLALEWLERMDMVDGKVIALVRARSDEEARARLDKTFDSGDPKLLAHYQ
QLAADHLEVIAGDKGEANLGLRQDVWQRLADTVDVIVDPAALVNHVLPYSELFGPNALGTAELIRLALTSKQKPYTYVSTIGVGDQIEPGKFVENADIRQMSATRAINDSYANGYGNSK
WAGEVLLREAHDLCGLPVAVFRCDMILADTTYAGQLNLPDMFTRLMLSLVATGIAPGSFYELDADGNRQRAHYDGLPVEFIAAAISTLGSQITTGFQTYHVMNPYDDGIGLDEYVDWLV
DAGYSIERIADYSEWLRRFETSLRALPDRQRQYSLLPLLHNYRTPEKPINGSIAPTDVFRAAVQEAKIGPDKDIPHVSPPVIVKYITDLQLLGLL

>Mycobacterium_colombiense|WP_007774522.1
MSTAIHDEELDRRIEHLVATDPQFAATRPDPAITAATEQPGLRLPQIIRTVLDGYADRPALGQRVVEFVKDAKTGRTSAELLPRFETVTYGELGQRVSALGRAWASDSVSPGDRVCVLG
FNSVDYATIDIALGTIGAVSVPLQTSAALSSLQPIVAETEPSLIASSVNQLPDAVELILAGHVPGRLVVFDYQPEVDDQREAVESAVARLAGIGVVVEQLADVLRRGKDLPPVPEQQTD
EDSLALLIYTSGSTGAPKGAMYPQSNVGKMWRRGSKNWFGESAASITLNFMPMSHVMGRGILYGTLGNGGTAYFAARSDLSTLLEDLELVRPTEMNFVPRIWETLYGEFQRQVERRLTD
GADREAVEAEVLEEQRQYLLGGRFIFAMTGSAPTSPELKKWAESLLQMHLMDGYGSTEAGMVLFDGEIQRPPVIDYKLVDVPDLGYFSTDRPYPRGELLLRTENMFPGYYKRAETTAGV
FDDDGYYRTGDVFAEIAPDRLVYVDRRNNVLKLAQGEFVTLAKLEAVFGNSPLIRQIYVYGNSAQPYLLAVVVPTEEALADNDIEALKPKIADSLQKVAKETGLQSYEVPRDFIIETTP
FTLENGLLTGIRKLAWPKLKQHYGERLEQMYADLAAGQANELAELRRSGAQAPVLQTVSRAAAAMLGAATGDLSGDAHFTDLGGDSLSALTFGNLLREIFDVDVPVGVIVSPANDLAAI
AAYIEAERQGSKRPTFAAVHGRGATTVRAGDLTLDKFLDEALLAGAPSLPKPSTEVRTVLLTGATGFLGRYLALDWLERMDMVDGKVIALVRARSDEEARARLDKTFDSGDPTLLAHYQ
ELAADHLEVIAGDKGEANLGLDQQTWQRLADTVDIIVDPAALVNHVLPYSELFGPNALGTAELIRIALTSKQKPYTYVSTIGVGDQIQPGKFVENADIRQISATREINDNYANGYGNSK
WAGEVLLREAHDLCGLPVSVFRCDMILADTTYAGQLNLPDMFTRLMLSVVATGIAPRSFYELDAEGNRQRAHYDGLPVEFIAAAISTLGTQITTGFQTYHVMNPYDDGIGLDEYIDWLV
EAGYSIERIPDYSEWLRRFETSLRALPDRQRQYSLLPLLHNYQKPEKPINGSMAPTDVFRAAVQEAKIGPDKDIPHVSAPVIVKYITNLQLLGLL

>Mycobacterium_leprae|WP_041322427.1
MWRTKQEKQLARRVDDLTANDPQFAAAKPDPAVAAALAQPGLRLPQIIQTALDGYAERPALGQRVAEFTKDPKTGRTSMELLPSFETITYRQLGDRVGALARAWRHDLLHAGYRVCVLG
FNSVDYAIIDMALGVIGAVAVPLQTSAAITQLQSIVTETEPSMIATSVNQLPDTVELILSGQAPAKLVVFDYHPEVDEQHDAVATARARLADSSVVVESLTEVLGRGKTLPATPIPVAD
SADPLALLIYTSGSTGAPKGAMYLQSNVGKMWRRSDGNWFGPTAASITLNFMPMSHVMGRGILYGTLGNGGTAYFAARSDLSTLLEDLKLVRPTELNFVPRIWETLYDESKRAVDRRLA
NSADRAAIKAEVMDEQRQSLLGGRYIAAMTGSAPTSPELKHGVESLLEMHLLEGYGSTEAGMVLFDGEVQRPPVIDYKLVDVPDLGYFSTDQPYPRGELLLKTQNMFPGYYKRPEVTAT
VFDSDGYYQTGDIVAEVGPDRLVYVDRRNNVLKLAQGQFVTVAKLEAAFSNSPLVRQIYIYGNSAHPYLLAVVVPTEDALATNDIEVLKPLIIDSLQKVAKEADLQSYEVPRDLIVETT
PFSLENGLLTGIRKLAWPKLKQHYGARLEQLYADLVEGQANALHVLKQSVANAPVLQTVSRAVGTILGVATTDLPSNAHFTDLGGDSLSALTFGSLLRELFDIDVPVGVIVSPVNNLVA
IADYIERERQGTKRPTFIAIHGRDAGKVHASDLTLDKFIDVSTLTAAPVLAQPGTEVRTVLLTGATGFLGRYLALKWLERMDLVEGKVIALVRAKSNEDARARLDKTFDSGDPKLLAHY
QELATDHLEVIAGDKGEVDLELDRQTWRRLADTVDLIVDPAALVNHVLPYSELFGPNTLGTAELIRIALTSKQKPYIYVSTIGVGNQIEPAKFTEDSDIRVISPTRNINNNYANGYGNS
KWAGEVLLREAHDLCGLPVTVFRCDMILADTSYAGQLNVPDMFTRMMLSLAATGIAPGSFYELDAESNRQRAHYDGLPVEFIAEAISTLGDQSLDGFTTYHVMNPHDDGIGMDEFVDWL
IDAGCPIQRINDYDEWLRRFEISLRALPERQRHSSLLPLLHNYQKPEKPLHGSLAPTIRFRTAVQNANIGQDKDIPHISPAIIAKYVSDLQLLGLV

>Mycobacterium_parascrofulaceum|WP_007172010.1
MSTIDHDERLERRIEELTANDPQFAAARPDPAIEAALEKPGLRLPQVIRTVLAGYADRPALAHRAVEFVADSASGRTTLELLPRFETLTYRDLGDRVGALGRAWAHDEVRVGDRVCILG

FNSVDYATIDMALATISAVSVPLQTSASLTSLQPIVAETEPTVIAASANQLPDAVELILTGQRPAKLVVFDYHPEVDDEREAVETARTRLADTGVVVETLAEVLERGKALPDTELPAGD
EPDPLALLIYTSGSTGAPKGAMYPQSNVGKIWRRGSRNWFGESAASITLNFMPMSHVMGRGILYGTLGNGGTAYFAAKSDLSTLLEDLELVRPTELNFVPRIWETLFGEFQRQVARRLS
EGGDRAAVEAEVLAEQREYLLGGRFIFAMTGSAPTSPELRNWVESLLQMHLMDGYGSTEAGMVLFDGEIQRPPVIDYKLVDVPDLGYFGTDRPHPRGELLLRTENMFPGYYKRAEITAN
VFDEDGYYRTGDVFAEVAPDKLVYVDRRNNVLKLAQGEFVTLAKLEAEFGNSPLVRQIYVYGNSSQPYLLAVVVPTQEALGRWDSEALKGKIADSLQNVARQAGLQSYEVPRDFLIETT
PFSLENGLLTGIRKLAWPKLKQHYGERLEQLYAELAEGQANELAELRRNGADAPVLQTVSRAAAAMLGTAATELTPDAHFTDLGGDSLSALTFGNLLREIFDIDVPVGVIVSPASDLQA
IADYIEGERRGSKRPTFAAVHGRDATEVRAADLTLDKFLEAETLAAAPSLPKPTAEVRTVLLTGATGFLGRYLALEWLERMDLVDGRVIALVRAKSDDEARARLDRTFDSGDPKLLAHY
RELAADHLEVIAGDKGEPNLGLDQQTWQRLADTVDLIVDPAALVNHVLPYSELFGPNALGTAELIRIALTTKLKPYTYVSTIGVGDQITPGQFVEDADIRQVSATRAVNDNYANGYGNS
KWAGEVLLREAHDLCGLPVAVFRCDMILADTTYAGQLNLPDMFTRLMLSLVATGVAPGSFYELDADGNRQRSHYDGLPVEFIAAAISTLGTQVLDGFQTYHVMNPYDDGIGLDEYVDWL
IDAGHRIQRIADYGEWLRRFEGTMRGLPERQRQYSLLPLLHNYQQPEKPINGSMAPTDRFRAAVQEAKIGPDKDIPHVSPPIIVKYATDLQLLGLL

>Mycobacterium_lepromatosis|WP_045842500.1
MNTQEEQLARRVDYLTANDPQFAAAKPDPAVVAALAQPGLRLPQIIQTTLDGYAERPALGQRVVEFIKDPKTGRTSIELLPCFETITYRELSDRVGALARAWIHDLLHAGDRVCVLGFN
SVDYAIIDMALGVISAVAVPLQTSAAITQLQPIVIETEPRVIAASVNQLPDTVELILSGPAPAKLVVFDYHPEADEQRDAVATARERLVDNNVVVESLIEVLDRGKTLPATPIPVADSD
DPLALLIYTSGSTGAPKGAMYPQSNVGKMWRRSDGNWFRSTAASITLNFMPMSHVMGHGILYGTLGNGGTAYFAARSDLSTLLEDLKLVRPTELNFVPRIWETLYDECQHAVDRRLTNS
ADRAAIEAEVMDEQRQSLLGGRYIAAMTGSAPTSPELKHGVESLLEMHLLEGYGSTEAGMVLFDGEVQRPPVIDYKLVDVPDLGYFSTDQPYPRGELLLKTQNMFPGYYKRPEVTATVF
DGDGYYQTGDIVAEVGPARLVYVDRRNNVLKLAQGQFVTVAQLEAAFSNSPLVRQIYIYGNSAHPYLLAVVVPTEDALATNDIEALKPLIIDSLQEVAKEAELQSYEVPRDLIIETTPF
SLENGLLTGIRKLAWPKLKQHYGARLEQLYADLAEGQTNALHALRQSGAEAPVLQTVSRAAGTILGAATTDLPSNAHFTDLGGDSLSALTFSNLLRELFNIDVPVGVIVSPVNDLAAIA
DYIESEQQGTKRPTFTAIHGRDAGEVHASDLTLDKFIDVSTLTAAPMLAQPDAEVRTVLLTGATGFLGRYLALKWLERMDLVDGKVIALVRAKSNEEARARLGKTFDSGDPKLLAHYQE
LAADHLEVIAGDKGEVELELDRQTWRRLADTVDLIVDPAALVNHVLPYSELFGPNALGTAELIRIALTSKQKPYIYVSTIGVGDQIEPAKFTEDSDIRVTSPTRKINDNYANGYGNSKW
AGEVLLREAHELCGLPVAVFRCDMILADTSYAGQLNVPDMFTRMMLSLAATGIAPGSFYELDAESNRQRAHYDGLPVEFIAEAISTLGAQSLDGFATYHVMNPHDDGIGMDEFVDWLID
AGCPIQRINDYDEWLRRFEISLRALPERQRHNSLLPLLHNYQKPEKPLNGSLAPTDRFRTAVQESKIVQDKDIPHISSAIIGKYVSDLQLLGLL

>Mycobacterium_triplex|WP_036468454.1
MSTTTREERLERRIENLTATDPQFAAAKPDPAVVEALEQPGLQLPQIIQTVLEGYGDRPALGQRAVEFVKDAKTGRTVLGLLPRYDTITFGELRERVDAVARALTRDGLRPGDRVAALG
FNSVDFTTIDIALGMVGAVSVPLQTSAAVAQLQPIVTETEPAVFAASTNQLSDAVELILSTHRPTKLVVFDYHPEVDDEREAVESARARLADTALTVEPLADLLQRGATLPATPAAAVD
DDELALLIYTSGSTGAPKGAMYPRRNVGKMWRRSGRNWFGETVASITLNFMPMSHVMGRGILYGTLGNGGTAYFAARSDLSTLLEDLELVRPTEMNFVPRIWETLYGEYQRRVDRGGSE
ADVMNEISQHLLGGRFIFAMTGSAPTSGDLKAWVEELLDMHLLDGYGSTEAGMVLFDGEVQRPPVIDYKLVDVPDLGYFATDRPYPRGELLLKTENMFPGYYKRPETTAGVFDPDGYYR
TGDVVAEIAPNKVVYVDRRNNVLKLAQGEFVTVAKLEAVFGNSPLVRQIYVYGNSAHPYLLAVIVPSEEALERYGADELKGRIADSLQTVAKEAGLQSYEVPRDFLIETTPFTLENGLL
TGIRKLAWPKLKQQYGERLEQLYADLAAGQANELGELRRSGATAPVLQTVSRAAAALLGAASTELTPDAHFTDLGGDSLSALTFGNLLHEIFEVDVPVGVIVSPANDLAAIAAYIESER
QGSKRPSFASVHGRDAVEVHASDLTLDKFIDAATLAAAPNLPAPASEVRTVLLTGATGFLGRYLALQWLERMDLVDGKVIALVRAKTDADARARLDKTFDSGDPKLLAHYRELAADHLE
VIAGDKGEADLGLDAATWRRLADTVDLIVDPAALVNHVLPYSELFGPNAVGTAELIRVALTGKKKPYTYVSTIGVGDQIKPGAFVEDADIREISATRQINDSYANGYGNSKWAGEVLLR
EANDLCGLPVAVFRCDMILADTSYAGQLNVPDMFTRMMLSLVATGIAPGSFYERPAEGNRQRAHYDGLPVEFIAEAVSTLGAQNVQSFQTYHVMNPYDDGIGMDEFVDWLIADGNAIQR
IDDYGDWLPRFETALRGLPEKQRNASLLPLLHNYQKPEQPIRGSMAPTDRFRAAVQDAKVGPDKDIPHIGAPIIAKYVSDLRLLGLL

>Mycobacterium_xenopi|EID15772.1
MDTREDQLERRIAALTANDPQFAAARPDEAVATAVQRPGLRLPEVIETVLQGYADRPALGQRAVEFVKDPNTGRTSAHLLPRFDTITYRELADRVGALASAWAREAVSPGDRVAILGFT
SVDYTTIDVTLARIGAVSVPLQTSAALAQLRPIVVETEPTVIAASVDYLSDAVELIRTGHAPARLVVFDHHPEVDDHREALDAARGRLAGHAVIVETLAEVLERGTSLPAPTVAAEDND
LALLIYTSGSTGAPKGAMYPQRNVAKMWQRSSRNWFGPSAASITLNFMPMSHVMGRGILYGTLGNGGTAYFGATSDLSTLLEDLTLVRPTELNFVPRVWDTLHAEFLTRVDRLTAEGAD
RASAEALVMGDLRDNLLGGRAIFAMTGSAPISSQLKTWVESLLGIHLLDGYGSTEAGMVLYDGVVQRPPVIDYKLADVPDLGYFSTDRPFPRGELLLKTENMFPGYYKRPEITAGVFDD
DGYYRTGDVVAEVGPDRLVYVDRRNNVLKLAQGEFVTVAKLEAGFNNSPLVRQIYIYGNSAHPYLLAVVVPTDVNASKSAIAESLQRVAKDAGLQSYEVPRDFLIEPEPFTLENGLLTG
IRKLAWPKLKERYGERLEQLYAELDRSQADELSELRRSGAQRPVLETVTRAAGALLGAAASELQPDAHFTDLGGDSLSALTFGNLLREIFDVDVPVGVIVSPASDLQAIAGYIEAERQG
SKRPTFASVHGREAVEVRARDLRLDKFLDARTLEYVPALPGPSTELRTVLLTGATGFLGRYLALEWLERMDAVDGTVIALVRAKDDAAARERLDRTFDSDPKLRAHYRALAADHLEVVA
GDKGEANLGLSQQVWQRLADTVDVIVDPAALVNHVLPYSELFGPNVLGTAELIRLALTTKIKPYTYVSTIGVGDQIEPGKFTEDADIRVISPTRRISDSYANGYGNSKWAGEVLLREAH
DRCGLPVAVFRCDMILADTTYAGQLNLPDMFTRLMLSLAATGIAPRSFYELDAEGNRQRAHYDGLPVEFIAKAVSTLGAQTVEGYQTYHVMNPHDDGIGLDEYVDWLIEAGYPIRRVDD
YADWLQRFETAMRALPDRQRRYSLLPLLHNYQKPEKPMRGSMAPTDRFRAAVQEAKIGPDKDIPHVTREVIVKYATDLQLLGLL

>Mycobacterium_heraklionense|WP_047319167.1
MSTVADEEQLARRIADLVATDPQFAAARPDPAVAAAVEGQSRLAQIARTVFDGYAERPALGQRAVEYVTDPATGRTAAALLPHYETITYAELGERVRAAAAALVAGAVRPGDRVAVLGF
TSVDYTVIDVALGQIGAVSVPLQTSAAISTLVPIVTETEPRVIAASVDSLPDAVELVRTGPAPTRLVVFDYHEQVDDHREALADARARLAGVEVVVEPLADLLTRGRSLSLPQVDSGDD
ADPLALLVYTSGSTGAPKGAMYPERNVARMWVRSTKNWFGPTAVSITLNFMPMSHIMGRGILYGTLGNGGTAYFSARSDLSTFLEDLALVRPTELNFVPRIWEMLYQHFRSEVDGLGGD
RAAAEAQVLAQMRTELLGGRCVFAMTGSAPISDELRSWVDQLTEQHVLNGYGSTEAGMVLFDGEVQRPPVLDYKLVDVPDLGYFGTDTPHPRGELLLKTENLFPGYYKRPEVTAEVFDA
DGYYRTGDVVAEVAPDHLVYVDRRNNVLKLAQGEFVTLAKLEAVFGNSPLVRQIYVYGNSAQPYLLAVVVPTPEAQARFDSAALKAGIAESLQQVAKDADLQSYEVPRDFIVETEPFTL
ENGLLTGIRKLAWPKLKAHYGDRLERRYAELAEGQASELSELRQHGAQRPALETVGRAAAALLGASAGEVSADAHFTDLGGDSLSALTFGNLLRDIFGVDVPVGVIVSPANDLAAIADY
ITAERNGSGRPSFASVHGRDAVEVAAADLTLDKFLDAATLAAAPSLPGPSEQVRTVLLTGATGFLGRYLALEWLQRLAPVDGKLICLVRAKSDEDARRRLDSVFDSGDARLLARYTELA
AKHLEVLAGDKGEANLGLDQEVWQRLADTVDVIVDPAALVNHVLPYSELFGPNALGTAELIRVALTGKIKPFTYFSTIGVGDQIPLGKFTEDADVRVMSSVRAINDGYANGYGTSKWAG
EVLLREANDLCGLPVAVFRCDMILADTSYAGQLNLPDMFTRMMFSLVATGVAPGSFYELSADGQRQRAHYDGLPVEFIAEASTTLGSFASGGYTTYHVMNPYDDGLGMDEFVDWLIEAG
YPIRRIADYGDWLQRFETTLRALPEHQRQASLLPLLHNYQKPQPPICGSIAPTDRFRAAVQSAGIGPDPDIPHVTPAVIVKYITDLQLLGLL

>Mycobacterium_mageritense|WP_036441155.1
MSTETREERLARRITELSATDPQFAAAIPDEAVTAAVDEPGLPLPQIVQTVLQGYSDRPALGERAVEFVADPATGRTMARLLPRFDTITYGQLWDRVRATAAAWHEAGVTAGDRVAILG
FTSADYTALDTALGQLGAVSVPLQTSSSPAALAPIVVETQPRVIAASVDHLPDAVELALTAHAPAQLVVFDHHPEVDDHRDALVAARERLDAAGLVVPLVTLAELVEQGRELPQAPAPQ
VDESDPLALLIYTSGSTGAPKGAMYQQSAVAKFWRRNSKAWFGPTAASINLSFMPMSHVMGRGILYASLAAGGTAYFAARSDLSTLLEDLALARPTELNFVPRVWEMIYGEFQSRVDRQ
AADGADRADVEAAVIADVRDHLLGGRYISAMTGSAPISAELKAWVEQSLDIHLLEGYGSTEAGMVLFDDQIQRPPVIDYKLVDVPDLGYFGTDKPHPRGELLVKTDNMFPGYYKRPEVT
AGVFDEDGFYKTGDIVAEVAPDHLKYVDRRNNVLKLAQGEFVTVAKLEAVFGNSPLVRQIYIYGNSARPYLLAVVVPTEDALDRHEPSELKALIAESLQDVAKAADLQSYEIPRDFLVE
TTPFTLENGLLTGIRKLAWPKLKAHYGDRLEQLYAELADVQANELRALRANGADAPVLETVSRAAGALLGASAGDVQPDAHFTDLGGDSLSALTFGNLLQEIFDVEVPVGVIVSPANDL
AAIAAHIESARHGSKRPTFASVHGRHAAEVSAADLTLDKFIDAETLAAAPSLPKAGSEVRTVLLTGATGFLGRYLALEWLERMDLVDGKVITLVRAKSDADARARLDATFDSGDPKLLA
HYRELAADHLEVLAGDKGEADLGLDRETWQRLADTVDLIVDPAALVNHVLPYGELFGPNALGTAELLRIALTTKIKPYTYVSTIGVGDQVSPGTFVESGDIRAISPIRRIDDSYANGYG
NSKWAGEVLLREANDLCGLPVAVFRCDMILADTTYSGQLNLPDMFTRMMFSLVASGIAPGSFYQLGADGKRQRAHYDGLPVEFIAEAISTLGAQSVDSFETYHVMNPYDDGIGMDEFVD
WLVEAGYPIQRIADYREWVQRFESTLRALPDRQRQASLLPLLHNYQQPAVPLNGAMAPTDVFRAAVQEAKIGPDKDIPHVTREVIVQYITNLEQLGLL

>Mycobacterium_fortuitum|WP_003880694.1
MSFDTRDEQLATRIADLTATDPQFAAAIPSDTVTASVDVPGLLLPEIVQRVLEGYAERPALGERALEFVADPATGRTTARLLPRFDTISYGQVWDRVRALAAALHASGVAAGDRVAILG
FTSADYTVIDTALGQIGAVSVPLQTSSSPEALAPIVTETEPRVIAASVDHLADAVELALTAHAPAQLVVFDHHPEIDDHREAVASAAERITAAGASIAVDTLAGLLDRGSNLPAPEAPK
ADGSDPLALLIYTSGSTGAPKGAMYLQSAVAKFWRRNSKAWLGPVSSAINLSFMPMSHVMGRGILYASLAAGGTCYFAARSDLSTLLEDLALTRPTELNFVPRVWEMIHSEYQSRVDQR
LAEGRDREAVEAEVLAEVRDKVLGGRFVAAMTGSAPISAELKTWTQDMLGIHLLEGYGSTEAGMALFDGVVQRPPVIDYKLVDVPDLGYFGTDQPHPRGELLIKTENLFPGYYKRPEVT
ASVFDEDGFYRTGDVVAEIGPDQLRYVDRRNNVLKLAQGEFVTLAKLEAVFGNSPLVQQIYVYGNSAQPYLLAVVVPTDPSVSKEAIAESLQEVAREADLQSYEIPRDFIVETTPFSLE
NGLLTGIRKLAWPKLKAHYGERLEQLYAELAETQAAELRELRSASADAPVVETVSRAAGALLGAAASDLGPDAHFTDLGGDSLSALTFGNLLREIFDVDVPVGVIVSPATDLAGIAEYI
ETQRSGSKRPTYASVHGRHAAEVSAADLTLDKFLDADTLAAAPNLPKAGSEVRTVLLTGATGFLGRYLALEWLERMDLVDGKVIALVRAKSDEEARTRLDATFDSGDAKLLAHYQNLAA
DHLEVVAGDKGEENLGLDQQTWQRLADEVDLIVDPAALVNHVLPYSELFGPNALGTAELIKIALTTKIKPYTYVSTIGVGDQIEPGKFVENVDVREMSAVRKINDGYANGYGNSKWAGE
VLLREANDLCGLPVAVFRCDMILADTSYSGQLNLPDMFTRMMLSLVASGIAPKSFYELDSEGNRQRSHYDGLPVEFIAESISTLGGQSVESFETYHVMNPYDDGLGMDEFVDWLIEAGY
PIERIEDYGQWVQRFESTLRALPDKQRQASLLPLLHNYQKPERPMLGALAPTDHFRAAVQEAKIGPDKDIPHVSPAVIVKYITDLQQLGLL

>Mycobacterium_vaccae|WP_003933798.1
MSTDTREGRLARRIADLFATDPQFAAAVPDETVAAAVEEHAAHLPDIMRTVLDGYADRPALARRAVRFVEDATGRTVAELLPHFETITYAELAHRIHGVTAALTDVHPGDRVALLGFTS
VDYTTVDMALSMLGAVLVPLQTSAPLSTLRPIIAETEPVLIASSVDTLDDAVALALDAPDAARLVVFDHRAEVDDHRDALTSATARLRAAGSPLEIETLAEVIARGSTMPAREQFSPDA
DTLMLLIYTSGSTGAPKGAMYTERLVATTWRRSSRSFWGDHGPLPSITLNFLPMSHVMGRGLLYATLGAGGTAYFAAKSDLSTFLEDLALVRPTQLSFVPRIWDIIFAEVAKELERRTA
DAADVLADLRQSLLGGRYVSAMTGSAPLSAEMESFVEQLLDMHLIDGYGSTEAGAVLVDGQIQRPPVIDYKLVDVPDLGYFSTDRPHPRGELLVRSETLFPGYYKRPDITAEMFDEDGY
YRTGDIVAETAPDRLTYLDRRNNVLKLSQGEFVTVSKLEAVFGDSPLIHQIYVYGNSARPYLLAVVVPTDAALAREDVKTAVAESLQDVARAADLQSYEIPRDFLIETTPFTLENGLLT
GIRKLARPALKERYGDRLEALYAELAEGQTDELRELRRSGAERPVQETVLRAAAALLGAAATDLQPDAHFTDLGGDSLSALTFGNLLHDIFGVEIPVGVIVSPATDLQALAGHIETART
TGSARPSFTSVHQHPVTEVYARDLTLDKFVDTDTLAAAPALPGPAAEIRTVLLTGATGFLGRYLALEWLERLSLVGGTLICLVRAKDDAAARDRLDRTFDSGDPKLLEHYRRLAADHLE
VVAGDKGDADLGLDARTWQRLADTVDLIVDPAALVNHVLPYNQLFGPNVVGTAELIRLALTTKLKPFTYVSTMAVGAGVEPGRFTEDGDVRQISATRKVDDSYANGYGTSKWAGEVLLR
EAHDLCGLPVSVFRCDMILADTTYAGQLNLPDMFTRLILSLVATGVAPESFYRLDADGRRPRAHYDGLPVEFVAEAISTLGAQVVSGFETYHVMNPHDDGIGLDEFVDWLIEAGYPVRR
VGDYPTWLQRFTVAVNALPDRQRQASLLPLLHNYQQPEIPVCGAVAPTDRFREAVQDAKIGPDKDIPHLSAQVIVKYVTDLQLLGLL

>Mycobacterium_obuense|WP_048423142.1
MPTDTREERLAHRIDDLSATDTQFAAALPDEAIAEAIEDPQLRLPQIIATVLDGYADRPALGQRAVRLVADPHTERTEAQLLPHFDTITYGELSTRIHHLLTALTDVDPGDRVAILGFT
SVDYTVIDTTLVLRGAVSVPLQTSAPAATLRPIVAETEPVVFAASVDHLSDAVDLVADAESVGRLIVFDYRAEVDDHRDAIADARARLADAGRSIEIVTLSEVLAHGATLPAAQPFTSP
DDDPLLLLIYTSGSTGAPKGAMYPERLITNAWRRSGRSAWGGEQTTPSITLNFMPMSHMMGRGVLYGTLGAGGTAYFAARSDLSTFLDDLALVRPTQLSFVPRIWDTIAAEVAKEVDRR
PDDLADVYADLRQSLLGGRHVMAMSGSAPLSPEMRTFVEDLIDIHLTDGYGSTEAGAVFVDGQVQRPPVIDYKLVDVPDLGYFTTDRPHPRGELLVKSETLFPGYYKRPEVTAEMFDPD
GYYRTGDVVAETGPDQLVYLDRRNNVQKLSQGEFVTVSKLEAVFGDSPRVRQIYVYGNSARSYLLAVVVPTEDVLGRDDAKALVAESLQNVARAAGLQSYEIPRDFLIEPTPFTLENGL
LTGIRKLARPRLKEHYGEQLEALYAELADGQADEMRTLRADGANRPMLETVGRAAAALLGTAATDVQPDAHFTDLGGDSLSALTFGNLLRDIFDVEVPVGVIVSPATDLASLAAYIEAQ
RQPGAKRPTFTAVHGAGATEARASDLTLDKFIDAETLSAAPSLPGPNTEVRTVLLTGATGFLGRYLALDWLERMDLVDGKVICLVRAKDDDAARARLDATFDSGDETLLAHYRELAADH
LEVLAGDKGEADLGLDPQVWQRLADTVDLIVDPAALVNHVLPYSELFGPNAVGTAELIRLALTTRQKPFAYVSTIGVGAGIEPGKFVEDGDIRQISAVRQIDESYANGYGNSKWAGEVL
LREAHDLCGLPVSVFRCDMILADTTYAGQLNLPDMFTRLMFSLVATGVAPESFYQLDADGQRPRAHYDGLPVEFIAEAISTLGAAVKSGFETYHVMNPYDDGIGLDEFVDWLIEAGYPV
HRVGDYATWLARFTAAINALPERKRQASLLPLLHNYQRPEIPIPGSIAPTDRFRTAVQEAKIGPDKDIPHITPAVIVQYVSNLELLGLL

>Mycobacterium_chlorophenolicum|WP_048469799.1
MPTETRDERLARRIADLHATDPEFAAATPDDAISETIDQPGVRLPQIMATVLDGYADRPALGQRAVRFVIDPQTGRTSADLLPRFETITYAELSARVHAVMNTLTDVAPGDRVALLGFT
SVDYTVIDMALALSGAVSVPLQTSAPAATLRPIIAETEPVIIASAVDHLADAVELAREADTVRRVIVFDHRAEVDDHRDAVADARTRLTEGGRAIEVLTLAEVLEHGATLPAAQPFSSP
EQDPLTLLIYTSGSTGAPKGAMYPERLVAGAWLRSGRSTWYGEHATPSITLNFLPMSHMMGRGLLYGTLGAGGTAYFAARSDLSTFLEDLALVRPTQLTFVPRIWDTIFAEVAKELERR
PDGEAEVYADLRQSMLGGRYVMSMTGSAPLSPEMRTFVEAFLDLHLIDGYGSTEAGAVFVDGEVQRPPVIDYKLVDVPDLGYFTTDRPHPRGELLVKSETLFPGYYRRPEITAEMFDED
GFYRTGDVVAETGPDRLVYLDRRNNVLKLSQGEFVTVSKLEAVFGDSPLIRQIYIYGNSSRSYLLAVVVPTEDVLGRDDAKSLIGESLQDVAKAAGLQSYEIPRDFLIETTPFTLENGM
LTGIRKLARPKLKERYGDQLEALYTQLAEGQADELRALRRDGATRPVVETVGRAAGALLGTAATDVQPDAHFTDLGGDSLSALTFGNLLRDIFDVEVPVGVIVSPANDLAALAAYIETQ
RQPGTKRPTFASVHGADATEAHARDLTLDKFIDAETLAAAPSLPGPSSEVRTVLLTGATGFLGRYLALEWLERMDLVDGTVICLVRAKDDDAARARLDATFDTGDEKLLAHYRALAADH
LEVIAGDKGEVDLGLDPAVWQRLADTVDLIVDPAALVNHVLPYSQLFGPNAVGTAELIRMALTTRQKPFAYVSTIGVGAGIEPGRFVEDGDIRQISATRRVDDSYANGYGNSKWAGEVL
LREAHDLCGLPVSVFRCDMILADTTYAGQLNLPDMFTRLVFSLVATGVAPESFYQLGDDGKRPRAHYDGLPVEFIAEAISTLGAAVETGFETYHVMNPYDDGIGLDEFVDWLIEAGYPV
QRIGDYATWLQRFTAAINALPERKRQASLLPLLHNYQHPEFPVRGSIAPTDRFRTAVQEAKIGPDKDIPHVTREVIVKYVTDLELLGLL

>Nocardia_vulneris|WP_036444667.1
MTTETREDRLQRRIAQLYETDSQFADARPSDAVNAAVAQPELRLPAVVKGIFAGYADRPALGQRAVELVTDAAGRTSARLLPRFDTITYRQLGDRVQAVTNAWHNHPVKPGDRVAILGF
TSVDYTTIDTALIELGAVSVPLQTSAPVTTLRPIVTETEPTVIAASIDFLDDAVELVRSGPAPHRLVVFDYQPKVDAQRETFEAAKTALAGTGVIVEPLADVLDRGRSLADAPLYTPGQ
ADPLTMLIYTSGSTGTPKGAMYPESKVANMWQLASKATWDENAILPAITLNFMPMSHVMGRGILIGTLSSGGTAYFAARSDLSTFLDDLALVRPTQLSFVPRIWDMLFQEYQSRLDRSG
SPEDEVLAEVREDLLGGRFVSAMTGSAPISAEMKTWVERLLDMHLLEGYGSTEAGSVFVDGQIQRPPVIDYKLVDVPDLGYFRTDQPHPRGELLVKSEQMFPGYYKRPEITAEMFDEDG
YYRTGDIVAELGPDHVEYLDRRNNVLKLSQGEFVTVSKLEAVFGDSPLVRQIFIYGNSARSYLLAVVVPTDPAVSKQAISDSLQDAARAAGLQSYEVPRDFIVETTPFSLENGLLTGIR
KLARPNLKSHYGERLEQLYAELAEGQANELSELRRSGADAPVLDTVSRAAGALLGAAASDLAADAHFTDLGGDSLSALTFSNLLHEIFDVDVPVGVIVSPATDLAGIAGYIEGQRHGSK
RPTYASVHGRDATEVHAADLTLDKFLDADTLAAAPSLPKAPAEVRTVLLTGATGFLGRYLALEWLERMDLVDGKVIALVRAKNDAEARARLDATFDVGDPKLLAHYQDLAADHLEVIAG
DKGEADLGLDHSTWQRLADDVDLIVDPAALVNHVLPYSQMFDANALGTAELIRIALTTKIKPFVYVSTIGVGWGIKPGEFVEDADIRVISPTRQVDDSYANGYGNSKWAGEVLLREAND
LCGLPVSVFRCDMILADTTYSGQLNLPDMFTRMMLSLVATGIAPGSFYELDSDGNRQRAHYDGLPVEFIAEAISTLGAHVTEGFETYHVMNPYDDGLGLDEFTDWLIEAGYPVHRIDDY
GQWLQRFETALRTLPDKQRQASLLPLLHNYQKPSQPLLGAAAPTDRFRAAVQEAKIGPDKDIPHVSPAVIVKYITNLQMLGLL

>Nocardia_araoensis|WP_039804648.1
MAPETRRERLERRITRLYAEYDDIRNATPRADVAKKVREPGIGLAQIVETVMVGYADRPALGQRATEPRTDETGRTTLTLLPRFDTITYGELWDRVRAVAAAWHGDPLRAGDFVGILGF
TSSDYTTLDLACIHLGLVAVPMQSSAPIAQLSAIVAETEPRVLAATPELLDAAVTCALSDSGPKRLIVFDYHPEDDDQRAAFEAARARLAEAGSPVRVESLDEVLTRGRSLPPAPLFVE
PDADPLALLIYTSGSTGTPKGAMYSRRLVARGWVNRPEVAAINFNYMPMSHVAGRGSLTGVLARGGTAYFAAKSDMSTLFDDLALARPTELFFVPRVCDMVFQRFQSEMDRRDGPGVDR
AALEAEVKAELREHFLGGRMVTAICGSAPVSAEMKAFVESVLDLELHDGYGSTEAGVVVIDERVQRPPVLDYKLADVPELGYFRTDKPHPRGELLLKSTTLISGYYKRPDITAEVFDSD
GFYKSGDIVAELAPDRLVYVDRRNNVLKLSQGEFVAVARLESVYATSPLIRQIFVYGSSERSYLLAVIVPTEDVLAWGDPEKMKTALSESLRQLAKDAELQSYEIPREFLIEPEPFSTA
NGLLSGIGKLLRPKLKERYEPRLQELYAELAAGQANELLDLRHTADDLPVLETVSRAAKAVLGCADADLRPDARFGDLGGDSLSALSLSNLLHEIFEIEVPVSVVISPANGLREVADYI
AAERASGARGATYASVHGAGSQVHAADLTLDKFIDARTLAAAPTLPRPSATPRTVLLTGANGYLGRFLCLEWLRRLHDSGGTLVCLVRGSDADAARKRLDSAFDSGDPGLVREFQDLAA
AHLEVLPGDIGDPNLGLDDRTWQRLAEQVDVIVHPAALVNHVLPYRQLFGPNVVGTAELIRLALTTRLKPFTYLSTVAVATQIDPAVFAEDGDIREISAVRAVNDSYANGYGNSKWAGE
VLLREANDLCGLPVTVFRSDMILADRRYAGQLNVPDIFTRLLLSLLVTGIAPFSFYRTGANGERARAHYDGLPADFTAEAITTLGAATGTGFHSFDVLNPHDDGRSLDEFVDWLIDAGH
RIERIADYDEWVTRFETALRALPEGLRQASVLPLLHAYRRPAPAVRGSALPAEKFRAAVRAAKIGADQDIPQISRDLIEKYVADLHVRGLL

>Nocardia_asteroides|WP_019047132.1
MTVEVDADRLADRIRALYAQDAQIRAATPIPEAHARVTTPGTPLARIVSTVMTAYADRPALGVRRTELVVEAGRATRRLLPEFELLTYGEVWERARALAASWYAEGLAAGEFVATLGFT
GADYTVLDLATIHLGAVAVPLQAGASATQLRSILDETAPRVLAVDTANLAVALDVVLAGAAPRALVVFDHHADDDNDREVLAAARARLRAANSPIVLSTVAEVIDRGRLADPAPLVPAP
QDDPLAMLIYTSGSTGTPKGAMYTDRLVAAGWQPARPVAVLNVNFLPMSHIAARLTLNGVLARGGTAYFTAAADMSTLFEDIALVRPTEIFLVPRVCDMLLHRFRREVDRRADAGVDPE
VLAEEVRGELRERVLGGRLLTVLCGSAPIAPELRRFVESVLRLRLHDGYGSTETGGVIFDTKVMRPPVLDYKLVDVPELGYFSTDKPYPRGELLLKTTTMISGYYRRPEVTAQVFDEDG
FCRTGDVVAELGPDRVAYVDRRNNVLKLSQGEFVTVSRLEAVFAGADLVRQIYVYGSSERAYLLAVIVPTEAALAGPAASLRAELGASLQRAAVAAELEPYEIPRDFLIESVPFSVDNG
LLSGVSKLLRPALKQRYGARLDALYDEVVREQEAELERLRREAPMLPVDEVVARAALAVLGCAAADLRPSARFGELGGDSLAALTYSTLLRDLLGVEVPVNVLLGPDSDLAGIAGYVRR
EREPGARRVDVETVHGVGATEIRASELTLAKFLDPALLDAAVGLEPAADPARTVLITGANGYLGRFLLLAWLERLAPVGGRVICVVRGADVTAARARLDAVFDTDPSLRAHVEALSGAL
EVVAGDIGEPRFGVDDATWRRLAGEVDLIVHSAALVNHVLPYAQLFGPNVVGTAEVIRLALTGRRTPVSYLSTVAVAAQGESFAEDGDVRTMSPVRRLDGSYANGYGNSKWAGEVLLRE
ASDRFGLPVAVFRSDMILAHSEYPGQVNVPDVFTRLLLSLLVTGIAPKSFYRTDSQGRRQRAHYDGLPADFVAAAVTALGAAATTGYRTFDVVNPHDDGISLDVFVDWLIEAGHRIDRI
DDFATWSARFEAALRALPEERRKHTVLPLMHAYRRPGVPVAGSALPADGFRAAVRAAGVGADADIPHLGPELMRKYVGDLGALGLL

>Nocardia_transvalensis|WP_040753929.1
MEITDAQAQLIRRATELIEGDEQVRAALPDEAVAKAVQAPGLGLASVVATIMEGYADRPAAGRRAVEFVADDSGRRHARLLPRYDTITYGELWERVRALMAAWHHDPTRAGDFVAILGF
TGIDYTVVDLACACLGAVSVPLQAGASLAQLTPIAAETEPRVLATDIEQLGAGVDLVLSGDSVRSVVVFDYYAEDDDHRAALESARARLADSPVTVDTLDEVLARGRDLPAAPLHTDGD
EDELSLLIYTSGSTGTPKGAIYPARLLTRMWRSGGDRPMPVLGFSYMPMSHVAGRGSLISALSLGGTVYFAARSDMSTLFDDITLCRPTMVFFVPRVCDMVFQRYRSEVDRRLAAGGDR
EQVEREVKTELREHFLGGRFLLALVGSAPLSPEMRAFMESVLEIGMFDGYGATETGGVLLNNELQRPPVLDYRLEDVPELGYFGTDKPYPRGELLLKSETLVPGYYKRPELNAEIFDAD
GFYRTGDVMAEIGRDRLVYVDRRNNVLKLSQGEFVAVSKLEALYTSSPLVRQIYVYGSSERAYLLAVIVPTDDAVTAPEPAELKAAIGESLQRVARDAELESYEIPRDFLVEPEAFSMA
NGLLSGIGKLLRPKLKQRYGDRLEERYDELSREQQDELTALRTAAADLPVLETVSRAAKALLGCATTDLRPDAHFADLGGDSLSALSLSTLLQEIFAVEVPVGVIVGPATDLRRLAEYI
ETERASGGTRPTAASVHGAGTEIRAADLTLDKFIDAETLAGATALPRAPRSPDTVLLTGANGYLGRFLCLEWLRRLHDSGGTLICVVRGSDAAAARARLDEVFDSGDPELLSHYRELAE

GTLEVLAGDIGEPNFGVAEADWRRLADSVDLIVHPAALVNHVLPYDQLFGPNVVGTAEVIRLALTARLKPVTYLSTVGVADQVNPQVFTEDGDIREISGVRSLGDGYANGYGNSKWAGE
VLLREAHDLCGLPVAVFRSDMILAHSRYGGQFNLPDMFTRLFLSVLATGVAPKSFYEADAEGNRQRAHYDGLPADFTAEAITTLGMRVGSGYETFDVLNPHDDGLGLDEFIDWLIAAGH
PIERIDDYGQWLSRFETALRALPEQQRQHSVLPLLHAYRRPGAPIRGAMLPAKKFQAAVQEARIGAAADIPHLTPALIEKYATDLKLRNLL

>Nocardia_seriolae|WP_033090657.1
MVEDTARAEIHRRIAERVLADEQVRVAMPLPEVSEAARQPGLGLARAVEVLMVGYAERPAIGERAAEIVTGADGRRIRRLLPEYRTITYAELWSRAGAIAAAWQHDPLRAGDFLCVLGF
GSGDFAALEIAAIRQGLVTVPLQANAAAAQWRSIIEETGARTLAVSLELLDSALDVVLDGSPVTSIVVFDFEPEEDRQAEILVGARDRIAASGSTITLESLAAVLERGATLPAVPLHVP
ADEDEVALLIYTSGSTGTPKGAIYPHRLVTGMWLGPNVIPAPVMNFCYMPLSHVAGRMVLSGTFARGGTAYFAASSDMSTLFEDIALVRPTEVFFVPRVCDMLFQRCQSEVQRRTAAGE
SVEDADAAVKTALREEFLGGRLVRVMVGSAPVSAEMKEFMRSVMGQPVIDGYGSTEAGGILIDNEIRRPPVIDYKLADVPELGYFSTDKPHPRGELLVKSTQQIPGYFKRPDVTAEIFD
ADGFYRTGDIVAEVRPDHLVFVDRRNNVLKLSQGEFVAVSKLEAVYATSPLIAQIFVHGSGERSHLLAVIVPTAAARALAPAERTAAIAESLRQIARDAELESYEIPRDFIVEDEPFTQ
ENGLLSGIAKLLRPKLRERYGARLEQMYDEQAQRQRDELATLRREAAELPVLETVCRAARAVLGGTQPPPDAHFTDLGGDSLSALSFSTLLAEIFGVEVPVGVIVSPANALTELAAHIE
AERDSGGSRPTAATIHGGSEIRAADLTLDRFIDAATLAAAPALPLAPQPPRTVLLTGANGYLGRFLCLEWLERLDDTDGTLVCVVRGRDAEAARQRLDEVFDSGDPELTQRYRELAARR
LRVLPGDIGEPNLGLGQADWQELADTVDLIVHPAALVNHVLPYGQLFGPNVVGTAEVIRLALTTVRKPVTYLSTVAVAAQIDPGVFTEDGDIREISAVRAVDDGYANGYGNSKWAGEVV
LREAHELCGLPVAVFRSDMILAHSEFAGQLNLPDMFTRLLLSVLATGLAPKSFYALDSHGNRQRAHYDGLPADFTAAAITVLGAQVREGFETYDVLNPHDDGISLDEFVDWLIDSGHPI
DRIDDYRDWFTRFETALRTLPEHQRSASVQPLLHAYRRPGVPIPGSMLPAKRFHAAVQQAELGPGGDIPHLTRELIDKYVADLKLRGLL

>Nocardia_aobensis|WP_036503986.1
MPDDVQKAERKRRLAAAMADDEMRAAWPDADVSAALARPGLRLAELIDTVMTAYADRPAVGQRSGEIVVDAEGRRVWRLLPRFETLSYRQLWSRAGALASAWQAAGLRAGDFVCTVGFV
SSDYVTVDLAGVRLGTVAVPLQATAAVAQWHSIIAETEARVLACSAELLDAAVEAALASVTIGQLVVFDSADDDAERAAVAAARARLAESGREITLESLHDLVEHGLALPAVPLAGVPG
DDPLSLLVYTSGSTGTPKGAMYTDRLAAAMWLYTGKAPVPAITLNYLPLSHVAGRLQLGGTLARGGTAYFTARSDMSTLFEDLELTRPTELVFVPRVCEMLLQHHQGEVEARVAAGRER
VVVEDEVKTALRERLLGGRFLGAMCASAPLVPEMRAFMESVLGIGLHDGYGSTEAGSVIVDNVVRRPPVLDYKLVDVPELGYFGTDRPHPRGELLLKTTTMFPGYFKRPEITAEMFDAD
GFYRTGDVVAELGPDHLVYVDRRNNVLKLSQGEFVTVAKLESVFSTSALIRQIFVYGSSERAYLLAVIVPSEEALTLPDPRAALAESLQQLAKEAGLDSYEIPRDFLLETEPFTQDDGL
LSGIGKLLRPKLKQRYGERLEQLYAELSREQADELAALRHGAADRPVLETVGRAARALLGCASTDIRPEAHFTDLGGDSLSALSLSNLLTELFGVEVPVGTIVHPANTLRRLAEYIDDE
RHEGGRRPTLATVHGPGTRVRAADLTLDAFIDHETLTAAKSLPPASSPPRTVLLTGANGYLGRFLCLEWLDRLDACDGTLICVIRGRDAEAARARLDEAFDSGDPVLLQRYRELAQRRL
TVLPGDIAEPNFGLDDDRWRELTETVDLVVHSAALVNHVLPYDQLFGPNVVGTAEVVRLAITARRKPVTYLSTVAVASQVDHFTEDGDIRGTSPERVIDGGYANGYGNSKWAGEVLLRE
AHDLCGLPVAVFRSDMILAHSEFAGQFNLPDMFTRLLLSVLATGLAPESFYRPAADGSRARAHYDGLPADFTAAAITALGPGADGGFETFDVLNPHDDGIGLDTFVDWLVDDGHRITRI
TDYADWLGRFETALRALPERQRRHSVLPLLHAFRRPAPAIAGSALPAERFRAAVRAAGTGPDGDIPHLSRELIGKYVRDLTAAGLL

>Segniliparus_rotundus|WP_013139787.1
MGSGADRAKLFFQKIEELTAADPQFAAAVPDQEVVAAVSDPTLSFTRYLDTLMRGYADRPALAHRVGDGYATISYGELWSRVGAIAAAWSADGLEPGDFVATIGFTSPDYTALDLAATR
SGLVSVPLQAGASVAQLSAILEETAPKVFAASAESLEGAVDCVLRTPSVQRLVIFDLRDDSPEHRAALAAAKAKLAQPQNPEQARGPVAVETLDELVARGAALPEPPVFEPAEGEDPLA
LLIYTSGSTGTPKGAMYSQRLVSRFWPRTPVVAQLPSISLHYMPLSHSYGRAVLCGTLAAGGTAHFTAHSDLSTLFEDIALARPTFLALVPRVCEMLLHESRRARDLAELRERVLGERL
LVAVCGSAPLAPETRAFMEELLGFPLLDGYGSTEALSLMRDGVIQRPPVIDYKLVDVPELGYFTTDKPHPRGELLIRSESLVSGYYKRPELTAEMFDEQGYYKTGDVMAEIAPDRLVYV
DRSKNVLKLSQGEFVAVAKLEAAFGASPYVKQIFVYGNSERSFLLAVVVPNAELVGRLDTVQALAEVKPLIADSLAAIAKESGLQSYEVPRDFIVETEPFTTGNGLLSEVGKLLRPKLK
ERYGERLEALYDQIAQGQADELRALREQAGERPVIDTVRKAAAAVVGSSGADFRPDANFADLGGDSLSALGFANLLQDVFGVETPVRIIIGPTASLAGIAEHIERALGGRPGEAAPNSA
SVHGAGAEVIRASDLTLDKFLDAQALEAAQSLPRPTGSHRTVLLTGANGWLGRFLALEQLQRLEATGGKLICLVRGKDAASARARVEEALGTDPALAARFAELAADRLEVVPGDVGEPK
FGLDDRTWDRLAGEVDAVVHSGALVNHVLPYHQLFGSNVVGVAEIIRFAVASKLKPVAYLSTVAVAAGADPAAFDEDGDIREVVPQRPVDDSYANGYGNSKWAGEVLLREAHERTGLPV
RVFRSDMILAHRQHTGQLNATDQFTRLILSLLATGLAPKSFYQLDPQGRRQRAHYDGIPVDFTAEAIVALAAEGNNGHRSYNVFNPHHDGVGLDEFVDWLIEAGHPITRIEDHATWFAR
FTTALRALPEKQRQLSLLPLAQVYSFPHPAVDGSPFRNAVFRADVQRARIGKDHDIPHLTRELILKYAADLAALGLL

>Nocardia_gamkensis|WP_062972233.1
MRRITRLIAENDDIRNATPRAEIAEKLREPGAGLAQMVETVMVGYADRPALGTRATELRTGESGRTTLSLLPEFDTITYGELWERVRAVAAAWHGDTREPLRAGDFVGILGFTSSDYTT
LDLACIHLGLVAVPLQSNAPVAQLSAIVAETAPRVLAATPELLDAAVTCALSQAGPQRLIVFDYHPGDDDQRSAFEAARARLAEAGSSVLVEPLDEVLARGRSLPPAPLFSAEQGVDPL
ALLIYTSGSTGTPKGAMYSQRLVARGWVNRRDVAAINLNYMPMSHVAGRGSLTGVLARGGTAYFTAESDMSTLFDDIALARPTELFFVPRVCDMIFQRFRGEVDRRDGPGVDRAALEAQ
VKTALREHFLGGRMVMAICGSAPVSAEMKSFVESVLELELHDGYGSTEAGGVVVIDERVQRPPVLDYKLADVPELGYFRTDKPHPRGELLLKSTTLISGYYKRPELTAEIFDEEGFYKT
GDIVAELAPDRLVYVDRRNNVLKLSQGEFVAVSHLEAVYATSPLIQQIFVYGSSERAYLLAVIVPTEDVLGWGDLEKMKTALSESLRQLAKDAELQSYEIPRDFLIEPEPFTTANGLLS
GIGKLLRPKLRERYEPRLQQLYADLDAGQANELLDLRRSAVDLPVLQTVGRAAKAVLGCADADLRPDAHFTDLGGDSLSALSLSNLLHEVFDVEVPVSVVISPANNLREVADYIAAVRV
SGGRGATYTTVHGAGPQVRAADLTLDKFLDARTLAAAPSLPRPTETARTVLLTGANGYLGRFLCLEWLRRMHDTAGTLVCLVRGSDAAAARQRLDSAFDTGDPTLVREFRELAAAHLEV
IPGDIGEPNLGLDDRTWDRLADRVDLIVHPAALVNHVLPYRQLFGPNVVGTAELIRLALTTRLKPFTYLSTVAVAAQIDPAVFAEDGDIREISPVRAVDDSYANGYGNSKWAGEVLLRE
ANDLCGLPVAVFRSDMILADRRYAGQLNVPDIFTRLLLSLIVTGIAPFSFYRTDADGNRSRAHYDGLPADFTADAITALGATAGTGFQSFDVLNPHDDGRSLDEFVDWLIAAGHRIERI
ADYREWFTRFETALRALPEWQRQASVLPLLHAYRRPAPAVRGSALPAERFRSAVRAAKIGAEHDIPQISRELIEKYATDLQLRGLL

>Nocardia_mikamii|WP_062999567.1
MPDDVQKAERKRRLAAAMADDEVRAAWPDADVSAALARPGLRLAELIDTVVTAYADRPAVGQRAGEIVVDAEGRRVRRLLPRFETLSYRQLWSRAGALASAWQAAGVRAGDFVCTVGFV
SSDYVTVDLAGVRLGAVAVPLQATAAVAQWHSIIAETEARVLACSAELLDAAVEAALTSATIGQLVVFDSADDDAEREAVAAARTRLAESGRDITIDSLHDLVERGLALPAVPLASAPG
DDPLSLLVYTSGSTGTPKGAMYTDRLAAAMWLYTAKAPVPAITLNYLPLSHVAGRLQLGGTLARGGTAYFTARSDMSTLFEDLELSRPTELVFVPRVCEMLLQHHQGEVEARIAAGGER
AVVEDEVKTELRERLLGGRFLGAMCASAPLAPEMRAFMESVLGIGLHDGYGSTEAGGSVIVDNKVRRPPVLDYKLVDVPELGYFGTDQPHPRGELLLKTTTMFPGYFKRPEITAEMFDE
DGFYRTGDVVAELGPDHLVYVDRRNNVLKLSQGEFVTIAKLESVFSTSALIRQIFVYGSSERAYLLAVIVPSEEALTLPDPRAALAESLQELAKEAGLDSYEIPRDFLLETEPFTQDDG
LLSGIGKLLRPKLKQRYGKRLEQLYAELSQEQADELAALRHGAADRPVLETVGRAARALLGCASTDIRPEAHFTDLGGDSLSALSLSNLLTDLFGVQVPVGTIVHPANTLRRLAEYIDG
ERNEGGRRPTLASVHGPGTRVRAADLTLDAFIDHETLTAAQSLPPASTPPRTVLLTGANGYLGRFLCLEWLDRLDTCDGTLICVIRGRDAEAARARLDEVFDSGDPVLLQRYRELAQRR
LTVLPGDIAEPNFGLDEDRWRELAETVDLVVHPAALVNHVLPYDQLFGPNVVGTAEVVRLAITARRKPVTYLSTVAVAAQVDHFTEDGDIREVSPERVIDGGYANGYGNSKWAGEVLLR
EAHDLCGLPVAVFRSDMILAHSDFAGQFNLPDMFTRLLLSVLATGLAPESFYRPAPGGGRARAHYDGLPADFTAAAITALGPGADSGFETFDVLNPHDDGIGLDTFVDWLVDDGHRITR
IADYADWLARFETALRALPERQRRHSVLPLLHAFRRPAPAVAGSALPAERFRAAVRATRTGPDGGIPHLSRELIGKYVRDLTAAGLL

>Nocardia_concava|WP_051178149.1
MVEDVRGEEVMKRFEALLADEQIRAAMPVPEVAEAARRPGLGLAGIAAVLMEGYADRPAIGERAAEIVVDADGRRTRRLLPAYVTTTYRDLWTRAGHIAASWQHDPLRAGDFLCILGFA
SGDFAALELAAIRQGLVTVPLQSSAAAAQWHSIITETESRTLAVSLELLAPALECVLDGTAVTSIVVFDFEPEDDRQAEAFAAAQRRLEGTGITLESLATVVDRGARLPEAPLHVPADE
NELAVLIYTSGSTGTPKGAMYPHRLAAGMWLGPTVIPAPVMNFCYMPLSHVAGRMVLNGTFSRGGTAYFAASSDMSTLFEDIALVRPTEVFFVPRVCDMVYQRYQSETQRRIAAGESAE
DADRIVKTALREEFLGGRMARVMVGSAPISAEMKEFMRSVLGQPIIDGYGSTEAGGLLIDNEIRRPPVIDYKLADVPELGYFSTDKPYPRGELVVKSMQQIPGYFKRPEVTAEIFDEDG
YYRTGDIVAEIRPDYLVYVDRRNNVLKLSQGEFVTVAKLEAVYATSPLIAQIFVHGSSERSHLVAVIVPTDAARALEPAERIAAITESMQRIAKENGLESYEIPRAFLLEDEPFTQDNG
LLSGIGKLLRPKFKERFGERLEQLYTEQAQRQRDELAVLRREAADRPVIETVCRAARAVLGGGEPQPDAHFTDLGGDSLSALSFSTLLAEIFGVEVPVGVIVSPANNLDRLAAHIEAER
DSGGTRPTVATIHGGKEIRATDLTLDKFLDAATLAAAPALPRASQPPRTVLLTGANGYLGRFLCLEWLQRLDGTDGTLICVIRGRDAETARRRLDEVFDSGDPELLRRYRELAQRRLRV
LPGDIGEPNFGLSQQDWQELADTVDLIVHPAALVNHVLPYGQLFGPNVVGTAEVIRLALTSTLKPVTYLSTVAVAAQIDPAVFTEDGDIREISAVRSVDDGYANGYGNSKWAGEVLLRE
AHDLCGLPVAVFRSDMILAHSDFAGQLNLPDMFTRLLLSVLATGLAPKSFYALDSHGNRRRAHYDGLPADFTAAAITALGARVTEGFETYDVLNPHDDGISLDEFVDWLIESGHAIDRI
DDYRDWFTRFETALRTLPEHQRGASVQPLLHAYRRPGLPIAGSVLPAKRFQAAVQQAKLGPGGDIPHLTRELIDKYVADLKLRGLL

>Nocardia_thailandica|WP_043657970.1
MARDNDAARLSQRIEALYAADDQIRAARPLPEVQERLGAPGLSVNQLITTVMTGYADRPAVGTRRRDGRTLLPDHDLLTYAQLWERAGAIAAAWHADGVRPGDFVVVIGFTSAEYLAID
LAVTHLGAVVVPLTGGVSPGRLRAILDETEPAVLAVDHDNLPAALAATGDGGGPRSLLLFDHREDDDTHAAALAAARTAAADTGIAVRTHDEVRARGEQAPPAPLFPEPGTDPLAMLIY
TSGSTGTPKGAMYTHRLVADGWRAGRPLAAITLNYLPMSHIAARLTLISTLTRGGTAYFASAPDMSTLFDDFALVRPTEVFLVPRVCDIVFQRYRREMDSRGTGVDEEALAERVRTELR
EDLLGGRLMMTICGSAPLAPEMRAFMESVLQLQLHDGYGSTEAGALLVNGYLLRPPVLDYKLVDVPELGYFATDKPYPRGELLLKTSSMVPGYYKRPDVTAEMFDADGFYRTGDVVAEL

GPDRLSYVDRRNNVLKLSQGEFVTVSNLEAAYVASPLIRQIYVHGSSERAYLLAVIVPTERAAALPEARLRPALHEELRAIARAAGLESYEIPREFLLEPEPFTIANGLLSGVAKLLRP
ALKQRYGGRLDQLYADLARGQEDELRALRRDAAVLPVAETVARATRAVLGCAVEDLRPDAHFADLGGDSLAALSYADLLRELLGVDVPVNVVLGPAGDLAGIAAYITRRRAGGARPTPA
SVHGDGAAEIRAADLTLERFLDHETLTAAADLPAAAPEPRTVLITGANGYLGRFLVLEWLSRPGTRVIALVRGADAGAARDRLLAGFGDHVPAALTGADGPEVLAGDISEPDFGLPVQQ
WRRLAAEVDLIVHSGALVNHVLPYAQLFGPNVVGTAEVLRLALTTTRKPVAYLSTVAVAAQGESFAEDGDVRVMSPVRTLDASYANGYGNSKWAGEVLLREAADRFGLPVTVFRSDMIL
AHRTLPGQLNVPDVFTRLLLSVLLTGLAPASFYRTGPDGTRQRAHYDGLPADFVAAAIVALGAHTTGHRTYDVVNPHDDGIGLDTFVDWLIEAGHPIDRIDDHAAWFERFATALRGLPE
QQRRHSLLPVLEVYRHPGRARAGSALPAAGFRDAVRAAGLPGAPDIPHLDPALITKYVTDLRELGLL

>Streptomyces_aureofaciens|KOG75697.1
MYATDPQFRDAAPLDSVTEAIRRPGLPLADLVATVMEGYADRPALGERATEPVTDPDTGRTTLRLLERFDTITYGELWERVGAVASEWRHHPGHAVDRGDFVALLGPTSAEYAMVDLAC
LRCGAVSVPLQAGASAEHLAPIIAQTGPRLLAVDMAHLDVALQLAADAPSLDRIVVLGHRSEITAHQEGLDSARDRLAAQGRGVTVDTLASVIERGRALPPLPRCPEGATPDALSSLIY
TSGSTGTPKGAMYTERLVRQFWVDFVPGQGVRPSIVLNYMPLSHMMGRGVLFGTLAKGGIAYFVASSDLSTLFEDLSLTRPTEFIMVPRISDMLFQRYQAELARRSDTGAEAPGTTADR
AEHVQEDVQAELQAELREELREKTLGGRLLWALSASAPLSAEMTAFVEKCLHVRLLNGYGSTEAGIVSLDGRVVRPPVTDHKLADVPELGYFRTDSPHPRGELLIKSDRLFSGYFQRPD
ATAQVFDEDGFYRTGDIMARTGPDTLVYVDRRSNVLKLSQGEFVATSRLEALFIGSPFVRQVFVYGNSTRAYLLAVIVPTQDALDRSGEDTQRLRSILRESLQRLAAEAGLNAYEIPRD
FLIETEPFSQQNGLLSGVRKLLRPALTKRYGERLEALYTELAERGTDELQALRQAGPSQPVPETVLRAARALLGHRQGDVKPDTHFLELGGDSLSALSFSQLLKEIFHVDVPVDVLINP
VNTLRQVADHIENALAGHQRPTADSVHGPGAKRLLASDLKLGAFLDTGTLTKTGRPAGPLPEARTVLLTGANGYLGRFLCLEWLERVAERGGTLVCVVRGSTDEAARARLDAAFDSGDA
ELLRHYREVAAEHLEVIAGDIGEADLGLDKETWQRLADTVDLIVHPAALVNHVLPYDQQFGPNVLGTAELIRLALTSRVKQFTYLSTVAVVFGAEAAADETADIRTACAVRDLGGGYAD
GYAASKWAGEVLLREAHETYGLPVAVFRSNMILAHRRYRGQLNIPDVFTRLLLSLLATGIAPGSFYAGGAGAHTGSGHYDGLPVDFTARAVAALGDGTREGYRTFNVVNPHEDGISLDT
FVDWLTAAGHPLTRIHDYDAWLDRFETALRGPPDRQRQHSLLPLLHAFTKPEEPLPGSALPAQRFRAAVRAAALDGKADIPHLSQDLITKYVADLRAQHLL

>Streptomyces_griseofuscus|WP_037655238.1
MHPSRIPAGELDARTARRGAHLYATDTQFRDTAPLDTVTAAVRRPGLPLAALVATVMEAYADRPALGERATEPFTDPETGRTTLRLLNRFDTLTYGELWERAGAVAAEWRHQRHHPEHA
VRPGDFVALLGRPGTEYTMVELACVRSGAVSVPLPAGASAEQLAPLVEQTGPRLLAVDTDQLEVALRIAANTPSPPRIVVLGHRPEVTAHQADLAAARDRLAAHGASLDTLASVIDRGR
TLPPLPRVPETSAADALSTLIYTSGSTGTPKGAMYTERVVRQFWVDFVPGQAARPSIVLNYLPLSHMVGRGVLFGTLAKGGLACFAASGDLSTLFEDLSLVRPTEFIMVPRIADLLLRH
YRDELSRRGGTGTDLAEIEARVKEELREEVMGGRLLWAVSASAPLSAETTAFVESCLQVRLLDGYGSTEAGIVLLDGRVLRPPVTDHKLADVPELGYFGTDSPYPRGELLVRSERLVPG
YFRRPDATSEVFDEHGFYRTGDIMARVGPDELRYVDRRANVLKLSQGEFVAVSRLEALFGGSPAVRQIFLYGNSARAYLLAVVVPTRDALDRADGDTRRLRTALRESLRSLAAEAGLNS
YEVPREFLVETEPFSQENGLLSGVRKPLRPALTKRYGERLEALYAELSDREATELEALRRVGADEPVLDTVLRAVRAHLGQEDAAVEPGTRFLELGGDSLTALSFSRTLKEIFQVDVPV
DVVISPVNTLRQVAEHIERALAPDHRRPTADRVHGPDATRLDAADLRLDAFLDARSAARRPAGPPPEARTVLLTGANGYLGRFLCLEWLERLAERDGTLVCLVRGSSADEARARLDAAF
DSGDPELLKRYHEVADRHLHVIPGDIGEPDLGLDGETWRRLADTVDLIVHPAAQVNHLLPYGQLFGPNVLGTAELIRLAITSRIKRFTYLSTVAVVFGDEAAADESADIRTACPTRDLK
GEYADGYAAAKWAGEVLLREAHDAFGLPVAVFRSNLILAHPRYAGQLNVSDVFTRLLLSLLATGIAPGSFYAPGTGAGGGHYDALPVDFTARAVTALGDDARAGFRTYNVVNPHEDGIS
LDTFVDWLAAAGHPLTRVPDHTEWFGRFETALRALPDRLRQHSLLPLLHAFAAPEPPLSGSALPAERFRSAVRAASLGGDNDIPHLSPELITKYVTDLRALRLI

Supplementary Figure 2 - All CAR sequences used in this study to build phylogenetic tree in figure 2. Sequences were retrieved from GenBank by homology search to the *N. iowensis* CAR It must be noted that the majority of CAR homologues identified are autoannotaded as oxidoreductases, and not carboxylic acid reductase.

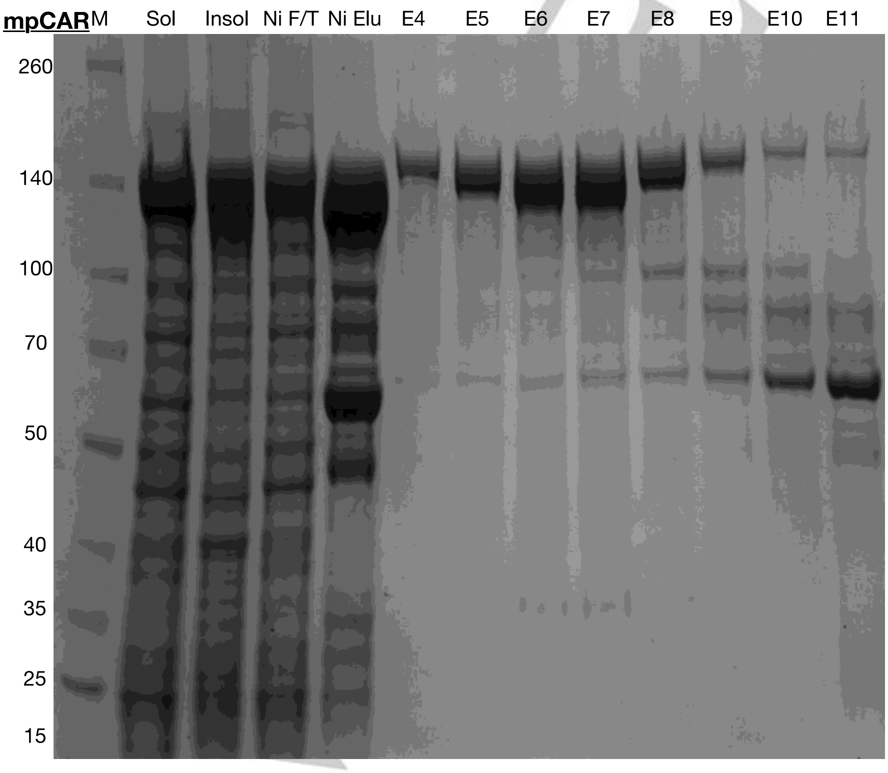

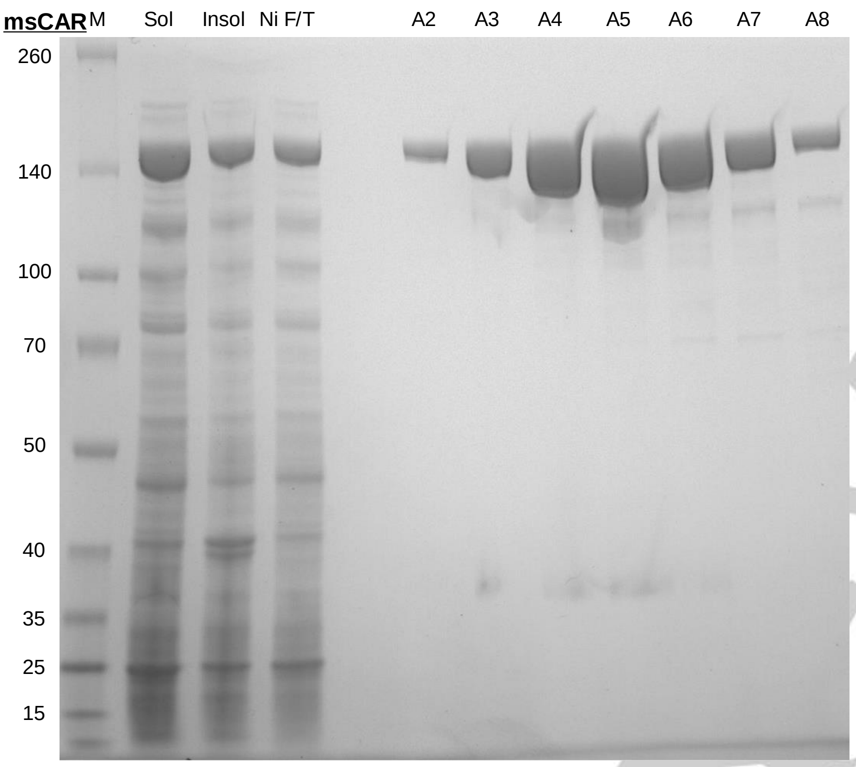
msCAR
M
Sol
Insol
Ni F/T
A2
A3
A4
A5
A6
A7
A8
260
140
100
70
50
40
35
25
15

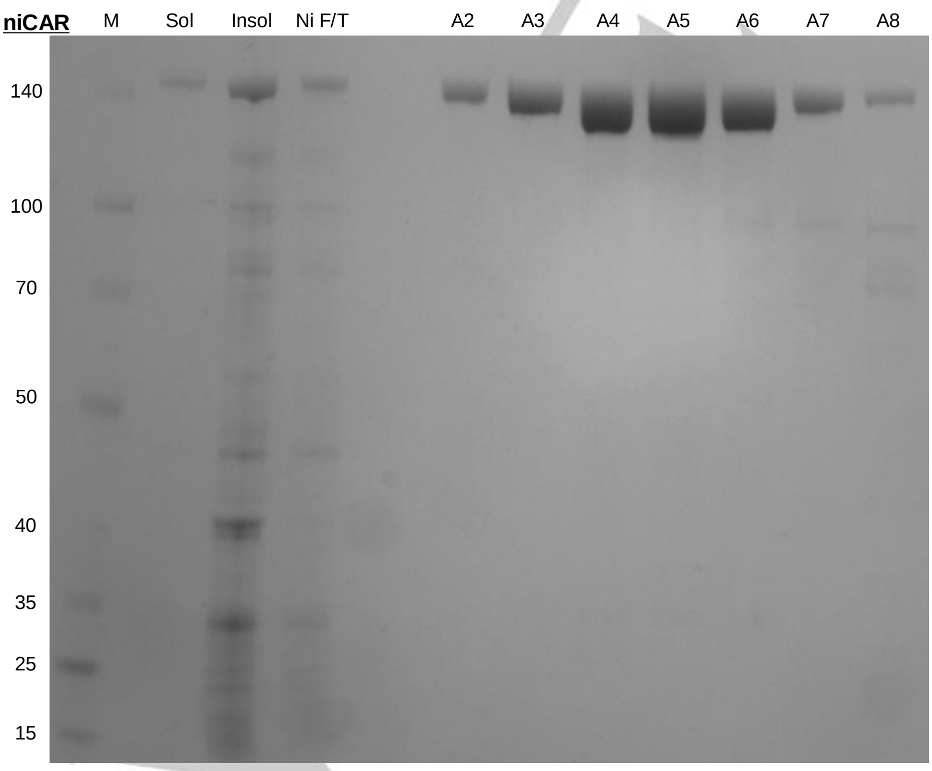
niCAR
M
Sol
Insol
Ni F/T
A2
A3
A4
A5
A6
A7
A8
140
100
70
50
40
35
25
15

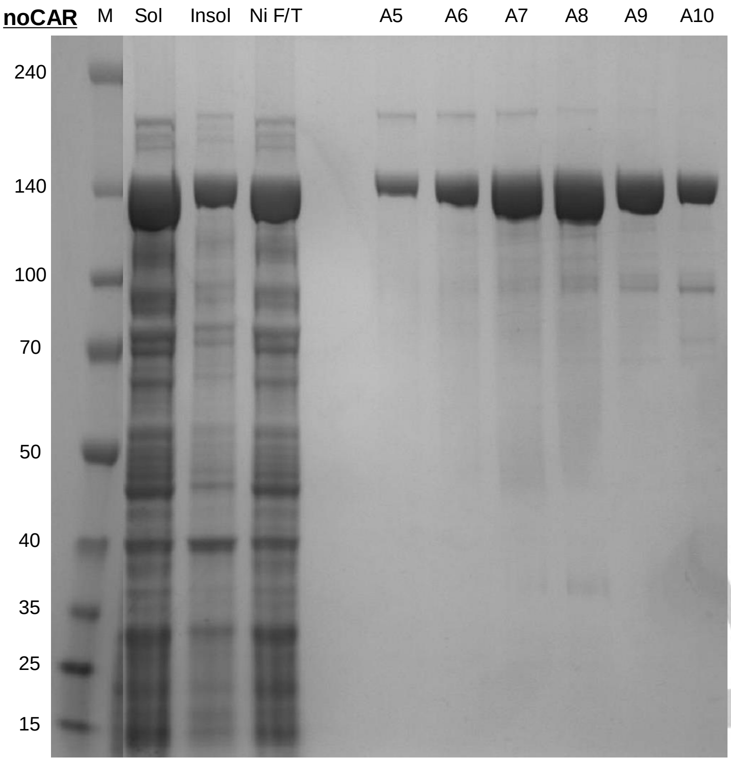
noCAR
M
Sol
Insol
Ni F/T
A5
A6
A7
A8
A9
A10
240
140
100
70
50
40
35
25
15

WILEY-VCH

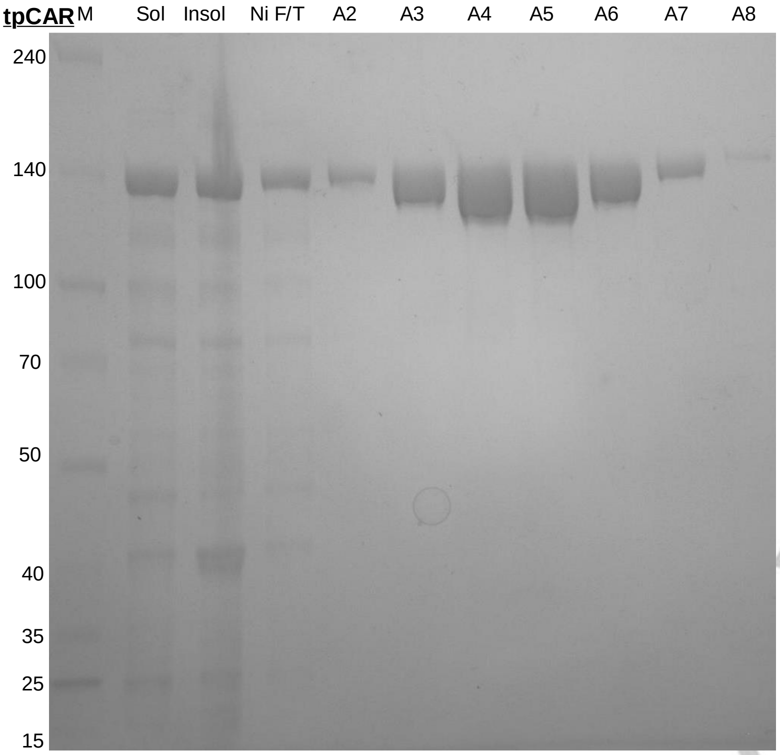

Supplementary Figure 3 – SDS-PAGE analysis of the purification of mpCAR, msCAR, niCAR, noCAR and tpCAR. Each gel shows the soluble fraction of the cell lysate (Sol), the insoluble fraction (Insol), the unbound flow-through from the nickel column (Ni F/T) and the fractions which were collected from the subsequent gel filtration column (eg A2-A8). SDS-PAGE analysis of the gel filtration fractions were used to select fractions for pooling for subsequent assays.

WILEY-VCH

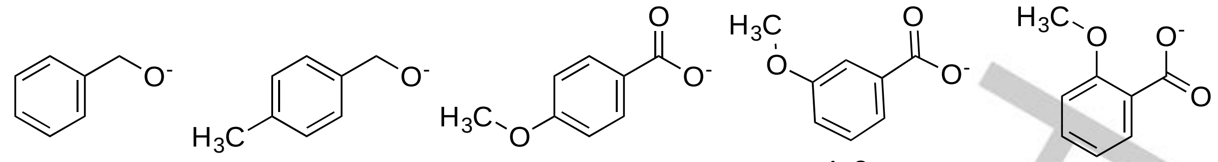

1. Benzoic acid

2. 4-methyl benzoic acid

3. *4*-methoxybenzoic acid

4. 3-methoxybenzoic acid

5. 2-methoxybenzoic acid

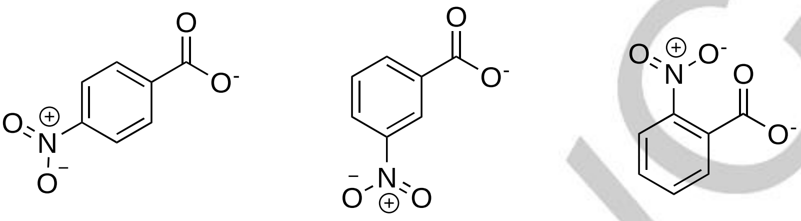

6. 4-nitrobenzoic acid

7. 3-nitrobenzoic acid

8. 2-nitrobenzoic acid

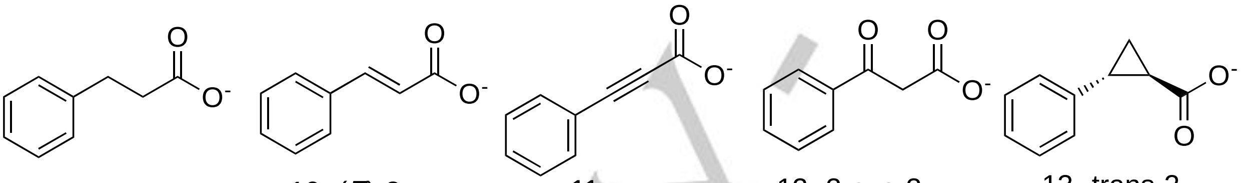

9. 3-phenylpropionic acid

10. (*E*)-3-phenylprop-2-enoic acid

11. Phenylpropiolic acid

12. 3-oxo-3-phenylpropanoic acid

13. trans-2-phenylcyclopropane-1-carboxylic acid

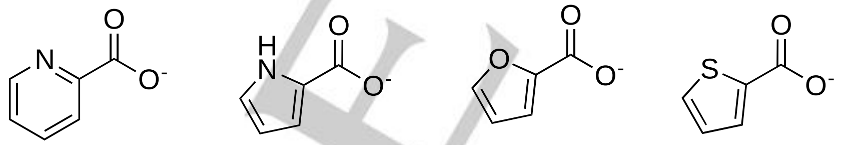

14. Pyridine-2-carboxylic acid

15. 1H-Pyrrole-2-carboxylic acid

16. Furan-2-carboxylic acid

17. Thiophene-2-carboxylic acid

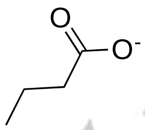

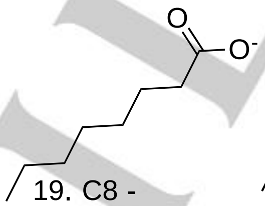

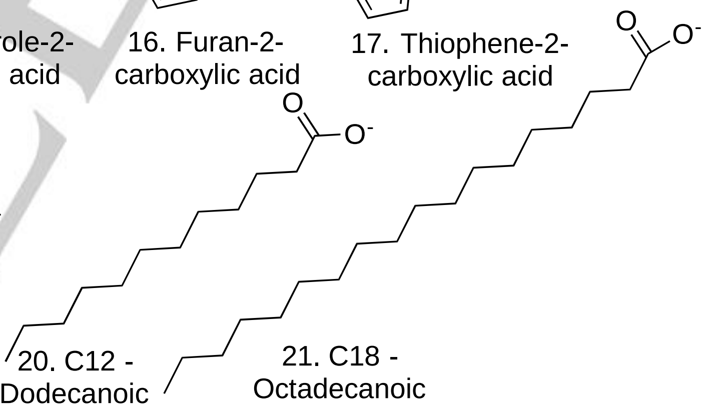

18. C4 - Butanoic acid

19. C8 - Octanoic acid

20. C12 - Dodecanoic acid

21. C18 - Octadecanoic acid

Supplementary Figure 4 – Carboxylic acid substrates - The names and structures of all 20 carboxylic acid substrates tested are shown. The effects of the addition of electron donating or withdrawing groups were tested (2 to 8), extending the carboxylic group away from the benzene ring with various modification (9-13), the inclusion nitrogen, sulphur or oxygen in the benzene ring (14-17) and fatty acids with chain lengths between four and sixteen carbons in length (18-21).

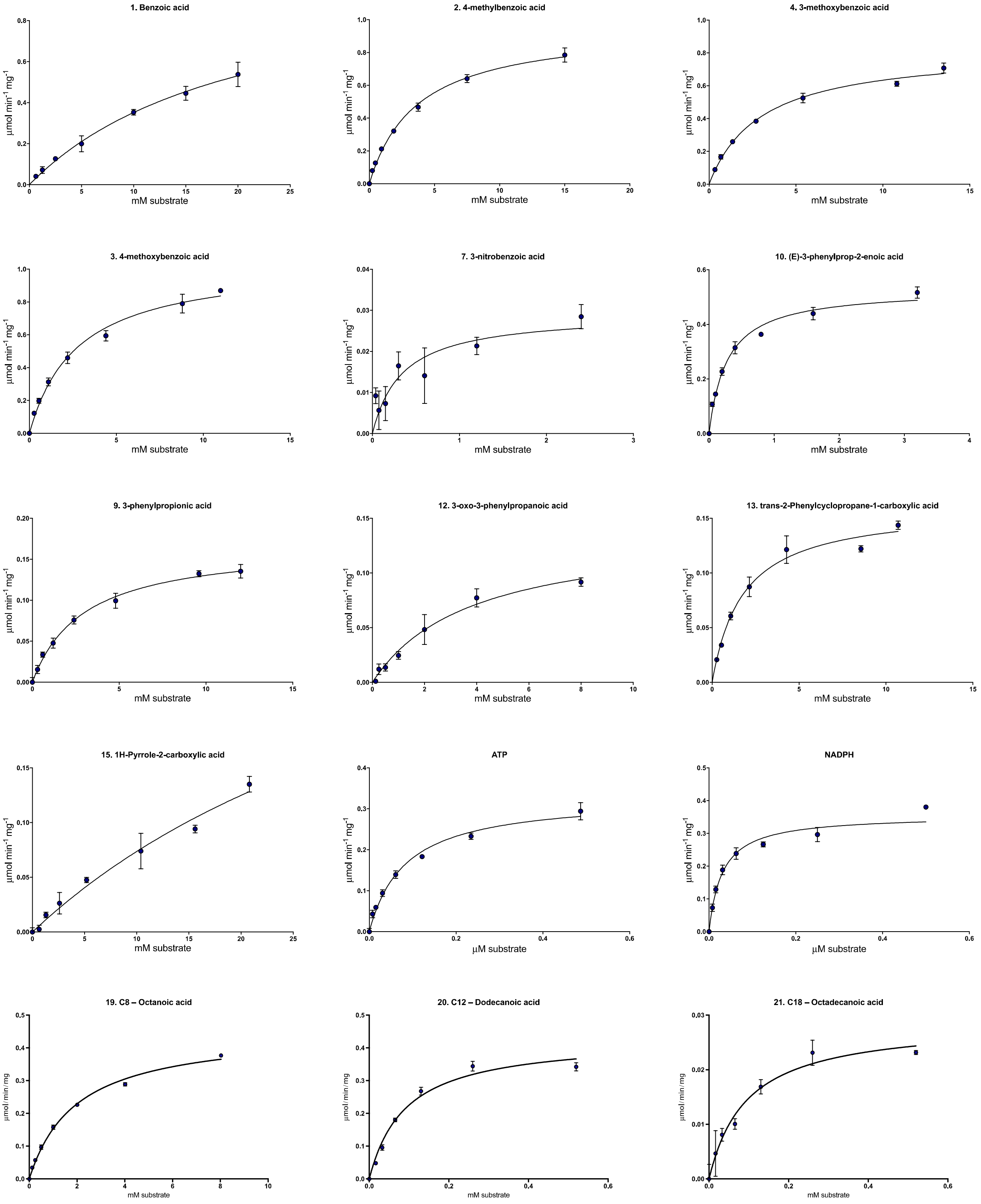

Supplementary Figure 5 – mpCAR kinetic data - Initial rates of activity at changing substrate concentration for each substrate. Appropriate substrate concentrations were calculated from preliminary experiments determining both which substrates the enzyme showed activity against and a rough estimate of the K_M value from initial rates of activity over a wide range of substrate concentrations

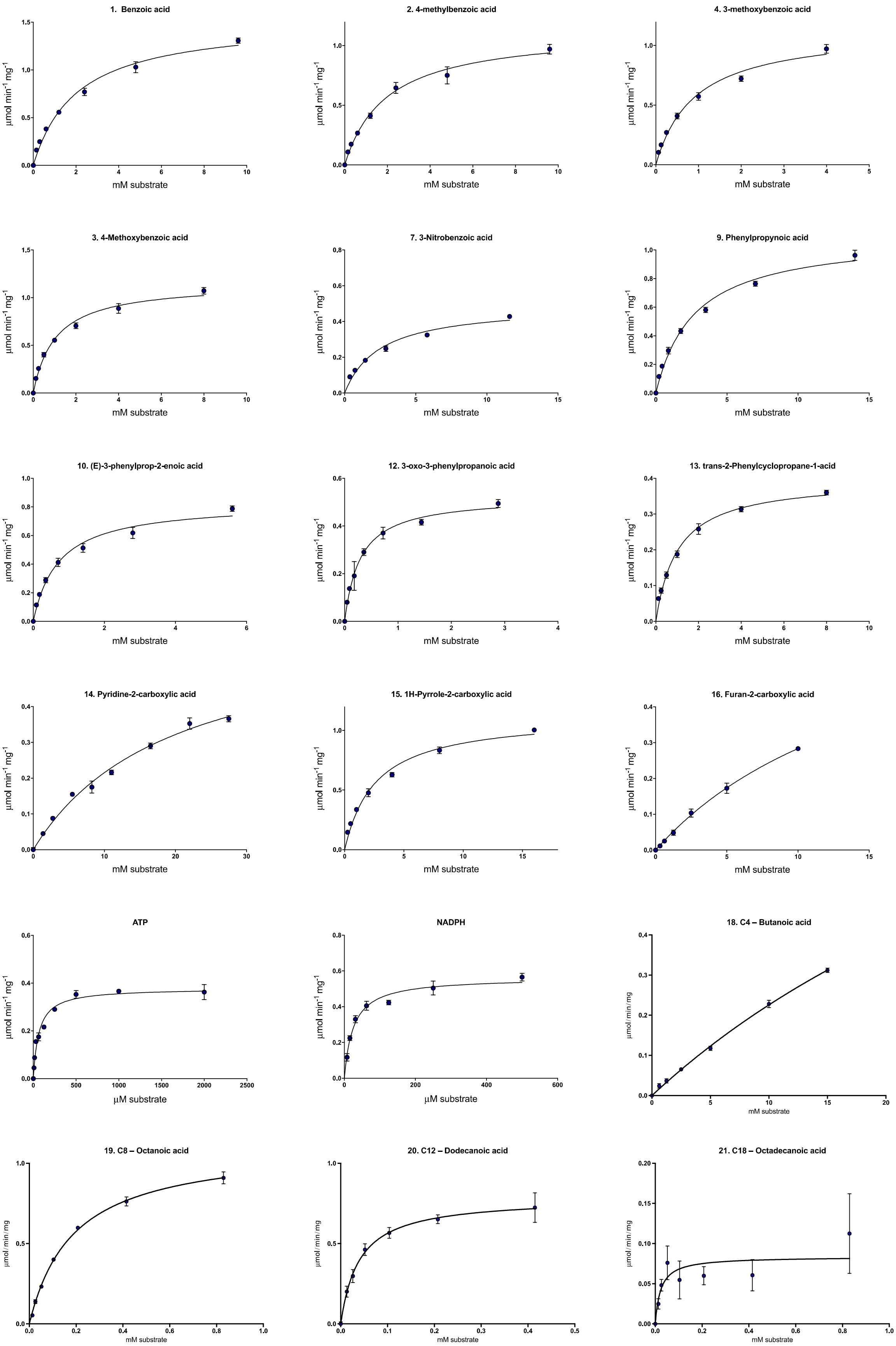
1. Benzoic acid
2. 4-methylbenzoic acid
4. 3-methoxybenzoic acid
3. 4-Methoxybenzoic acid
7. 3-Nitrobenzoic acid
9. Phenylpropynoic acid
10. (E)-3-phenylprop-2-enoic acid
12. 3-oxo-3-phenylpropanoic acid
13. trans-2-Phenylcyclopropane-1-acid
14. Pyridine-2-carboxylic acid
15. 1H-Pyrrole-2-carboxylic acid
16. Furan-2-carboxylic acid
ATP
NADPH
18. C4 – Butanoic acid
19. C8 – Octanoic acid
20. C12 – Dodecanoic acid
21. C18 – Octadecanoic acid
µmol min-1 mg-1
µmol/min/mg
mM substrate
µM substrate

Supplementary Figure 6 – noCAR kinetic data - Initial rates of activity at changing substrate concentration for each substrate. Appropriate substrate concentrations were calculated from preliminary experiments determining both which substrates the enzyme showed activity against and a rough estimate of the K_M value from initial rates of activity over a wide range of substrate concentrations.

WILEY-VCH

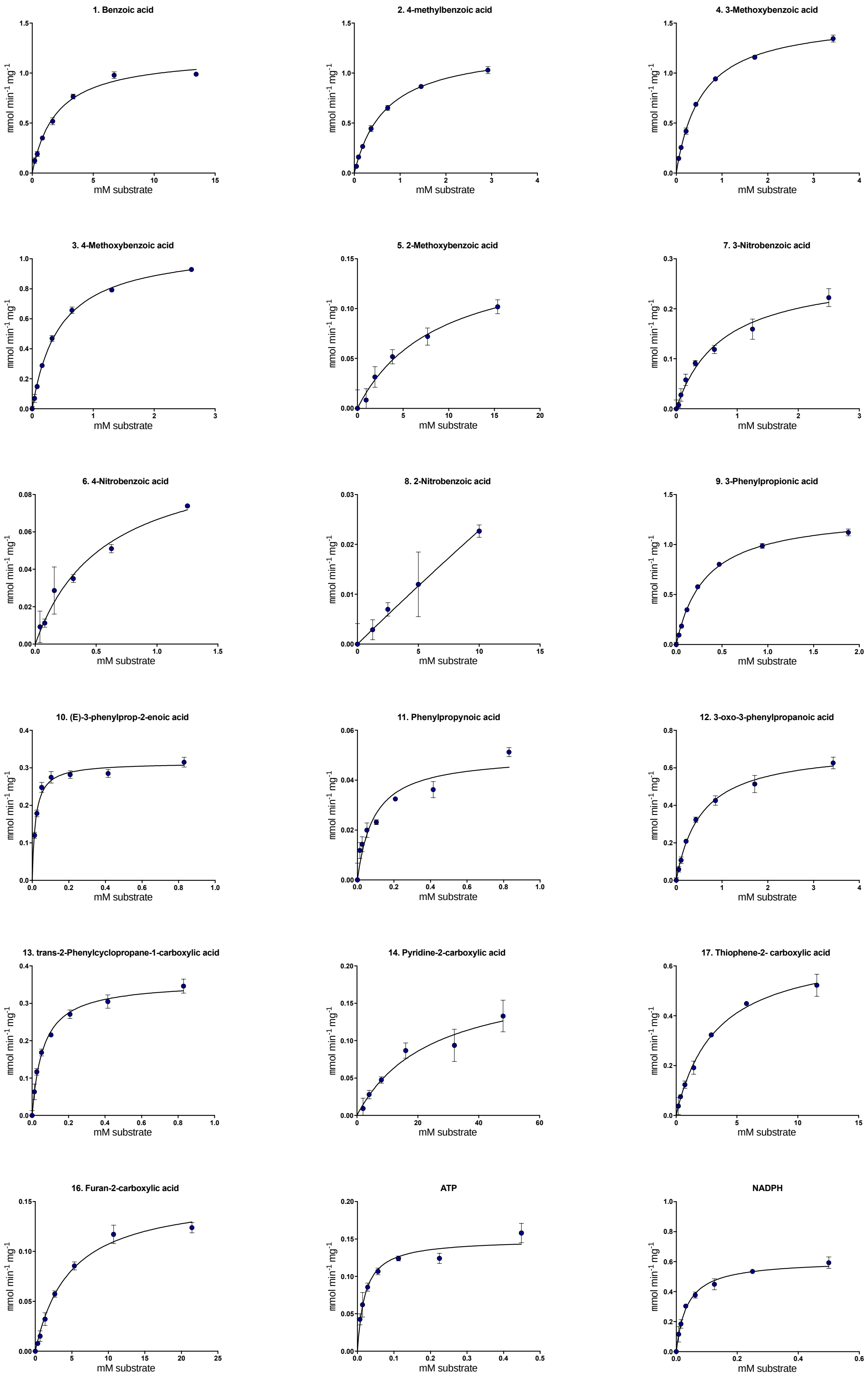
1. Benzoic acid
2. 4-methylbenzoic acid
4. 3-Methoxybenzoic acid
3. 4-Methoxybenzoic acid
5. 2-Methoxybenzoic acid
7. 3-Nitrobenzoic acid
6. 4-Nitrobenzoic acid
8. 2-Nitrobenzoic acid
9. 3-Phenylpropionic acid
10. (E)-3-phenylprop-2-enoic acid
11. Phenylpropynoic acid
12. 3-oxo-3-phenylpropanoic acid
13. trans-2-Phenylcyclopropane-1-carboxylic acid
14. Pyridine-2-carboxylic acid
17. Thiophene-2- carboxylic acid
16. Furan-2-carboxylic acid
ATP
NADPH
mmol min-1 mg-1
mM substrate

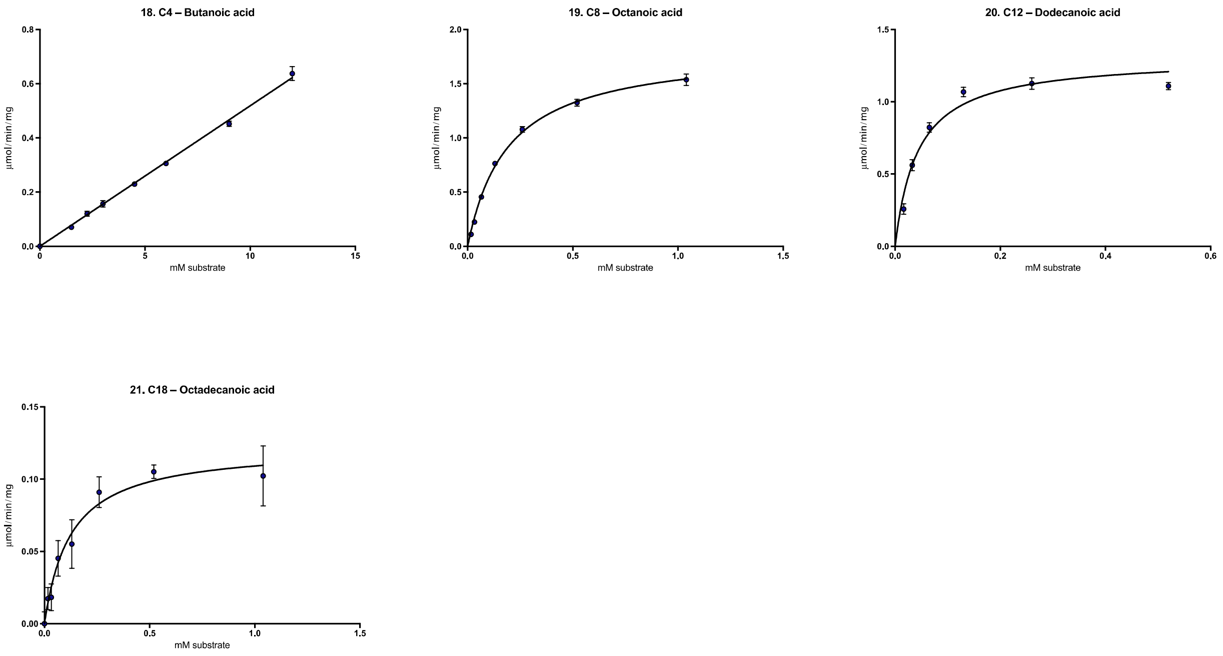

Supplementary Figure 7 – tpCAR kinetic data - Initial rates of activity at changing substrate concentration for each substrate. Appropriate substrate concentrations were calculated from preliminary experiments determining both which substrates the enzyme showed activity against and a rough estimate of the K_M value from initial rates of activity over a wide range of substrate concentrations.

1. Benzoic acid

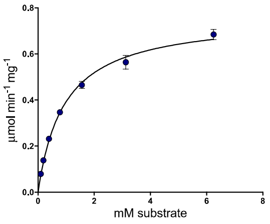

2. 4-methylbenzoic acid

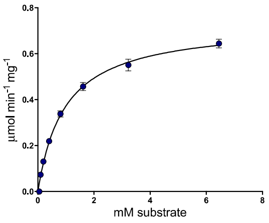

4. 3-Methoxybenzoic acid

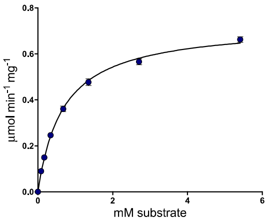

3. 4-Methoxybenzoic acid

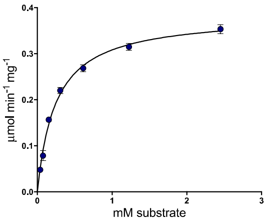

7. 3-Nitrobenzoic acid

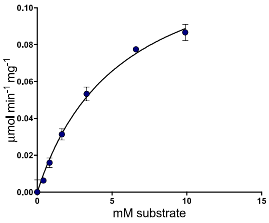

9. 3-Phenylpropionic acid

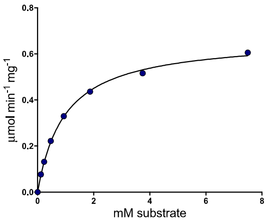

10. (E)-3-phenylprop-2-enoic acid

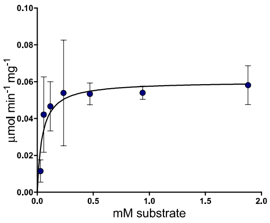

11. Phenylpropynoic acid

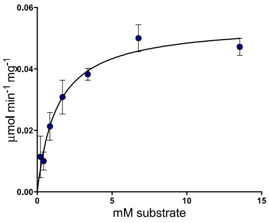

12. 3-oxo-3-phenylpropanoic acid

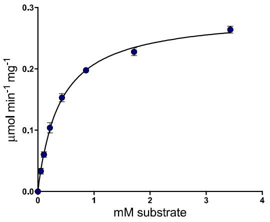

13. trans-2-Phenylcyclopropane-1-carboxylic acid

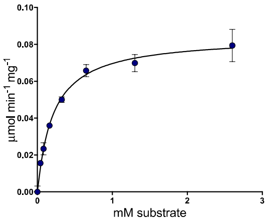

17. Thiophene-2- carboxylic acid

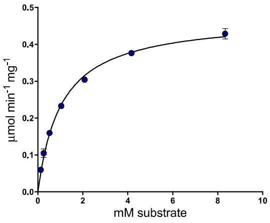

ATP.

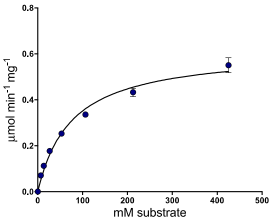

NADPH

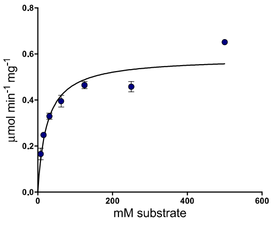

18. C4 – Butanoic acid

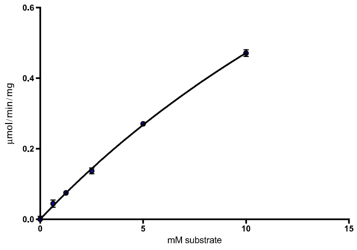

19. C8 – Octanoic acid

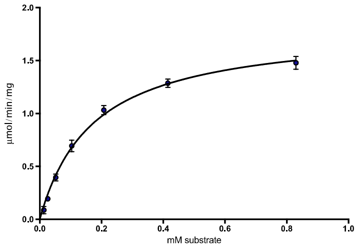

20. C12 – Dodecanoic acid

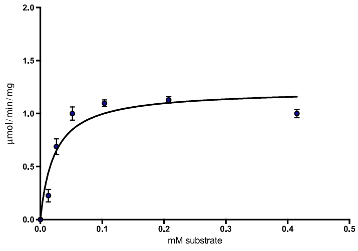

21. C18 – Octadecanoic acid

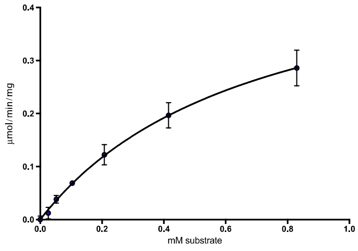

Supplementary Figure 8– niCAR kinetic data - Initial rates of activity at changing substrate concentration for each substrate. Appropriate substrate concentrations were calculated from preliminary experiments determining both which substrates the enzyme showed activity against and a rough estimate of the K_M value from initial rates of activity over a wide range of substrate concentrations.

WILEY-VCH

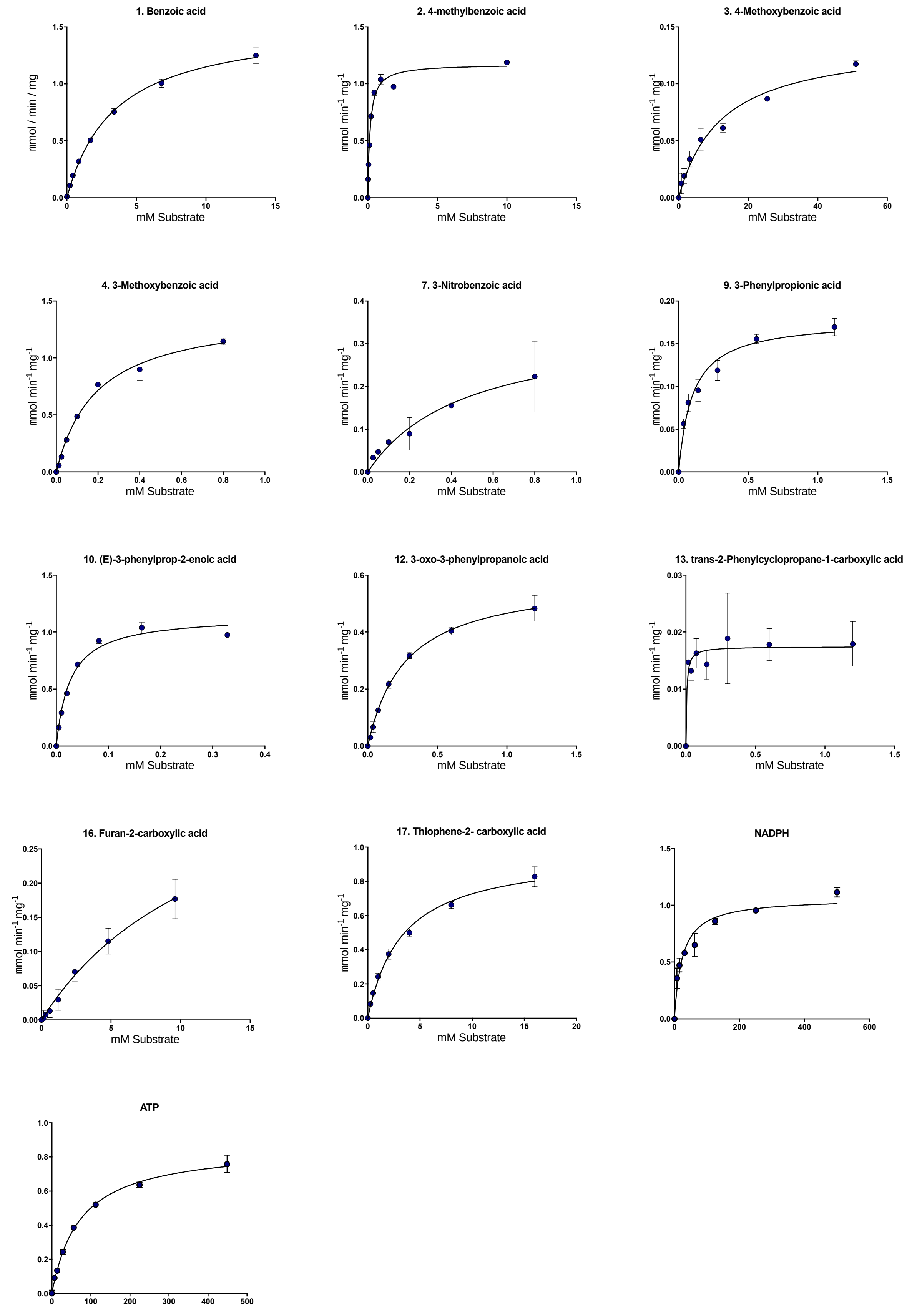
1. Benzoic acid
mmol / min / mg
mM Substrate
2. 4-methylbenzoic acid
mmol min-1 mg-1
mM Substrate
3. 4-Methoxybenzoic acid
mmol min-1 mg-1
mM Substrate
4. 3-Methoxybenzoic acid
mmol min-1 mg-1
mM Substrate
7. 3-Nitrobenzoic acid
mmol min-1 mg-1
mM Substrate
9. 3-Phenylpropionic acid
mmol min-1 mg-1
mM Substrate
10. (E)-3-phenylprop-2-enoic acid
mmol min-1 mg-1
mM Substrate
12. 3-oxo-3-phenylpropanoic acid
mmol min-1 mg-1
mM Substrate
13. trans-2-Phenylcyclopropane-1-carboxylic acid
mmol min-1 mg-1
mM Substrate
16. Furan-2-carboxylic acid
mmol min-1 mg-1
mM Substrate
17. Thiophene-2- carboxylic acid
mmol min-1 mg-1
mM Substrate
NADPH
ATP

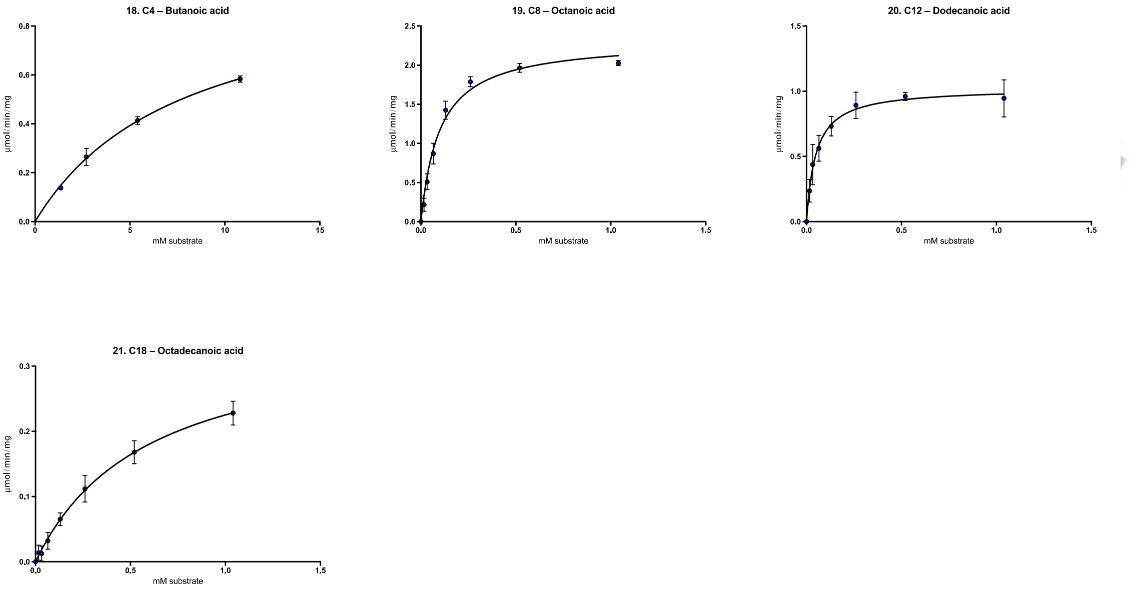

Supplementary Figure 9 – msCAR kinetic data - Initial rates of activity at changing substrate concentration for each substrate. Appropriate substrate concentrations were calculated from preliminary experiments determining both which substrates the enzyme showed activity against and a rough estimate of the K_M value from initial rates of activity over a wide range of substrate concentrations.

WILEY-

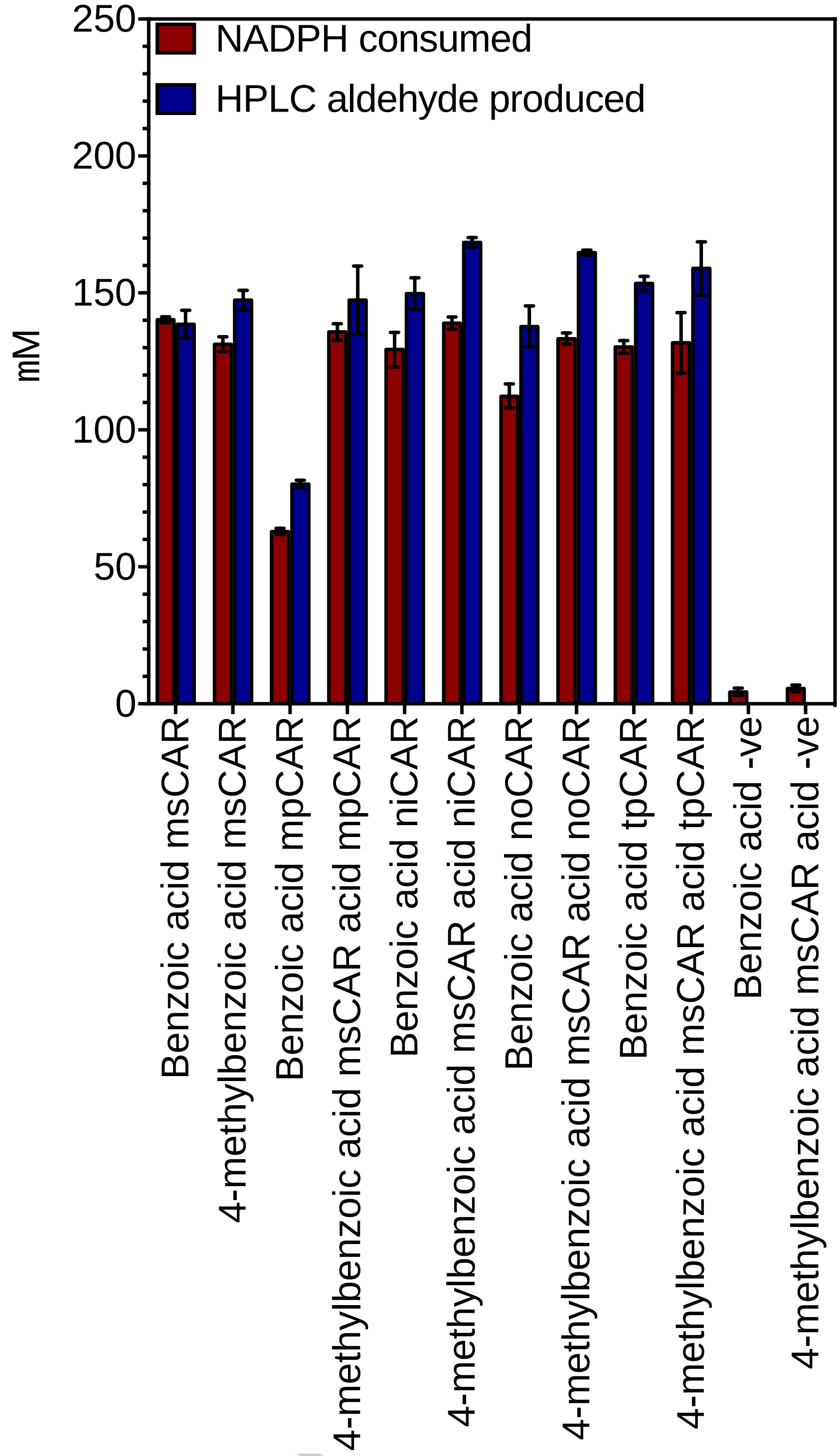

Supplementary Figure 10 – Comparison of NADPH consumption with aldehyde production - Reactions containing 2 mM benzoic acid or 4-methylbenzoic acid were set up and the consumption of NADPH monitored over 10 minutes at OD_{340nm}. Reactions were stopped by the addition of 50 % acetonitrile and aldehyde concentration determined by HPLC. The comparison shows slightly more aldehyde was produced in the reaction than the observed NADPH consumption. This can be attributed to the reaction occurring before the monitoring of NADPH could begin. This comparison shows that measuring NADPH consumption is a good measure of the CAR reaction.

1. Standard - Benzaldehyde

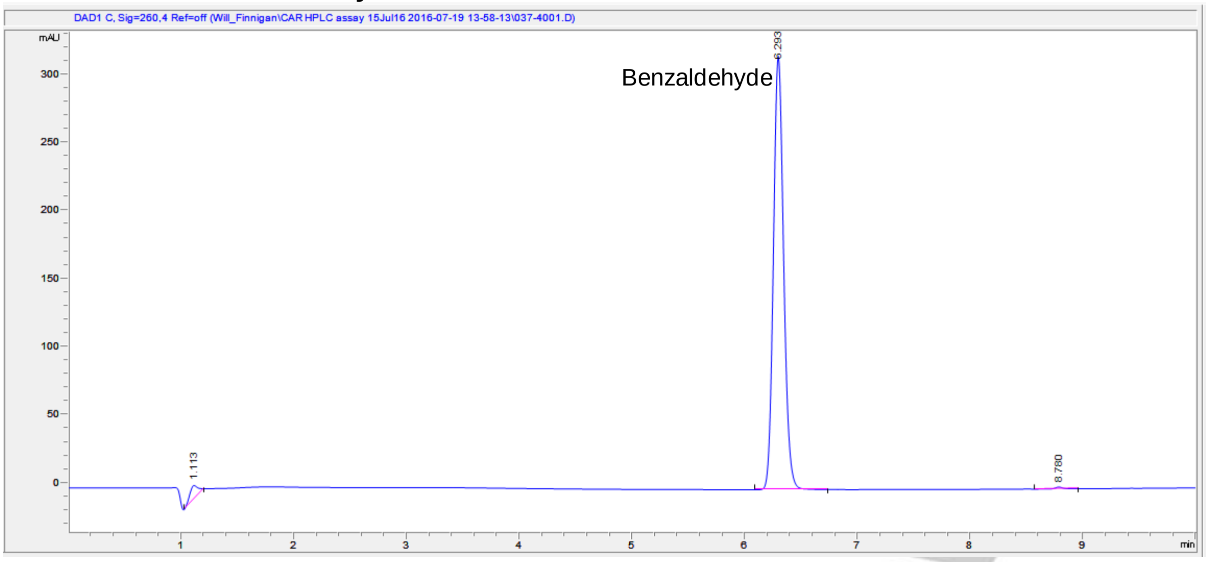

2. Standard - 4-methylbenzoic acid

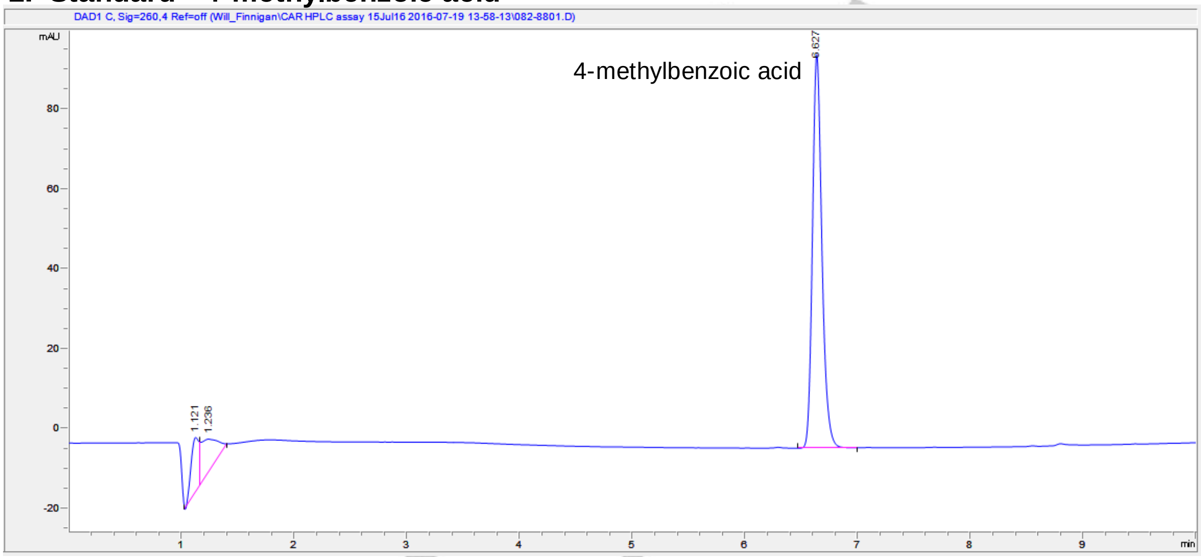

3. Standard - 4-methylbenzaldehyde

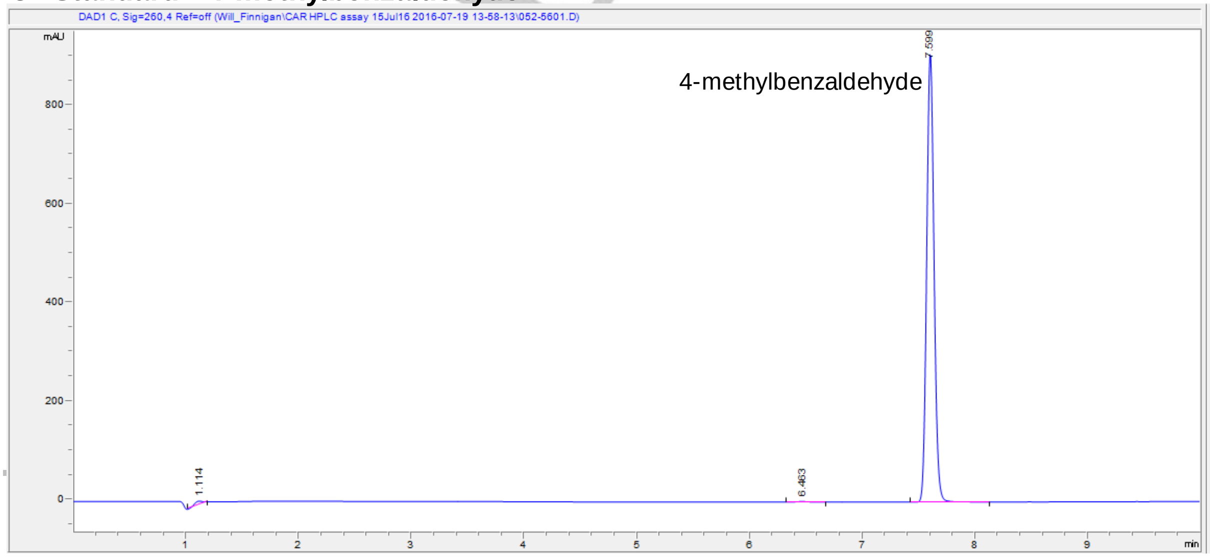

4. msCAR - benzaldehyde

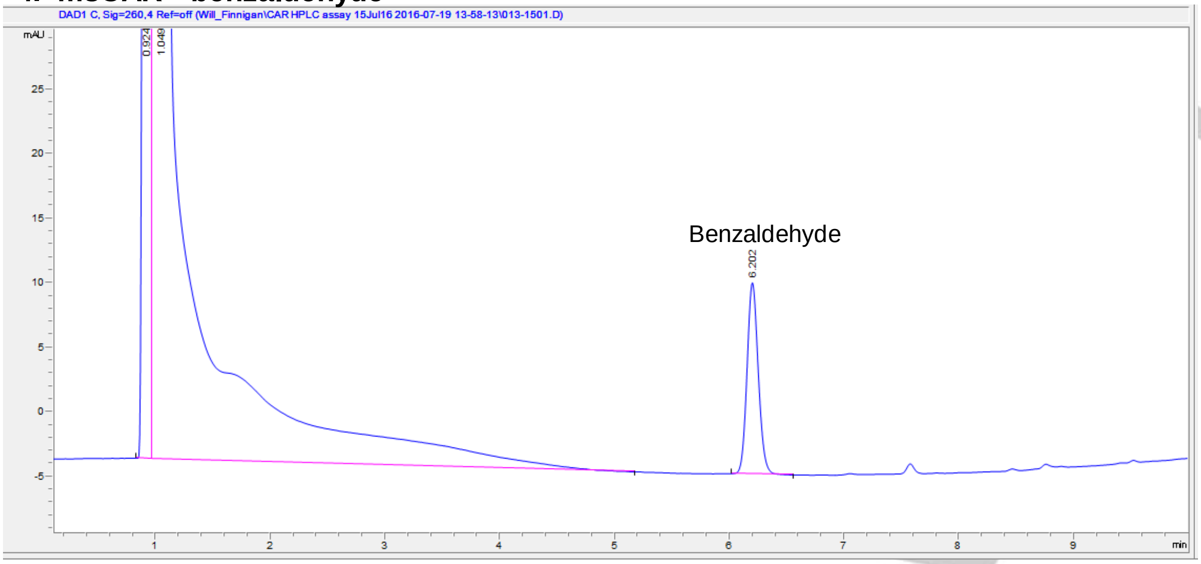

5. msCAR - 4-methylbenzaldehyde

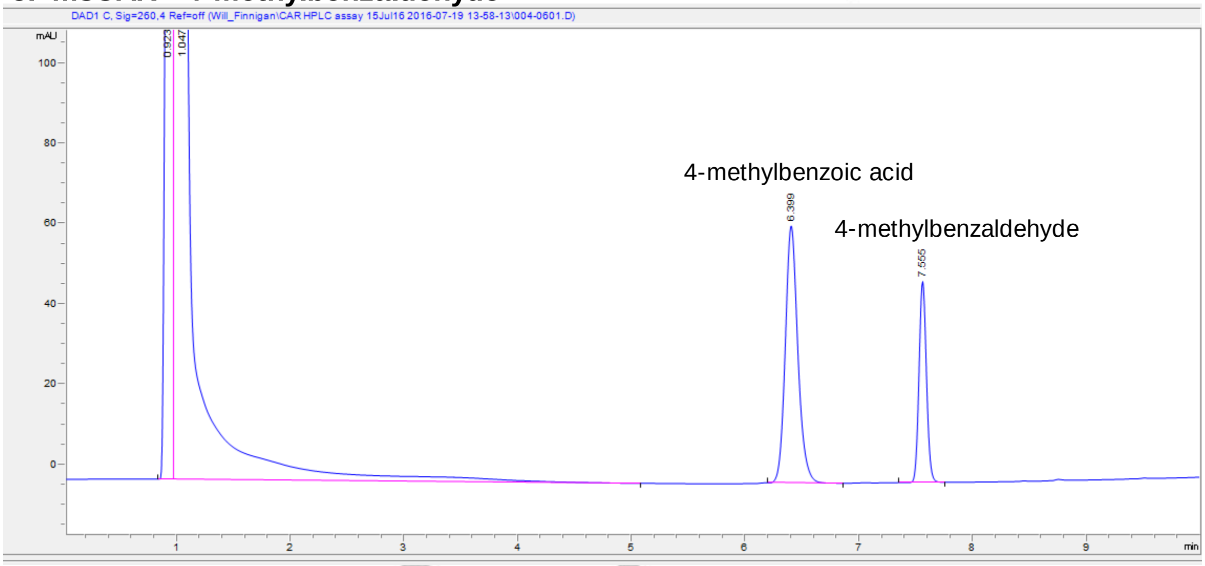

6. mpCAR - benzaldehyde

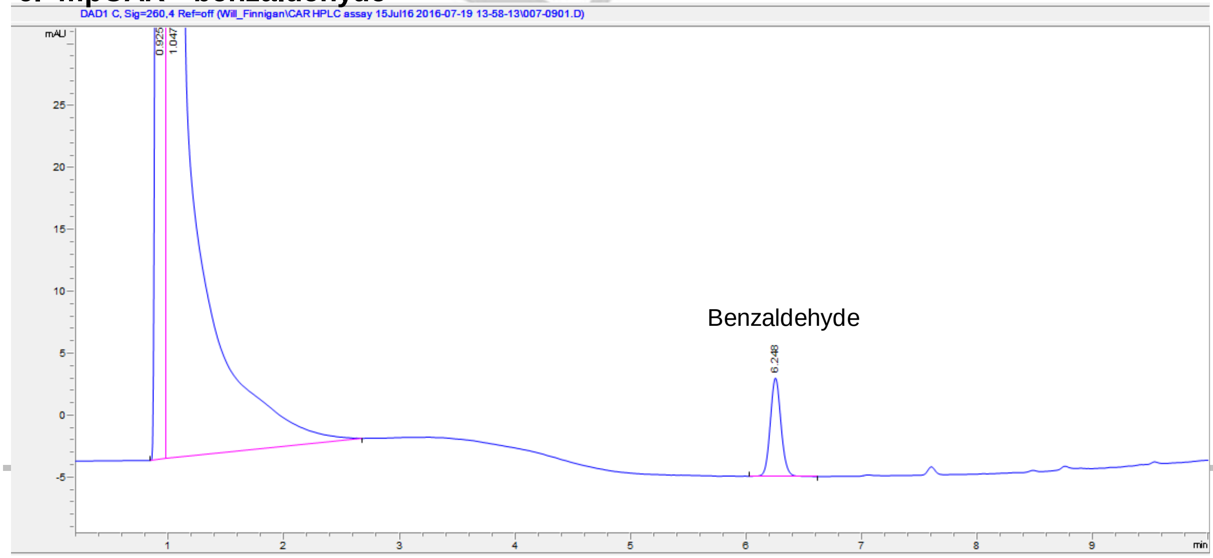

7. mpCAR - 4-methylbenzaldehyde

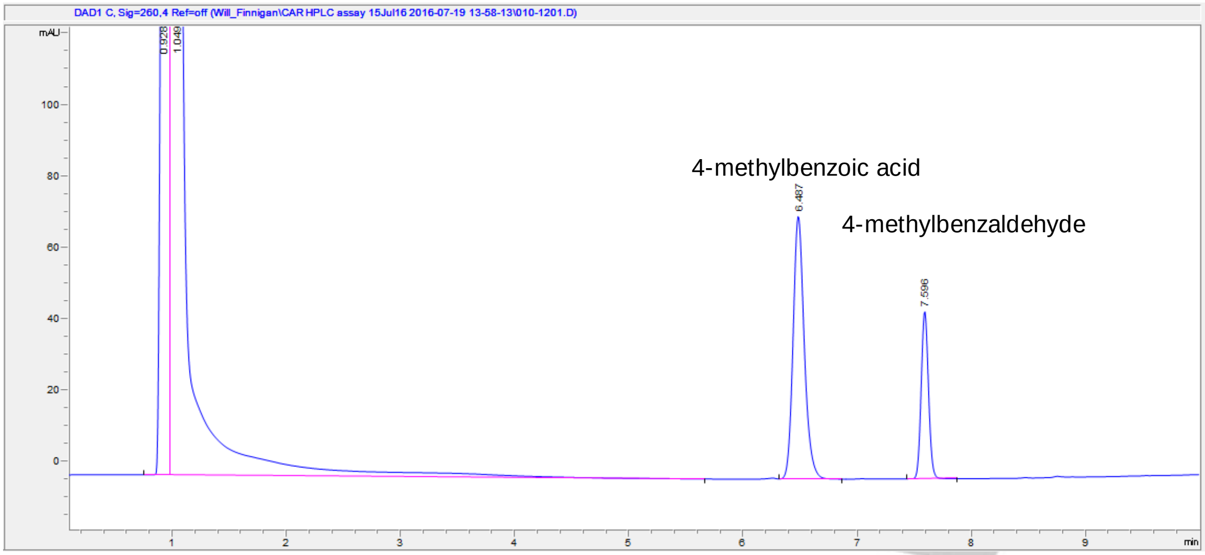

8. niCAR - benzaldehyde

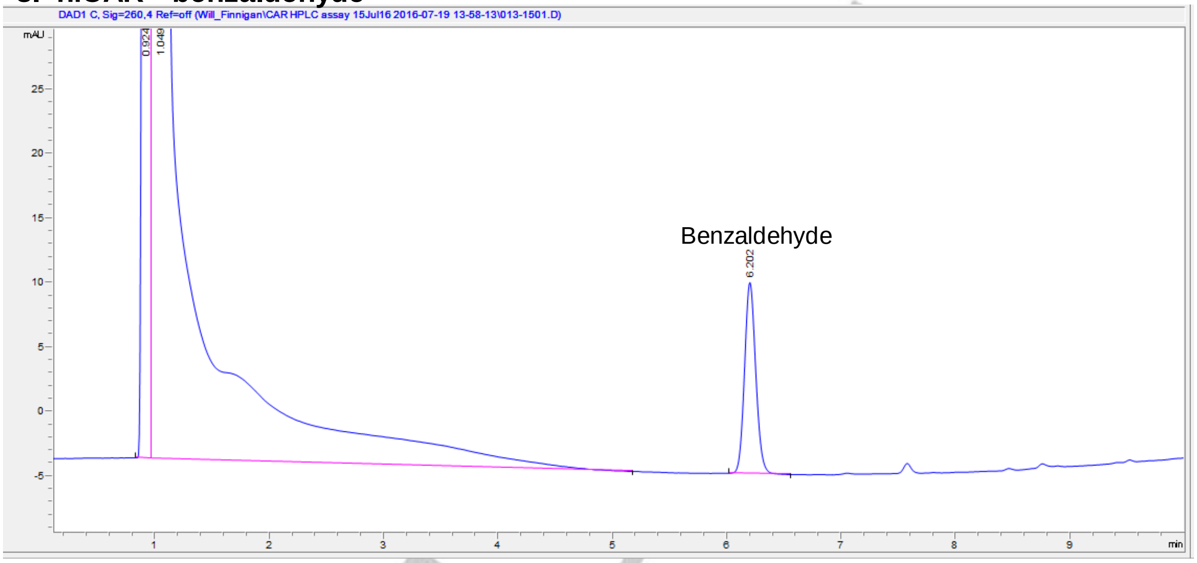

9. niCAR - 4-methylbenzaldehyde

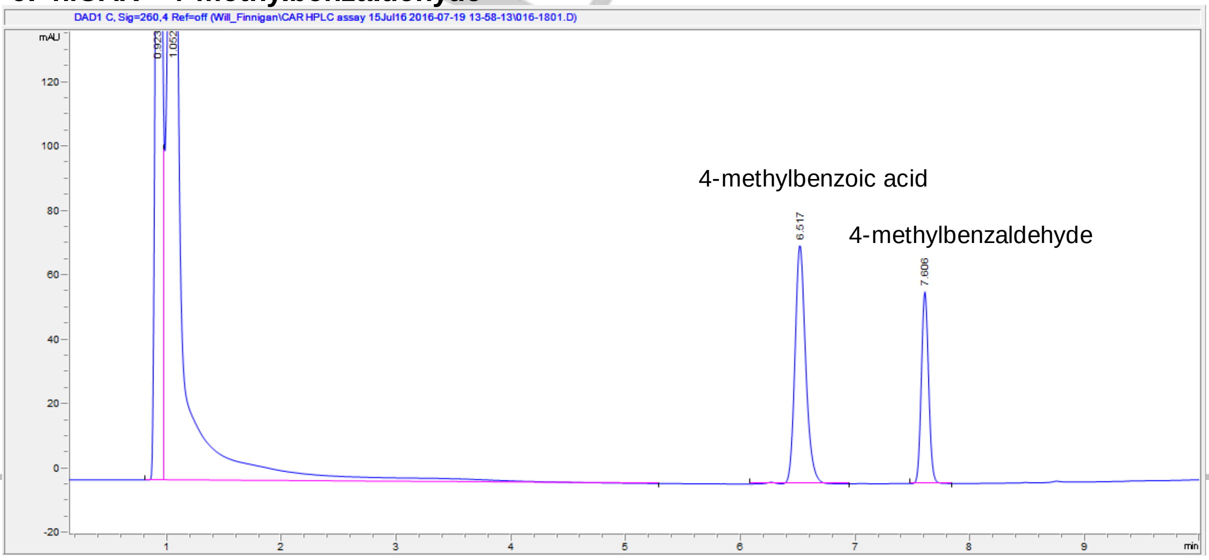

10. noCAR - benzaldehyde

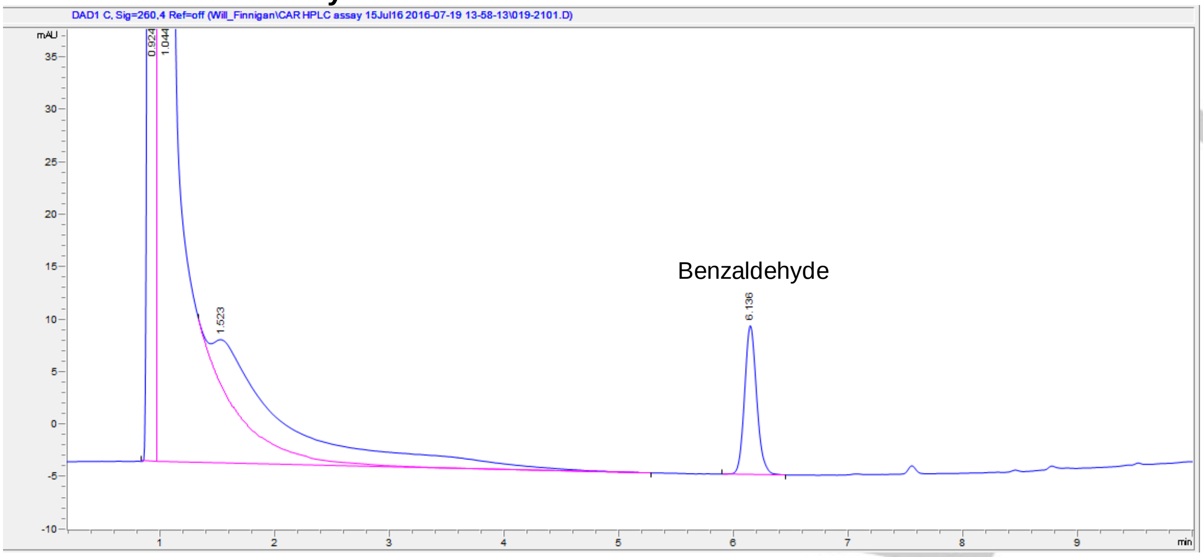

11. noCAR - 4-methylbenzaldehyde

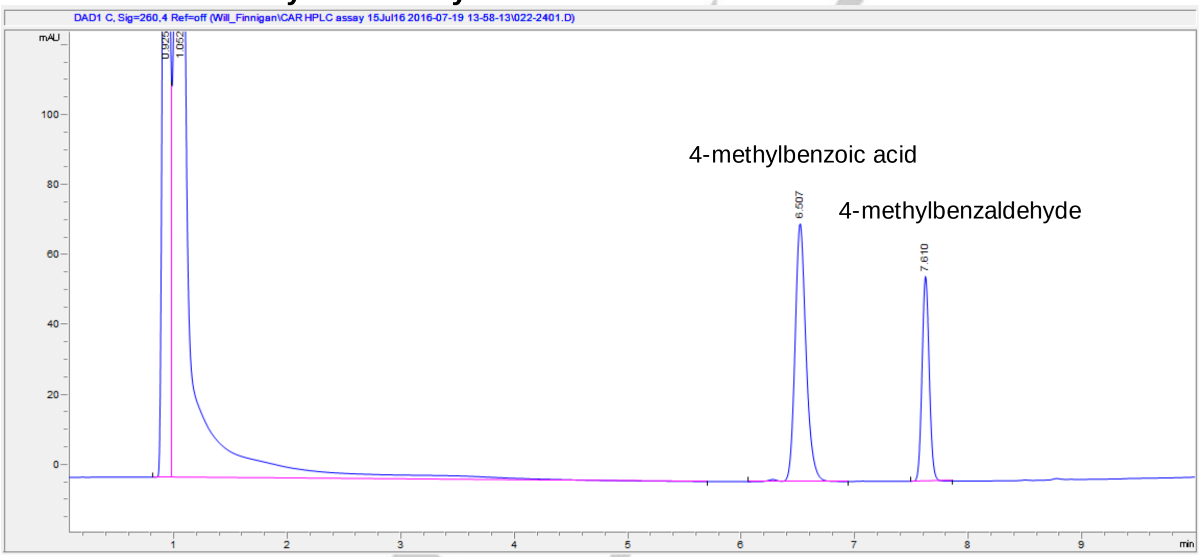

12. tpCAR - benzaldehyde

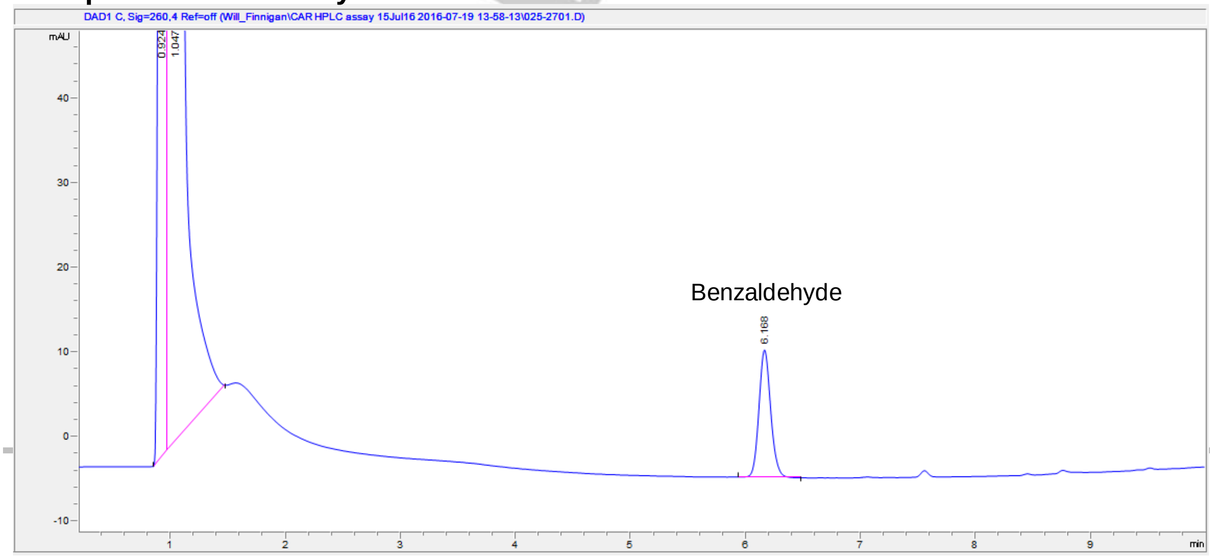

13. tpCAR - 4-methylbenzaldehyde

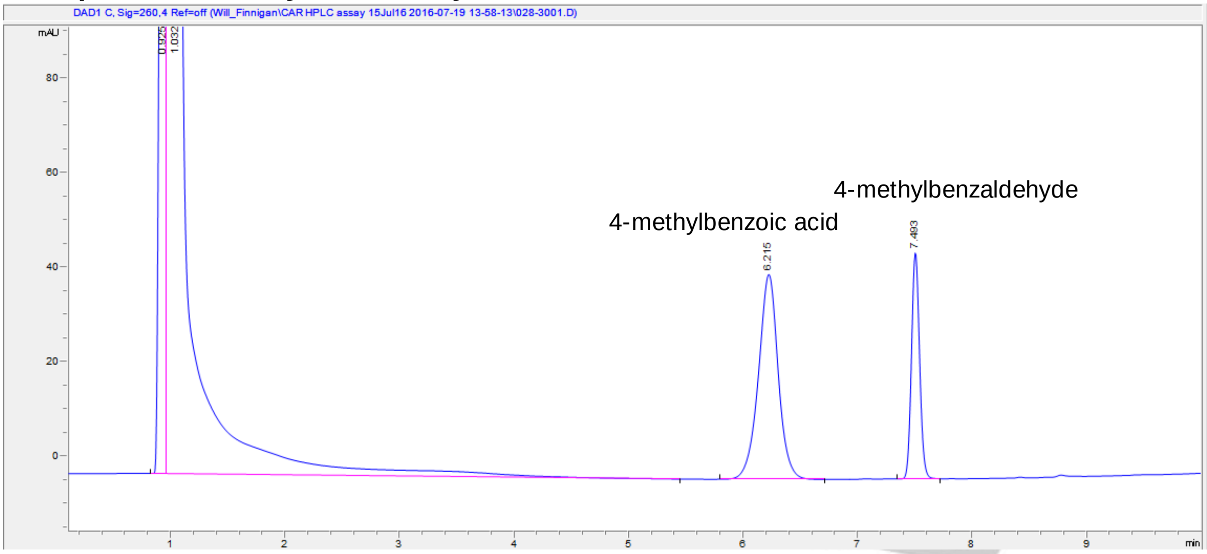

Supplementary Figure 11 – Confirmation of benzaldehyde and 4-methylbenzaldehyde production by all the CARs tested. - 1 mM standards of benzaldehyde, 4-methylbenzoic acid and 4-methylbenzalde are shown in panels 1 to 3. Unfortunately benzoic acid could not be detected using this HPLC method. Panels 4 to 13 show peaks detected following a 10 minute reaction containing 2 mM benzoic acid or 4-methylbenzoic acid, following the standard assay detailed in the main methods section. These reactions were also used to compare of NADPH consumption with aldehyde production shown in supplementary figure 10. Assays were stopped by the addition of 50 % acetonitrile for analysis by HPLC. For all the CARs, the derivative aldehyde product was detected by HPLC confirming it as the product of the CAR reaction. To carry out the HPLC analysis an Eclipse Plus C18 column with a particle size of 3.5 μm, measuring 4.6 x 100 mm, was used. The column was run at 60 ºC on the following method using two buffers, buffer A: 95 % H_2O, 5 % (v/v) acetonitrile, 0.1 % (v/v) trifluoroacetic acid, and buffer B: 5 % H_20, 95 (v/v) % acetonitrile, 0.1 % (v/v) trifluoroacetic acid. 3 μl of sample was injected and eluted on a gradient from 0 to 100 % buffer B over 10 minutes. Buffer B was maintained at 100 % for a further 2 minutes before the column was re-equilibrated with buffer A for 2 minutes before the next run.

WILEY

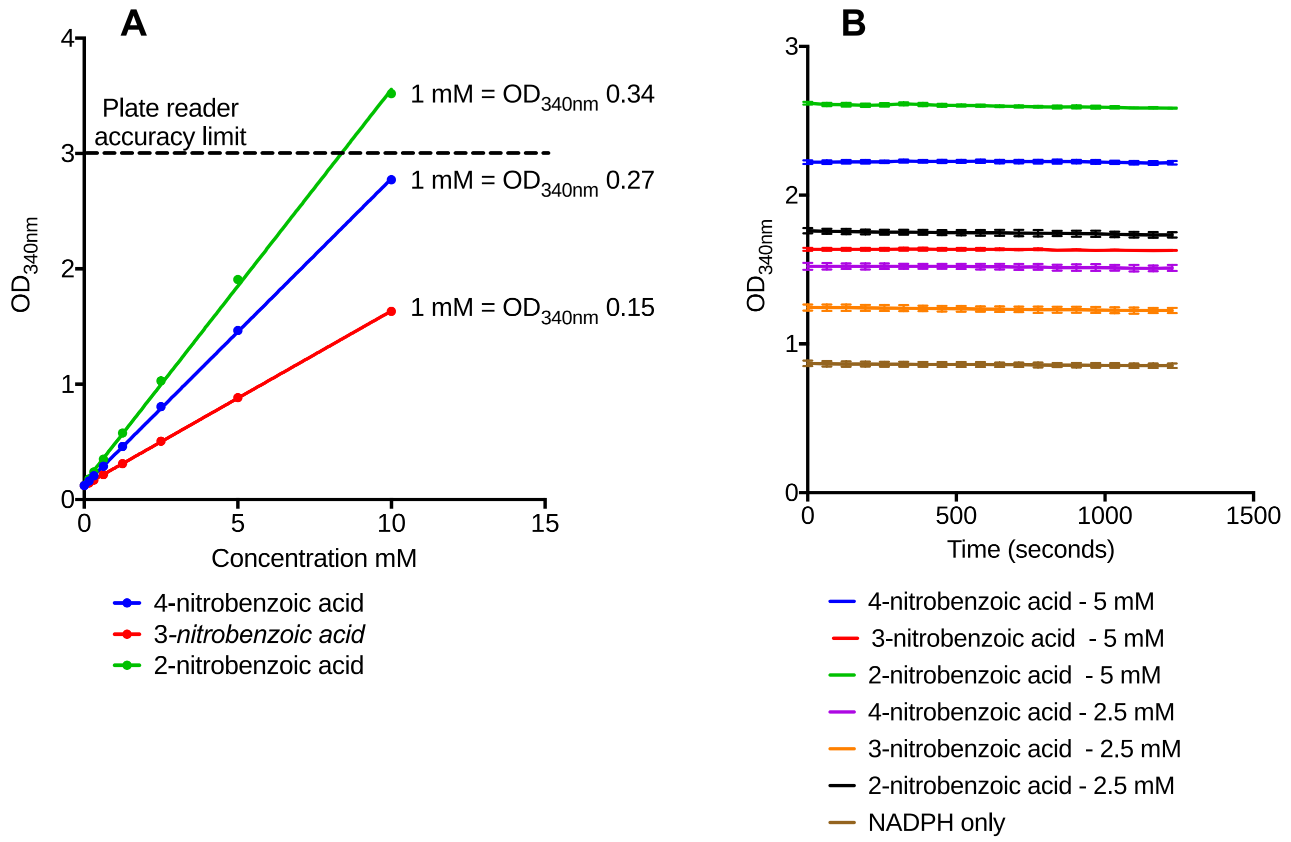

Supplementary Figure 12 – Controls looking at the feasibility of using NADPH consumption to measure activity of CAR enzymes with nitro aromatic acid substrates. - Substrate concentration was titrated from 10 mM and OD_{340nm} plotted as a function of concentration. The plate reader used is accurate up to OD_{340nm} 3.0 as shown. Standard CAR reaction mix without CAR enzyme was assayed with the addition of 5 mM and 2.5 mM of the three nitro aromatic acids used. NADPH can be measured accurately as additional absorbance despite the inclusion of the nitro aromatic compounds. Error bars show the standard deviation of three readings.

WILEY

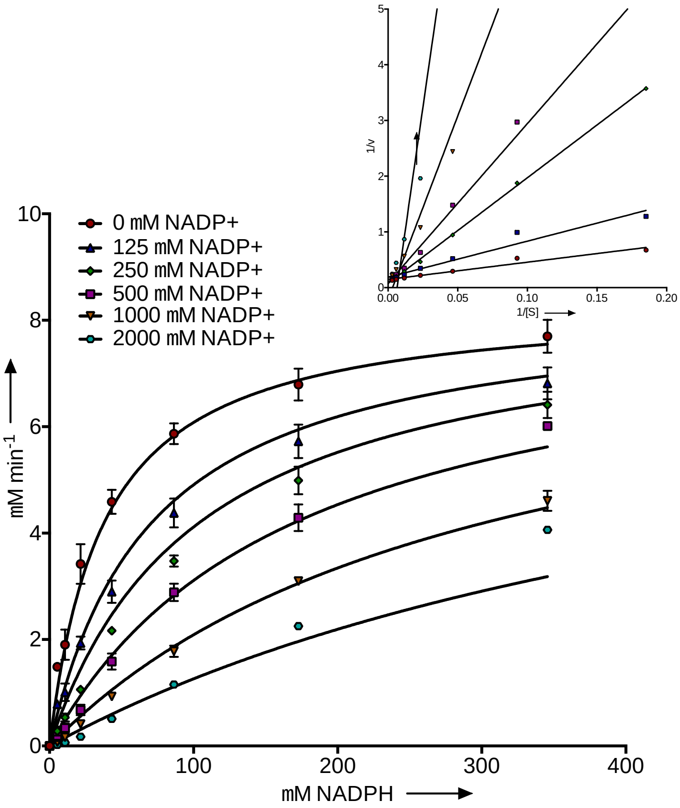

Supplementary Figure 13 – Competitive inhibition of NADPH by NADP⁺ - Initial rates at changing concentrations of NADPH at various NADP⁺ concentrations were fit to Michaelis-Menten inhibition equations in Graphpad using non-linear least squares regression. The best fit was shown to be competitive inhibition and the Lineweaver–Burk plot in the top right of the figure clearly shows this. The K_I was calculated as 143 ± 8 μM.

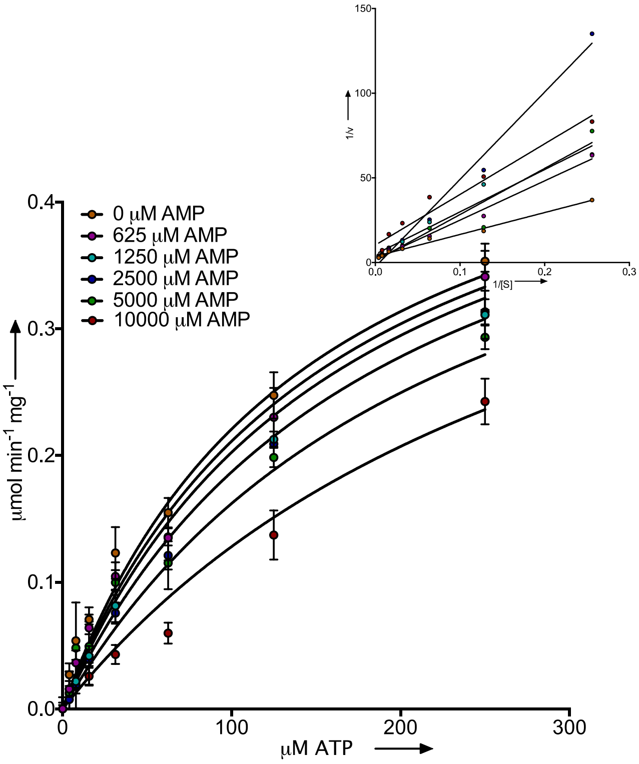

Supplementary Figure 14 – Competitive inhibition of ATP by AMP - Initial rates at changing concentrations of ATP at various AMP concentrations were fit to Michaelis-Menten inhibition equations in Graphpad using non-linear least squares regression. The best fit was shown to be competitive inhibition. The K_I was calculated as 8200 ± 900 μM.

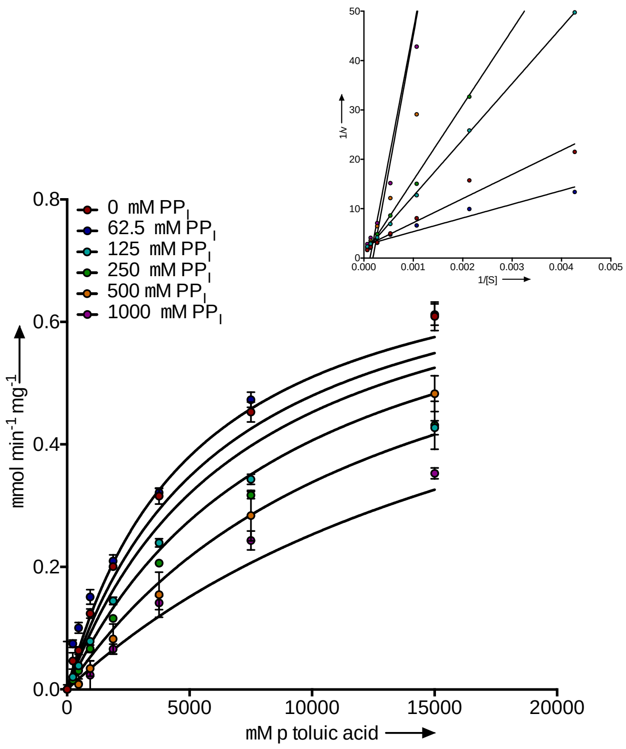

Supplementary Figure 15 – Competitive inhibition of p-toluic acid by PPI - Initial rates at changing concentrations of p-toluic acid at various PPI concentrations were fit to Michaelis-Menten inhibition equations in Graphpad using non-linear least squares regression. The best fit was shown to be competitive inhibition. The K_I was calculated as 340 ± 40 μM.

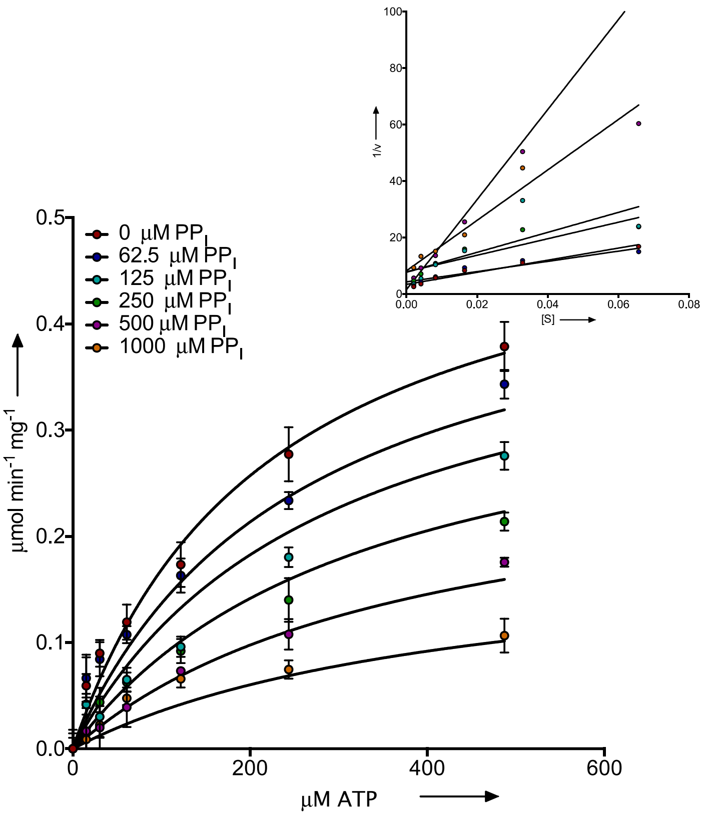

Supplementary Figure 16 – Mixed model inhibition of ATP acid by PPI - Initial rates at changing concentrations of p-toluic acid at various PPI concentrations were fit to Michaelis-Menten inhibition equations in Graphpad using non-linear least squares regression. The best fit was shown to be mixed inhibition, with a K_I of 220 ± 50 μM, and an α of 2.5 ± 1.4.

Supplementary Figure 17 – 124 identified CAR sequences - A FASTA file containing all 124 CAR protein sequences identified. A subset were used for the tree in the main text. An extended tree is shown in supplementary figure 18.

```
>Mycobacterium_marinum
MSITCVDTRAQRSARRIEQLYSTDAQFAAARPSTAVGIAISKSGLGLPQIIQTVMDGYPQRPALGQRATRVVTDPNTGRSSAQLLAEFETITYRELWNRTNALTNAFAAEA
LADRGQRVCVLGFASIDYATIDLALMLLGAVSVPLPTNAARAQLCHIVSETQPSLIASSTENLPDAISLVLSHRAPHRVVVFDYRPELDAHREALEAARARLAAIPVTVET
LTAIIARGRTVRPAEADCGAQSADAPALLIYTSGSTGAPKGVVYTRNRVADFWRTSKAEVEATEQRTAPSITLNFMPMSHANGRQVLYGTLSNGGTAYFTARSDLSTLFDD
LALVRPTELGFPPRIWDMLLERFGREVDRRLRDGTAEGADPGALKARVAADLRQVLLGGRYALAMMGSAPISEQMKASVESLLDLDVMEGYGSTEAGTVIINNEVQRPQVI
DYKLVDVAELGYFLTDRPYPRGELLVKTRTLFSGYYRDPEDGAQVFDPDGFYRTGDIMAQVGPDRLAYLDRRNNVLKLSQGEFVAVSRLEAIFANSPLVRQIFVYANGARA
YPLAVVVPTQDAQSRHGRAELKAELHTSLHRVAMSAGLAPYEIPRDFIVETTPFTPQNGLLTAIHKLARPHLTQRYGARLELLYTELADSQTRRLHRLRQTGGRLPALETI
RRAAGALLGTETTEPRPEAHFKDLGGDSVSAVTFSNLLHDIYGFDVPVGVILGPATDLRALASHVESRRGAGWSGPSFASVHVPRATSVHAGDLKLAKFLDTKTLAAATSL
PAADARARTVLLTGATGFLGRYLVLEWLRRLRAVGGKLICLVRAASDEQARVRLDTAFDSGDPQLPEHFRQLAVDRLEVLAGDKSEPGLGLDGPTWQRLADTVDLIVDPAT
LVNHVLSYRQLFAPNVAGTAELLRLALTTKRKPYAYVSTVSVANQIEPSAFTEDADIREISRTRTIDDSFANGYTTSKWASEVLLREAHDLCGLPVTVFRCDMILADTSYA
GQLNLADTFTRLMLSVAATGIAPASFYRLGPDGKRQPAHFDGLPVEFIAEAVATLGARRHDGFQVHHVANPHHDGVGLDEYVDWLVDAGCPIRRIPDYDEWLSRFETALHA
LPDRKRRHSLLPLLQNYREPAEPIRGGIAPAPRFRGAVRQAKIGRDNDIPHVGPAIIAKYASDLQLLGLA
>Nocardia_jiangxiensis
MSADMREERLARRVADLCATDRQFADARPDEAVTEAIEQPGLRLAEIVRTITAGYADRPALGQRAVELVIDPQTGRRSLDVLPRFETISYRELWDRAGWVARASAGSPLRP
GDRVCMLGFTSVDYTILDTAMIRLGAVAVPLQTSAPVTGLQAIVAETEPRMIAASIENLSDAVELVLTGPTLARLVVFDYHPEVDDEREAFDAARARLARADRDVVVETLA
DLVERGRALPGTPEMATGDNDSLALLIYTSGSTGAPKGAMYPQRMVADLWRRSFGAVWGRRGADPSITLNFMPMSHVLGRGTMYGALGGGGTVYFTAKSDLSTLLEDLALV
RPTQLTFVPRIWDMLFQEFHSELARRSFDGVDRAALEAEVMAEQRQNMLGGRFVVAFTGSAPISTELRAFVESYLDMHLMDGYGSTEAWPIYIDGLVQRPPVIDYKLVDVP
DLGYFHTDRPHPRGELLVKSDTVFPGYYKRPDVTAEVFDQDGYYRTGDVMAEVGPDRLVYLDRRNNVLKLSQGEFVTVSKLEAVFGDSPLIEQIYIYGNSARAYLLAVVVP
SKDASARGDTSALERLLSDELQKIGRTAGLQSYEIPRDFIMETTPFTPENGLLTGIRKLARPKLKERYGERLEHLYTETAEGQAEELRALRRNGSDRPVLETVGRAAAALL
GVAAGDLRPDAHFTDLGGDSLSALTFGNLLHEIFDVDVPVGVIVSPATDLRALAAYIEAQRARDGKRPTFATVHGRDATEVHARDLTLDKFIDAELLDTAATLPRASAQVR
TVLLTGATGFLGRFLALEWLERLSLVGGRLICLVRATDDTAARARLDAVFDSGDTQLLRHYRELAADHLEVVAGDKGEADLGLDRQRWQQLADTVDLIVDPAALVNHVLPY
SQLFGPNALGTAELIRIALTTKLKPFIYLSTVGVGDQITPSAFTEDADIRAISPDRAINDDYANGYGNSKWAGEVLLREAHDLCGLPVSVFRCDMILTDTEYLGQLNLPDM
FTRLMLSLVATGIAPGSFYELDAEGHRQRAHYDGLPVDFIAEAIATLGQHPADGFETYHVMNPYDDGIGLDEYVDWLTEAGYAIQRIPDYAAWLHRFDTAMRALPEQQRQY
SLLPLLHSYRHPAKPISGSIAPTDHFRSAVQAAKIGHDKDIPHVSAPIIVKYITDLQLLQLL
>Nocardia_cyriacigeorgica
MSIESPAARRKQRIDRLYRDDHQARAAAPDEAVLDAVRTPGIGVAQIVTSVLTGYAERPALGQRAFEIVSDPVTGRPERRWLDHFDTLTYGEVADRIEAVAAAWAQAGVEP
GAMIGLLGVTGVELTITDLACARLGAVVVPLQAGAPSAALVQIAVETEPRILVATPELLDRAVDCALACPATRRLIVIDHVPDLDAHRAALTAARERLAGAPITVETLAEM
IDAGRDMPRAAPYADPDADPLAMLIYTSGSTGTPKGAMYTDRLAAGIWVQGLARKPGSNPPAISVNYMPLSHLAGRLALAGVLVRGGIAYFTAAADMSTLFEDIALVRPTE
MVFVPRVSDMIYQRYQSELAGGRSEEQVREQLRGEVLGGRLISASTGSAPLAPELKAFMESLLGIEVHDAYGSTEAGGLVVDNRVRRPVVIDYKLVDVPELGYQTTDEPHP
RGELLLKTATMIPGYYRRPDITAQVFDGEGFYRTGDIVAEIGPDQLRYLDRRNNVLKLSQGEFVAVAQLEAVYAASPLIRQIFVYGSSVRAYLLAVVVPTEAALADADIEA
LTASIAASVRTIAADAGLEPYEIPREFLIETEPFTIDNGLLSGIGKLLRPALEQRYGPRLEQLYRERADGRSAELRALHTSAGEVPVVETVLRAARALLDCTAEQSTPQAH
FADLGGDSLSALSLATVLTEIFGVDVPVSVVTSPATTLGDLAEYIETAQQSGTGRVSFDSVHGAGADQVAAADLALEKFIDAETLAAAAGLPVAATTGTVLITGANGYLGR
WLCLLWLQRMQAVGGRVLCVVRAKDADTARRRLDAAFDSGDVDLLRRYRELADNHLEVIVGDISAPRLGLDEQAWAALARRVDRIVHCAALVNHVLPYEQLFGPNVVGTAE
LIRLAITDRLKPFTYLSTVAVAADVDRAVFTEGGDIRAGSPVRRNGAGYATGYGNSKWAGEVLLRSVHEMCGLPVAVFRSDMVLAHPGYAGQLNVPDMFTRLLIGLAATGL
APASFYRRGADGGRARAHYDGLPADFTAEAITELGAAIDDGHHSFHVVNPHDDGISLDVIVDWLIEAGVAIERVDDYTEWLTRFEAGLRALPEPQRNASVLPLLHAFARPA
PATPGSAIPATEFRAAVRAAKLGPDADIPHLSADLIAKYVGDLRLRGLI
>Mycobacterium_conceptionense
MSFDTRDEQLASRIADLTANDPQFAAAIPSDTVTAAVDVPGLLLPEIVQTVLQGYSDRPALGERAVEYVADPATGRTTARLLPRFDTISYGELWDRVRSLAAALHASGVAV
GDRVAILGFTSADYTVIDTALSQIGAVSVPLQTSSSPEALAPIVTETEPRVIAASVDHLADAVELALTAHAPAQLVVFDHHTEIDDHRDAVASATEKLAGAGVKVETLSEL
LNRGKDLPTPPVPESDGTDPLALLIYTSGSTGAPKGAMYLQSSVAKFWRRNSKAWLGPVSSAINLSFMPMSHVMGRGILYASLAAGGTCYFAARSDLSTLLEDLALTRPTE
LNFVPRVWEMIHSEFQSRVDHRLAEAGADRDAVEAEVLAEVRDQVLGGRFVAAMTGSAPISAELKAWTEDMLGIHLLEGYGSTEAGMALFDGVVQRPPVIDYKLIDVPDLG
YFATDQPYPRGELLIKTENLFPGYYKRPEVTASVFDEDGFYRTGDVVAEVGPDQLRYVDRRNNVLKLAQGEFVTLAKLEAVFGNSPLVQQIYVYGNSAQPYLLAVVVPTDP
SVSKEAIAESLQEVAREADLQSYEIPRDFIVETTPFSLENGLLTGIRKLAWPKLKAHYGERLEQLYAELAETQAAELRELRSASADAPVVETVSRAAGALLGAAASDLGPD
AHFTDLGGDSLSALTFGNLLREIFDVDVPVGVIVSPATDLAGIAEYIENQRSGSQRPTYASVHGRHTAEVSAADLTLDKFLDAATLAAAPNLPKAGSEVRTVLLTGATGFL
GRYLALEWLERMDLVDGKVIALVRAKSDSEARARLDATFDSGDAKLLAHYQGLAADHLEVIAGDKGEENLGLDQQTWQRLADEVDLIVDPAALVNHVLPYSELFGPNALGT
AELIKIALTTKIKPYTYVSTIGVGDQIEPGKFVENVDVREMSAVRKINDGYANGYGNSKWAGEVLLREANDLCGLPVAVFRCDMILADTTYSGQLNLPDMFTRMMLSLVAS
GIAPKSFYELDSDGNRQRSHYDGLPVEFIAESISTLGGQSVESFETYHVMNPYDDGLGMDEFVDWLIEAGYPIERIEDYGQWVQRFESTLRALPDKQRQASLLPLLHNYQK
PERPMLGALAPTDHFRAAVQEAKIGPDKDIPHVSPAVIVKYITDLQQLGLL
>Mycobacterium_vaccae2
MFVVSDHDQWLRRFEQLTSSDAQLVAAQPDPAVTDAISSPDARLVDVMRTVMTGYADRPALGQRAVEFVTDVTGRTVAELQPRFETLTYGETWARVRALAHALINDPVRPG
DRIATLGFTSADYAVVDMAVSLTGAVGVPLQTSAALAQLQPIVVESAPAVILSSVGDLADAVELALSAHAPERVIVFDYHPQLDEHRDAFAAAADRLHDIPLESLDDVIAR
GAEHLAESEPDAGAPDDLRMLVYTSGSTGAPKGAMYTDRLMANCWRGWFSPVDGRLPAITLDFMPLSHVMGRVVLYGTLGAGGTAYFTARSDLSTLLDDLALVRPTRLDFV
PRIWEMLFQEVQNSGGQGKDLLGGRQLFAMTGSAPTSPELRQWAEDYTGIHIIDGYGSTEAGIALVDGEIQRPAVLDYKLVDVPDLGYFTTDRPHPRGELFLKTTNLIPGY
YKRPDVTAELFDADGWYHTGDVMAEVGPDRLEYVDRRNNVLKLAQGEFVTVSTLEAAYGGHPSIRQIFVYGNSSRSYVLAVIVPTDDVLAGGDVTAVKPVLAEALQAVARG
AGLQSFEIPRDFLVETQPFTLENGLLTGIRKLARPQLKQRYGPALEQLYTDLAEGQADVLRELRRTGADGPALQTVTHAAEALLGTTGAAPDTAFTDLGGDSLSALTFGNL
LHDIFDVDVPVGVIVSPASDLAAIAAYVEAQRAGGATRPSYDAVHGPAATEVHAKDLTLDRFLDEATLAHAAALPGPSGEVRTVLLTGATGFLGRYLALHWLEQMSLVGGT
VIALIRAKDDAAARARLDATFSSDPRLLAHYRELAAEHLEVLAGDKGEADLGLTRATWQRLADTVDLIVDPAALVNHVLPYRQLFGPNVVGTAELIRLALTTRIKPFVYVS
TIGVGDGIAPGRFVEDADIRQISATRTIGDNYANGYGNSKWAGEVLLREAHDLAGLPVSVFRCDMIMADTTYAGQLNVPDMFTRLMLSLAVTGIAPNSFYELDEGGSRQRA
HFDGLPVEFIADAISTIGAQVIEGFETYHVMNPHDDAISLDTFVDWLIAAGYPIVRVPGYAAWLQRFDTALRGLPEKQRQASLLPLIHNYQHPEHPINGPLAPADRFRAAV
QEAKIGPDDIPHLSAPVIVKYLTDLELLGLI
>Williamsia_sp._Leaf354
MTENDPQVAAALPNPDLSGSHDPDTPLAVVISTVLQAYADRPALGTRATETTTDASTGRTTRTLVPRFDTVTYGELADRIAAVAAGWAEFVRPGQFVTTLGFTSVDYAVID
LACAHLGAVSVPLQTSATPAALAPIITETEPTVFAVSIDHLADAVELITTGFAPSRLVVFDNFTDDDDHRDLIAQARERLSGTDTAFETLADTVDRGRTAPAVPAFGTPES
GAQDTWADALATLIYTSGSTGTPKGAMYPTRNVARLWTTFWFDTGAIPEISVNYMPMSHVAGRAVLAKTLGSGGTAYFVAASDLSTLLEDLALVRPTALMLVPRVCDMIFQ
RFRSQVDHRLDADATDVGAVEDAVKAELREELLGGRVLSAMCGTAPLAPEMAEFITTCADVPLVDGYGSTEAGPVLINGVVNQPPVTEYKLIDVPELGYHTSDTPHPRGEL
VLKSDSMFPGYYKRDAVTAEVFDDDGFYLTGDIMAETEPGHLVYLDRRKNVLKLSQGEFVAVSRLESIFVTADGVAQIFVYGNSSRAFLLAVVVPTDEVSAEIDSDAQLRT
RLLDAIGESARGAGLNSYEIPRDIIVERSPFTREAGLLSGVGKLLRPALTEHYRGSLEALYTQLADGQNDELRDLRRHGNDRPVLETVTRAAMATVGGSGQVEGSVRFSDL
GGDSLSALTFSNLLTDIFDVEIPVGVLMSAATDLSAIAEHIERARAGAVRPTFTSVHGKGATEVFAEQLTLDKFIDAETLSAAPSLPRAPEKPGTVLLTGANGYLGRFLCL
EWLERLDASDGRLVCIIRGRDADDARARLESAFDSGDPELLSRFRELAGRRLEVLAGDIADTDLGLDASTWQRLADEVDLIVHPAALVNHVLPYDQLFGPNVVGTAELIAL
ALTSRVKPITYLSTVGVAADGGPSILDEDLDIRSASARRSVDETYANGYGNSKWAGEVLLREAHDLCELPVAVFRSDMILAHSTFGGQLNVPDMFTRLVLSVMASGVAPGS
```

```
FYRSDDGSTDRPRAHYDGLPADFTATAITDLGSSVHKGYETYNVVNPHDDGISLDTVVDWLIDSGVAVTRIDDYASWVARFETSIRALPEAQRRASLLPLLHAFAEPDVAI
AGSAIPSRRFAAAVAEARIGDNGEIPHLSAELIAKYVADLRSLGLVDAAR
>Williamsia_sp._ARP1
MSVDTQDDQLARRIADLVGDDAQFAAARPDDAVTAAINEPSLRLAQIMDTLAEGYADRPALGARAVEFVEDPATGRTTTTLLPRFETITYRELWARLRSVAAALADGAVSP
GDRVGVLGFTSIDYAVIDMATVLLGAVCVPLQTSAPLTQLQPIVDETEPTLIAASVDHLGDAIGLSGNSESPARLIVFDLHPEVDDHRDAVADAQRRLADGEHAVTVEALA
DVIARGESKVTGPVPVVGEGDPLSLLIYTSGSTGRPKGAMYPESLVTNAWRRSTNMVGAQGTTGPSITLSFMPMSHVMGRHILFETLASGGTSYFAATSDLSTFLEDLALV
RPTLLSFVPRIWEMIFTEHQSALNRRVNAGEDEATASEAVSADLRDNLLGGRYVAALTGSAPISAEMTAFVESLLGLHLVEGYGSTEAGMVFIDGAVRRPPVIDYKLVDVP
DLGYFHTDRPNPRGELLVKSDTMFPGYFNRPDATADVFDEDGYYRTGDVVAETGPDQLVYLDRRNNVLKLSQGEFVTVSKVEAVFADSPVIGQIYIYGNSARAFLLAVIVP
TDDALAQVDSDVDAVKPILGESLQRAAKAAGLQSYEIPRDFIVETTPFTLENGLLTGIRKLARPRLKEKYGEALEQHYADLADGQTNELAALRRDGDTAPVLESVSRAAAA
LLSASASDVQADAHFTDLGGDSLSAVTFANLLREIFDIDVPVGVIVNPAADLQAIADYIDNERNSGATRPTFATVHGADATAVHARDLTLDKFVDAETLAAAPSLPRPGEQ
VRTVLLTGATGFLGRFLALEWLERMDLVDGTLICIVRAKDDAAARARLDATFDSGDPALLARYEKLAADHLEVVAGDKSEADLGLDAQTWQRIADTVDLIVDPAALVNHVL
PYAQLFGPNALGTAELIRIALTSKLKQFAYVSTIGVGAGIEPASAFVEDADIREISATRAVDETYANGYGTSKWAGEVLLWEAHDLCGLPVSVFRCDMILADTQFVGQLNV
PDMFTRLMLSLVATGVAPKSFYELDSDGNRQRAHYDGLPVDFIAEAISTLSVQLLDGFETYHVMNPYDDGIGQDQFVDWLVDAGYSIQRVDHYDTWLQRFETAVRALPAKQ
RSASLLPLLHNYQQPVPPLNGAMASTDRFRAAVQDAKIGPDKDIPHITPQIIVKYATNLEVLGLL
>Nocardia_tenerifensis
MYASDAQTRLARIDEAVSAAAQQPGLTVGQIVATVFAGYADRPALGERAVEIVTDAVTGCRAARILPSYHTITYGEFATRIAEVAAAWHSDPLPVRPGDRVCVLGFASIDC
LTLDLAATHLGAVVVPLQAGAPASQLTPILAETEPRVVATSLAQLPVVVDAVRGADLSAPRLVVYDYHSDDDAQRSTFEAARARLADANSPVTAESLATLRTRGAALPAVP
AYAEADGDDLAMLIYTSGSTGTPKGAMHTQRIQRGKWLSGLDNSGDAALDVAAISLHYLPLSHVFGRSTLVGTLVRGGTAYFAARGDMSTLLADFALVRPTRVNLVPRVCD
MLFQHYLTELERCGIDSEAARARLRRRVFGERVVTAAIAGAPLTSAMREFMESLLDAELHDLYGSTEAGLLTVDNTVERAQVAEYKLVDVPELGYFTSDRPHPRGELLVKM
RNPMPGYFQRPEVTAAVFDDDGFYKTGDIVARIGPDELVYLDRRSNVLKLSQGEFVTVARLEAVYGKCPWIRQIFVYGNSERAFLLAVIVPTAAALAMFGSGNALMTALRK
SLAHTAAEAELNSYEIPRDFLVETEPFSVENGLLSGAGKLLRPKLLARYRDRLEERYVTLSESRADELRALRVSAAGLSALESVSRAARALLGVADSELRPDMRFLDLGGD
SLSALTFADLLRDVLDIEVPVGFIISPATDLAAIAHYIDAARTLESARPRYASVHGCSDRVRPGDLALDAFIDAETRTAAMDSAHVTGEPRTVLLTGANGYIGRFLCLEWM
ERLQTIGGTLICLLRGVDSEHARRRLDDVFDTGDAELLDRYRELAAGTLEVITGDVSARYFGLDESKWTDLAQRVDFVVHPAALVNHLLPYRELFGPNVVGTAEIIRLAVT
ARIKPVVYLSTVAVASDIDPERVAQSEGDDIRDVSPERTLSDARYANGYGNSKWAGEVLLREAHDLCAVPVTVFRANMTLAHSRYAGQLNVPDAFTRLLFSLVATGIAPRS
FYAPDDAGRRPHYDGLPVDFTAAAITALGGTDGFRTYHVVNPHDDGRSLDQFVDWLVEAGHSIHRVDDYHEWFARFGTALRSLPEKQRARSILPLLSSYSRPATAVSGSTL
PATAFRDAVQRARVGPNGDIPHLERSLIDKYVSDLRLLGLLQPSRQFKTR
>Rhodococcus_fascians
MKSTGAVDSEHDRDIRQRDRFQHLHDSDPEFRAAEPDRSVADAARELAPNLARVVAEIMTRYADRPALGRRARMIEDTEGSTTARLLPRFDTVTYGQAWADAGALASALGH
DDGGIAAGDFVATLGFAGIDYVITELATIRVGAVAVPLQAGATAGQLRAIVDEVEPVVLASDVDNLAVAMEVAGHCRSIRTVVVLDYDDRIDADTRALTSAREGVHGSGIA
VRPLTELVDEGSRLPQVPLFDVEDPERLAALIYTSGSTGTPKGAMHTEQIVSGAWTGAWHRTGASSEGVDEPAFPVITLDYLPMSHLAGRGLVFSTLAAGGTVHFAGSSDL
STLFEDFALARPTLALLIPRVCEMIRHTVLAEIDRAVDGTDAGEHEQISRGVLERVRSEQFGGRILAAMVGTAPIAAEVKDFVTDLLDVRVRDNYGSTEAGMVLLDGIVAR
PPVLEYKLDDVPELGYFSTDTPHPRGELLLKTTSIIPGYYKRPDVTADVFDSDGFYRTGDVVAELEPDRLAYVDRRKNVLKLAQGEFVALARLEAVFGTSELVDQIFLYGN
SQRSYLLAIVVPAQANSTAGAIMESLQRIARTESLNSYEIPREVVLEPQPFTQDNGLLSGAGKQLRPKLVERYGDELERRYAELESGQTDRLRELRRSGAQAPVLTTVGDA
AQALLGCSGADIRPDAHFTDLGGDSLSALTFSTLLRDIYDVDVPVGVITGPAMDLSALADYITSAIDNDSDTATFASVHGREATVALASDLTLSAFLDASLLDAAYRIPGP
RPVTNTVLLTGANGYLGRFLCLEWLEKMAAAGGRVICLVRGRSDADARGRLDAAFDSGDATLSDRYRALADAALDVIAGDLGTPRFGLTDDVWEELSRRVDRVVHPAALVN
HALPYEHLFGPNVVGTAEVIRLALTDHIKPVTYLSTVAVAVGVDDFHENGDIRVDSPQRAVDESYANGYGNSKWAGEVLLRNAFDEYSLPVSVFRSDMILAHSTWSGQLNV
PDVFTRLILSVVATGLAPRSFYATDAGAPTDEQGRPLAHYDGLPADFSAAAITSIAGDDVTGYRTFDILNPYDDDISLDTFVDWLRDAGCTIDIVDDYDEWFERFSAAVQE
LPEKQRSHSLLPLIHSYAHPQHPSSGAILPADEFASAVGDIPHLGRELIEKYLADLVALGLVAKS
>Sciscionella_sp._SE31
MESDAQFRAAMPSRQVSEFAREPGLGLGQTIARIMQGYADRPATAERATELVADPVSGRRSLRLLPEFRTRTYGQLWADATAIATEWAGSGIGAGDFIATIGFTSSNYVVL
DLTAVYLGAVSVPLQPSSAVPQLQAIVDETTPALFAASPGYLDKAVELILASGSKPRQLMVFDYHDEVDEEREGFESAVARLSAAGVEVRTLEDVLEHGKAKPAAKQFVPA
PGEDPLTMLIYTSGSTGAPKGAMYPEHTMLRYWCGELPVEDQSPAFTLSFMPMSHMFGRVTIASTLGRGGTNYFAAKADLSTLFEDLALARPTELFAVPRIFDMLFQEYQG
ELDRREAEFSDPAELDAAVKADLHTRFLGGRVLTAMVGSAPISAEIKAFAEACLQIPLHDGYGATEFGVVLFDGEIQRPPVREYKLIDVPELGYFHTDRPHPRGELLLKTD
TIFPGYYNRPEVTASMFDEDGFYKTGDVMAETGPDRLVYVDRRNNVQKLSQGEFVTLSKVESVFTANPLIRQIFAYGSSERPYLLAVVVPSEQALAEAGSAEAVKPKLAEA
IGQTAKEAGLEAFEIPRDFLVEPEPFSMANGLLSDIRKLLRPRLRERYGEQLEALYAELAHGQTDELRALRRGGPGQPVYETLARAARALLGCAESEIHEHARFTELGGDS
LSALSFAKLLGEIFEVDVLVGVVISPATDLRAIADYVTAQRSGEGQRPSFASVHGADATEIRASELTLEKFIDPATIAAATGLPGPAATPRTVLLTGATGYLGRFMCLDWL
ERLAENGGKLVCLVRGTDDAAARERLDATFDTGDAELLRHYRELAEDHLEVLAGDIGEADLGLDGAVWQRLAGEVDTIMHPGALVNHVLPYEQLFGPNVVGTAELIRLAIT
GRIKPFTNVSTIGVADQIEPGRFDEVTDIRELSSVRQINDDYANGYGMSKWAGEVLLREAHDSCGLPVSVFRSDMILTHSRYTGQLNLPDMFTRLMLSLVATGIAPESFYR
TDSAHAHYDGLPVDFSAEAIDTIGGGNTEGYHTYHVFNPHDDGISLDTFIGWLIEAGYRIERVPGYQDWLTRFSTALRGLPENQRQHSVLPLLHSYAEPGEPVSGGLVPAD
QFRKAVQEAKIGADHDIPHITKELIVKYITDLEQLELL
>Sciscionella_marina
MPSDTREERLARRVAELYATDQQFAAAQPDMTVSNMVERAGSRLPEIVSTLLKGYAQRPALGQRAVRFAEDPATGRTRAELLPRFETITYQQLADRVGAVAAALASDPIAP
GDRVCVLGFTSVDYAIVDLALLALNAVSVPLQASAAAAALRPVVAETEPTVIATSVDYLSVAVGLVSAGSVPVRLVVFDYYPQVDDHREAVEAARARLADTGSPVVVELLA
DLLERGTTRPASVIESEADNPLALLIYTSGSTGRPKGAMYRQDLVALIWRSSALTARVRERVVPSITLNFRPMSHVVGRAALYGTLGTGGTAYFAATRDLSTFFEDLALVR
PTRLDFVPRVWEMLYDEYRREVTARSVPGGDRAAVEAQVMAEQRQSLLGGRYVSASTGSAPMSAELRAWVESFSDLHLADTFGLTESVLVLIDGRVQRPPVIDYKLVDVPE
LGYFGSDRPHPRGELAVKSATQFAGYYKRPELTAEVFDSDGYYLTGDIMAEVGPDQLTYIGRRNNVLKLAQGEFITAAKLEATFGGSPLVRQVYVYANSERSYPLAVVVPT
ENALQQHDVDSLKPLINESLRDIAKVAELQSHEIPRDILIETSPFTADNGLLTDLGKPAQKKLQQCYGARLEQRYSELAEEQAKKLRALRDSAAGRPVLDTVRRAASAVLG
ADGADPRPDAHFAELGGDSLSALTFGNLLRETFDVDVPVGVIVSPATDLAALAAYIEEQREGSRRPSFATVHGRGATTVHAGDLTLDTFLDADTLAAAPSLPKAATEVRTV
LLTGATGFLGRYLALDWLKRMDLVGGKVIALVRAKSDEEAGARLDEVFDSGDPELLEYYRKLAADHLEVIAGDKGEADLGLDEANWWRLADTVDVIVDPAALVNHLLPYSE
LFGPNTLGTAELIKLALTTKIKPYTYVSTIGVGDQIEAGKFVEHADIRRASATRAVNDSYANGYGNSKWAAEVLLREASDLCGLPVTVFRCGMILADTSYAGQLNLPDMVT
RMLLSLVATGIAPGSFYEVDAGGNRQYAHFDGLPVEFIAEAITTLAAAGGGEFATYHVMNPHDDGLGMDEFTDWLIEARYEITRVADYDEWLQRFDTTLRALPEKQRQASL
LPLLHNYRKPQTPINGSLAPTDRFRAAVQESKIGPANDIPHVSRPIIIKYVTDLQLLGLL
>Rhodococcus_wratislaviensis
MSTDIREERLARRIADLYANDRQFVAARPSEALTAAIEQPGLRLPQLVRTVMEGYADRPALGQRAVQFIKDPATGRTFPELLPRFDTITYHELWDRVGAVASALAGGRSPS
VRPGDRVCILGFTSVDYTTIDMALVQMGAVCVPLQSSAPFTQLRPIVAETEPRMIASSIDYLADAVELVLTGHAHARLVVFDYHPEVDDQIEAYDAARARLTEAGSPAVLE
RLTDVLERGQTLPAAPVFIPDDPLTLLIYTSGSTGAPKGAMYPERLVANFWRRSTRASWGQQGAEPSITLSFLPMSHAMGRGILYGTLGNGGTAYFAAKSDLSTFLDDLAL
VRPTQLTFVPRIWDMLLQEFRSEVDRRSSDGTDRGALEAEVMAEQRQTLLGGRFVSALSSSAPISAETKAFVEYCLDLRLVEGYGSTEAGSVFVDGVVRRPPVIDYKLADT
PGLGYFHTDQPHPRGELLVRSDDVFPGYYKRPEVTAEVFDVDGYYRTGDIVAEIAPDQLVYLDRRNNVLKLSQGEFVAVSKLEAVFSSSPLVRQIYVYGNSARAYLLAVVV
PTESALSRGSGDLESLQPLISDSLQDVARTAGLQSYEIPRGFIVEASPFTLENGLLTGIRKLARPSLKERYGQRLEQMYTALAEGQTDELRALRRSGADRPVLETVTRAAG
ALLSAAAVDLQPDAHFTDLGGDSLSALTFANLLREIFDIDVPVGVIVSPATDLRVIADYIEAKRTSSTKRPTFATVHGRDATELRASDLTLDKFLDGTTRAAAPTLPGPSA
EIRTVLLTGATGFLGRYLALEWLQRMEGVGGTLICLVRAKDDAAARSRLDTIFDGGDPDLLRHYRELAADHLEVIVGDMSETNLGLDKQTWQRLADTVDLIVDPAALVNHV
LPYCQLFGPNVLGTAELIRIALTTKRKPFAYVSTIGVGAQIEPATFAEDADIRVVSPTRTVDDSYANGYSNSKWAGEVLLREAHDLCGLPVAVFRCDMILVDTEYAGQLNL
PDMFTRMMLSLVATGIAPGSFYELDANGNRQRAHYDGLPVDFIAEAMATLAEKVTDGFETYHVMNPYDDGIGLDDYVDWLIDAGYSIQRIPDYGAWLQRFETAMRALPEQQ
RQHSLLPLLHNYQRPEKPVCGSIAPTERFRAAVHDAKIGPDKDIPHVVAPIIVKYITNLQLLGLL
>Mycobacterium_rhodesiae3
```

MSIDTREARFEQRVADLYATDAQFAAATPDPAVTAAASRPGLGLAEVVETVFDGYADRPALGQRVVRFIEDPQTHRTTAELEPHFETITYRDVWDRVRAVAAAWSDNGVRP
GDRVAILGFTSVDYTIIDLALIQLGAVSVPLQTSASVTQLQPIITETEPVLIASSIDNLDGAVELALAGPTPKRLVVFDFRPEVDDQREALAAITQRLADTDVTVATLSGE
IAYGTYRSIDPAGTTDADPLALLIYTSGSTGAPKGAMYPAAKVADMWLAAAAHWDDKQGAYPAIVLSFMPMSHVMGRGALYGTLSSGGTVYFAARPDLSTFLEDLALTRPT
QLNLVPRVWDMIHQEVQSDVDRRGDEAQVLAEKRASLLGGRFVSVMTGSAPIAPELRSWVETFLDMHLIEGYGSTEAGAVFVDGVVRRPPVTDYKLVDVPELGYFGTDLPH
PRGELLVKTTQLFPGYYKRPEVTAAMFDEEGFYRTGDIVAEPAPDRLQYVDRRNNVLKLSQGEFVTVSKLEAAFLGSPLVHQIYLYGNSARPYLLAVVVPTADAAAAHDAA
ELKTLIGESLQEVARATGLQSYEIPRDFLIETTPFTVENGLLTGIRKLAWPRLKERYGPALEQLYTDLADGQADELRALRQSGAKAPVLTTITRAAGALLGAAAADLQPDA
HFTDLGGDSLSALTFANLLNEIFEIEVPVGVIVSPANDLQAIADYIDAQRQEGGKRPTFASVHGAGATEAHAGDLTLDRFIDAATLAAAPRLPGPSGEIRTVLLTGATGFL
GRYLALEWLDRMSLVGGTVICLVRARSNDEARVRLDATFDSGDPKLLKRYRELAAEHLEVIAGDKGEKNLGLDALTWQRLADTVDLIVDPAALVNHVLPYRELFGPNAVGT
AELIRIALTSKIKPFVYVSTIGVGAGLAPGEFTEDGDIRAISPTRAVDDSYANGYSNSKWAGEVLLREANDLCGLPVSVFRCDMILADTAYAGQLNLPDMFTRMMLSLVAT
GIAPASFYRLDAGGNRPRAHYDGLPVEFIAEAISTLGADVGKDFETYHVMNPYDDGVGLDEFVDWLIEAGYPVRRVGEYASWLQRFETSMRALPDRQRQYSLLPLLHNYQH
PEQPVRGSIAPTDRFRSAVQDAKIGPDKDIPHVTPEVIVKYVTDLQLLGLL
>Kutzneria_albida
MRAQDEQVRAAAPLDAVNEATSSPGQRLTQVVAAIMAGYADRPALGERARELVTDPGTGRTSIRLLPWFDTISYRELWTRVGAIASDWHHHPDHPLAAGEFVGILGFTSCD
YTTLDLVCLHLGAVCVPLQSSSPASQLRPIIAETGPSILATSAERLDTAVELALGSPTVRRLVVFDSHPEVDEQREALESARQRLTEAGHPAVVDSLAAVLERGRALPPAP
LFTPGPDEDPLTMLIYTSGSTGTPKGAMYPERLVHSLWDGLWRDKNALPVIGINYMPMSHLAGRISLLRALSSGGTSYFAAKSDLSTLFEDIALIRPTELNLVPRVCDMLF
QRYQSELDRRAPGTSDLDAVDAQVKQELREGFLGGRVVRAMCSTAPLSAEMAAFVESCLDLELHDGYGSTEAGGVVIDKHVLRPPVLDYKLVDVPELGYFRTDTPHPRGEL
LIKTRTIIPGYFKRPDATAEIFDADGYYQTGDIMAEIGPDQLVYVDRRKNVLKLSQGEFVAVSRLEAVFATSPLVRQVFVYGSSARAYLLAVVVPTEEALRRTVTDNAALK
SSISESLQRIAREAELNSYEIPRDLLIETDPFSTENGLLSDARKLLRPRLKEHYGERLEQLYAELAKGQVDELHALRVTGRDRPVLETVTRAAQALLGCASTDLSPDAHFT
ELGGDSLSALSLSNLLQEIFTVEVPVGVVISPANDLRQLANYVETELSSGAKRPTFATVHGQGSLEVRAADLTLDKFIDSATLAGAKDLPGPSGTARTVLLTGANGYLGRF
LCLEWLRRLSQDGGKLVCIVRGSSAEAARRRLEQAFDSGDAELLRLFRELAAEHLEVLAGDIGEPDLGLDEQTWHRLADSVDLIVHPAALVNHVLPYQQLFGPNVVGTAGL
IRMAITKRLKPFVYLSTVGVLSAQIAPSALREDLDIRDTSPVRRLDQSYASGYGTSKWAGEVLLREAHEAFGLPAVVFRSDMILAHSRYTGQLNVPDMFTRLLLSLVLTGI
APKSFYRTGSDGGRQRAHYDGLPAEFTAEAITELGARAAAGYRTFNVLNPHDDGISLDVLVDWLAETGHPIQRIEDYQEWFARFDTALRALPEKQRQHCLLPLMHAFEQPG
VPVAGSVIPADEFRAAVRTAKIGPDKDIPHLSASLITKYVRDLEQLGLV
>Kibdelosporangium_sp._MJ126-NF4
MCATDQQVRAAIPLADVSAAIHQPDLSLHQIVATVMTGYADRPALAARAWDVTADPVTGRAGSRLLPRYDTISYRELWDRAGAVAADWYHDPHTPLRGNEFVCTLGFTSPD
YMTIDLACVRLGAVTVPLQHSASVDHLAPIVAEVEPRLLATSLEQLPIAVDCALASTTVRRVVVFDYHPEIDDHREALETARLRLTESGRPITLTTLAAALERDRTLPPAP
VPTPGDDQLALIIYTSGSTGTPKGAMFSQRRVASMWLGGMIPVVAGQPSLNINYMPMSHVAGRGTLTATLSTGGTCYFTARSDLSTLFEDIELARPTEMLLVPRICDMVFE
RYHSELNRRAVNGADRAVVAAQVRGEVREKLLGGRVASFTTGSAPLSAELADFLESCLDLPIHDGYGSTEAGAILFDRKLARPPVLDYKLVDVPELGYFGTDAPYPRGELL
IKSQTLMPGYFKRPDVTAEVFDADGYYRTGDIMAQIGPDELVYVDRSKNVLKLSQGEFVAVSRLEALFVTSPLVRQIFVYGNSERAYLLAVVVPTGDALRHVAGDLGQLRA
LLHDSLRQVAKEAGLNSYEIPRELVIETEPFSTANGLLSEIRKNLRPRLTERYGRRLEQLYTEIAERETEELRALRTVGTDQSMIDIVLRVADTVLGASGVARPDSHFTDL
GGDSLSALSFSNLLKEVVGVEVPVGVIVSPANDLQRLSGHVEAALRPGVARPTFDTVHGADSTQARASDLTLDKFIDATTLDAASGLPAVSGRAHTVLLTGANGYLGRFLC
LEWLERLAERGGTLVCVVRGSDAAAARKRLDMAFDSGDPDLLDHYRELAAEHLEVLAGDIGEPDLGLDESTWRRLADTVDLIVHPAALVNHVLPYQQLFGPNVVGTAELIR
MALTSRIKPVAYLSTAAVLDAHTSVIDEDSDIRQTSPVRELDQGYASGYATSKWAGEVLLREASDAYGLPVAVFRSDMILAHSRFAGQLNVTDMFTRLLFTLITTGIAPSS
FYRANAGGDRPRAHYDGLPADFTSEAITTLTEQNNSGYRTFNVLNPHDDGISLDLFVDWLVEAGNPITRIEDYQEWLRRTETALHALPERQKQQSLLPLLHAYAHPGEGNV
SGIPVDRFRAAVRANKVGPDQDIPHITKELILKYVADLRTLRLI
>Salinispora_arenicola
MTTTEQTLTERLIAEDEQIRRAQVSAEVSAAMRVPGMSQAQIVAAGFTGYADRAALGERAREAVTDPVTGRTTHRLLPWFDTITYGEVRSRVLAISAAWWHDVDAPLRPGA
FVVSVGVPSADLVTVELAVLHTGAVSVPLQVSSTAEQLRPILDEAAPLIVATSVDRLAVVTAAMSGNASVRRIMVLNHDAAITAHRDAVDAARSALAGTAVVVHTLTEVLD
RGRGLPAPEPYAAPTGEDPLSLLIYTSGSTGTPKGAMFPESMTRANWVRFDPKPTDMAVIRLNYLPLSHNVGRIVLFEALAVGGIAFFTAHSDLSTLLEDMALARPTDLFL
IPRLCDMLAQRHDSELARRRITTADHEGVRQVHTHLREAVLGGRVTRAMSLSAPLSPQLRRFVESCLGFAVHDVFGSTEAGGLLVNGRVLRPPVLDYRLVDVPDLGYFTTD
RPYPRGELLVRTATIIPGYYQRPELNAELFTEDGYYRTGDIMAEYGPDHLGYVDRTTSVLKLSQGEFVAVSRLEELFAASPLIRQIYLYGNSERPYLLAVVVPTEEAHAAT
REPAALKAVLGESLQRIAQQHGLHPYEVPRDLLIETTPFSTANGLLSDIRKPLRPKLKTRYAPRLEALYTELAEREADRIRTLRDAGSAQPVLPALREAARAFLGRPGAAL
DVNDRFVDLGGDSLSALALSNLLSDIFEVRVPVGIMISATGTLGSVAAWIEAERATAGAGIGRATPTSVHGANLTQVHADDLTLGTFLDVTTLAAAACLPRAPLSDPRVVL
LTGATGYLGRFLALEWLDRLSRSGGTLVCVVRAADDAEAARRLESVYGSSDPELLERFRSLAGHVRVLAGDVAEARFGLPAGVWQELAETVDLIVHSAALVNHVLPYEQLF
GPNVAGTAELVRLAVSVRVKGIAFLSTVAVITSQTTTPDEDADIRQASPHRVLDDSYANGYAASKWAGEVLLRRAHEEYGVPVSVFRSDVILAHSRYAGQLNVPDMFTRLL
LSILATGIAPASFYRTGPDGERQPAHYDGLPVDFTAAAVAAVGVTEGHRTFNVLNPHEDGIGLDTFVDWLVAAGHPVQRIADHDEWVTRFATAMRGLPERQRRSSILPLLH
AFAEPAPPTFGSRLPTDRFRAAVKAANVVPGNEIPHLDAALVTKYADDLRLLDLL
>Sphingobium_sp._C100
MDDSVYAQRKADRIGMLMARDTQFRDSAPLDSVVAAKTRPGLSVAEAMATVMEGYADRPALGQRARELVMDPATGRHMLKLLPRFETISYRDLWANARAIASEWHHNGANP
LSPGDFVCILGFTSPDYATLLLANTHRGAVNVPLQTSAPASQHAAIIAETEPRILATSIDYVDNAVDAVLAGITPPRLVVFDFDERDDDHRDALAEARARLTREGRAVVVD
TLGEVIARGRSMEEPSLYKPAEGEDPLGWLFYTSGSTGTPKGAMFTHSLCMNTWTGHNPMTAITLSFMPMSHLVGNGYLLMALSNGGTSYCSPKSDLSTLFEDLALVRPTQ
ASLVPRVCEIFYHRFLSDVDARVAAGIDIATAEAEVKRDMRERLLGGRLVSVGCGSASLAPETYAFMESMLDTHMAIGYSSTEMAGGTVLVDWKVQRNQVIDYKLADVPEL
GYFSTDKPHPRGELLVKPSRFMGGYYKRPDLTAEKMDADGYYATGDVMAELAPDHLVYVDRRNNVIKLSQGEFVAISRLEALYVHSPAIRQIYLYGTSERAFLVAVIVPGE
ELAERLGAPGGHDAVKAELRRALQQIAEKERLNGYEVPRDFLIETEPFSLRNGLLSEVGKHQRPKLKDRYGERLEQIYTQLAQDQVDVLRSLRAGGGDMSVLDAVNRATQA
TLGVGASDVRPDARFVELGGDSLAALTFSTLLEEIFGVEVPVGVIIDPTGSLQRIADYIEVARGTGDSARRPTFASVHDADSALVEASDLALHKFIDEATLAAAPALPPPD
GEIRTVFMTGATGYLGRFQALAWLERLATTGGKLICIARGANAQEAGARIEAALDSDETLLDHFRTLAADHLEVLAGDIGLPNLGLDAASWTRLAETVDLIVHPAAHVNHL
LPYNQLFAANVAGTAELIRLAINTRIKRLNILSTLGINTVAQMLVDEDGDVRVTIPRCTMNDSYANGYGISKWAGEVLLREAFDLCRVPVSVFRPGMILAHSRYAGQLNVP
DMFTRLLYSLAVTGLAPGSFYAADTANGRPRGRYEGFAVDFLADVLTAIGASDRPGYHSYNLASTHEDCSSLDDFVDWMVEAGCPIERIDQYDAWIDRFETVLRALPDDQR
QQSVLAILDPYRHHQTPGGRMHLPAERFRAAAEAAGFDVPAVSKALIAKYVEDLRHLKLL
>Ideonella_sp._B508-1
MQAANARPGTPLAQIVTTVLESYADRPALGQRARELVTDPSTGRRTLQLMPAFETISYRELRARVDALARACHHDADAPVRPGDRICMMGFAGIDYAVIDLACILLGVVSV
PLQTHAPTAQTVRILEETQARWFATSLDCLDAAIDAVLAGARPARLVVFECAIEDEDQRERLDAGRRRLAAAGVALPLETMAETLRRGAALPPAPPAPPASDDTLVTLFYT
SGSTGSPKGAMYTERMFRPPWLRPATHPLVCLNYMPMNHSFGRSWLGMILGSGGLCCFTARSDLSTLFEDIELVRPTMVNLVPRICEMLYHRFVDDVERRLAADAATTKEV
AVRDALERLRVRSLGGRVLTATTGAAPLTDEMAAFIEAALDTRLNNGYGTTEVGMVSFNTQIARPPVLDYKLVDVPELGYFRTDKPYPRGELLVKTLSAMPGYYANPEATA
AMFDEDGYYRTGDIMAEIGPDRIVYVDRRNNVLKLAQGEFVAIAKLEAVFAGGHPAVHQIYVHGSSERAYLLAVVVPHAPAVEAALGPRPEPAAVKALLREALRSAAQAAG
LNSYEVPRDFIVEPEPFSVENGLLTSVGKARRPGLKERYDARLAALYSALADDQAEELAALRRDGARRPVLETMARAVKATLGIADIPADARVSFQDLGGDSLSALSFSLL
LEEVFGVELPVSAVIGPDSSLQRLAAMVEARRAGGVRRPGFAEIHGRDATEVHASDLVLDKFIDTAALSAARALPRANRTPRTVLLTGANGFLGRFLCLEWLERLATSGGR
LVCIARGADAAAARGRIAEAFDGGDPGLKAHFETLAGAHLEVLAGDIGEPGLGLDAAAWQRLAEEVDLIVHPAALVNHRLPYAQLFGPNVAGTAELIRLALTARVKPFVNV
STVAVAINPDGALIDEDADVRVAAPRRPIDGRYAGGYATSKWAGEVLLREAHERCGLPVTVLRSDMILAHSRFHGQLNVPDMFTRLILSLVVTGLAPASFYQPGPAGAMRP
HYDGLPVDFVAAAVAALGDGDADGFRTYHVLNPHDDGISLDSFADWIAESGHRLERVADHADWLARFETALRALPDERRQQSVLALLDAFRQPMPATAGAKLSAARFHEAV
RLRGIGTEGDIPHLSPALIRKYLSDLSALRMI
>Streptomyces_showdoensis
MQSHADRPAIGERVKELATDAETGRVSIRLLPRYETTTYRELWARVRAVASEWHHHPDRPLAAGERVALLGFTSRDYTTIDLACIHLGAVSVPLQTSSAPAQLRAIVAETE
PFTVAASLERLHVAVDLVAASDAVRRLVVFDVHPEADAHREALAAARERLAETRPDVTLDVLGDLVERGSALPEAPLFTAAEGEDPLAMLIYTSGSTGTPKGAMYTERLAA
AMWGGAWAKLFDETYAVTFHYAPMSHVAGHSSLKATMARGGTSYFTASSDLSTFFEDVALARPTELSLVPRVCEMLHQRFRGDVDRRVAAGADRATAEAEVRTHLRENVLG
GRVTWASSSSAPLSAELTAFTESLLGLELHNVYGSTEAGVSVDGVLLRPPVRDYKLADVPELGYFRTDTPHPRGELLLKTDVIIPGYYKQPELSAELFDEDGYYRTGDIA

AELGPDRIAIVDRRKSVLKLSQGEFVATSRLEALFAASPLVRQIFVYGNSERSYLLAVVVPTPETLAELGAESPVLKQRIGETLQELAKEAGLNSYEIPRDVLIETEPFSQ
ANGLLSDHRKLLWPRLVERYGERLEELYADTEAREDAELADIRAAGADRPVLETVQRAVRALISGSLAEVDPAAHFRDLGGDSLTAVQFSTLLQETFGVRVPVDLLISPAY
DLRHLAEHIEARRGAAADRPTFASVHGEGSTEVFAKDLTLDAFLDARTLADARALPRAEGEPRTVFLTGASGYLGRFLVLTWLERLAPVGGRLIALVRGKDAAAARGRLDA
AFDSGDPELLRTYGELAEGHLEVVAGDMADPRLGLDQETWDRLADEVDVIVHAGALVNHVLPYPELFEANVVGTAELVQLALTRRMKPFTYISSVAVATARPGEPALDEYT
DVRAALPVMEVDGGYAGGYATSKWAGEVLLREAHDLCGLPVTTFRSNMILAHSRYGGQLNVPDMFSRLLFSVLATGIAPRSFYRADGERAHYDGLPVDFTAAAVVALGGRG
GGPTAPQYRTFSLVNPNDDGVSLDRIVDWLAEEGHRIERVDDHADWYVRFEAAMRTLPETQRAYTALQILHGFREPEESVPGSVIPSERFRAVVRAGRVDGHQDIPSLGRE
LVAKYARDLKGLMGAPAGVGTRA
>Streptomyces_acidiscabies
MPAKPPADVSPLSRAARLVAELSAHDPQYRAAMPLPAVREAVREAARDQVLSRTVATVMAGYADRPALARRATEPVTDPVSGRTSLRRLPEFTTVTYGELWARAGAVSAEW
AADTRLPLAPGDFVALYGFTSGDYVTVDLACLRHGAVSVPLQSGAPVAGLAPILAETGPKVLAVSLELLDRAVELALSADTSPRLVVFDFHAGDDAQREAFEAASARLTAA
GHAAPLPLEEVIERGRALPPAPLFVPGPDEDPVRLLIYTSGSTGTPKGAIQTERMLHRAWAGAVPIPDDVASIVVNYLPLSHVAGRSSLVETLRRGGISYFTAHSDLSDLF
EDIALARPTALLFVPRVCDLLFQEYQAELARRAGEFADGEALDAAVKADLRERFVGGRLIQALYGSAPLSAELREFMRTCLDLPVLDGYGSTETGSVLLNTRVQRPPVTDI
KLVDVPELGYFATDSPYPRGELVLKSATLTPGYYRRPEVTAAAFDADGFYRTGDIMAEVGPDQYVYVDRRNNVVKLAQGEFVALSRLEGVYVTHPLIRQIYVYGNSERAHL
LAVIVPTRENLTHEDLSAALQQAARDAELNSYEIPRAFLVETEPFSLANGLLSDTRKNLPPRLKARYGERLEALYEELARDQEDAVRVLRDEGAGRPVSETVERAARALLG
SSAADARFTDLGGDSLSALSFSTLLAEIFGVEVPVGTVLSPANDLRALAAHIEARLASGVSRPTFASVHGVGSTVVRAGDLTLEKFIDAGTLAEAAQLPAPGSETPRTVLL
TGANGYLGRFMCLDWLERLADSGGRLVCVVRGKDDADARARLDAAFDSGDPELLRRYRELAAGRLDVLAGDIGAERLGLAGETWRRLAEDVDLVAHPAALVNHVLPYEQLF
GPNVVGTAELIRLALTARVKPFVYVSTTAISTTLDETSDIRESIPERALTDAYAAGYGTSKWAGEVLLREAHARFGLPVAVFRSDLILAHPHHTGQLNPADVLTRLLFSIL
STGLAPTSFYSEEGRAHFDGLPVDFTAEAINTLGAQPTSTHRTYNAVNPHDDGVSLDTFITWLEEAGHPLRRLPHTTWSPRLETALRSLPEHRRPHTLLPLLHAFATPQPP
TPTSPVPATHFHEAVREAGIGPDKDIPHITRNLITKYATDLRQLGLW
>Streptomyces_torulosus
MENVMVRYADRPAIGERAKEFVRDPGTGRVSYRLLPRYEKTSFGELWARVRAVASEWHMDGRRQLRPGDRVALLGFASGDYTVIDLACTYLGCVSVPLQTSSPASQQRAIT
DETESRLVAASVERLDRAVDLALESPSVERVLVFDVHPEVSDHADALDEARSRLKSAGRAVSVDSLPEVLRRGAGLPDAPLFVPADGDDPLAMLIYTSGSTGTPKGAMYTD
RLTSAMWGGAWSKLFSEGERVTFSYMPMSHVAGHSSLKNTLARGGITYFTAKSDLSTFFEDIALARPTELSLIPRICELIFQKYQSELDRRAVTSAADRAALEEQVRADMR
VELLGGRVAWAGCASAPLSAELTAFMESLLGLKLHNIYGSTEAAAVSVDGEMLSPPVIDYKLVDVPELGYYRTDSPHPRGELLLRTDALIPGYFNHPELTAELLDEDGYYR
TGDIVAEVAPGRHAIVDRRKSVLKLSQGEFVATARLEAIFAYSPLVRQTFVYGNSERSYLLAVVVPSADALTRFEGRTEELRQRLSDSFVQVAKEEGLNSYEIPREFLIET
EPFSQENGLLSDHRKLLRPRLLERYRERLEALYADIAARENEELLDVRRGAGERPVLETLQRAARALLAGSGAEVSPTARFRDLGGDSLTAVAFSDLLDDLFTVRVPVDLI
ISTATGLQQIADHIEAKRATGPDRPGFASVHGHGSVEVKAADLTLDKFIDPRTIAEAERLPSPAESPRTVLLTGASGYLGRFLCMEWLERLENTDGRLVCLVRGKDVAAAR
KRLEESFAGGDGQLARRFGTLAADRLEVVAGDMAEPQLGLGDRTWQRLSREVDVIVHAGALVNHVLPYNHLFEANVVGTAELVRLALTGRMKPFTYISSVAVATSRHPALG
EDPDVRQALPVQPVDAGYASGYATSKWAGEVLLREAHDLCGLPVTVFRSNMILAHSGYGGQLNVPDMFSRLLFSVLATGIAPSSFYRDEGKGKRPRAHYDGLPVDFTAAAV
VTLGGGNTSAYETFSLVNPHDDGISLDTFIDWLTDAGHRIERIDDYADWLDRFETAMRALPEEQRQHSSLPLIHGFKDPEIAVRGSDIPSERFREAVRAAKVGAEKDIPHI
TPSLIEKYARDLTSLMSA
>Streptomyces_europaeiscabiei
MAEPLDAAAVPAHDPGQGLAEVLASVEPGRALAEVMASVLESHGDRPALGERARDPETGRLLPHFDTISYRELWSRVRALAGRWHHDPAYPLGPGDRICTLGFTSTDYATL
DLACIHLGAVPVPLQSNAALPRLAPIVEESGPTVLAASVDRLDTAVDVVLASRTIRRLLVFDDGPGTTRPSGALAAARERLAGSPVTVDTLAELIDRGRDLPPPPLYTPDP
GEDPLALLIYTSGSTGAPKGAMYTQRLLGTAWYGFSYGAADTPAISVLYLPQSHLAGRYAVMGSLVKGGTGYFTAADDLSTLFEDIALVRPTELTMVPRLCDMLLQHYRSE
LERRSDEPGDIEAAVRKAVREDFLGGRVAKAFVGTAPLSAELTAFVESVLGFHLYTGYGSTEAGGVLLDTVVQRPPVTDYKLVDVPELGYYATDLPHPRGELLLKSHTLIP
GYYRRPDLTATIFDADGYYRTGDVFAETGPDRLVYVDRTKDTLKLSQGEFVAVSRLETVLLDSPLVQHLYLYGNSERAYLLAVVVPTPAALAGSGGDTEALRPLLMESLRS
VARRAGLNAYEIPRGILVEPEPFSAGNGLFTESHKLLRPRLKERYGPVLELLYDRLADGQDRRLRELRRTGADRPVPETVVRAAQALLGCLSSDLRPGAHFTDLGGDSLSA
VSFSELLKEIFHVDVPVGVIIGPAADLAEVARYITAARRPTGIRRPTFASVHGEHLTEVRAGDLVPEKFLDAPTLAAAPGLPRPDGDVRTVLLTGATGYLGRFLCLEWLER
LAPSGGRLICLVRGSDATVATRRLEAAFDSGDAALLRRYRKAAAKTLEVVAGDIGEPLLGLAEDTWRELAGTVDLIVHPAALVNHLLPYGELFGPNVVGTAEVIRLALTAR
LKPVNHVSTVAVCLGTPAETADENADIRATVPVRTIGQGYADGYATSKWAGEVLLREAHERYGLPVAVFRSDMVLAHRTYAGQVNVPDVLTRLLLSLVNTGIAPGSFYRTD
TRAHYDGLPVDFTAEAVVALGARVTEGHRTFNVLNPHDDGVCLDTFVDWLIEAGHPIRRIDDHGAWLTRFTAALRALPEKHRQHSLLPLIGAWAEPDEGAPGPLLPAERFH
AAVRAAGVGPERDIPRVSPDLIRKYVTDLRALGLLVDP
>Streptomyces_flavidovirens
MADTMERYADRPALGERATEFIREADTGRTRIGLLPRYEMTTFGDLWKRVRSVASEWHHRAEYGVRAGDRVAILGFASAEYTAIDLACAHLGAVSVPLQTSSPVPQLAAIV
EETGPVVVATSAERLDVAVELTLKSPSVRRLVLFDYHPEVEDQFDIFTSARDRLEQHGREVDIDLLSEVFKRGAELPRAPLFEPAGDEDPLAMLIYTSGSTGTPKGAMYTE
RLSRAMWGGAWSQLFSDEHAVNLHYMPMSHVAGHSSLKNTLSRGGTTYFTAKSDLSSFLEDMALARPTEMSLVPRVCEMLFQKYQSELDRHTGRTGDPELIEAEVKKDMRE
KVLGGRVGWASCGSAPLSAELKDFTESLLGIELHIIYGSTEAAAVSVDGRLLSPPVTDYKLADVPELGYYRTDLPHPRGELLLKSEAMVPGYYKRPDLNAEIFDEDGYYRT
GDIVAEIEPGRHAIVDRRKNVLKLSQGEFVATSGLEATFAVSPLVRQIFVYGNSERSYLLAVVVPTSDALDQFGGDEATLKNLLGESFQSIAKDIGLNSYEIPRDFLIETE
PFSQHNGLLSDHRKLLRPQLLEKYRQDLEEMYSDISARETNELREVRRTGMNRPVLETVQRAARALLSTSAVEVSPTARFREIGGDSMSAVTFSDLLHDIFGIRVPVDVII
SPGYDLQLLAGYIEGKKSAGVRRVDFASVHGAGSTEVHARDLTLDKFIDAETLAGARSLPRPVGAPRTVLLTGASGYLGRFLCLEWLKRLAPTGGKLICVVRGKDNASARS
RLDEAFSGGDDELTRAFQDLAAAHLEVVTGDMAEPQLGLSDQMWQQLADDVDLIVHAGALVNHILPYNHLFDANVVGTAELIRLGLTSRLKAFTYISSVAVAASLDGDQAL
DEDSDVREAIPDQPVDDGYASGYATSKWAGEVLLREAHDLCGLPVTTFRSNMILAHSTYPGQLNIPDMFTRLLLSLIITGIAPHSFYATDPAGKRQRAHYDGLPVDFTAEA
VVTLGGSALQGYQTFSLVNPHDDGISLDTFVDWLNEAGHRIDRVDEYDEWLARFETALRAAPEPQRQHSILPLLHGHRTPESAVTGSAIPSARFRAAVQAARLGPAGDIPQ
LSSAFIEKYATDLKELISPRTGRLAD
>Streptomyces_celluloflavus
MYATDPQFRGAAPLDAVTEAIRRPGLPLADLVDTVMEAYADRPALGERATEPVTDPVTGRTTLRLLARFDTITYGELWERVGALAAEWHHHPDHAVGPGDFVAVLGHPSAE
YTVVDLACVRSAAVSVPLQAGASAQQLAPIVAQTGPRLLAVDMAHLEVALRIAADAPSPVRIVVFGHRPEVTAHREQLDSARDRLAAQGRGVTLDTLAAGIERGRSLPPAP
RSREGSTADALSALIYTSGSTGTPKGAMYTERLVTRFWVDFVPGQEIRPSIVLNYLPLSHVMGRGALYGTLAKGGTAYFVASSDLSTLFEDLSLVRPTEFLMVPRICDMLF
QRYQSELIWWNAAGGDAAEQADRVKEELREKTLGGRLLWAVSASAPLSAEMSAFVESCFQVRMLDGYGSTEAGVVSLDGRLLRPPVTDHKLADVPELGYFRTDSPHPRGEL
LIKTDRLVPGYFRRPDATAQVFDEDGFYHTGDIMARTGPDELVYVDRRSHVLKLSQGEFVAVSRLEALFTGSPVVRQIFVYGNSARAYLLAVIVPTQDALDRVGGDTRLLG
PALRESLQLLATEAGLNSYEIPRDFLIETEPFSQRNGLLSGVRKLLRTALTKHYGERLEALYTELTERETDELRALRQAGPSRPVGETVCRAARALLGHRQGDLMPGTRFL
ELGGDSLSALSFSQLLKEIYDVDVPVDVVINPVNTLQQVADHIEKALASGHRRPTAESVHGPGATRLVAGDLTLNAFFDTEPLAQADRPADPLPQARTVLLTGANGYLGRF
LCLEWLERVAERGGTLVCLVRGGTDELARTRLDAAFDSGDPGLLRRYHDLAAEHLEVVAGDVGETGLGLGEETWQRLADTVDLIVHPAALVNHVLPYDQLFGPNVLGTAEL
IRLAVTSRVKQFTFLSTVAVVFGYEAAADETADIRTACATRDLDSGYADGYAAGKWAGEVLLREAHDRFGLPVAVFRSNLILAHPRYRGQLNIPDVFTRLVLSLLATGIAP
GSFYARGTGEGGGHYDGLPVDFTARAVASLGDDAREGYRTFNVVNPHEDGISLDTFVDWLVAAGHPLARIHDYDEWLHRFETALRGLPDGRRQHSLLPLLHAFARPQEPLP
GSALPADRFRAAVRAAAFNEENDIPCLSRDLITKYVTDLRAEGLL
>Streptomyces_rimosus
MYATDPQFRDAAPLDSVTEAIRRPGLPLADLVATVMEGYADRPALGERATEPVTDPDTGRTTLRLLERFDTITYGELWERVGAVASEWRHHPGHAVDRGDFVALLGPTSAE
YAVVDLACVRSGAVSVPLQAGASAEHLAPIVAQTGPRLLAVDMAHLDVALQLAAEAPSLGRIVVLGHRPDITTHREGLDSARDRLAAQGRGVTVDTLASVIERGRDLPPLP
RCPEGTTPDALSSLIYTSGSTGTPKGAMYTERLVRQFWVDFVPGQGVRPSIVLNYMPLSHMMGRGVLFGTLAKGGIAYFVASSDLSTLFEDLSLTRPTEFIMVPRISDMLF
QRYQAELARRSDTGAEAPGATADQAEHVQEDVQAELQAELRKELREKTLGGRLLWAVSASAPLSAEMTAFVENCLHVKLFNGYGSTEAGIVSLDGRVVRPPVTDHKLADVP
ELGYFRTDSPHPRGELLIKSDRLFSGYFQRPDATAQVFDEDGFYRTGDIMARTGPDTLVYVDRRSNVLKLSQGEFVATSRLEALFIGSPFVRQVFVYGNSTRAYLLAVIVP
TQDALDRAGEDTQRLRSILRESLQRLATEAGLNAYEIPRDFLIETEPFSQQNGLLSGVRKLLRPALTKRYGERLEALYTELAERGTDELQALRQAGPSQPIPETVLRAARA
LLGHRQGDVKPDTHFLELGGDSLSALSFSQLLKEIFHVDVPVDVLINPVNTLRQVADHIENALAGHRRPTADSVHGPGAKRLLASDLKLDAFLDTGTLAKTGRAAGPLPEA
RTVLLTGANGYLGRFLCLEWLERVAERGGTLVCVVRGSTDEAARARLDAAFDSGDAELLRHYRELAAEHLEVVAGDIGETDLGLGKETWQRLADTVDLIVHPAALVNHVLP

YDQQFGPNVLGTAELIRLALTYRVKQFTYLSTVAVVFGAEAAADETADIRTACAVRDLGGGYADGYAAGKWAGEVLLREAHETYGLPVAVFRSNMILAHRRYRGQLNIPDV
FTRLLLSLLATGIAPGSFYAGGAGADTGSGHYDGLPVDFTARAVAALGDGTREGYRTFNVVNPHEDGISLDTFVDWLTAAGHPLTRIHDYDAWLDRFETAMRGLPDRQRQH
SLLPLLHAFTKPEEPLPGSALPAQRFRAAVRAAALDGEADIPHLSQDLITKYVADLRAQHLL
>Streptomyces_griseus
MSVHDPQYRNAMPLQSVGTAVRDAGRDQVLSRTVATIMEGYAERPALAQRARERVTDPVSGRTSLRLLPEFTTISYGELWARAGAIAAEWAADTGRPLAPGDFVAIYGFTS
CDYVTLDLACLRLGAVSVPLQSGAPVSQLAQIVAETGPRVLATSIELLDRAVELVLPAESAPRLVVFDYHPEDDAEREAFDAARTRLAVSGHAAPDTLTAVIGRGYDLPPV
PLFIPGPYDDPTRLLIYTSGSTGTPKGAIYTERMLHRSWAGSVPIPDSVASIVVNYLPLSHVAGRSSLVETLRRGGISYFTARSDLSSLFEDIALARPTALLMVPRICDML
FQEYQAERARRAAEFTDGEALDAAVKTDLRERFVGGRLLQALCGSAPLSAELREFVESCLDLPLLEGYGSTETGSVLLNTVVQRPPVLDCKLVDVPELGYYGTDSPYPRGE
LVLKSETITPGYYRRPDATAQAFDENGFYRTGDIMARIGPDRYMYVDRRNNVMKLAQGEFVALTRLEGVYVTSPLIRQIFVYGNSERAYLLAVIVPTEEALVRVAHPDDLK
ASLSEALQETARHAELNSYEIPRDFLIETEPFTLENGLLSDTRKNLPPRLRGRYGERLEALYATVEQEQDDAVRALRDGGPDQPVFETVSRAAQALLGCSTAELDAATHFT
DLGGDSLSAMSFATLLTEIFGVEVPVGAVLSSSNDLRALADHIESGRVSGAKPSTFATVHGAGSTEVRASDLTLDRFVDAETLAAARQLPQPEETTPRTVLLTGANGYLGR
FMCLDWLERLADTGGRLICVVRGRDEAAARARLDRAFDSGDAELLRRYRDLAARHLDVLAGDIGQENLGLSKATWQRLAAEVDLIAHPAALVNHMLSYEQLFGPNVVGTAE
VIRLAITTKIKPFIHLSTVAVAAPLDPSALDETGDIRELNPVRALDDTYAGGYGTSKWAGEVLLREAHDLCGLPVTTFRSDMILAHSRYTGQLNVPDVFTRLLFSLVRTGI
APTSFYRTEGLTGSGQAHFDGLPVDFTAEAVNALGAQAARGYRTFNVVNPHDDGISLDDFVTWLVEAGYPIRHLADYDDWIVRFETAIRALPEQQRQHSLLPLLHAFRRPE
EPLPGSAIPADRFRTAVREAGIGADKDIPHLTPELIVKYPADLRQRGLM
>Streptomyces_sp._NRRL_F-5135
MPLPAVDAAIGRPGLTLKKIIATAMEGYADRPALGERARELIRDPATGRAEYRLLPHFDTITYAQLWSRVEALAADLHHDPRQPLRADEFIAVLGFTSTDFVTIDLTCARL
GAVCVPLQSSASAARLGPVIAETGPRILAAGVEFLDTAVDCALDSGSVGRLIVFDHRPEADDERERFEAARQRLTDAGSTVVLESLADVLDRGRGLPPAPELEDGTDTGRL
ALLLYTSGSTGTPKGAMYTERLVGRMWHGFWPGKSSLPLIMLSYMPMSHLAGRATLYTVLGSGGTVCFTARSDLSTLFEDLGLVRPTDLLLVPRVCDMLLQHYRGELDRRT
AAGGDPAVLEAEVKRDLGERSLGGRLLWIGSGGAPLSDEMTDFVASCVDVPLHDGYGSTEAGGMLADHRPIRPAVRDYRLVDVPELGYFRSDHPHPRGELLIRTDALIPGY
YKRPDVMAGLVDEDGYYRTGDIFAERGPDELFYVDRRNNVLKLSQGEFVAVSRLEAVFAGSPLVRQIFVYGSSERAYLLAVVVPVPEAAERAGGDPAELKARIAASLRGTA
KEAGLNSYEIPRDFLIETEPFSTENGLLSDIRKLLRPRLTERYGERLERLYADLAARENDELRALRRSGRDRPVADTVVRAAQAVLGTPADPAARYTDLGGDSLSAVSFSQ
LLGEIFGVEVPVGVVISPAHDLRRLAEHVERALSSGDRRPSPATVHGAGATEVRARDLALDAFVDAETLAAAPSLPHVAGPARTVLLTGANGYLGRFLCLEWLERLARTGG
TLVCVVRGSDAAVARRRLYEAFDSGDPELLARFGELAEGRLEVLAGDIGEPDLGLDGPTWNRLADTVDLIVHPAALVNHVLPYEQLFGPNVVGTAELIRLALTRRVKRFTY
LSTVGVIAAQAATADESADIRVASPVRRLDDSYASGYATSKWAGEVLLREAHDLCGLPVATFRSDMILAHSRYGGQLNVPDVFTRLLLSLAATGIAPGSFYRAGPGGVRRP
AHYEGLPVDFTAEAVTALGERATEGHRTYNVLNPHDDGVSLDVFVDWLVDAGHPVRRIDDYDEWLARFGTAMRALPQEQRQHSLLPLLHAFAEPAEPVAGSAVPADAFRAA
VLEAGVGPDADIPHLSASLIGKYAADLRALKLI
>Streptomyces_sp._NRRL_WC-3773
MYATDPQFRDAAPLDAVTEAIRRPGLPLADLVATVMEGYADRPALGERATEPVTDPDTGRTTLRLLDRFDTITYGELWERVGAVASEWRHHPSHAVGRGDFVALLGPTSAE
YTMVDLACVRSGAVSVPLQAGASAEYLTPIVAQTGPRLLAVDMAHLDVALQLAADAPSLGRIVVLGHRPEATTHQEWLDSARDRLAAQGRGVTVDTLASVIERGRNMPPLP
RCSEGTTPDALSSLIYTSGSTGTPKGALYTERLVRQFWVDFVPGQEVRPSIVLNYMPLSHMMGRGVLFGTLAKGGIAYFVASSDLSTLFEDLSLTRPTEFMMVPRISDMLF
QRYQAELARRRGTGAEAAGGTADQAGHEQEDLQAELREKVLGGRLLWAVSASAPLSAEMTAFVENCLHVRLFDGYGSTEAGIVSLDGRVVRPPVTDHKLADVPELGYFQTD
SPHPRGELLIKSDRLFSGYFQRPDATAQVFDEDGFYRTGDIMARTGPDTLVYVDRRSNVLKLSQGEFVATSRLEALFIGSPFVRQVFVYGKSTRAYLLAVIVPTQDALDRA
GEDTQRLRSILRDSLQRLAAEAGLNAYEIPRDFLIETEPFSQQNGLLSGVRKLLRPALTKRYGERLEALYTELAERGTDELQALRQAGPSQPVPETVLRAARALLGHRQGD
VKPGTHFLELGGDSLSALSFSQLLKEIFHVDVPVDVLINPVNPLRQVADHIENALAGHQRPTAASVHGPGATRILASDLKLDAFLDPGTIGTLAKTGRPAGPLPEARTVLL
TGANGYLGRFLCLEWLERVAERGGTLVCVVRGSTDEAARARLDAAFDSGDPDLLRHYRDLADDHLEVVAGDIGEADLGLGKETWQRLADTVDLIVHPAALVNHVLPYDQQF
GPNVLGTAELIRLALTSRVKQFTYLSTVAVVFGAEAAADETADIRTACGVRDLDGGYADGYAASKWAGEVLLREAHETYGLPVAVFRSNMILAHRRYRGQLNIPDVFTRLL
LSLLATGIAPGSFYAGGAGADTGSGHYDGLPVDFTARAIAALGDDVREGHRTFNVVNPHEDGISLDTFVDWLTAAGHSLTRIHDYDEWLDRFETALRGLPDHQKQHSLLPL
LHAFTKPEQPLPGSALPAQRFRAAVRAAALDGEADIPHLSQDLITKYVADLRAQHLL
>Mycobacterium_tuberculosis
MSINDQRLTRRVEDLYASDAQFAAASPNEAITQAIDQPGVALPQLIRMVMEGYADRPALGQRALRFVTDPDSGRTMVELLPRFETITYRELWARAGTLATALSAPAIRPGD
RVCVLGFNSVDYTTIDIALIRLGAVSVPLQTSAPVTGLRPIVTETEPTMIATSIDNLGDAVEVLAGHAPARLVVFDYHGKVDTHREAVEAARARLAGSVTIDTLAELIERG
RALPATPIADSADDALALLIYTSGSTGAPKGAMYRESQVMSFWRKSSGWFEPSGYPSITLNFMPMSHVGGRQVLYGTLSNGGTAYFVAKSDLSTLFEDLALVRPTELCFVP
RIWDMVFAEFHSEVDRRLVDGADRAALEAQVKAELRENVLGGRFVMALTGSAPISAEMTAWVESLLDVHLVEGYGSTEAGMVLNDGMVRRPAVIDYKLVDVPELGYFGTDQ
PYPRGELLVKTQTMFPGYYQRPDVTAEVFDPDGFYRTGDIMAKVGPDQFVYLDRRNNVLKLSQGEFIAVSKLEAVFGDSPLVRQIFIYGNSARAYPLAVVVPSGDALSRHG
IENLKPVISESLQEVARAAGLQSYEIPRDFIIETTPFTLENGLLTGIRKLARPQLKKFYGERLERLYTELADSQSNELRELRQSGPDAPVLPTLCRAAAALLGSTAADVRP
DAHFADLGGDSLSALSLANLLHEIFGVDVPVGVIVSPASDLRALADHIEAARTGVRRPSFASIHGRSATEVHASDLTLDKFIDAATLAAAPNLPAPSAQVRTVLLTGATGF
LGRYLALEWLDRMDLVNGKLICLVRARSDEEAQARLDATFDSGDPYLVRHYRELGAGRLEVLAGDKGEADLGLDRVTWQRLADTVDLIVDPAALVNHVLPYSQLFGPNAAG
TAELLRLALTGKRKPYIYTSTIAVGEQIPPEAFTEDADIRAISPTRRIDDSYANGYANSKWAGEVLLREAHEQCGLPVTVFRCDMILADTSYTGQLNLPDMFTRLMLSLAA
TGIAPGSFYELDAHGNRQRAHYDGLPVEFVAEAICTLGTHSPDRFVTYHVMNPYDDGIGLDEFVDWLNSSGCTIQRIADYGEWLQRFETSLRALPDRQRHASLLPLLHNYR
EPAKPICGSIAPTDQFRAAVQEAKIGPDKDIPHLTAAIIAKYISNLRLLGLL
>Mycobacterium_avium
MSTATHDERLDRRVHELIATDPQFAAAQPDPAITAALEQPGLRLPQIIRTVLDGYADRPALGQRVVEFVTDAKTGRTSAQLLPRFETITYGEVAQRVSALGRALSDDAVHP
GDRVCVLGFNSVDYATIDMALGAIGAVSVPLQTSAAISSLQPIVAETEPTLIASSVNQLSDAVQLITGAQAPTRLVVFDYHPQVDDQREAVQDAAARLSGTGVAVQTLAEL
LERGKDLPAVAEPPADEDSLALLIYTSGSTGAPKGAMYPQSNVGKMWRRGSKNWFGESAASITLNFMPMSHVMGRSILYGTLGNGGTAYFAARSDLSTLLEDLELVRPTEL
NFVPRIWETLYGEFQRQVERRLSEAGERRAVEAEVLAEQRQYLLGGRFTFAMTGSAPISPELRNWVESLLEMHLMDGYGSTEAGMVLFDGEIQRPPVVDYKLVDVPDLGYF
STDRPHPRGELLLRTENMFPGYYKRAETTAGVFDEDGYYRTGDVFAEIAPDRLVYVDRRNNVLKLAQGEFVTLAKLEAVFGNSPLIRQIYVYGNSAQPYLLAVVVPTEEAL
ASGDPETLKPKIADSLQQVAKEAGLQSYEVPRDFIIETTPFSLENGLLTGIRKLAWPKLKQHYGERLEQMYADLAAGQADELAELRRNGAQAPVLQTVSRAAGAMLGSAAS
DLSPDAHFTDLGGDSLSALTFGNLLREIFDVDVPVGVIVSPANDLAAIASYIEAERQGSKRPTFASVHGRDATVVRAADLTLDKFLDADTLAAAPNLPKPATEVRTVLLTG
ATGFLGRYLALEWLERMDMVDGKVIALVRARSDEEARARLDKTFDSGDPKLLAHYQQLAADHLEVIAGDKGEANLGLRQDVWQRLADTVDVIVDPAALVNHVLPYSELFGP
NALGTAELIRLALTSKQKPYTYVSTIGVGDQIEPGKFVENADIRQMSATRAINDSYANGYGNSKWAGEVLLREAHDLCGLPVAVFRCDMILADTTYAGQLNLPDMFTRLML
SLVATGIAPGSFYELDADGNRQRAHYDGLPVEFIAAAISTLGSQITTGFQTYHVMNPYDDGIGLDEYVDWLVDAGYSIERIADYSEWLRRFETSLRALPDRQRQYSLLPLL
HNYRTPEKPINGSIAPTDVFRAAVQEAKIGPDKDIPHVSPPVIVKYITDLQLLGLL
>Mycobacterium_colombiense
MSTAIHDEELDRRIEHLVATDPQFAATRPDPAITAATEQPGLRLPQIIRTVLDGYADRPALGQRVVEFVKDAKTGRTSAELLPRFETVTYGELGQRVSALGRAWASDSVSP
GDRVCVLGFNSVDYATIDIALGTIGAVSVPLQTSAALSSLQPIVAETEPSLIASSVNQLPDAVELILAGHVPGRLVVFDYQPEVDDQREAVESAVARLAGIGVVVEQLADV
LRRGKDLPPVPEQQTDEDSLALLIYTSGSTGAPKGAMYPQSNVGKMWRRGSKNWFGESAASITLNFMPMSHVMGRGILYGTLGNGGTAYFAARSDLSTLLEDLELVRPTEM
NFVPRIWETLYGEFQRQVERRLTDGADREAVEAEVLEEQRQYLLGGRFIFAMTGSAPTSPELKKWAESLLQMHLMDGYGSTEAGMVLFDGEIQRPPVIDYKLVDVPDLGYF
STDRPYPRGELLLRTENMFPGYYKRAETTAGVFDDDGYYRTGDVFAEIAPDRLVYVDRRNNVLKLAQGEFVTLAKLEAVFGNSPLIRQIYVYGNSAQPYLLAVVVPTEEAL
ADNDIEALKPKIADSLQKVAKETGLQSYEVPRDFIIETTPFTLENGLLTGIRKLAWPKLKQHYGERLEQMYADLAAGQANELAELRRSGAQAPVLQTVSRAAAAMLGAATG
DLSGDAHFTDLGGDSLSALTFGNLLREIFDVDVPVGVIVSPANDLAAIAAYIEAERQGSKRPTFAAVHGRGATTVRAGDLTLDKFLDEALLAGAPSLPKPSTEVRTVLLTG
ATGFLGRYLALDWLERMDMVDGKVIALVRARSDEEARARLDKTFDSGDPTLLAHYQELAADHLEVIAGDKGEANLGLDQQTWQRLADTVDIIVDPAALVNHVLPYSELFGP
NALGTAELIRIALTSKQKPYTYVSTIGVGDQIQPGKFVENADIRQISATREINDNYANGYGNSKWAGEVLLREAHDLCGLPVSVFRCDMILADTTYAGQLNLPDMFTRLML
SVVATGIAPRSFYELDAEGNRQRAHYDGLPVEFIAAAISTLGTQITTGFQTYHVMNPYDDGIGLDEYIDWLVEAGYSIERIPDYSEWLRRFETSLRALPDRQRQYSLLPLL
HNYQKPEKPINGSMAPTDVFRAAVQEAKIGPDKDIPHVSAPVIVKYITNLQLLGLL
>Mycobacterium_indicus_pranii

Supporting information

MSTAIHDERLDRRIEELIANDPQFAAARPDPAITAATEAPGLRLPQIIRTVLDGYADRPALAQRVAEFVTDAKTGRTTAELLPRFETITYGELGERVSALGRAWAGDAVRP
GDRVCVLGFNSVDYATIDIALGTIGAVSVPLQTSAAISSLQPIVAETEPSLVASSVNQLPDAVELILAGHVPGKLVVFDYQPQVDDQREAVEAAAARLADSGVAVEALADV
LRRGKDLPAVELPASDEDSLALLIYTSGSTGAPKGAMYPQSNVGKMWRRGSKNWFGESAASITLNFMPMSHVMGRGILYGTLGNGGTAYFAARSDLSTLLEDLELVRPTEM
NFVPRIWETLYGEFQRQVERRLADGEARETVEAAVLEEQRQYLLGGRFIFAMTGSAPTSPELKAWAESLLQMHLMDGYGSTEAGMVLFDGEIQRPPVIDYKLVDVPDLGYF
STDRPHPRGELLLRTENMFPGYYKRAETTANVFDEDGYYRTGDVFAEIAPDRLVYVDRRNNVLKLAQGEFVTLAKLEAVFGNSPLIRQIYVYGNSAQPYLLAVVVPTEEAL
ADNDLESLKAKIADSLQKVAKETGLQSYEVPRDFIIETTPFTLENGLLTGIRKLAWPKLKAHYGDRLEQMYAELAAGQANELAELRRSGAAAPVAQTVSRAAAALLGATAG
DLSADAHFTDLGGDSLSALTFGNLLREIFDVDVPVGVIVSPANDLAGIAAYIEAERQGSKRPTFAAVHGRGATTVHAGDLTLDKFLDEATLAAAPSLPTPATEVRTVLLTG
ATGFLGRYLALDWLERMDMVDGKVIALVRARTDEEARARLDKTFDSGDPKLLAHYQRLAADHLEVIAGDKGEANLGLDPQTWQRLAEEVDVIVDPAALVNHVLPYSELFGP
NALGTAELIRIALTSRQKPYTYVSTIGVGDQIQPGEFVENADIRQISATREINDGYANGYGNSKWAGEVLLREAHDLCGLPVTVFRCDMILADTTYAGQLNLPDMFTRLML
SLVATGIAPGSFYELDTDGNRQRAHYDGLPVEFIAAAISTLGTQITTGFQTYHVMNPYDDGIGLDEYIDWLIEAGYSIERIADYSEWLRRFETSLRALPDRQRQYSLLPLL
HNYQKPEKPINGSMAPTDVFRAAVQEAKIGPDKDIPHVSAPVIVKYITDLELLGLL
>Mycobacterium_kansasii
MSTTTRDERLERRIDTLIHDDAQFAAAKPDPAIAAALEKPGLSLPEIIQTALQGYADRPALGQRAVEFVTDTQTGRTSVRLLTRFETITYRQLGDRVGALARALTHDSVHA
GDRVCVLGFNSLDYTTIDMALAKVGAVSVPLQTSAAVTQLQPIVAETEPTMMAASVNQLSDAVDVLLSGHLPAKLVVFDYHPEVDDQREALDTARERLADTAVVVQTLKDV
LDHGATLAAGPLAASGDNDSLALLIYTSGSTGAPKGAMYRQSNVGKMWRRSSKNWFGPTAASITLNFMPMSHIMGRGVLYGTLGNGGTAYFAARSDLSTLLEDLRLVRPTE
LNFVPRIWETLYGEYQRAVDQRSVDPAAREAVEAQVMAEQRQDLLGGRYIFAMTGSAPMSPELRNWVEALLEIPLLDGYGSTEAGMVMFDGEIQRPPVIDYKLVDVPDLGY
FSTDQPYPRGELLLKTENMFPGYYKRPEVTASVFDADGYYRTGDVVAEVAPDRLVYVDRRNNVLKLAQGEFVTVAKLEAVFGNSPLVRQIYVYGNSAHPYLLAVVVPTEEA
SAGTDIAALKPLIADSLQTVAKEAGLQSYEVPRDFLIETTPFTLENGLLTGIRKLAWPKLRQHYGERLEQLYTELAASQANELSELRRSGAHAPVLETVSRAAGALLGAAS
TALSPDAHFTDLGGDSLSALTFGNLLREIFDVDVPVGVIVSPASDLAAIAAYIEGERQGSKRPTFAVIHGRDALEVHASDLTLDKFIDASTLAAAPVLPPPSAAVRTVLLT
GATGFLGRYLALDWLERMDLVDGKVIALVRAKSDDDARARLDKTFDSGDPELLTHYRRLATDHLEVIAGDKGEANLGLDQLTWQRLADTVDLIVDPAALVNHVLPYSELFG
PNALGTAELIRIALTGKLKPYTYVSTIGVGDQIEPGKFTEDADIRHISATRKINDSYANGYGNSKWAGEVLLREAHDLCGLPVAVFRCDMILADTTWAGQLNVPDMFTRMM
LSLVATGIAPGSFYELDADGNRQRAHYDGLPVEFIAEAIATLGARDGKGFQTYHVMNPYDDGIGMDRFVDWLVDAGCAIHRIDDYGDWLRRFETALRGLPEKQRHASLLPL
LHNYQKPAPPLRGSMAPTDRFRAAVQDAKVGPDKDIPHISPQIIAKYLSDLRLLGLL
>Mycobacterium_ulcerans
MSPITREERLERRIQDLYANDPQFAAAKPVTAITAAIERPGLPLPQIIETVMTGYADRPALAQRSVEFVTDAGTGHTTLRLLPHFETISYGELWDRISALADVLSTQTVKP
SDRVCLLGFNSVDYATIDMTLARLGAVAVPLQTSAAITQLQPIVAETQPTMIAASVDALADATELALSGQTATRVLVFDHHRQVDAHRAAVESARERLAGSAVVETLAEAI
ARGDVPRSAPGTDVSDDSLALLIYTSGSTGAPKGAMYPRRNVATFWRKRTWFEGGYEPSITLNFMPMSHVMGRQILYGTLCNGGTAYFVVKSDLSTLFEDLALVRPTELTF
VPRVWDMVFDEFQSEVDRRLVDGADRVALEAQVKAEIRNDVLGGRYTSALTGSAPISDEMKAWVEELLDMHLVEGYGSTEAGMILIDGAIRRPAVLDYKLVDVPDLGYFLT
DRPHPRGELLVKTDSLFPGYYQRAEVTADVFDADGFYRTGDIMAEVGPEQFVYLDRRNNVLKLSQGEFVTVSKLEAVFGDSPLVRQIYIYGNSARAYLLAVIVPTQEALDA
VPVEELKARLGDSLQEVAKAAGLQSYEIPRDFIIETTPWTLQNGLLTGIRKLARPQLKKHYGELLEQIYTDLAHGQADELRSLRQSGADAPVLVTVCRAAAALLGGSASDV
QPDAHFTDLGGDSLSALSFTNLLHEIFDIDVPVGVIVSPANDLQALADYVEAARKPGSSRPTFASVHGASVTEVHAGDLSLDKFIDAATLAEAPRLPAANTQVRTVLLTGA
TGFLGRYLALEWLERMDLVDGKLICLVRAKSDTEARARLEKTFDSGAPELLAHYRALAGDHLEVLAGDKGEADLGLDRQTWQRLADTVDLIVDPAALVNHVLPYSQLFGPN
ALGTAELLRLALTSKIKPYSYTSTIGVADQIPPSAFTEDADIRVISATRAVDDSYANGYSNSKWAGEVLLREAHVLCGLPVAVFRCDMILADTTWAGQLNVPDMFTRMILS
LAATGIAPGSFYELAADGARQRAHYDGLPVEFIAEAISTLGAQSQDGFHTYHVMNPYDDGIGLDEFVDWLNESGCPIQRIADYGDWLQRFETALRALPDRQRHSSLLPLLH
NYRQPERPVRGSIAPTDRFRAAVQEAKIGPDKDIPHVGAPIIVKYVSDLRLLGLL
>Mycobacterium_genavense
MTSDNRDERTARRVAELFNNDPQFRAAAPLPEVIEAACAPGLRLTEVLARLVEGYADRPALGERVRELVTDADGRTVLRLRPRFETISYRDVWDRVRAIATAWSSDPVTAG
DVVATVGFSSADYLVVDLVCAYLGLVTVPLQHNAPPARLRPIIEECEPKIVAVSAEYLDLAAESALTSTSLRQLMVFDYRAEVDEQRENFEQTRVRLQGSGTRVAVTTVDE
VVARGRRLPAVAACADGDDQRLAMIMYTSGSTGTPKGAMYTERTVTTVWTTMRFLAPGLPVINANFMPLNHLGGRLPLASAFLSGGTSYFVPESDLSTLFEDWALVRPTEA
AMVPRVVEMLYQHYRGAVDRGIAEGADPATAEHDAATEMREQVLGGRVLGGFVGSAPLATEMKAFLDSMLDAHITDGYGLTETGMLTRDNVVNRNRVIDYKLIDVPELGYF
LTDRPYPRGELLVKTDTMTPGYYKRPEVTAQVFDEDGFYKTGDVMAEIEPDHLVYVDRRNNVLKLAQGEFVAVANLESIYAGAPLVRQIFVYGNSERSNLLAVIVPTPEAL
AEGNSPALKTAIHQSLRQTAAAAQLQSYELPVDFILETKPFTDENGLLSGLGKQLRPRLKERYGEQLERLYSEIAAAQVDEIRVLREAAADRPAVETLAGACRALLGTSGA
DSESHFTDLGGDSLSALTLSRLLEDIFGVEVPVAVITSPANNIAKIAEYIDVQRGAGIRRPGFSTVHGHGATCIAAADLTLDKFIDAQTLCTAPSLPHAGGAAHTVLLTGA
NGWLGRFLTLEWLDRLAERGGKLVTIVRGRDVDEARARLEKAFDSGDPELLSRFRELAATHLEVLAGDIGEENLGLSPATWHRLAETVDLIVHPAALVNHVLPYDQLFGPN
VVGTAEIIRLAITSQIKPVTYLSTIAVASTVPPGQFQEDGDIRRVSPDRPLNGDYANGYANSKWAGEVLLREANDLCGLPVAVFRSDMILAHTRYRGQLNVPDMFTRLIFS
LLVTGIAPHSFYEHDGVGSRARAHYDGLPVDFIAEAITTIGSRIRKGYTSFDVMNPYDDGVSLDVFVDWLIRAGNKIQRIPDYDEWLARFQTALTGLPERQRQQSVLPLLH
AFHRPEKATRGACAPTEVFRAAVRADEIGPDKDIPHISAELIDKYADDLRQLALL
>Mycobacterium_parascrofulaceum1
MSTIDHDERLERRIEELTANDPQFAAARPDPAIEAALEKPGLRLPQVIRTVLAGYADRPALAHRAVEFVADSASGRTTLELLPRFETLTYRDLGDRVGALGRAWAHDEVRV
GDRVCILGFNSVDYATIDMALATISAVSVPLQTSASLTSLQPIVAETEPTVIAASANQLPDAVELILTGQRPAKLVVFDYHPEVDDEREAVETARTRLADTGVVVETLAEV
LERGKALPDTELPAGDEPDPLALLIYTSGSTGAPKGAMYPQSNVGKIWRRGSRNWFGESAASITLNFMPMSHVMGRGILYGTLGNGGTAYFAAKSDLSTLLEDLELVRPTE
LNFVPRIWETLFGEFQRQVARRLSEGGDRAAVEAEVLAEQREYLLGGRFIFAMTGSAPTSPELRNWVESLLQMHLMDGYGSTEAGMVLFDGEIQRPPVIDYKLVDVPDLGY
FGTDRPHPRGELLLRTENMFPGYYKRAEITANVFDEDGYYRTGDVFAEVAPDKLVYVDRRNNVLKLAQGEFVTLAKLEAEFGNSPLVRQIYVYGNSSQPYLLAVVVPTQEA
LGRWDSEALKGKIADSLQNVARQAGLQSYEVPRDFLIETTPFSLENGLLTGIRKLAWPKLKQHYGERLEQLYAELAEGQANELAELRRNGADAPVLQTVSRAAAAMLGTAA
TELTPDAHFTDLGGDSLSALTFGNLLREIFDIDVPVGVIVSPASDLQAIADYIEGERRGSKRPTFAAVHGRDATEVRAADLTLDKFLEAETLAAAPSLPKPTAEVRTVLLT
GATGFLGRYLALEWLERMDLVDGRVIALVRAKSDDEARARLDRTFDSGDPKLLAHYRELAADHLEVIAGDKGEPNLGLDQQTWQRLADTVDLIVDPAALVNHVLPYSELFG
PNALGTAELIRIALTTKLKPYTYVSTIGVGDQITPGQFVEDADIRQVSATRAVNDNYANGYGNSKWAGEVLLREAHDLCGLPVAVFRCDMILADTTYAGQLNLPDMFTRLM
LSLVATGVAPGSFYELDADGNRQRSHYDGLPVEFIAAAISTLGTQVLDGFQTYHVMNPYDDGIGLDEYVDWLIDAGHRIQRIADYGEWLRRFEGTMRGLPERQRQYSLLPL
LHNYQQPEKPINGSMAPTDRFRAAVQEAKIGPDKDIPHVSPPIIVKYATDLQLLGLL
>Mycobacterium_parascrofulaceum2
MTGDAKRARVADRIRQLDATDEQFRSTKPDAALQRAARQPELRLTQILQTLVEGYADRPALGWRARSSVTDPATGRTSARLLPRFDTISYRDLWANVGAIASAWRRDPVTP
GDFVATVGFASAEYLTVDLVCGYLGLVAVPLQHNGAASRLQPIVAEVEPRVLAAGAGYLDLAVEAALGSASLRRLVVFDYSPQIDDQREALERARAKLSAAGLTVTIETFD
ELVERGRGLPPEPAYTGDTDERLAMIMYTSGSTGLPKGAMYTERMLSKLWTNELMPDFADTPVLNVNFMPLNHLGGRIPLSTAFQAGGTSYFVPESDLSTLFEDWNLVRPT
EMGLVPRVAEMLYQRYQSAVDRRVSQGADPHTADAEARAELREWVLGGRIVTAFCGTAPLAAEMRGFVETCLDVHVLDGYGLTEVGMVTKDGRIAKPPVLGYKLVDVPELG
YFLTDKPFPRGELLVKSLTATPGYFKRPDVTANAFDPDGYYRTGDVMAELEPDRLAYVDRRNNVLKLAQGEFVAVARLEAVFSSAALVRQIFVYGNSERPYLLAVIVPTVD
ALDRGDHDGLKAALGESLRHAGRLAELQSYEVPADFLVEAEPFSEDNGLLSGVGKLLRPKLKERYGDRLERLYADLAANRVTELRALRDRAADRPVIDTLARAAEALLGLA
GGPPEPGALFTELGGDSLSALTFSNLLQDIFDVEVPVGMIVGPATDLRQLADFVDSERKSGSRRPTFATVHGREATEVRAADLTLDKFIDATTLAAAPALPRATGTPRTVL
LTGANGYLGRFLCLEWLERLAETGGRLVCVVRGADAAAAAERLEQVYRSGDPRLVERFRELAAGHLEVVAGDIGQPNLGLDGATWGALARSVDLIVHPAALVNHVLPYDQL
FGPNVVGTAEIIRLAITERIKPVTYLSTVAVAMSVDPAQFTEDGDIRAISPVRPIGDSYANGYANSKWAGEVLLREAHDLCELPVAVFRSDMILAHSRYAGQLNVPDAFTR
LIFSLLVTGIAPSSFYEATDGGDRPIAHYDGLPADFVAEAVTALGEQTATVFRSFDVMNPHDDGISLDVFVDWLIADGHDVTRIDDYDEWLGRFGTALRALPDGQRQQSVL
PLLDAYRKPERPLRGAPAPTDVFRNAVRSAKLGADNDIPHLSADLIAKYVVDLRLLGLL
>Mycobacterium_leprae
MWRTKQEKQLARRVDDLTANDPQFAAAKPDPAVAAALAQPGLRLPQIIQTALDGYAERPALGQRVAEFTKDPKTGRTSMELLPSFETITYRQLGDRVGALARAWRHDLLHA
GYRVCVLGFNSVDYAIIDMALGVIGAVAVPLQTSAAITQLQSIVTETEPSMIATSVNQLPDTVELILSGQAPAKLVVFDYHPEVDEQHDAVATARARLADSSVVVESLTEV
LGRGKTLPATPIPVADSADPLALLIYTSGSTGAPKGAMYLQSNVGKMWRRSDGNWFGPTAASITLNFMPMSHVMGRGILYGTLGNGGTAYFAARSDLSTLLEDLKLVRPTE
LNFVPRIWETLYDESKRAVDRRLANSADRAAIKAEVMDEQRQSLLGGRYIAAMTGSAPTSPELKHGVESLLEMHLLEGYGSTEAGMVLFDGEVQRPPVIDYKLVDVPDLGY

FSTDQPYPRGELLLKTQNMFPGYYKRPEVTATVFDSDGYYQTGDIVAEVGPDRLVYVDRRNNVLKLAQGQFVTVAKLEAAFSNSPLVRQIYIYGNSAHPYLLAVVVPTEDA
LATNDIEVLKPLIIDSLQKVAKEADLQSYEVPRDLIVETTPFSLENGLLTGIRKLAWPKLKQHYGARLEQLYADLVEGQANALHVLKQSVANAPVLQTVSRAVGTILGVAT
TDLPSNAHFTDLGGDSLSALTFGSLLRELFDIDVPVGVIVSPVNNLVAIADYIERERQGTKRPTFIAIHGRDAGKVHASDLTLDKFIDVSTLTAAPVLAQPGTEVRTVLLT
GATGFLGRYLALKWLERMDLVEGKVIALVRAKSNEDARARLDKTFDSGDPKLLAHYQELATDHLEVIAGDKGEVDLELDRQTWRRLADTVDLIVDPAALVNHVLPYSELFG
PNTLGTAELIRIALTSKQKPYIYVSTIGVGNQIEPAKFTEDSDIRVISPTRNINNNYANGYGNSKWAGEVLLREAHDLCGLPVTVFRCDMILADTSYAGQLNVPDMFTRMM
LSLAATGIAPGSFYELDAESNRQRAHYDGLPVEFIAEAISTLGDQSLDGFTTYHVMNPHDDGIGMDEFVDWLIDAGCPIQRINDYDEWLRRFEISLRALPERQRHSSLLPL
LHNYQKPEKPLHGSLAPTIRFRTAVQNANIGQDKDIPHISPAIIAKYVSDLQLLGLV
>Mycobacterium_intracellulare
MTGDVKRERVAARIRELAATDEQFRNAQPDLSLQQAARQPGLRLPQILELFVEGYADRPAVGWRAKTLSTDPATGRTTTRLLPRFDTMTYRELWADVRAIAAAWRHDPVSP
GDFVATVGFASAEYLTLDLVCGYLGLVAVPLQHNTTPSRLRPIVDEVEPSILAAGVGYLDLAVEAASGSSSLRRLVVFDYQPEVDEQREALQRAQATLAAAGAAVTIETLD
EIIERGRALPPEPMYTGDTDQRLAMIMYTSGSTGLPKGAMYTEQMLAKVWTNELMPDFADTPVFNVNFMPLNHLGGRIPLSTAFQAGGTSYFVPESDLSTLFDDWNLVRPT
EMGLVPRVAEMLYQRYQSAVDRLVASGADAGSAEARARAELREQVLGGRIVTAFCGTAPLAAEMRAFVETCLDVHVLDGYGLTEVGMVTKDGRMTRPPVLDYKLIDVPELG
YFHTDKPYPRGELLVKSLTATPGYFKRPDVTANAFDPDGYYRTGDVMAELEPDRLAYVDRRNNVLKLAQGEFVAVARLEAVFASAPLIRQIFVYGNSERPYLLAVVVPTAD
AAERTDPEGLKAAVAESLRQSAQLAELQSYEVPVDFIVETEPFSEDNGLLSGVGKLLRPKLKERYADRLEQLYAELAENRVTELRALREGADKHPVVFTLTRAAEALLGVA
GGPPAPDALFIELGGDSLSALTFSNLLRDIFDVDVPVGMITGPATDLGQLAEYVESERKSGSRRPTFATVHGRDAAEVRAAELTLDKFIDATTLAAAPNLPRATGTPHTVL
LTGANGYLGRFLALEWLERLAETGGKLVSIVRATDTATAGKRLEAVFDSGDPQLLERFRTLAAEHLEVIVGDIGEPNLGLDQSTWQRLAQSVDLIVHPAALVNHVLPYDQL
FGPNVVGTAELIRLAITTRIKPVTYLSTVAVAMTVDPGEFAEDGDIRAVSAVRPIDDSYANGYANSKWAGEVLLREAHDLCGLPVAVFRSDMILAHSRYAGQLNVPDAFTR
LMFSLLTTGIAPTTFYRTDEHGNRAVAHYDGLPADFVAEAVTTLGEQMAAECRSYDVMNPHDDGVSLDVFVDWLIAAGHDIRRIEDYDEWLGRFTTALRALPDKQRQHSVL
PLLDAYREPAAPLRGAPAPTDVFRHAVRTAKIGADEDIPHLSAALIDKYVADLRLLGLV
>Mycobacterium_tusciae
MSADTREERLERRIANLYVTDQQFADAKPNPEISAAIEQPGLPLADAVRAVMEGYSDRPAVGQRAVEFVTDTQSGRTTANVLPHFETISYGELWSRVGALTNALSDVLPGD
RICILGFTSADYTVIDLATVTLGAVSVPLQTSAPTTALRPIVTETEPVVIASSIDYIDDAVELVLTGHTPDRLIVFDFHPQIDEHREAYDAARARLTEAGSPTVVETLSEV
VERGHALPAASAAQRADDEIALLIYTSGSTGAPKGAIYTRSLAAKMWRPAAWGADSPDAFITLNFMPMSHVMGRASLYGTLAHGGTAYFAAKSDLSTFLEDLALVRPTQLN
FVPRVWEMLSAEVHSQLNRRLPEGADGATEAEIAADVRSHLLGDRYITVVTGSAPTSPELTQWVESFLDMHLVDGYGSTEAGGVLADGQVRRPPVLDYKLVDVPELGYFST
DRPHPRGELLVKTTNMFPGYYKRPEVTAEVFDADGFYRTGDIVVELEPERLQYVDRRNFVLKLSQGEFVTASKLEAVFQNSPLVRQIYIYGNSARSYLLAVVVPVADAAAQ
GDTEELHRSINDSLQDVAKAAGLQAFEIPRDFIIEPTPFTLENGRLTGIRKLARPKLKEIYGPRLEQLYTDLAEGQATELRELRQNAGRRPVVETVSRAAAALLGAAASEV
PADAQFTDLGGDSLSALTFANLLREIFDVDVPVGVIVSPANDLAALADYIEGERAGNTRPTFASVHGRGATEAHAHDLTLDKFLDANTLASAPALPYPSQEVRTVLLTGAT
GFLGRYLTLDWLERMNLVGGKVICLVRAKDDATARQRLDATFDSGDAELLSHYRRLAAEHLEVLAGDKGEAGLGLDDRTWQRLAHTVDLIVDPAALVNHVLPYDQLFGPNV
VGTAELIRLALTTKLKPFIYLSTVGVGDQIEPSKFVEDADVRQMSATRAVNDSYANGYGNSKWAGEVLLREAHDLSGLPVAVFRCDMILAEPRYAGQLNVPDMFTRLMLSL
VATGIAPGSFYELDAAGNRQRAHYDGLPVDFIADAISILGAHQVHGFETYHVMNPYDDGIGLDEYVDWLVEAGYPIDRVADYGEWLQRFETSMRALPDRQRQASLLPLLHS
YAQPAKPVLGSFASTERFRAAVQEAKVGPDDIPHVSAEIIVKYITDLQLLGLL
>Mycobacterium_xenopi
MDTREDQLERRIAALTANDPQFAAARPDEAVATAVQRPGLRLPEVIETVLQGYADRPALGQRAVEFVKDPNTGRTSAHLLPRFDTITYRELADRVGALASAWAREAVSPGD
RVAILGFTSVDYTTIDVTLARIGAVSVPLQTSAALAQLRPIVVETEPTVIAASVDYLSDAVELIRTGHAPARLVVFDHHPEVDDHREALDAARGRLAGHAVIVETLAEVLE
RGTSLPAPTVAAEDNDLALLIYTSGSTGAPKGAMYPQRNVAKMWQRSSRNWFGPSAASITLNFMPMSHVMGRGILYGTLGNGGTAYFGATSDLSTLLEDLTLVRPTELNFV
PRVWDTLHAEFLTRVDRLTAEGADRASAEALVMGDLRDNLLGGRAIFAMTGSAPISSQLKTWVESLLGIHLLDGYGSTEAGMVLYDGVVQRPPVIDYKLADVPDLGYFSTD
RPFPRGELLLKTENMFPGYYKRPEITAGVFDDDGYYRTGDVVAEVGPDRLVYVDRRNNVLKLAQGEFVTVAKLEAGFNNSPLVRQIYIYGNSAHPYLLAVVVPTDVNASKS
AIAESLQRVAKDAGLQSYEVPRDFLIEPEPFTLENGLLTGIRKLAWPKLKERYGERLEQLYAELDRSQADELSELRRSGAQRPVLETVTRAAGALLGAAASELQPDAHFTD
LGGDSLSALTFGNLLREIFDVDVPVGVIVSPASDLQAIAGYIEAERQGSKRPTFASVHGREAVEVRARDLRLDKFLDARTLEYVPALPGPSTELRTVLLTGATGFLGRYLA
LEWLERMDAVDGTVIALVRAKDDAAARERLDRTFDSDPKLRAHYRALAADHLEVVAGDKGEANLGLSQQVWQRLADTVDVIVDPAALVNHVLPYSELFGPNVLGTAELIRL
ALTTKIKPYTYVSTIGVGDQIEPGKFTEDADIRVISPTRRISDSYANGYGNSKWAGEVLLREAHDRCGLPVAVFRCDMILADTTYAGQLNLPDMFTRLMLSLAATGIAPRS
FYELDAEGNRQRAHYDGLPVEFIAKAVSTLGAQTVEGYQTYHVMNPHDDGIGLDEYVDWLIEAGYPIRRVDDYADWLQRFETAMRALPDRQRRYSLLPLLHNYQKPEKPMR
GSMAPTDRFRAAVQEAKIGPDKDIPHVTREVIVKYATDLQLLGLL
>Mycobacterium_abscessus
MTAGTAARVAKLFESDPQFRAAMPDPAVMDSLLAPGLRLSQVLHALLSGYAERPVMGFRSRESVVDTATGRTVDRLLPAFETITYGQLLEDISAILAEWQHGPMGAGDFIA
TIGFSSPDYVTLDLATLMNGSVSIPLQHNTSVVQLRMMLEETSPRLVAASADCLDLAVEAAVGLTDLRRVVVFDYRAATDDHREKLATARERLHAAGMDVVVEPLAEVIGR
GRDLPEPVLYTAGDDQRTALIMYTSGSTGAPKGAMFTEWTVTRFWSSGAAPNRDTPIINVNFLPLNHLAGRVGLLTAFIPGGTCYFVPESDLSTLFEDWQLARPTHMGVVP
RVVDMLFQHYQTRVDALMAGGTDVDTADRLAKTELREDVLGGRVVAGMLATAPLSPEMKAFLESSLDFHLLDLYGLTEVGGVFRDGKISRPPVLDYKLVDVPELGYYTTDK
PHPRGELLVKSATATPGYYKRPDVTAEVFDADGYYRTGDVMAEVAPDQLVYVDRRNNVIKLAQGEFVAVANLETVYVGAPLVRQIFVYGNSERAYLLAVVVPTEEALRAPD
PVELKNSIRESLQRTARSNHLHSYELPADFIIETTPFTIESGMLAAVGKPIRPKMIEHYGDRLEQLYVDLAEARVQELRQLRDTAQQRPVLDTVTEAAQALLGMSADAVRP
DHHFIDLGGDSLSALTFSNLLRDLFDVEVPVGVITGPAADLRKLAAYIQHEREHSTATAASVHGLDTTVISAAELTLDKFIDAETLYNASQLDVPAGTVATVLLTGANGYL
GRFLCLEWLQRLSQTDGQLICLVRGDNDDQALARLVAAYGDTDRTLLEEFHTLARRHLRVVAADIAQPRFGVDDATWEQLARDVDKIVHPAALVNHVLPYNQLFGPNVFGT
AEVIRLALTTRIKPVTYLSTMAVAMTVPDFDEDGDIRTVSPTRHIDPGYANGYANSKWAGEVLLREAHDLCGLPVSVFRSDMILTHRRYSGQLNVTDAFTRMLLSLVLTGI
APRSFYQGDGSGARPRAHYEGLPVDFVAEAITSLGLSSSEGFRSYDVMNPHDDGISVDTFVDWLVEDGHSIDIIDNYDEWLSRFETALRGLPDEQRRASVLPLLDAYRVPG
NPRRAAATPNHVFRKAVQESNIGGDADIPQIDRALIAKYIADLRAHGLL
>Mycobacterium_fortuitum1
MSFDTRDEQLATRIADLTATDPQFAAAIPSDTVTASVDVPGLLLPEIVQRVLEGYAERPALGERALEFVADPATGRTTARLLPRFDTISYGQVWDRVRALAAALHASGVAA
GDRVAILGFTSADYTVIDTALGQIGAVSVPLQTSSSPEALAPIVTETEPRVIAASVDHLADAVELALTAHAPAQLVVFDHHPEIDDHREAVASAAERITAAGASIAVDTLA
GLLDRGSNLPAPEAPKADGSDPLALLIYTSGSTGAPKGAMYLQSAVAKFWRRNSKAWLGPVSSAINLSFMPMSHVMGRGILYASLAAGGTCYFAARSDLSTLLEDLALTRP
TELNFVPRVWEMIHSEYQSRVDQRLAEGRDREAVEAEVLAEVRDKVLGGRFVAAMTGSAPISAELKTWTQDMLGIHLLEGYGSTEAGMALFDGVVQRPPVIDYKLVDVPDL
GYFGTDQPHPRGELLIKTENLFPGYYKRPEVTASVFDEDGFYRTGDVVAEIGPDQLRYVDRRNNVLKLAQGEFVTLAKLEAVFGNSPLVQQIYVYGNSAQPYLLAVVVPTD
PSVSKEAIAESLQEVAREADLQSYEIPRDFIVETTPFSLENGLLTGIRKLAWPKLKAHYGERLEQLYAELAETQAAELRELRSASADAPVVETVSRAAGALLGAAASDLGP
DAHFTDLGGDSLSALTFGNLLREIFDVDVPVGVIVSPATDLAGIAEYIETQRSGSKRPTYASVHGRHAAEVSAADLTLDKFLDADTLAAAPNLPKAGSEVRTVLLTGATGF
LGRYLALEWLERMDLVDGKVIALVRAKSDEEARTRLDATFDSGDAKLLAHYQNLAADHLEVVAGDKGEENLGLDQQTWQRLADEVDLIVDPAALVNHVLPYSELFGPNALG
TAELIKIALTTKIKPYTYVSTIGVGDQIEPGKFVENVDVREMSAVRKINDGYANGYGNSKWAGEVLLREANDLCGLPVAVFRCDMILADTSYSGQLNLPDMFTRMMLSLVA
SGIAPKSFYELDSEGNRQRSHYDGLPVEFIAESISTLGGQSVESFETYHVMNPYDDGLGMDEFVDWLIEAGYPIERIEDYGQWVQRFESTLRALPDKQRQASLLPLLHNYQ
KPERPMLGALAPTDHFRAAVQEAKIGPDKDIPHVSPAVIVKYITDLQQLGLL
>Mycobacterium_fortuitum2
MTTETREDRLQRRIAHLYEADSQFAAARPSEAVNTAVAEPELRLPAVVKGVFAGYADRPALGQRAVEYVTDADGRTSAQLLPRFDTITYRQLGDRVQAVTNAWHNHPVKPG
DRVAILGFTSVDYTTVDTALIELGAVSVPLQTSAPVTTLRPIVAETEPTVIAASIDFLDDAVELVKSGPAPRRLVVFDYRPRVDAQREAFEAAKAALAGTDVVVEPLADVL
DRGRSLADAPLYTPGQPDPLTMLIYTSGSTGTPKGAMYPESKVANMWQLATKATWDENAALPAITLNFMPMSHVMGRGILIGTLSSGGTAYFAARSDLSTFLEDLALVRPT
QLSFVPRIWDMLFQEYQSRLDRSGAPEDEVLAEVRQDLLGGRFVSAMTGSAPISAEMKNWVERLLDMHLLEGYGSTEAGSVFVDGHIQRPPVIDYKLVDVPDLGYFLTDRP
HPRGELLVKSEQMFPGYYKRPEITAEMFDEDGYYRTGDIVAELGPDQVEYLDRRNNVLKLSQGEFVTVSKLEAVFGDSPLVRQIFVYGNSARSYLLAVVVPTDPSLSKQAI
GDSLQDAARAAGLQSYEIPRDFIVETTPFSLENGLLTGIRKLARPNLKAYYGDRLEQLYTELAEGQANELSELRRNGAQAPVLDTVSRAAGALLGAAASDLAPEAHFTDLG
GDSLSALTFGNLLQEIFDVEVPVSAIVSPASDLRTIAEYIEAQRSGADVRPTFTSVHGRNATEVHASDLTLDKFIDAATLAAAPSLPGPVSEIRTVLLTGATGFLGRYLAL
EWLERMDLVDGKVICLVRAKSDEEARARLDKTFDSGDPKLWAHYQKLAADHLEVIAGDKGEADLGLDQVTWQRLADTVDFIVDPAALVNHVLPYSELFGPNALGTAELIRI

ALTTRIKPFAYVSTIGVGGGIEPGKFVEAGDIRAISPVRRVDDGYANGYGNSKWAGEVLLREAHDLAGLPVTVFRCDMILADTTYAGQLNLPDMFTRMMFSLVATGVAPKS
FNQLDADGNRQRSHYDGLPVEFIAEAISTLGAHVQDGFETYHVMNPHDDGIGMDEFVDWLIEAGYPIQRVEDYQEWLARFETTLRALPDKQRQASLLPLLHNYQQPGVPVN
GAMAPTDVFRTAVQDAKIGPDKDIPHVSREVIVKYISDLKLLGLL
>Mycobacterium_neoaurum
MFAENLDDQQRLADLYATDPEFAAAAPDPAIADAVAAAEMRLPEIVRTVLTGYAERPALGSRAVQLVTDDATGRTRAELLGHFETITYGQLWDRVRAVTNAWSDTVRPGDR
VAILGFGSVDFTVIDIALTQLGAVSVPLQTSATASALAPIVAETEPALIASDVNHLDDAVTLALGSGVQTVVVFDQNPAVDDDREAIAAATARLDGQTLATLDEVIAAGAE
RADVAIPAQEGDPLSLLIYTSGSTGAPKGAMYPQGKVADIWRPAINSHWDARGHVPAIVLSFMPMSHVMGRGILYASLASGGVVNFAARADLSTLLEDLALTRPTQLNFVP
RVWDMLFQDYQSRAAHGGSEADILADMRTNLLGGRYVSALTGSAPISPELKAWVERLLELHLVEGYGSTEAGAVFVDGQISRPPVLAYKLVDVPELGYHSTDVPHPRGELL
IRSEQLFPGYYKRPEVTASVFDEDGFYRTGDIVAELGPDQVAYIDRRNNVLKLSQGEFVTVSKLEAVFNTAPLVHQIYIYGNSARPYLLAVVVPTDPNATKADIADSLKAA
ARTADLQSYELPRDFLVETTPFSTENGLLTGIKKLAWPKLKERYGAELEQLYTDLADGQAGELQALRATGADAPVLETVGRAAVALLGAASSDIAPDVHFTDLGGDSLSAL
TFGNLLADIFDVEVPVSVIVGPTADLASIAAHIETQRSGAGLRPSFGSVHGKDATVARAADLTLDKFIDAETLTAAGDLAAPATNVRTVLLTGATGFLGRYLALEWLERMD
LVDGKVIALVRARSDAEARARLDATFDTGDPKLLAHYRKLADKHLEVLAGDKGEHDLGLDRRTWQRLADSVDLIVDPAALVNHVLPYSELFGPNALGTAELIRIALTTRIK
PFVYVSTIGVGAGIEPGRFIEDADIREISATRVLDDSYANGYGASKWAGEVLLREAHEQFGLPVSVFRCDMILADTSYAGQLNLPDMFTRMMLSLVATGVAPKSFNQLDAQ
GNRQRSHYDGLPVEFIAEAISTLGADVTDGFETYHVMNPHDDGLGLDEFVDWLIEAGHPIRRIDDYQAWFEQFGATLRTLPDRQRQASLLPLLHNYTTPGLPVNGAMAPTD
VFRTAVQEAKIGPDKDIPHVGKDVIVKYITDLQLLGLL
>Mycobacterium_vaccae1
MSTDTREGRLARRIADLFATDPQFAAAVPDETVAAAVEEHAAHLPDIMRTVLDGYADRPALARRAVRFVEDATGRTVAELLPHFETITYAELAHRIHGVTAALTDVHPGDR
VALLGFTSVDYTTVDMALSMLGAVLVPLQTSAPLSTLRPIIAETEPVLIASSVDTLDDAVALALDAPDAARLVVFDHRAEVDDHRDALTSATARLRAAGSPLEIETLAEVI
ARGSTMPAREQFSPDADTLMLLIYTSGSTGAPKGAMYTERLVATTWRRSSRSFWGDHGPLPSITLNFLPMSHVMGRGLLYATLGAGGTAYFAAKSDLSTFLEDLALVRPTQ
LSFVPRIWDIIFAEVAKELERRTADAADVLADLRQSLLGGRYVSAMTGSAPLSAEMESFVEQLLDMHLIDGYGSTEAGAVLVDGQIQRPPVIDYKLVDVPDLGYFSTDRPH
PRGELLVRSETLFPGYYKRPDITAEMFDEDGYYRTGDIVAETAPDRLTYLDRRNNVLKLSQGEFVTVSKLEAVFGDSPLIHQIYVYGNSARPYLLAVVVPTDAALAREDVK
TAVAESLQDVARAADLQSYEIPRDFLIETTPFTLENGLLTGIRKLARPALKERYGDRLEALYAELAEGQTDELRELRRSGAERPVQETVLRAAAALLGAAATDLQPDAHFT
DLGGDSLSALTFGNLLHDIFGVEIPVGVIVSPATDLQALAGHIETARTTGSARPSFTSVHQHPVTEVYARDLTLDKFVDTDTLAAAPALPGPAAEIRTVLLTGATGFLGRY
LALEWLERLSLVGGTLICLVRAKDDAAARDRLDRTFDSGDPKLLEHYRRLAADHLEVVAGDKGDADLGLDARTWQRLADTVDLIVDPAALVNHVLPYNQLFGPNVVGTAEL
IRLALTTKLKPFTYVSTMAVGAGVEPGRFTEDGDVRQISATRKVDDSYANGYGTSKWAGEVLLREAHDLCGLPVSVFRCDMILADTTYAGQLNLPDMFTRLILSLVATGVA
PESFYRLDADGRRPRAHYDGLPVEFVAEAISTLGAQVVSGFETYHVMNPHDDGIGLDEFVDWLIEAGYPVRRVGDYPTWLQRFTVAVNALPDRQRQASLLPLLHNYQQPEI
PVCGAVAPTDRFREAVQDAKIGPDKDIPHLSAQVIVKYVTDLQLLGLL
>Mycobacterium_phlei
MASESRDVRLQRRIAELYDTDPQFAAARPDEAVARAVNAPGLTLSQVIRTVLDNYADRPALGYRAVEFAADPASARTVARLLPRFDTITYRELGERIDATTAALGHDGLRP
GERVAILGFSSVDYTTIDIAAFNLGAVSVPLQTSAPPSQLRPMTAETEPAVIAASVDFLDDALELIRTGHAPRRLVVFDFHPEIDDHRDALAAATTGLADTAMTVETLDSL
LTRGRTLPAPTGYRDRDDDLALLIYTSGSTGAPKGAMYQRRMVTNMWRRATTAIGGKEAAPWLTLNFMPMSHVMGRGILSTTLCSGGTAYFAARSDLSTLLEDLALVRPTQ
LTFVPRIWEMIFQEYQRDVARRPEAEVLADLREHRLGGRFLAAMTGSAPMSPEMTAFAESVLDLHLVDGYGSTEAGSIVLDGQVLRPPVLDYKLVDVPELGYFSTDRPYPR
GELLVKTELMFPGYYKRPDITAEVFDEDGYYRTGDIVAELGPDRLAYVDRRNNVLKLSQGEFVTVSKLEAAFAASPLVRQIYIYGNSAHPYLLAVVVPTEDALTRYDAATL
KTAISESLQDVGRAAGLQSYEIPRDFLVETTPFTLENGLLTGLRKLARPKLKEHYGERLEQLYTELADGQAEELKELRTHGAQQPTLTTVSRAATALLGTASAEVRPDAHF
TDLGGDSLSALTFGNLLGEIYAVEVPVGVIVSPANDLAAIADYIDTARRPGDGRPTFAGVHGDDAAEVHARDLTLDRFLDDATLAAATALPGPAPEIRTVLLTGATGFLGR
YLALEWLERMAMVGGTLICLVRGRDDAAARARLDQIFDSGDPELLRHYRELADRHLEVIAGDKSDADLGLDRRTWQRLADTVDLIVDPAALVNHVLPYRELFGPNVVGTAE
LIRLALTGRLKPYLYTSTIAVGAGIAPGQFTEDADIRQISATRTLDDSYANGYATSKWAGEVLLREAHDLCGLPVAVFRCDMILADTSYAGQLNVPDMFTRLILSLVATGI
APLSFYELDAAGHRQRAHYDGLPVEFVAEAVSALGLDVAGGFATYHVMNPYDDGIGLDEFVDWLTGAGYPIEHVNDYGTWFQRFETAIRGLPERQRQASLLPLLHSYQRPQ
PPIRGSAAPTDRFRSAVQDAKIGPDKDIPHITPEVIVKYVTDLRLLGLL
>Mycobacterium_smegmatis1
MTSDVHDEQSTRRIAELYATDPEFAAAAPLPAVVDAAHKPGLRLAEILQTLFTGYGDRPALGYRARELATDEGGRTVTRLLPRFDTLTYAQVWSRVQAVAAALRHNPIYPG
DAVATIGFASPDYLTLDLVCAYLGLVSVPLQHNAPVSRLAPILAEVEPRILTVSAEYLDLAVESVRDVNSVSQLVVFDHHPEVDDHRDALARAREQLAGKGIAVTTLDAIA
DEGAGLPAEPIYTADHDQRLAMILYTSGSTGAPKGAMYTEAMVARLWTMSFITGDPTPVINVNFMPLNHLGGRIPISTAVQNGGTSYFVPESDMSTLFEDLALVRPTELGL
VPRVADMLYQHHLATVDRLVTQGADELTAEKQAGAELREQVLGGRVITGFVSTAPLAAEMRAFLDITLGAHIVDGYGLTETGAVTRDGVIVRPPVIDYKLIDVPELGYFST
DKPYPRGELLVRSQTLTPGYYKRPEVTASVFDRDGYYHTGDVMAETAPDHLVYVDRRNNVLKLAQGEFVAVANLEAVFSGAALVRQIFVYGNSERSFLLAVVVPTPEALEQ
DPAALKAALADSLQRTARDAELQSYEVPADFIVETEPFSAANGLLSGVGKLLRPNLKDRYGQRLEQMYADIAATQANQLRELRRAAATQPVIDTLTQAAATILGTGSEVAS
DAHFTDLGGDSLSALTLSNLLSDFFGFEVPVGTIVNPATNLAQLAQHIEAQRTAGDRRPSFTTVHGADATEIRASELTLDKFIDAETLRAAPGLPKVTTEPRTVLLSGANG
WLGRFLTLQWLERLAPVGGTLITIVRGRDDAAARARLTQAYDTDPELSRRFAELADRHLRVVAGDIGDPNLGLTPEIWHRLAAEVDLVVHPAALVNHVLPYRQLFGPNVVG
TAEVIKLALTERIKPVTYLSTVSVAMGIPDFEEDGDIRTVSPVRPLDGGYANGYGNSKWAGEVLLREAHDLCGLPVATFRSDMILAHPRYRGQVNVPDMFTRLLLSLLITG
VAPRSFYIGDGERPRAHYPGLTVDFVAEAVTTLGAQQREGYVSYDVMNPHDDGISLDVFVDWLIRAGHPIDRVDDYDDWVRRFETALTALPEKRRAQTVLPLLHAFRAPQA
PLRGAPEPTEVFHAAVRTAKVGPGDIPHLDEALIDKYIRDLREFGLI
>Mycobacterium_smegmatis2
MTIETREDRFNRRIDHLFETDPQFAAARPDEAISAAAADPELRLPAAVKQILAGYADRPALGKRAVEFVTDEEGRTTAKLLPRFDTITYRQLAGRIQAVTNAWHNHPVNAG
DRVAILGFTSVDYTTIDIALLELGAVSVPLQTSAPVAQLQPIVAETEPKVIASSVDFLADAVALVESGPAPSRLVVFDYSHEVDDQREAFEAAKGKLAGTGVVVETITDAL
DRGRSLADAPLYVPDEADPLTLLIYTSGSTGTPKGAMYPESKTATMWQAGSKARWDETGVMPSITLNFMPMSHVMGRGILCSTLASGGTAYFAARSDLSTFLEDLALVRPT
QLNFVPRIWDMLFQEYQSRLDNRRAEGSEDRAEAAVLEEVRTQLLGGRFVSALTGSAPISAEMKSWVEDLLDMHLLEGYGSTEAGAVFIDGQIQRPPVIDYKLVDVPDLGY
FATDRPYPRGELLVKSEQMFPGYYKRPEITAEMFDEDGYYRTGDIVAELGPDHLEYLDRRNNVLKLSQGEFVTVSKLEAVFGDSPLVRQIYVYGNSARSYLLAVVVPTEEA
LSRWDGDELKSRISDSLQDAARAAGLQSYEIPRDFLVETTPFTLENGLLTGIRKLARPKLKAHYGERLEQLYTDLAEGQANELRELRRNGADRPVVETVSRAAVALLGASV
TDLRSDAHFTDLGGDSLSALSFSNLLHEIFDVDVPVGVIVSPATDLAGVAAYIEGELRGSKRPTYASVHGRDATEVRARDLALGKFIDAKTLSAAPGLPRSGTEIRTVLLT
GATGFLGRYLALEWLERMDLVDGKVICLVRARSDDEARARLDATFDTGDATLLEHYRALAADHLEVIAGDKGEADLGLDHDTWQRLADTVDLIVDPAALVNHVLPYSQMFG
PNALGTAELIRIALTTTIKPYVYVSTIGVGQGISPEAFVEDADIREISATRRVDDSYANGYGNSKWAGEVLLREAHDWCGLPVSVFRCDMILADTTYSGQLNLPDMFTRLM
LSLVATGIAPGSFYELDADGNRQRAHYDGLPVEFIAEAISTIGSQVTDGFETFHVMNPYDDGIGLDEYVDWLIEAGYPVHRVDDYATWLSRFETALRALPERQRQASLLPL
LHNYQQPSPPVCGAMAPTDRFRAAVQDAKIGPDKDIPHVTADVIVKYISNLQMLGLL
>Mycobacterium_chubuense
MSTDTREERLDRRIADLHATDEQFAHATPDDTISHTIDQPGARLPQIMQTVLDGYADRPALGQRAVEFVADPHTGRTVAELLPRFDTITYAEVSRRVHAIANALTDVRPGD
RVALLGFTSVDYTTIDMALAYLGAVSVPLQTSAAASALSPIVAETEPVVIASAVGALADAVELTLDAPAVSRLVVFDYRPEVDDDRDALAAATSRLFEAGSTVEVATLGEV
IAAGAGSPAGQPFLSPDDDPLMLLIYTSGSTGAPKGAMYPERLVANAWRRSTRASWGDQGALPSITLNFLPMSHMMGRGLLYGTLGAGGTAYFAAKSDLSTFLDDLAMVRP
TQLTFVPRIWDTIFGEVAKELERRPSDQDAVYAEMRQSLLGGRYVMAMTGSAPLSPEMTVFVESLLDMHLIDGYGSTEAGAVFVDGQVQRPPVLDYKLVDVPDLGYFRTDR
PHPRGELLVKSETLFAGYYKRPEITAEMFDEDGFYRTGDIVAETGPDQVRYLDRRNNVLKLSQGEFVTVSKLEAVFGDSPLVKQIYVYGNSSRPYLLAVVVPTEEALARED
AKPAISESLADVAKAAGLQSYEIPRDFLVETTPFTLENGLLTGIRKLARPKLKAHYGDRLEALYHELAEGQADELRELRRSGADRPVLETVSRAAGALLGAGAADVQPDAH
FTDLGGDSLSALTFGNLLHDIFGIEVPVGVIVSPATDLQALAAYIEAARLPGSKRPTFASVHGRDAAEAHARDLTLDKFIDEATLAQAPSLPGPSSEVRTVLLTGATGFLG
RYLALEWLERMDMVDGTVICLVRAKDDAARQRLDDTFDSGDPTLLAHYRELAADHLEVLAGDKGEADLGLDPQTWQRLADSVDLIVDPAALVNHVLPYSQLFGPNALGTA
ELIRIALTTRQKPFVYVSTIGVGAGITPGDFTEDGDVRQYSATRKVDDSYANGYSNSKWAGEVLLREAHDLCGLPVSVFRCDMILADTTYAGQLNLPDMFTRLMFSLVATG
IAPESFYELDTDGRRQRAHYDGLPVEFIAEAISTLGVHVTEGFETYHVMNPYDDGVGLDEFVDWLIDAGFPLHRVGDYAGWLQRFTTAINALPDRQRQASLLPLLHNYQRP
EMPIRGSVAPTDRFRTAVQEAKIGPDKDIPHVTPDVIVKYITDLQLLGLL
>Mycobacterium_vanbaalenii

Supporting information

MSTDTREDRLARRIADLYATDPQFAAAAPDDAISHAIDQPGTHLPVIVQTVLDGYAERPALGQRAVRFVTDPATGRTTTELLPRFETITYAELSRRIHAVTAALTDVHPGD
RVAVLGFTSIDYTTVDMALAMLGAVAVPLQTSAPATTVRPIVAETEPVVIASSVDALTDAVGLALDAPTVTRLVVFDHRAGVDDHRDALISASDRLRAANSPIEVETITDI
VARGSKLPVRAQFSADGDALSLLIYTSGSTGAPKGAMYPQHLVANSWRRLARSFWGDLGVFPAITLNFMPMSHVMGRGLLYGTLDAGGTAYFAARSDLSTFLEDLALVRPT
QLSFVPRIWDTIHAEVSQELERRPSDATEVIADLRRSLLGGRYVTAMTGSAPLSPEMRAFVENLLDVHLIDGYGSTEAGAVFVDGRVQRPPVIDYKLVDVADLGYFSTDRP
HPRGELLVKSETLFPGYYKRPDVTAEMFDEDGYYRTGDIVAETGADQLTYLDRRNNVLKLSQGEFVTVSRLEAVFGNSPLVRQIYVYGNSARPYLLAVVVPTEAALAGADA
KAAVAESLQDVAKATGLQSYEIPRDFLLETTPFTLENGLLTGIRKLARPRLRERYGEQLEALYTMLSEEQADELRELRRSGGERPALETVGRAAGALLGTTAGELEPSAHF
TDLGGDSLSALTFANLLRDIFDVDVPVGVIVSPATDLQALADYVESARRHGSVRPTFESVHGHPGTEVHARDLTLDEFVDAATLAHAPTLPGPRAEVRTVLLTGATGFLGR
YLALEWLERMALVGGKLICLVRAKDDAAARVRLDSTFDSGDPELLRHYRRLAADHLEVIAGDKADADLGLDARTWQRLADTVDLIVDPAALVNHVLPYRQLFAPNVLGTAE
LLRIALTTRMKPFVYVSTIGVGAGIEPARFTEDADIRQISATRRIDDSYANGYGNSKWAGEVLLREAHDLCGLPVSVFRCDMILADTTYAGQLNLPDMFTRLIFSLVATGV
APESFYHLATDGTRQRAHYDGLPVEFIAEAISTLGSDVASGFRTYHVMNPHDDGIGLDEYVDWLIDAGHPIRRVGDYPTWLQRFTVAITALPERQRQASLLPLLHNYQHPE
TPIRGSIAPTDRFREAVQDAKIGPDKDIPHVTPQIVIKYVTDLQRLGLL
>Mycobacterium_rhodesiae1
MSADTREERLARRIADLYENDQQFADARPDTAISRAISEPQQPLAEIIRGAMDGYADRPAVGTRAVDFVTDPQTGRTTVRLLPRFDTITYGELWNRVEALASALATDVDAG
DRIAILGFTSVDYTVADLAAFRLGAVSVPLQTSAPVTQLRPIVTETEPTAIASSVDFLDDAVDLVLTGHAPRRLVMFDIHPEVDDHRDALESARARLADAGSSVAIETLAD
AVTRGGALPSAPTYTPNADALALLIYTSGSTGAPKGAIYTRFLVGQTWRPASWGNDSPDPFITLSFMPMSHVMGRSGLYATLAHGGTAYFAAKSDLSTLLDDLALVRPTQL
TFVPRVWDMLAAEARREIEARLSPAADADTEAQVRAEVRTLMLGNRYLTAMTGSAPLSPDLRAWVEDFLDMHLSDGYGSTEAGGVFHDGKVVRPPVIDYKLVDVPELGYFH
TDRPHPRGELLLKTTNMFPGYYKRPEVTAEVFDVEGFYKTGDIVAEIGPDQLQYLDRRNFVLKLSQGEFVTASKIESVFETSPLVRQIYIYGNSARSYLLAVVVPTVEALE
ALDGEALPKAINESLQDVAKTAGLQSFEIPRDFIVETTPFTLENGLLTGIRKLARPKLKEFYGPRLEELYTEMADGQAAELRELRQNGAQRPVVETISRAAGALLSAATAD
IGPEAHFTDLGGDSLSALTFANLLKDIFDIDVPVGVIVSPANDIQALADYVETERTGSKRPTFAAVHGPDATAIHAGDLTLDKFLDEQTVAAASSLPGPASEIRTVLLTGA
TGFLGRYLALEWLERMSLVGGKVICVVRAKDDAAARRRLDATFDSGDPELLEHYHSMAADHLEVLAGDKGDADLGLDAATWQRLADTVDLIVDPAALVNHVLPYDQLFGPN
VVGTAELIRIALTTKIKPYIYVSTVGVGDQIDPPAFVEDADVRVMSATRRVNDTYANGYGNSKWAGEVLLREAHDLSGLPVAVFRCDMILAEPRYAGQLNVPDMFTRLMLS
LVATGIAPGSFYELDTAGNKQRAHYDGLPVDFIAEAISILGVHHAGAFETYHVMNPYDDGIGLDEYVDWLIEAGYALDRVPDYRQWLQRFETSMRSLPDRQRQASLLPLLH
NYQQPSTPMRGSFAPTDRFRAAVQEAKIGPDDIPHITADIIAKYITDLQLIGLL
>Mycobacterium_rhodesiae2
MTTELRPGGVGERVTYLYENDRQFRAASPRLDVLDAARKPGLRLSQVLRTLAEGYSDRPAVGTRAVQTIGDELTGRCATQLLPAFNTISYGELWSRVQAVANAWHHDPVEA
GDFVATIGFSSADYLTIDLVCGYLGLVAVPLQHNTSASRLVPILEESAPRVLAVSADYLDLAVEAALGSGWLSRLVVFDYRPQDDNHRDRVQHARDSLAGAGMEVIVETLP
DIIARGTRAPEAPLFTDGSDDRLAMILYTSGSTGAPKGAMWTERMVRTLWTIPMKSTESVVINLNFMPLNHLGGRLPLAASFQTGGTSYFVAESDLSTLFDDWMLVRPTDL
GLVPRVVDMLYQRYQQIVDRLSADGLDLDTAERTAKTEIRETLLGGRVLGGFVSTAPLSAEMRLFLESCLQADIVDTYGLTEIGAVTTDGLVVRPLVLDYKLVDVPELGYF
STDKPHPRGELLVKSAAAMPGYYKRPEITAEVFDADGYYRTGDIVAELEPDRLAYVDRRNNVIKLSQGEFVAVANLEAEFARTPLIRQIYVYGNSERSSLLAVVVPTEEAL
AVGVNDQLRAAVRAAMSQTARDAGLQPYEVPVDFLIETEPFSVADGLLSGVGKLLRPKLKERYGAELEALYDQVDAARADDVRALRDAAQHQPVVDTVIQAAAALLGIPSA
SVTRDDHFIDLGGDSLSALTFSNLLQELFGIETPVGTLTGPTTTLGDVASHLEQGAGDGVARPTATSVHHSDTTIHAADLTLDKFLPANLLDDARALPLPDAEPHTVLLTG
ANGYLGRFLALEWLQRLAQTGGTLICLLRGSDNESARMRLEQIFDDGDAQMSERFHDLAAEHLEVIAGDISQPQLGVDPQTWDALTQRVDLIVHPAALVNHVLPYRQLFGP
NVVGTAEVIALALSSRLKPINYLSTVSVAMTVEPERFEEDGDIRTISPTRPIDDSYANGYGNSKWAGEVLLRQAHDLCGLPVSVFRSGMILADRRYDGQLNVSDMFTRLIY
SLVRTGLAPESFYQRSESGERTRSHYDGLPVDFVAESITTLGAGARRGFRSYDVMNPHDDGISLDVFVDWLIETGHSITRVDDYDDWLSQFDSALRALPDAQRQQSVLPLL
TAYSRPERPLLGSLAPAQVFRKAVQENRIGADQDIPHLSRQLIEKYVSGLRGQDLL
>Nocardia_seriolae
MVEDTARAEIHRRIAERVLADEQVRVAMPLPEVSEAARQPGLGLARAVEVLMVGYAERPAIGERAAEIVTGADGRRIRRLLPEYRTITYAELWSRAGAIAAAWQHDPLRAG
DFLCVLGFGSGDFAALEIAAIRQGLVTVPLQANAAAAQWRSIIEETGARTLAVSLELLDSALDVVLDGSPVTSIVVFDFEPEEDRQAEILVGARDRIAASGSTITLESLAA
VLERGATLPAVPLHVPADEDEVALLIYTSGSTGTPKGAIYPHRLVTGMWLGPNVIPAPVMNFCYMPLSHVAGRMVLSGTFARGGTAYFAASSDMSTLFEDIALVRPTEVFF
VPRVCDMLFQRCQSEVQRRTAAGESVEDADAAVKTALREEFLGGRLVRVMVGSAPVSAEMKEFMRSVMGQPVIDGYGSTEAGGILIDNEIRRPPVIDYKLADVPELGYFST
DKPHPRGELLVKSTQQIPGYFKRPDVTAEIFDADGFYRTGDIVAEVRPDHLVFVDRRNNVLKLSQGEFVAVSKLEAVYATSPLIAQIFVHGSGERSHLLAVIVPTAAARAL
APAERTAAIAESLRQIARDAELESYEIPRDFIVEDEPFTQENGLLSGIAKLLRPKLRERYGARLEQMYDEQAQRQRDELATLRREAAELPVLETVCRAARAVLGGTQPPPD
AHFTDLGGDSLSALSFSTLLAEIFGVEVPVGVIVSPANALTELAAHIEAERDSGGSRPTAATIHGGSEIRAADLTLDRFIDAATLAAAPALPLAPQPPRTVLLTGANGYLG
RFLCLEWLERLDDTDGTLVCVVRGRDAEAARQRLDEVFDSGDPELTQRYRELAARRLRVLPGDIGEPNLGLGQADWQELADTVDLIVHPAALVNHVLPYGQLFGPNVVGTA
EVIRLALTTVRKPVTYLSTVAVAAQIDPGVFTEDGDIREISAVRAVDDGYANGYGNSKWAGEVVLREAHELCGLPVAVFRSDMILAHSEFAGQLNLPDMFTRLLLSVLATG
LAPKSFYALDSHGNRQRAHYDGLPADFTAAAITVLGAQVREGFETYDVLNPHDDGISLDEFVDWLIDSGHPIDRIDDYRDWFTRFETALRTLPEHQRSASVQPLLHAYRRP
GVPIPGSMLPAKRFHAAVQQAELGPGGDIPHLTRELIDKYVADLKLRGLL
>Nocardia_farcinica
MESTRATRLRQRIAALYADDAQVRDARPDEAISTALREPGLRLRELVATVVDGYRDRPALAARSVQPAVDAATGACVARLLPEYTTMSYGELGLRLRAVAAAWQHDRLRPG
EFVATLGFTSPDYAVVDLACVWAGAVAVPLQASASVTQLTAILAETAPAILATGLDTLPHAVDCVLAGATPRALHVFDFDPAIDAQRTVYEAACARLAGTGVRVRTLAEVE
DRGRALPPAVIDDGPGDDRLALLIYTSGSTGTPKGAMYTERLVALMWLGQPQVAALTVNYLPLSHVAGRLALFGLLARGGTAYFTARADMSTLFEDLALARPTELFVVPRV
CEMVLQRFQTERLRRQADDDRVKADLRLELFGDRLLSVVCGSAPLAPELKAFMESVLDLTLHDGYGSTEAGSVVIDTTVRRPPVLDYRLADVPELGYFRTDKPHPRGELLL
KTTTMIPGYYRRPELNAQIFDEDGFYRTGDVVAELAPDRLVYVDRRNNVLKLAQGEFVTIARLEAIFANSPLVRQIFVYGNSERAYLLAVIVPSRQAMAGDPATLKTRIAE
SLQLIGRDAELEAYEIPRDFLIETEPFTTESGLLSGIGKILRPAVEARYRDRLEQLYADLAAAQQDELAALRREAGQRPVLETVTRAAAAILGGTASDLSPAAHFTDLGGD
SLAALALSNLLREIFAVEVPVGVITGPATDLRGLAAHIAAERENRTETPLFDRVHPDQILIRATDLALEKFFDAEELAAAATAAPPVAEPRVVLLTGANGYLGRFLCLEWL
ERLDRVDGRLICLVRGADEAAALARLEAAFDSGDPELVRRFKELAQRRLTVVAGDIGEPGLGLATATWRRLAAEVEHIVHPAALVNHVLPYRQLFGPNVAGTAEILRLALT
ERRKPIDFLSTVAVAAQIPADRFAEDGDIRVISPTRTVDRGYANGYGNSKWAAEVLLRAAHDRFDLPVAVFRSDMILAHGSFAGQLNIPDVFTRLLLSLLVTGIAPASFHA
ATVTGERPRAHYDGLPADFTAAAITALGARTAGFHTYDVLNPHDDGISLDTFVDWLIEAGHPIERIPEHSEWVTRFETALHALPERQRKHSLLPLLHAYRRPVPALRGSAL
PAAEFRAAVRAAGITADGDIPHLTRALIEKYVADLRLHGLL
>Nocardia_sp._NRRL_WC3656
MPDDVQKAERKRRLAAAMADDEVRAAWPDADVSAALARPGLRLAELVDTVMTAYADRPAVGQRAGEIVVDAEGRRVRRPLPRFETLSYRQLWSRAGALASTWQAGLRAGDF
VCTVGFVSSDYVTVDLAGVRLGTVAVPLQATAAVAQWNSIIAETEARVLACSAELLDAAVEAALASATIGQLVVFDSTGDDDERAAVAAARARLAESGREIAFDSLHDLVE
HGFALPAVPLASAPGDDPLSLLIYTSGSTGTPKGAMYTDRLAAAMWLYTAKAPVPAITLNYLPLSHVAGRLQLGGTLARGGTAYFTARSDMSTLFEDLELTRPTELVFVPR
VCEMLLQHHQGEVEARIAAGGERGVIEDEVKAELRERLLGGRFLGAMCASAPLAPEMRAFMESVLTIGLHDGYGSTEAGSVIVDNKVRRPPVLDYKLADVPELGYFGTDQP
HPRGELLLKTTTMFPGYFKRPEITAEMFDEDGFYRTGDVVAELGPDHLVYVDRRNNVLKLSQGEFVTVAKLESVFSTSALIRQIFVYGSSERAYLLAVIVPADEAQALPDP
RAALTESLQQLAKEAGLNSYEIPRDFLLETEPFTQDNGLLSGIGKLLRPKLKERYGERLERLYAELSQEQADELAALRHGAADRPVLETVGRAARALLGCASGDIRPEAHF
TDLGGDSLSALSLSNLLSELFGVEVPVGTIVHPANTLRRLAEHIDTERRGGGRRPTLATVHGRGTRVRAADLTLDAFIDHETLTAAKSLPPAPAPPRTVLLTGANGYLGRF
LCLEWLHRLDACDGTLICVIRGRDADAARARLDEAFDSGDPVLLQRYRELADRRLTVVPGDIGEPNFGLGEDRWRELAETVDLVVHPAALVNHVLPYDQLFGPNVVGTAEV
VRLAITARRKPVTYLSTVAVASQVDPARFTEDGDIREISPERIIDGGYANGYGNSKWAGEVLLREAHDLCGLPVAVFRSDMILAHSEFAGQFNLPDLFTRLLLSVLATGLA
PESFYRPAADGGRARAHYDGLPADFTAAAITALGPGAESGFDTFDVLNPHDDGIGLDTFVDWLVADGHRITRIADYADWLARFETALRALPERQRRHSVLPLLHAFRRPAP
AVPGSALPAERFRAAVQAAGTGPDGDIPHLSRELIEKYVRDLTAAVLL
>Nocardia_rhamnosiphila
MSIETRETRLEHRIAELYRTDEQFAAARPDPAVTERAGKPGLRAFEIARTVMEGYADRPALGQRAIEYVTDERTGRTAAKLRPEFETVTYREVWDRAGAIAAALTADGVRP
GDRVCLLGFTSVDYTVIDVALTRMAAVAVPLPTSSATARLLPIVAETEPVVIAASIDRLADAVELALTGHSPARLIAFDHHPRDDDDNAIATAARARLAEADGTVVAETLA
EVVARGAVLPPVPVPVTEADSLALLIYTSGSTGAPKGAMLTERLVADHWRAASSEKWGQRGTEPAIGLGFMPMSHIMGRAILYMTLGRGGTVFFAATSDLSTLLDDLALVR
PTQLSFVPRIWEMLFHRFEGEVARRGGDGADRTALEAEVAADLRRNLLGGRYLAATTGSAPISAEMRAWVESFLDMHVVDGYGATETGSIAVDGRVRRPPVSHYELVDVPE

LGYFRTDRPHPRGELVVRSDTLVPGYYKRPEVTARVFDADGAYHTGDIFAEVGPDQLVYVDRRSFVLKLSQGEFVTVSKVEAVFAESPLVRQIYVYGNSTRSYLVAVVVPT
AAARERDDSHVLKQLISESLQQVAKSAGLQSYEIPRDFLLEYQRFTPENGLLTGIRKLARPALKEHYGPRLEQLYTELAEAEAEELRAVRHGAGDRPTIETVVRAAAALLG
AATGEVRPDAHFTDLGGDSLSALTFGQLLREIFGVEVPVGFLIGPTADLRAVAHYIDEQRAGARRPTFAAVHGAEATEVHAGDLTLDKFLDEHTLAQAPHLPGPSATVRTV
LLTGATGFLGRYLALEWLERMARAGGTLICLVRATDDTAARSRLDETFDSGDPVLTARYRELAAGHLEVIAGDKGRADLGLDHDTWQRLADTVDVIVDPAALVNHLLPYTE
LFGPNVVGTAELIRLALTTKQKPYTYTSTIAVGDQVDPANFTEDADIRVISPHRGIDDRYANGYGNSKWAGEVLLREAADLCGLPVAVFRCDMILADTGYAGQLNVPDMFT
RLMLSLEATGTAPRSFYELDPDGRPQRAHYDGLPVGFIAEAIVSLGARAGTGFRTYHVMNPYDDGISLDTYVDWLIEAGHPIHRIPDYDSWLRRFETAVRALPERQRRYSL
LPLLDSYRKPQRAVRGSIAPTERFRAAVRHAEIGADRDIPHVTPEIIVKYTTDLHLLGLL
>Nocardia_otitidiscaviarum
MLDDARAERRERRIADALADDQVREAAADAAVSESVRRVEVRLARIVDAVMSGYGDRAALAWRRSELVDGAVRLLPEYSTMTYRELWRQAGAVAAEWGADPVRAEDFVCTL
GFTSPDYTVVDLALMRLAAVAVPLQASASVAQWRSIMAETEPRMLAASAETLPAAVEAVLGGFAPRRVLVFDYRPELEAHRSAVDSARERLAEVGCTVATVADAVDRGANL
PAPLRIPSDRERLALLIYTSGSTGAPKGAMYTDRLVAGLWLSANEIRVPALTMNYMPLSHIAGRMSLYGTLMRGGTAYFAAASDMSTLLDDFGLARPTELFLVPRVCELLH
QRYQSELDRRVVAGEDAETAATNVKAELRERVLGGRYLTALSGSAPLAAEMKTFMESLLDDELHDGYGSTEAGSVLLDNRIKRPPVLDYRLVDVPELGYFRTDKPHPRGEL
LLKTESMFPGYYKRPEITAEMFDADGFYRTGDVVAELGPEQLVYVDRRNNVLKLSQGEFVTVAALEAVYATSPLIRQIFVYGSSERAYLLAVVVPTDAVLALPAARARAEV
SESLQRIAKESGLRPYEIPRDLIIESEPFTIDNGLLSGIGKLLRPKLKEHYGERLEQLYAELAEQREDELTALRRGAHDRPILDTVTRAAGAVLDLTAGEVSPDAHFADLG
GDSLSALSFSTLLRDIFGVEVPVGFIVGPATDLARIAEYLVSERDSGSRPTAATVHGDDGLLRADDLALEAFLDPATLDAAAHLPSALEPPRTVLLTGANGYLGRFLALEW
LQRLDVSGGTLICLIRGSDADSARRRLDAVFATGDPELEAHYRELAERRLRVLPGDIGEPNLGLREQDWRDLAETVDLIVHPAALVNHVLPYAQLFGPNVVGTAEVIRLAL
TSRLKPVTYLSTVAVSAGIDPETFTEDGDIREISPVRRLDDGYANGYGNSKWAGEVLLRNAHDRFGLPVAVFRSDMILAHSRYAGQLNVPDMFTRLLLSVLATGLAPGSFH
DAHGERHRAHYDGLPADFTAAAVTTLGSRVTSGYETYDVLNPHDDGISLDTFVDWLIEAGHPIDRIDDYAEWFARFDTALRALPEHQRQHSLLPLLHAYRRPTPPLHGVAL
PAKHFRAAVQQAKLGPDGDIPHVTRELIEKYASDLRLLGLI
>Nocardia_sp._BMG51109
MEIKDEQAQLIRRATELIENDEEIRAARPLERVGEAAGRPETRLSQILATIMEGYADRPALGRRAVEFVADEHGRRHARLLPRFETITYGQLWERVRALGGAWYHDPTRAG
DFVAVLGFTSIDYTVVDLACTYLGAVSVPLQAGAAQAQLTPIVAETAPRVLATDVEQLGAAVELAIAADSVRSLIVFDYHAEDDDHRATVDTARRRLADSPVEVEILDDVL
ARGRELPSAPPHTAGDENDLALLIYTSGSTGTPKGAMYSEQLLGRMWRGGQDRPLPSIGMSYMPMSHVAGRASMIGCLATGGTVYFAARSDMSTLFDDIALVRPTILFFVP
RVCDMVFQRFRSEVDRRMAAGQGRESAEQEVKTELREHFLGGRFLVAVCGSAPVSADMRAFMESVLEFGMLDGYGATETGIVLVDNQVQRPPVVDYKLVDVPELGYFGTDK
PHPRGELLLKSQTLVPGYYKRPELNAELFDADGFYHTGDVMAEIEPDHLVYVDRRNNVLKLSQGEFVAVSKLEAVYTGSPLVRQIYVYGSSERAFLLAVIVPTADAVAAPQ
VEDLKTAIGESLQQVAKDAELNSYEIPRDFLLETEPFTMAGGLLSGIGKLLRPRLKERYGAELDRRYEQLEREQQDELSALRSAAAERPVLEIVERATKALLGCATTDLSP
DAHFTDLGGDSLSALSLSNLLQEVFGVEVPVGVIVGPATDLRRLADYIQDERNSDNGRPTSTSVHGSGTEVRAADLTVDKFVDAATLAVAGGLPAATRPPRTVLLTGANGY
LGRFLCLEWLERLHDSGGTLVCVVRGSDAAAARARLDQVFDSGDPELLRRYREVADGTLEVLAGDIGEPNAGVGEREWQRLAETVDLIVHPAALVNHVLPYDQLFGPNVVG
TAEVIRLALTARMKPVTYLSTVGVAAQVDPEVFTEDGDIREVSPARALDDSYANGYGNSKWAGEVLLREAHELYGLPVAVFRSDMILAHSRYAGQFNVPDMFTRLLLSVLA
TGLAPKSFYATDSDGNRQRAHYDGLPADFTAAAITTLGTRVATGYETFDVLNPHDDGLSLDVFVDWLIAAGHSIERIDDYADWLSRFETALRALPERQRQHSVLPLLHAYR
HPDLPIRGAALPAKRFQVAVQEAGLGDGDIPHLSPALIEKYASDLKLRNLL
>Nocardia_iowensis
MAVDSPDERLQRRIAQLFAEDEQVKAARPLEAVSAAVSAPGMRLAQIAATVMAGYADRPAAGQRAFELNTDDATGRTSLRLLPRFETITYRELWQRVGEVAAAWHHDPLRA
GDFVALLGFTSIDYATLDLADIHLGAVTVPLQASAAVSQLIAILTETSPRLLASTPEHLDAAVECLLAGTTPERLVVFDYHPEDDDQRAAFESARRRLADAGSLVIVETLD
AVRARGRDLPAAPLFVDTDDDPLALLIYTSGSTGTPKGAMYTNRLAATMWQGNSMLQGNSQRVGINLNYMPMSHIAGRISLFGVLARGGTAYFAAKSDMSTLFEDIGLVRP
TEIFFVPRVCDMVFQRYQSELDRRSVAGADLDTLDREVKADLRQNYLGGRFLVAVVGSAPLAAEMKTFMESVLDLPLHDGYGSTEAGSVLLDNQIQRPPVLDYKLVDVPEL
GYFRTDRPHPRGELLLKAETTIPGYYKRPEVTAEIFDEDGFYKTGDIVAELEHDRLVYVDRRNNVLKLSQGEFVTVAHLEAVFASSPLIRQIFIYGSSERSYLLAVIVPTD
DALRGRDTATLKSALAESIQRIAKDANLQPYEIPRDFLIETEPFTIANGLLSGIAKLLRPNLKERYGAQLEQMYTDLATGQADELLALRREAADLPVLETVSRAAKAMLGV
ASADMRPDAHFTDLGGDSLSALSFSNLLHEIFGVEVPVGVVVSPANELRDLANYIEAERNSGAKRPTFTSVHGGGSEIRAADLTLDKFIDARTLAAADSIPHAPVPAQTVL
LTGANGYLGRFLCLEWLERLDKTGGTLICVVRGSDAAAARKRLDSAFDSGDPGLLEHYQQLAARTLEVLAGDIGDPNLGLDDATWQRLAETVDLIVHPAALVNHVLPYTQL
FGPNVVGTAEIVRLAITARRKPVTYLSTVGVADQVDPAEYQEDSDVREMSAVRVVRESYANGYGNSKWAGEVLLREAHDLCGLPVAVFRSDMILAHSRYAGQLNVQDVFTR
LILSLVATGIAPYSFYRTDADGNRQRAHYDGLPADFTAAAITALGIQATEGFRTYDVLNPYDDGISLDEFVDWLVESGHPIQRITDYSDWFHRFETAIRALPEKQRQASVL
PLLDAYRNPCPAVRGAILPAKEFQAAVQTAKIGPEQDIPHLSAPLIDKYVSDLELLQLL
>Tsukamurella_paurometabola
MSIETVQNGVPAEGSVPPADQQTERLPQVIARIFAQFADRPAFATREAGPGTPYATVSYREIWRRVTALVASWQSEVAPGDFVAILGFTSSDFVTVDLATTLLGAPNVPLQ
AGAPAARIATILDETRPKILAVSADQVDLAQEALAESAATPRVVVFDGERDGYEGIEADILSGSALPAPEFFAEPGTDPLVTLIYTSGSTGTPKGAMYTEQLVRDAWLKVD
SIVDIDMPAESLLHFLPMSHMYGRNWLIAGLASGGTGYFAGASDMSTLFDDLAAARPTAIGLVPRVCELIHQRYLAVEADTDAETARVELRDRVLGGRLQAAMCGSAALSS
ELQTFMEWLLGIDIQIGYGSTEAGGVIRDGVVVRPPVTEYKLIDVPELGYFVTDSPHPRGELLVKSTQLIPGYYNSDKRIRDDEGFYRTGDVMAELGPDRLEYVDRRSNVI
KLAQGEFVPIAQLEAIYAAGPDVHQIFLYGTSERSYLIGVVVPAPGPDGETDAQTRTRVLDGLAAIARENDLAAYEVPRDVLIERDPFSQENGLRSGIGKLVRPALIARYG
DRLHDLYAQADTRQREGLRALDASGPIIDTVLGAAALTLGADIADFDADTRFGDLGGDSLSALSLATTLEGLYDVPVPVQTIVGPTATLGGVARHIEKARSGGVAAPTADS
VHGVGASVARATDLTLEKFIDPELLALAPTLPAATGEPNTVLLTGSTGYLGRFLLLDWLRRVAPHGGTVIALVRGADADDARRRVTAAIGDSDPDLTQEFTSLAEHHLHVI
AGDFGSPALGLDDATWSDLAGRVDHVVHCGALVNHVLPYDQLFGPNVVATGEVVRLALTTRRKSVDYVSTVAVVPQDDGRVLVEDDDVRELGAERRIGDAYANGYAVSKWA
GEVLLHEAADLADLPVRVFRSDMILAHSRFHGQFNEVDQFTRLLLSIAETGLAPASFYTPDPSGHRPHYDGLPVDFTAEAITTLSAAGRSGYRTFHVLNANDDGVSLDSFV
DWIAASGRSIERIDDYDTWFARFEQALQQLPDEARQRSVLPLLHAVREPAPAAGTSALSVDRFRGAVRETGVGPGDIPVLDRALIEKYLRDFETAGWL
>Mycobacterium_obuense
MPTDTREERLAHRIDDLSATDTQFAAALPDEAIAEAIEDPQLRLPQIIATVLDGYADRPALGQRAVRLVADPHTERTEAQLLPHFDTITYGELSTRIHHLLTALTDVDPGD
RVAILGFTSVDYTVIDTTLVLRGAVSVPLQTSAPAATLRPIVAETEPVVFAASVDHLSDAVDLVADAESVGRLIVFDYRAEVDDHRDAIADARARLADAGRSIEIVTLSEV
LAHGATLPAAQPFTSPDDDPLLLLIYTSGSTGAPKGAMYPERLITNAWRRSGRSAWGGEQTTPSITLNFMPMSHMMGRGVLYGTLGAGGTAYFAARSDLSTFLDDLALVRP
TQLSFVPRIWDTIAAEVAKEVDRRPDDLADVYADLRQSLLGGRHVMAMSGSAPLSPEMRTFVEDLIDIHLTDGYGSTEAGAVFVDGQVQRPPVIDYKLVDVPDLGYFTTDR
PHPRGELLVKSETLFPGYYKRPEVTAEMFDPDGYYRTGDVVAETGPDQLVYLDRRNNVQKLSQGEFVTVSKLEAVFGDSPRVRQIYVYGNSARSYLLAVVVPTEDVLGRDD
AKALVAESLQNVARAAGLQSYEIPRDFLIEPTPFTLENGLLTGIRKLARPRLKEHYGEQLEALYAELADGQADEMRTLRADGANRPMLETVGRAAAALLGTAATDVQPDAH
FTDLGGDSLSALTFGNLLRDIFDVEVPVGVIVSPATDLASLAAYIEAQRQPGAKRPTFTAVHGAGATEARASDLTLDKFIDAETLSAAPSLPGPNTEVRTVLLTGATGFLG
RYLALDWLERMDLVDGKVICLVRAKDDDAARARLDATFDSGDETLLAHYRELAADHLEVLAGDKGEADLGLDPQVWQRLADTVDLIVDPAALVNHVLPYSELFGPNAVGTA
ELIRLALTTRQKPFAYVSTIGVGAGIEPGKFVEDGDIRQISAVRQIDESYANGYGNSKWAGEVLLREAHDLCGLPVSVFRCDMILADTTYAGQLNLPDMFTRLMFSLVATG
VAPESFYQLDADGQRPRAHYDGLPVEFIAEAISTLGAAVKSGFETYHVMNPYDDGIGLDEFVDWLIEAGYPVHRVGDYATWLARFTAAINALPERKRQASLLPLLHNYQRP
EIPIPGSIAPTDRFRTAVQEAKIGPDKDIPHITPAVIVQYVSNLELLGLL
>Mycobacterium_chlorophenolicum
MPTETRDERLARRIADLHATDPEFAAATPDDAISETIDQPGVRLPQIMATVLDGYADRPALGQRAVRFVIDPQTGRTSADLLPRFETITYAELSARVHAVMNTLTDVAPGD
RVALLGFTSVDYTVIDMALALSGAVSVPLQTSAPAATLRPIIAETEPVIIASAVDHLADAVELAREADTVRRVIVFDHRAEVDDHRDAVADARTRLTEGGRAIEVLTLAEV
LEHGATLPAAQPFSSPEQDPLTLLIYTSGSTGAPKGAMYPERLVAGAWLRSGRSTWYGEHATPSITLNFLPMSHMMGRGLLYGTLGAGGTAYFAARSDLSTFLEDLALVRP
TQLTFVPRIWDTIFAEVAKELERRPDGEAEVYADLRQSMLGGRYVMSMTGSAPLSPEMRTFVEAFLDLHLIDGYGSTEAGAVFVDGEVQRPPVIDYKLVDVPDLGYFTTDR
PHPRGELLVKSETLFPGYYRRPEITAEMFDEDGFYRTGDVVAETGPDRLVYLDRRNNVLKLSQGEFVTVSKLEAVFGDSPLIRQIYIYGNSSRSYLLAVVVPTEDVLGRDD
AKSLIGESLQDVAKAAGLQSYEIPRDFLIETTPFTLENGMLTGIRKLARPKLKERYGDQLEALYTQLAEGQADELRALRRDGATRPVVETVGRAAGALLGTAATDVQPDAH
FTDLGGDSLSALTFGNLLRDIFDVEVPVGVIVSPANDLAALAAYIETQRQPGTKRPTFASVHGADATEAHARDLTLDKFIDAETLAAAPSLPGPSSEVRTVLLTGATGFLG
RYLALEWLERMDLVDGTVICLVRAKDDDAARARLDATFDTGDEKLLAHYRALAADHLEVIAGDKGEVDLGLDPAVWQRLADTVDLIVDPAALVNHVLPYSQLFGPNAVGTA
ELIRMALTTRQKPFAYVSTIGVGAGIEPGRFVEDGDIRQISATRRVDDSYANGYGNSKWAGEVLLREAHDLCGLPVSVFRCDMILADTTYAGQLNLPDMFTRLVFSLVATG

VAPESFYQLGDDGKRPRAHYDGLPVEFIAEAISTLGAAVETGFETYHVMNPYDDGIGLDEFVDWLIEAGYPVQRIGDYATWLQRFTAAINALPERKRQASLLPLLHNYQHP
EFPVRGSIAPTDRFRTAVQEAKIGPDKDIPHVTREVIVKYVTDLELLGLL
>Nocardia_asteroides
MTVEVDADRLADRIRALYAQDAQIRAATPIPEAHARVTTPGTPLARIVSTVMTAYADRPALGVRRTELVVEAGRATRRLLPEFELLTYGEVWERARALAASWYAEGLAAGE
FVATLGFTGADYTVLDLATIHLGAVAVPLQAGASATQLRSILDETAPRVLAVDTANLAVALDVVLAGAAPRALVVFDHHADDDNDREVLAAARARLRAANSPIVLSTVAEV
IDRGRLADPAPLVPAPQDDPLAMLIYTSGSTGTPKGAMYTDRLVAAGWQPARPVAVLNVNFLPMSHIAARLTLNGVLARGGTAYFTAAADMSTLFEDIALVRPTEIFLVPR
VCDMLLHRFRREVDRRADAGVDPEVLAEEVRGELRERVLGGRLLTVLCGSAPIAPELRRFVESVLRLRLHDGYGSTETGGVIFDTKVMRPPVLDYKLVDVPELGYFSTDKP
YPRGELLLKTTTMISGYYRRPEVTAQVFDEDGFCRTGDVVAELGPDRVAYVDRRNNVLKLSQGEFVTVSRLEAVFAGADLVRQIYVYGSSERAYLLAVIVPTEAALAGPAA
SLRAELGASLQRAAVAAELEPYEIPRDFLIESVPFSVDNGLLSGVSKLLRPALKQRYGARLDALYDEVVREQEAELERLRREAPMLPVDEVVARAALAVLGCAAADLRPSA
RFGELGGDSLAALTYSTLLRDLLGVEVPVNVLLGPDSDLAGIAGYVRREREPGARRVDVETVHGVGATEIRASELTLAKFLDPALLDAAVGLEPAADPARTVLITGANGYL
GRFLLLAWLERLAPVGGRVICVVRGADVTAARARLDAVFDTDPSLRAHVEALSGALEVVAGDIGEPRFGVDDATWRRLAGEVDLIVHSAALVNHVLPYAQLFGPNVVGTAE
VIRLALTGRRTPVSYLSTVAVAAQGESFAEDGDVRTMSPVRRLDGSYANGYGNSKWAGEVLLREASDRFGLPVAVFRSDMILAHSEYPGQVNVPDVFTRLLLSLLVTGIAP
KSFYRTDSQGRRQRAHYDGLPADFVAAAVTALGAAATTGYRTFDVVNPHDDGISLDVFVDWLIEAGHRIDRIDDFATWSARFEAALRALPEERRKHTVLPLMHAYRRPGVP
VAGSALPADGFRAAVRAAGVGADADIPHLGPELMRKYVGDLGALGLL
>Mycobacterium_heckeshornense
MSIDTREEQLERRIAELAANDPQFAAARPDPAVAAALEQPGLRLPQVIQTVLEGYADRPALGQRAVEVVKDPTTGRTSARLLPRFDTITYRELTDRVDALASAWAHEAVSP
GDRVAILGFTSVDYTTIDVTLARIGAVSVPLQTSAAVDRLRPIVAETEPRVIAASVAYLSDAVELILTGHAPARLVVFDYHREVDDHREALDAARGRLAGHSVPVETLAEV
LERGRALPAPTVAAEDDDLALLIYTSGSTGTPKGAMYPQRNVAKMWQRSSRNWFGPSAASITLNFMPMSHVMGRGILYGTLGNGGTAYFGATSDLSTLLEDLALVRPTELN
FVPRVWDTLHAEFLARVDRLTAEGADRATAETQVMDDLRDHLLGGRAIFAMTGSAPISAELKAWVESLVGIHLLDGYGSTEAGMVLYDGVVQRPPVIDYKLADVPDLGYFS
TDRPFPRGELLLKTENMFPGYYQRPEITADVFDDDGYYRTGDVVAEVGPDRLVYVDRRNNVLKLAQGEFVTVAKLEAVLGNSPLVRQIYVYGNSAHPYLLAVVVPTEDALA
RPDRDELKRRIAESLQRVAKDAGLQSYEVPRDFLIETEPFTVENGLLTGIRKLAWPKLKERYGERLERLYAELDRNQADELSELRRGGAERPVLETVTRAAGALLGAAVSE
LQPDVRFTDLGGDSLSAVTFGSLLREIYDVDVPVGVIVSPASDLQAIAGFIAAALADGSKRPTFASVHAGAAVEVHARDLTLDKFLDAHTLATAPTLPGPGTEVRTVLLTG
ATGFLGRYLALEWLERMNLVDGTVIALVRAKDDASARERLDRTFAGDPKLRAHYRELAADHLEVVAGDKSEANLGLDQPTWQRLADTVDVIVDPAALVNHVLPYRELFGPN
VLGTAELIRLALTTKIKPYTYVSTIGVGDQIEPARFTEDADIRVISPTRRISDAYANGYGNSKWAGEVLLREAHDLCGLPVTVFRCDMILADTTYAGQLNLPDMFTRLMLS
LVATGIAPRSFYELDAEGNRQRAHYDGLPVEFIAAAISTLGTPAPAGYQTYHVMNPHDDGIGLDEYVDWLIESGYPIQRVDDYADWLRRFETAMRALPDRQRQYSLLPLLH
NYQKPEKPIRGSMAPTDRFRAAVHEKKIGPDHDIPHVTREIIVKYATDLQLLGFL
>Mycobacterium_lepromatosis
MNTQEEQLARRVDYLTANDPQFAAAKPDPAVVAALAQPGLRLPQIIQTTLDGYAERPALGQRVVEFIKDPKTGRTSIELLPCFETITYRELSDRVGALARAWIHDLLHAGD
RVCVLGFNSVDYAIIDMALGVISAVAVPLQTSAAITQLQPIVIETEPRVIAASVNQLPDTVELILSGPAPAKLVVFDYHPEADEQRDAVATARERLVDNNVVVESLIEVLD
RGKTLPATPIPVADSDDPLALLIYTSGSTGAPKGAMYPQSNVGKMWRRSDGNWFRSTAASITLNFMPMSHVMGHGILYGTLGNGGTAYFAARSDLSTLLEDLKLVRPTELN
FVPRIWETLYDECQHAVDRRLTNSADRAAIEAEVMDEQRQSLLGGRYIAAMTGSAPTSPELKHGVESLLEMHLLEGYGSTEAGMVLFDGEVQRPPVIDYKLVDVPDLGYFS
TDQPYPRGELLLKTQNMFPGYYKRPEVTATVFDGDGYYQTGDIVAEVGPARLVYVDRRNNVLKLAQGQFVTVAQLEAAFSNSPLVRQIYIYGNSAHPYLLAVVVPTEDALA
TNDIEALKPLIIDSLQEVAKEAELQSYEVPRDLIIETTPFSLENGLLTGIRKLAWPKLKQHYGARLEQLYADLAEGQTNALHALRQSGAEAPVLQTVSRAAGTILGAATTD
LPSNAHFTDLGGDSLSALTFSNLLRELFNIDVPVGVIVSPVNDLAAIADYIESEQQGTKRPTFTAIHGRDAGEVHASDLTLDKFIDVSTLTAAPMLAQPDAEVRTVLLTGA
TGFLGRYLALKWLERMDLVDGKVIALVRAKSNEEARARLGKTFDSGDPKLLAHYQELAADHLEVIAGDKGEVELELDRQTWRRLADTVDLIVDPAALVNHVLPYSELFGPN
ALGTAELIRIALTSKQKPYIYVSTIGVGDQIEPAKFTEDSDIRVTSPTRKINDNYANGYGNSKWAGEVLLREAHELCGLPVAVFRCDMILADTSYAGQLNVPDMFTRMMLS
LAATGIAPGSFYELDAESNRQRAHYDGLPVEFIAEAISTLGAQSLDGFATYHVMNPHDDGIGMDEFVDWLIDAGCPIQRINDYDEWLRRFEISLRALPERQRHNSLLPLLH
NYQKPEKPLNGSLAPTDRFRTAVQESKIVQDKDIPHISSAIIGKYVSDLQLLGLL
>Mycobacterium_heraklionense
MSTVADEEQLARRIADLVATDPQFAAARPDPAVAAAVEGQSRLAQIARTVFDGYAERPALGQRAVEYVTDPATGRTAAALLPHYETITYAELGERVRAAAAALVAGAVRPG
DRVAVLGFTSVDYTVIDVALGQIGAVSVPLQTSAAISTLVPIVTETEPRVIAASVDSLPDAVELVRTGPAPTRLVVFDYHEQVDDHREALADARARLAGVEVVVEPLADLL
TRGRSLSLPQVDSGDDADPLALLVYTSGSTGAPKGAMYPERNVARMWVRSTKNWFGPTAVSITLNFMPMSHIMGRGILYGTLGNGGTAYFSARSDLSTFLEDLALVRPTEL
NFVPRIWEMLYQHFRSEVDGLGGDRAAAEAQVLAQMRTELLGGRCVFAMTGSAPISDELRSWVDQLTEQHVLNGYGSTEAGMVLFDGEVQRPPVLDYKLVDVPDLGYFGTD
TPHPRGELLLKTENLFPGYYKRPEVTAEVFDADGYYRTGDVVAEVAPDHLVYVDRRNNVLKLAQGEFVTLAKLEAVFGNSPLVRQIYVYGNSAQPYLLAVVVPTPEAQARF
DSAALKAGIAESLQQVAKDADLQSYEVPRDFIVETEPFTLENGLLTGIRKLAWPKLKAHYGDRLERRYAELAEGQASELSELRQHGAQRPALETVGRAAAALLGASAGEVS
ADAHFTDLGGDSLSALTFGNLLRDIFGVDVPVGVIVSPANDLAAIADYITAERNGSGRPSFASVHGRDAVEVAAADLTLDKFLDAATLAAAPSLPGPSEQVRTVLLTGATG
FLGRYLALEWLQRLAPVDGKLICLVRAKSDEDARRRLDSVFDSGDARLLARYTELAAKHLEVLAGDKGEANLGLDQEVWQRLADTVDVIVDPAALVNHVLPYSELFGPNAL
GTAELIRVALTGKIKPFTYFSTIGVGDQIPLGKFTEDADVRVMSSVRAINDGYANGYGTSKWAGEVLLREANDLCGLPVAVFRCDMILADTSYAGQLNLPDMFTRMMFSLV
ATGVAPGSFYELSADGQRQRAHYDGLPVEFIAEASTTLGSFASGGYTTYHVMNPYDDGLGMDEFVDWLIEAGYPIRRIADYGDWLQRFETTLRALPEHQRQASLLPLLHNY
QKPQPPICGSIAPTDRFRAAVQSAGIGPDPDIPHVTPAVIVKYITDLQLLGLL
>Mycobacterium_setense
MSFDTREEQLASRIADLTANDPQFAAAIPSDTVTAAVDVPGLLLPEIVQTLLQGYAERPALGERALEFVVDPATGRTVARLLPRFDTISYGELWGRVSALGAAFHASGVAA
GDRIAILGFTSADYTVIDTALSQIGAVSVPLQTSSTPEALAPIVAETEPRVIAASVDHLADAVELALSAHAPASVLVFDHHPEIDDHRDAVAAAAARLREAGSSVAVDTLA
DLLDRGRELPAPPVPEAEDTDPLALLIYTSGSTGAPKGAMYLQSAVAKFWRRNSKAWLGPVSSAINLSFMPMSHVMGRGILYASLAAGGTCYFAARSDLSTLLEDLALVRP
TELNFVPRVWEMIHSEYQTRVDQRLAEAQDRAAVEAEVLAEVRDNVLGGRFVAAMTGSAPISAELKAWTEDMLGIHLLEGYGSTEAGMALFDGVVQRPPVIDYKLVDVPDL
GYFATDQPHPRGELLIKTENLFPGYYKRPEVTASVFDEDGFYRTGDVVAQIGPDQLRYVDRRNNVLKLAQGEFVTLAKLEAVFGNSPLVQQIYVYGNSAQPYLLAVVVPTD
ADVSKQEISDSLQEVAREADLQSYEIPRDFIVETTPFSVENGLLTGIRKLAWPKLKAHYGDRLEQLYVELAETQANELRALRSGAADAPVLETVSRAAGALLGAAAADLEP
DAHFTDLGGDSLSALTFGNLLREIFDVDVPVGVIVSPATDLAGIADYIETQRNGSKRPTYASVHGRHAAEVSASDLTLDKFLDEATLAAAPGLPKAPTEVRTVLLTGATGF
LGRYLALEWLERMDLVDGKVIALVRAKSDEEARARLDSTFDTGDAKLLAHYRKLAAEHLEVIAGDKGEENLGLDQQTWQRLADEVDLIVDPAALVNHVLPYSELFGPNALG
TAELIKIALTTKIKPYTYVSTIGVGDQIEPGKFVENVDVRQMSAVRKVNDGYANGYGNSKWAGEVLLREANDLCGLPVAVFRCDMILADTTYSGQLNLPDMFTRMMFSLVA
SGIAPKSFYELDADGNRQRSHYDGLPVEFIAESISTLGGQSVESFETYHVMNPYDDGLGMDEFVDWLAEAGYAIERIADYGQWIQRFESTLRALPDKQRQASLLPLLHNYQ
KPEKPMLGALAPTDHFRAAVQEAKIGPDKDIPHVSPAIVVKYITDLQQLGLL
>Mycobacterium_rufum
MPTETREERLARRIADLHATDPEFAAATPDDAISTAIDQPGVRLPEIMATVLDGYADRPALGQRAVRFVTDPQTGRTSADLLPRFETITYAELSARVHALMNTLTDVAPGD
RVALLGFTSVDYTIIDMALALCGAVSVPLQTSAPAATLRPIIAETEPVIIASAVDHLADAVELAREADTVRRVIVFDHRAEVDDHRDAVADARARLTDGGRAIEVLTLSEA
LELGATLPAAQPFSSPEADPLTLLIYTSGSTGAPKGAMYPERLVAGAWLRSGRSTWGGEQATASITLNFLPMSHMMGRGLLYGTLGAGGTAYFAARSDLSTFLEDLALVRP
TQLTFVPRIWDTIFAEVAKELERRPDGQDEIYADLRTSMLGGRYVMSMTGSAPLSPEMRTFVESFLDLHLIDGYGSTEAGAVFVDGEVQRPPVIDYKLVDVPDLGYFTTDR
PHPRGELLVKSETLFPGYYRRPEITAEMFDEDGYYRTGDVVAETGPDRLVYLDRRNNVLKLSQGEFVTVSKLEAVFGDSPLVRQIYIYGNSSRSYLLAVVVPTEDVLGRDD
AKSLIGESLQDIAKAAGLQSYEIPRDFLIETTPFTLENGMLTGIRKLARPKLKERYGDRLEALYTELAEGQADELRALRQDGATRPVLETVGRAAGALLGTAATDVQPDAH
FTDLGGDSLSALTFGNLLRDIFDVEVPVGVIVSPATDLAALAAYIETQRQPGAKRPTFAAVHGADATEAHARDLTLDKFIDAETLAAAPSLPGPSSEVRTVLLTGATGFLG
RYLALEWLERMDLVDGTVICLVRAKDDDAARARLDATFDTGDEKLLAHYRTLAADHLEVIAGDKGEADLGLDPAVWQRLADTVDLIVDPAALVNHVLPYSQLFGPNAVGTA
ELIRMALTTRQKPFAYVSTIGVGAGIEPGKFVEDGDIRQISATRRVDDSYANGYGNSKWAGEVLLREAHDLCGLPVSVFRCDMILADTTYAGQLNLPDMFTRLMFSLVATG
VAPDSFYRLDDDGSRPRAHYDGLPVEFIAEAISTLGTAVESGFETYHVMNPYDDGIGLDEFVDWLIDAGYPVQRIGDYATWLQRFTAAINALPERKRQTSLLPLLHNYQHP
EFPVRGSIAPTDRFRAAVQEAKIGPDKDIPHVTREVIVKYVTDLELLGLL
>Mycobacterium_llatzerense

MSIDHADSALARRIADLSANDPQFAAVVPIESVAQSVEKPGMRLPEIVKTVLEGYADRTALGQRAVEFVTENGRTVARLLPKYDTITYGELWERVRAVAAALHADGVKAGD
RVAILGFTSTDYTVIDTALGQIGAVSVPLQTSSAASSLLPIVAETEPVLIAVSAGYVADAVELAVGGPAPARFVVFDHHAEVDDEREAVEEARAALSGLNVTVETFGEVLA
RGRDLPSAPTPVFDEADPLTLLIYTSGSTGAPKGAMYPESNMTSFWRRASSAWFGPSEASINLAFMPMSHVMGRGILYSSLANGGIVYFAAKSDLSTLLDDFALARPTELN
LVPRVWEMLYLEFQSRVDKLVPAGAERDGIEQQVMADMRENLIGGRYIKAMTGSAPITDELKAWVEKFLGIHVLEGYGSTEAGMVFFDGVIQRPPTLDYKLVDVPDLGYYL
TDQPYPRGELLVKTEYLFPGYYKRPEVTASVFDEDGFYRTGDVVAEIGPDQIRYVDRRNNVLKLAQGEFVTLAKLEAAFSNSPLVRQIYVYGNSAQPYLLAVVVPTEDALA
RWDSAELKQQISDSLQEVAKAAELQSYEIPRDFIVETEPFSLENGLLTGIRKLAWPKLKAHYGPVLEDLYVSLAAGQADELRALRQHGAEGPALPTVIRAASALLGASGEV
SADAHFTDLGGDSLSALTFGNLLEEIFGVEVQVGIIVSPANDLAALAAYIENERSGGAKRPSFSSVHGKNAAEVRAADLTLDKFVDAQTLAAAPSLPGPASEVRTVLLTGA
TGFLGRYLALEWLERMDLVDGKVIALVRAKSDGEARARLDATFDSGDPKLLAHYQALAADHLEVIAGDKGDENLGLPQEVWQRLADEVDVIVDPAALVNHVLPYSELFGPN
ALGTAELIRIALTTRQKPYIYVSTIGVGDQLAPGTFTEEADVRAMSPVRAVHDGYANGYGNSKWAGEVLLREANDLCGLPVSVFRCDMILADTSYAGQLNLPDMFTRMMFS
LVATGIAPYSFYERDDEGKRQAAHYDGLPVEFIAEAIDTLGVRPAGEFVTYHVMNPHSDGLGMDEFVDWLIDAGYSIARVDDYAEWLARFETTLRALPDRQRQASLLPLLH
NYQKPEHPHNGSIAPVVVFREAVQDAKLGPDKDIPHVTAPVIVKYITDLQLLGLL
>Mycobacterium_cosmeticum
MTNDVSVERFQRRLTHLYDTDPQFAAAAPSDEVIAAVDDPGLRLPAVVKTVLQGYGDRPALGQRAVEFVTDPATGRTTAALLPRFETISHKQLWDRVQAIANAWHDHPVRI
GDRVAILGFTSVDYTTVDTALTQLGAVSVPLQTSATVAALQPIVAETEPVLIATSIDYLDDAVALAESAQTAHLVVFDYHPEVDDQREAVAAAQARLGSGVSVEPLADVLE
RGAALPDIEGVVGPDDDPLALLIYTSGSTGAPKGAMYLESKVTNIWRPAANAHWDDSGKVPSIVLSFMPMSHVMGRGILYGALASGGTVNFAARADLSTFLDDLALTRPTQ
LNFVPRIWDMLFQEFQSRTSHGADEDTVLADMRENLLGGRYVSALTGSAPISPELKAWVERLLEQHLLEGYGSTEAGSVFVDGRITRPPVLEYKLVDVPELGYRLTDVPHP
RGELLVKSTQLFPGYYKRPEITAQMFDEDGFYRTGDIVAELGPDEVRYVDRRNNVLKLAQGEFVTVSKLEAAFTNSPLIRQIYIYGNSARPYLLAVVVPTDPTVSKADIAQ
SLKDTARAADLQSYEIPRDFLIETTPFTVENGLLTGIRKLAWPKLKERYGAELEGLYAELADGQANELQALRQHGADAPVLQTVSRAAVALLGAASSDVAPDAHFTDLGGD
SLSALTFGNLLQEIFGVEVPVSVIVSPTSDLATIAAHIETQRAGGDKRPTFSSVHGAGATEVHAADLTLDKFIDAATLAAAPALPGPDAEIRTVLLTGATGFLGRYLALEW
LERMDLVDGKLICLVRARSNEEARARLDKTFDSGDPKLLAHYRELAADHLEVIAGDKGEADLGLDKATWQRLADTVDFIVDPAALVNHVLPYSELFGPNALGTAELIRIAL
TTKIKRFAYVSTIGVGWGIEPGKFVEDADIRTISATRVVDESYANGYGTSKWAGEVLLREAHDLAGLPVSVFRCDMILADTSYAGQLNLPDMFTRMMFSLVATGVAPKSFN
QLDAQGNRQRAHYDGLPVEFIAEAIATLGAHVDDGFETYHVMNPYDDGIGMDTFVDWLVEAGYKIERVDDYAEWLDRFETTLRALPDKQRQASLLPLLHNYQQPGVPVNGA
MAPTDVFRAAVQEAKIGPDKDIPHVSAPVIVKYITDLQLLGLL
>Nocardia_testacea
MSIDTRESPLEHRIAELYRTDRQFAAARPDPAVTARAEKQGARPVEVAETVMQGYARRPAVGQRAIEFVTDPQTGRTSVALLPRFDTLTYGEVWDRAGALAAALSGDPVRP
GDRVCVLGFSSVDYTLVDLALVRLGAVAVPLQTSAPVTQLRSVVAETEPTVLAASIDHLDVAVDLVRTGPGAARLIVFDYHPQADDQRAAFEAVVARLSAAGSPVRVDDLS
EVIAHGATLAPPPVPPPAETDPPALLIYTSGSTGAPKGAMITERLVADQWRAATSERWGQPATEPAIALGFMPMSHIMGRAILYMTLARGGTVFFAARSDLSTLLDDLALV
RPTQLVFVPRIWEMLYQRFRGEVQRRTAAGADPATVEEEVRTELREKLLGGRFLAATTGSAPISRELRTWVETLLGFHLVDGYGSTEAGSIAVDGRIRRPPVLDYELVDVP
ELGYFHTDRPYPRGELVIRSEALIPGYYKRPDATAEVFDADGAYHTGDIFAEVGTDELVYLERRGFVLKLSQGEFVAVSTVEAALAESPLVRQVYVYGNSTRSYPLAVVVP
TAEAIEQGDIEALRPLLAESLRQVAKTAGLQSYEIPRDLLVETRPFTPENGLLTGIGKLARPALKQRYGPRLEQLYADLAAAEAEELRALHRSGDRPTVDTVVRAAGALLG
SATGDLRPDAHFTELGGDSLSALTFAQLLREIFDIEVPVGTIIGPATDLRALADYIDGQRRGGHRPTFASVHGAGATEVYARDLTLDQFIDAGTLAAAPGLPEPSAPARTV
LLTGATGFLGRYLVLEWLRRLNHSGGTLICPVRAPDDAAARARLDEVFETGDPALSARYRESAAGHLEVIAGDKSAPRLGTTRAVWERLADTVDVIVDPAALVNHMLPYRE
LFGPNVVGTAELIRLALTTRQKPYTYISTIGVSDQIDRAAFTEDADIRVISPRRAIDGGYANGYSNSKWAGEVLLREAADLCGLPIAVFRCDMILADTEYSGQLNLPDMFT
RLVLSVEATGTAPGSFYELDAEGRRPRAHYDGLSVGFIAEAVATLGARPGSGFRTYHVMNPYDDGISLDTYVDWLIDAGHPIERISGYDDWLQRMETAVRALPDRQRRYSL
LPLLDNYRKPQQAVRGSIAPTERFRDAVRHAEIGAEKDIPHVTPDIIVKYTTDLERLGLL
>Mycobacterium_triplex
MSTTTREERLERRIENLTATDPQFAAAKPDPAVVEALEQPGLQLPQIIQTVLEGYGDRPALGQRAVEFVKDAKTGRTVLGLLPRYDTITFGELRERVDAVARALTRDGLRP
GDRVAALGFNSVDFTTIDIALGMVGAVSVPLQTSAAVAQLQPIVTETEPAVFAASTNQLSDAVELILSTHRPTKLVVFDYHPEVDDEREAVESARARLADTALTVEPLADL
LQRGATLPATPAAAVDDDELALLIYTSGSTGAPKGAMYPRRNVGKMWRRSGRNWFGETVASITLNFMPMSHVMGRGILYGTLGNGGTAYFAARSDLSTLLEDLELVRPTEM
NFVPRIWETLYGEYQRRVDRGGSEADVMNEISQHLLGGRFIFAMTGSAPTSGDLKAWVEELLDMHLLDGYGSTEAGMVLFDGEVQRPPVIDYKLVDVPDLGYFATDRPYPR
GELLLKTENMFPGYYKRPETTAGVFDPDGYYRTGDVVAEIAPNKVVYVDRRNNVLKLAQGEFVTVAKLEAVFGNSPLVRQIYVYGNSAHPYLLAVIVPSEEALERYGADEL
KGRIADSLQTVAKEAGLQSYEVPRDFLIETTPFTLENGLLTGIRKLAWPKLKQQYGERLEQLYADLAAGQANELGELRRSGATAPVLQTVSRAAAALLGAASTELTPDAHF
TDLGGDSLSALTFGNLLHEIFEVDVPVGVIVSPANDLAAIAAYIESERQGSKRPSFASVHGRDAVEVHASDLTLDKFIDAATLAAAPNLPAPASEVRTVLLTGATGFLGRY
LALQWLERMDLVDGKVIALVRAKTDADARARLDKTFDSGDPKLLAHYRELAADHLEVIAGDKGEADLGLDAATWRRLADTVDLIVDPAALVNHVLPYSELFGPNAVGTAEL
IRVALTGKKKPYTYVSTIGVGDQIKPGAFVEDADIREISATRQINDSYANGYGNSKWAGEVLLREANDLCGLPVAVFRCDMILADTSYAGQLNVPDMFTRMMLSLVATGIA
PGSFYERPAEGNRQRAHYDGLPVEFIAEAVSTLGAQNVQSFQTYHVMNPYDDGIGMDEFVDWLIADGNAIQRIDDYGDWLPRFETALRGLPEKQRNASLLPLLHNYQKPEQ
PIRGSMAPTDRFRAAVQDAKVGPDKDIPHIGAPIIAKYVSDLRLLGLL
>Nocardia_vulneris
MTTETREDRLQRRIAQLYETDSQFADARPSDAVNAAVAQPELRLPAVVKGIFAGYADRPALGQRAVELVTDAAGRTSARLLPRFDTITYRQLGDRVQAVTNAWHNHPVKPG
DRVAILGFTSVDYTTIDTALIELGAVSVPLQTSAPVTTLRPIVTETEPTVIAASIDFLDDAVELVRSGPAPHRLVVFDYQPKVDAQRETFEAAKTALAGTGVIVEPLADVL
DRGRSLADAPLYTPGQADPLTMLIYTSGSTGTPKGAMYPESKVANMWQLASKATWDENAILPAITLNFMPMSHVMGRGILIGTLSSGGTAYFAARSDLSTFLDDLALVRPT
QLSFVPRIWDMLFQEYQSRLDRSGSPEDEVLAEVREDLLGGRFVSAMTGSAPISAEMKTWVERLLDMHLLEGYGSTEAGSVFVDGQIQRPPVIDYKLVDVPDLGYFRTDQP
HPRGELLVKSEQMFPGYYKRPEITAEMFDEDGYYRTGDIVAELGPDHVEYLDRRNNVLKLSQGEFVTVSKLEAVFGDSPLVRQIFIYGNSARSYLLAVVVPTDPAVSKQAI
SDSLQDAARAAGLQSYEVPRDFIVETTPFSLENGLLTGIRKLARPNLKSHYGERLEQLYAELAEGQANELSELRRSGADAPVLDTVSRAAGALLGAAASDLAADAHFTDLG
GDSLSALTFSNLLHEIFDVDVPVGVIVSPATDLAGIAGYIEGQRHGSKRPTYASVHGRDATEVHAADLTLDKFLDADTLAAAPSLPKAPAEVRTVLLTGATGFLGRYLALE
WLERMDLVDGKVIALVRAKNDAEARARLDATFDVGDPKLLAHYQDLAADHLEVIAGDKGEADLGLDHSTWQRLADDVDLIVDPAALVNHVLPYSQMFDANALGTAELIRIA
LTTKIKPFVYVSTIGVGWGIKPGEFVEDADIRVISPTRQVDDSYANGYGNSKWAGEVLLREANDLCGLPVSVFRCDMILADTTYSGQLNLPDMFTRMMLSLVATGIAPGSF
YELDSDGNRQRAHYDGLPVEFIAEAISTLGAHVTEGFETYHVMNPYDDGLGLDEFTDWLIEAGYPVHRIDDYGQWLQRFETALRTLPDKQRQASLLPLLHNYQKPSQPLLG
AAAPTDRFRAAVQEAKIGPDKDIPHVSPAVIVKYITNLQMLGLL
>Nocardia_brevicatena
MSVDTRESRLERRIAELYATDEQFAAARPDEAVTAAIEQSGLRAARVAQTVMEGYAHRPAVGQRAVEFVTDPGTGRTSTELLPRFETLTYREVWDRAGAIATALTGDPVRP
GDRVCVLGFTSVDYTIIDMALMRLAAVAVPLQTGASAERLRPIIAETEPTVLAASIDYLDDAVELVLTGHAPTRLIVFDYRPRVDDQREAFAAATAKLADAAGPVIVETLA
DVVDRGTGLPPVPVPTTDEPDSLALLVYTSGSTGAPKGAMITDGMMAEYWRASTSERWGQRSAEPSIVLSFMPMSHILGRAVLYMALGRGGTVYFAAKSDLSTLLDDLASV
RPTQLTFVPRIWEMLFQRFQSEMYRRASDGGDRATLEAEVTADLRRDLLGGRFLSATTGSAPMSTEMKAWVETFLDLHLRDGYGSTEAGSITLDGHVRRPPVVDYKLVDVP
ELGYFRTDRPYPRGELAVVSQSLTPGYYKRPDVTAEVFDADGFYHTGDIFAEVGPDQLVYVDRRSFVLKLSQGEFVTVSKLEAVFAESPLVRQIYVYGNSTRSYLLAVVVP
TEDAQARGDIEALKPLIGESLRKVAKTAGLQSYEIPRDLILDTTPFTMENGLLTGSRKLARPKLEEYYGPRLEQLYTELAEAETHELRTLRHSGAGRPVLETVSRAAGTLL
GAAAGVLRPEAHFTDLGGDSLSALTFANLLRDIFDVDVPVGVIVGPATDLRALAAYIEQRRGGTERPTFTAVHGDGATELHARDLRLDKFLDTQTLEAAPNLPGPSPKVRT
VLLTGATGFLGRYLALEWLQRMALVGGRLICLVRAKDDATARERSDNTFAGSDPRLLAHYREPAAEHLEVLAGDKGEADLGLDRATWQRLADTVDLIVDPAALVNHVLPYS
QLFGPNVVGTAELIRLALTTKQKPYTYVSTIGVSDQIDPSVFTEDADIRAIGPTRAIDDSYANGYSNSKWAGEVLLREANDLCGLPVAVFRCDLILADTEYAGQLNVPDMF
TRLMLSLLATGIAPGSFYELGPDGRRRRAHYDGLPVGFVAEAISTLGAQAAPGFRTYHVMNPYDDDISLDTYVDWLIDAGHPIERIPDYGTWLRRFETAVRALPDRQRRHS
LLPLLHSYRQPQRPIRGSIAPTERFRAAVQEAKIGPDKDIPHVTREIILEYATDLRQLGLL
>Nocardia_araoensis
MAPETRRERLERRITRLYAEYDDIRNATPRADVAKKVREPGIGLAQIVETVMVGYADRPALGQRATEPRTDETGRTTLTLLPRFDTITYGELWDRVRAVAAAWHGDPLRAG
DFVGILGFTSSDYTTLDLACIHLGLVAVPMQSSAPIAQLSAIVAETEPRVLAATPELLDAAVTCALSDSGPKRLIVFDYHPEDDDQRAAFEAARARLAEAGSPVRVESLDE
VLTRGRSLPPAPLFVEPDADPLALLIYTSGSTGTPKGAMYSRRLVARGWVNRPEVAAINFNYMPMSHVAGRGSLTGVLARGGTAYFAAKSDMSTLFDDLALARPTELFFVP
RVCDMVFQRFQSEMDRRDGPGVDRAALEAEVKAELREHFLGGRMVTAICGSAPVSAEMKAFVESVLDLELHDGYGSTEAGVVVIDERVQRPPVLDYKLADVPELGYFRTDK

PHPRGELLLKSTTLISGYYKRPDITAEVFDSDGFYKSGDIVAELAPDRLVYVDRRNNVLKLSQGEFVAVARLESVYATSPLIRQIFVYGSSERSYLLAVIVPTEDVLAWGD
PEKMKTALSESLRQLAKDAELQSYEIPREFLIEPEPFSTANGLLSGIGKLLRPKLKERYEPRLQELYAELAAGQANELLDLRHTADDLPVLETVSRAAKAVLGCADADLRP
DARFGDLGGDSLSALSLSNLLHEIFEIEVPVSVVISPANGLREVADYIAAERASGARGATYASVHGAGSQVHAADLTLDKFIDARTLAAAPTLPRPSATPRTVLLTGANGY
LGRFLCLEWLRRLHDSGGTLVCLVRGSDADAARKRLDSAFDSGDPGLVREFQDLAAAHLEVLPGDIGDPNLGLDDRTWQRLAEQVDVIVHPAALVNHVLPYRQLFGPNVVG
TAELIRLALTTRLKPFTYLSTVAVATQIDPAVFAEDGDIREISAVRAVNDSYANGYGNSKWAGEVLLREANDLCGLPVTVFRSDMILADRRYAGQLNVPDIFTRLLLSLLV
TGIAPFSFYRTGANGERARAHYDGLPADFTAEAITTLGAATGTGFHSFDVLNPHDDGRSLDEFVDWLIDAGHRIERIADYDEWVTRFETALRALPEGLRQASVLPLLHAYR
RPAPAVRGSALPAEKFRAAVRAAKIGADQDIPQISRDLIEKYVADLHVRGLL
>Nocardia_transvalensis
MEITDAQAQLIRRATELIEGDEQVRAALPDEAVAKAVQAPGLGLASVVATIMEGYADRPAAGRRAVEFVADDSGRRHARLLPRYDTITYGELWERVRALMAAWHHDPTRAG
DFVAILGFTGIDYTVVDLACACLGAVSVPLQAGASLAQLTPIAAETEPRVLATDIEQLGAGVDLVLSGDSVRSVVVFDYYAEDDDHRAALESARARLADSPVTVDTLDEVL
ARGRDLPAAPLHTDGDEDELSLLIYTSGSTGTPKGAIYPARLLTRMWRSGGDRPMPVLGFSYMPMSHVAGRGSLISALSLGGTVYFAARSDMSTLFDDITLCRPTMVFFVP
RVCDMVFQRYRSEVDRRLAAGGDREQVEREVKTELREHFLGGRFLLALVGSAPLSPEMRAFMESVLEIGMFDGYGATETGGVLLNNELQRPPVLDYRLEDVPELGYFGTDK
PYPRGELLLKSETLVPGYYKRPELNAEIFDADGFYRTGDVMAEIGRDRLVYVDRRNNVLKLSQGEFVAVSKLEALYTSSPLVRQIYVYGSSERAYLLAVIVPTDDAVTAPE
PAELKAAIGESLQRVARDAELESYEIPRDFLVEPEAFSMANGLLSGIGKLLRPKLKQRYGDRLEERYDELSREQQDELTALRTAAADLPVLETVSRAAKALLGCATTDLRP
DAHFADLGGDSLSALSLSTLLQEIFAVEVPVGVIVGPATDLRRLAEYIETERASGGTRPTAASVHGAGTEIRAADLTLDKFIDAETLAGATALPRAPRSPDTVLLTGANGY
LGRFLCLEWLRRLHDSGGTLICVVRGSDAAAARARLDEVFDSGDPELLSHYRELAEGTLEVLAGDIGEPNFGVAEADWRRLADSVDLIVHPAALVNHVLPYDQLFGPNVVG
TAEVIRLALTARLKPVTYLSTVGVADQVNPQVFTEDGDIREISGVRSLGDGYANGYGNSKWAGEVLLREAHDLCGLPVAVFRSDMILAHSRYGGQFNLPDMFTRLFLSVLA
TGVAPKSFYEADAEGNRQRAHYDGLPADFTAEAITTLGMRVGSGYETFDVLNPHDDGLGLDEFIDWLIAAGHPIERIDDYGQWLSRFETALRALPEQQRQHSVLPLLHAYR
RPGAPIRGAMLPAKKFQAAVQEARIGAAADIPHLTPALIEKYATDLKLRNLL
>Nocardia_cerradoensis
MPDDVQKAERKRRLAAAMADDEVRAAWPDADVSAALARPGLRFAELVDTVVTAYADRPAVGQRAGEIVVDAEGRRVRRLLPRFETLSYRQLWSRAGALASAWQASGVRAGD
FVCTVGFVSSDYVTVDLAGVRLGTVAVPLQATAAVAQWVSIIAETEARVLACSAELLDAAVEAALASATIGQLVVFDSADDDDERAAVAAARSRLAESGRDITIDSLRDLL
ERGLALPAVPLASAPGDDPLALLVYTSGSTGTPKGAMYTDRLAAAMWLYTAKAPVPAITLNYLPLSHVAGRLQLGGTLARGGTAYFTARSDMSTLFEDLELTRPTELVFVP
RVCEMLLQHHQGEVEARIAAGGERGVVEDEVKTELRDRLLGGRFLGAMCASAPLAPEMRAFMESVLGIGLHDGYGSTEAGSVIVDNKVRRPPVLDYKLVDVPELGYFGTDQ
PHPRGELLLKTTTMFPGYFKRPEITAEMFDADGFYRTGDVVAELGPDHLVYVDRRNNVLKLSQGEFVTVAKLESVFSTSALIRQIFVYGSSERAYLLAVIVPTEEALTLPE
PRAALAESLQELAKEAGLNSYEIPRDFLLETEPFTQDNGLLSGIGKLLRPKLKQRYGERLEQLYAELSREQADELAALRRGAADRPVLETVGRAARALLGCASTDIRPEAH
FTDLGGDSLSALSLSNLLTELFGVEVPVGTIVHPANTLRRLAEYIDTERNEGGRRPTLTSVHGHGTWVRAADLTLDAFIDHETLTAAKSLPSASAPPRTVLLTGANGYLGR
FLCLEWLDRLDACDGTLICVIRGRDAETARARLDEAFDSGDPVLLQRYRELAQRRLTVLPGDIAEPYFGLDEDRWRELAETVDLVVHPAALVNHVLPYDQLFGPNVVGTAE
VVRLAVTARRKPVAYLSTVAVASQVDHFTEDGDIREISPERVIDTGYANGYGNSKWAGEVLLREAHDLCGLPVAVFRSDMILAHSEYAGQFNLPDMFTRLLLSVLATGLAP
ESFYRPAADGGRARGHYDGLPAGFTAAAITALGPGADSGFETFDVLNPHDDGIGLDTFVDWLVDDGHRITRIADYADWLARFETALRALPERQRRHSVLPLLHAFGRPAPA
IAGSALPAERFRAAVRAAGTGPDGDIPHLSRELIGKYVRDLTAAGLL
>Nocardia_aobensis
MPDDVQKAERKRRLAAAMADDEMRAAWPDADVSAALARPGLRLAELIDTVMTAYADRPAVGQRSGEIVVDAEGRRVWRLLPRFETLSYRQLWSRAGALASAWQAAGLRAGD
FVCTVGFVSSDYVTVDLAGVRLGTVAVPLQATAAVAQWHSIIAETEARVLACSAELLDAAVEAALASVTIGQLVVFDSADDDAERAAVAAARARLAESGREITLESLHDLV
EHGLALPAVPLAGVPGDDPLSLLVYTSGSTGTPKGAMYTDRLAAAMWLYTGKAPVPAITLNYLPLSHVAGRLQLGGTLARGGTAYFTARSDMSTLFEDLELTRPTELVFVP
RVCEMLLQHHQGEVEARVAAGRERVVVEDEVKTALRERLLGGRFLGAMCASAPLVPEMRAFMESVLGIGLHDGYGSTEAGSVIVDNVVRRPPVLDYKLVDVPELGYFGTDR
PHPRGELLLKTTTMFPGYFKRPEITAEMFDADGFYRTGDVVAELGPDHLVYVDRRNNVLKLSQGEFVTVAKLESVFSTSALIRQIFVYGSSERAYLLAVIVPSEEALTLPD
PRAALAESLQQLAKEAGLDSYEIPRDFLLETEPFTQDDGLLSGIGKLLRPKLKQRYGERLEQLYAELSREQADELAALRHGAADRPVLETVGRAARALLGCASTDIRPEAH
FTDLGGDSLSALSLSNLLTELFGVEVPVGTIVHPANTLRRLAEYIDDERHEGGRRPTLATVHGPGTRVRAADLTLDAFIDHETLTAAKSLPPASSPPRTVLLTGANGYLGR
FLCLEWLDRLDACDGTLICVIRGRDAEAARARLDEAFDSGDPVLLQRYRELAQRRLTVLPGDIAEPNFGLDDDRWRELTETVDLVVHSAALVNHVLPYDQLFGPNVVGTAE
VVRLAITARRKPVTYLSTVAVASQVDHFTEDGDIRGTSPERVIDGGYANGYGNSKWAGEVLLREAHDLCGLPVAVFRSDMILAHSEFAGQFNLPDMFTRLLLSVLATGLAP
ESFYRPAADGSRARAHYDGLPADFTAAAITALGPGADGGFETFDVLNPHDDGIGLDTFVDWLVDDGHRITRITDYADWLGRFETALRALPERQRRHSVLPLLHAFRRPAPA
IAGSALPAERFRAAVRAAGTGPDGDIPHLSRELIGKYVRDLTAAGLL
>Nocardia_concava
MVEDVRGEEVMKRFEALLADEQIRAAMPVPEVAEAARRPGLGLAGIAAVLMEGYADRPAIGERAAEIVVDADGRRTRRLLPAYVTTTYRDLWTRAGHIAASWQHDPLRAGD
FLCILGFASGDFAALELAAIRQGLVTVPLQSSAAAAQWHSIITETESRTLAVSLELLAPALECVLDGTAVTSIVVFDFEPEDDRQAEAFAAAQRRLEGTGITLESLATVVD
RGARLPEAPLHVPADENELAVLIYTSGSTGTPKGAMYPHRLAAGMWLGPTVIPAPVMNFCYMPLSHVAGRMVLNGTFSRGGTAYFAASSDMSTLFEDIALVRPTEVFFVPR
VCDMVYQRYQSETQRRIAAGESAEDADRIVKTALREEFLGGRMARVMVGSAPISAEMKEFMRSVLGQPIIDGYGSTEAGGLLIDNEIRRPPVIDYKLADVPELGYFSTDKP
YPRGELVVKSMQQIPGYFKRPEVTAEIFDEDGYYRTGDIVAEIRPDYLVYVDRRNNVLKLSQGEFVTVAKLEAVYATSPLIAQIFVHGSSERSHLVAVIVPTDAARALEPA
ERIAAITESMQRIAKENGLESYEIPRAFLLEDEPFTQDNGLLSGIGKLLRPKFKERFGERLEQLYTEQAQRQRDELAVLRREAADRPVIETVCRAARAVLGGGEPQPDAHF
TDLGGDSLSALSFSTLLAEIFGVEVPVGVIVSPANNLDRLAAHIEAERDSGGTRPTVATIHGGKEIRATDLTLDKFLDAATLAAAPALPRASQPPRTVLLTGANGYLGRFL
CLEWLQRLDGTDGTLICVIRGRDAETARRRLDEVFDSGDPELLRRYRELAQRRLRVLPGDIGEPNFGLSQQDWQELADTVDLIVHPAALVNHVLPYGQLFGPNVVGTAEVI
RLALTSTLKPVTYLSTVAVAAQIDPAVFTEDGDIREISAVRSVDDGYANGYGNSKWAGEVLLREAHDLCGLPVAVFRSDMILAHSDFAGQLNLPDMFTRLLLSVLATGLAP
KSFYALDSHGNRRRAHYDGLPADFTAAAITALGARVTEGFETYDVLNPHDDGISLDEFVDWLIESGHAIDRIDDYRDWFTRFETALRTLPEHQRGASVQPLLHAYRRPGLP
IAGSVLPAKRFQAAVQQAKLGPGGDIPHLTRELIDKYVADLKLRGLL
>Nocardia_thailandica
MARDNDAARLSQRIEALYAADDQIRAARPLPEVQERLGAPGLSVNQLITTVMTGYADRPAVGTRRRDGRTLLPDHDLLTYAQLWERAGAIAAAWHADGVRPGDFVVVIGFT
SAEYLAIDLAVTHLGAVVVPLTGGVSPGRLRAILDETEPAVLAVDHDNLPAALAATGDGGGPRSLLLFDHREDDDTHAAALAAARTAAADTGIAVRTHDEVRARGEQAPPA
PLFPEPGTDPLAMLIYTSGSTGTPKGAMYTHRLVADGWRAGRPLAAITLNYLPMSHIAARLTLISTLTRGGTAYFASAPDMSTLFDDFALVRPTEVFLVPRVCDIVFQRYR
REMDSRGTGVDEEALAERVRTELREDLLGGRLMMTICGSAPLAPEMRAFMESVLQLQLHDGYGSTEAGALLVNGYLLRPPVLDYKLVDVPELGYFATDKPYPRGELLLKTS
SMVPGYYKRPDVTAEMFDADGFYRTGDVVAELGPDRLSYVDRRNNVLKLSQGEFVTVSNLEAAYVASPLIRQIYVHGSSERAYLLAVIVPTERAAALPEARLRPALHEELR
AIARAAGLESYEIPREFLLEPEPFTIANGLLSGVAKLLRPALKQRYGGRLDQLYADLARGQEDELRALRRDAAVLPVAETVARATRAVLGCAVEDLRPDAHFADLGGDSLA
ALSYADLLRELLGVDVPVNVVLGPAGDLAGIAAYITRRRAGGARPTPASVHGDGAAEIRAADLTLERFLDHETLTAAADLPAAAPEPRTVLITGANGYLGRFLVLEWLSRP
GTRVIALVRGADAGAARDRLLAGFGDHVPAALTGADGPEVLAGDISEPDFGLPVQQWRRLAAEVDLIVHSGALVNHVLPYAQLFGPNVVGTAEVLRLALTTTRKPVAYLST
VAVAAQGESFAEDGDVRVMSPVRTLDASYANGYGNSKWAGEVLLREAADRFGLPVTVFRSDMILAHRTLPGQLNVPDVFTRLLLSVLLTGLAPASFYRTGPDGTRQRAHYD
GLPADFVAAAIVALGAHTTGHRTYDVVNPHDDGIGLDTFVDWLIEAGHPIDRIDDHAAWFERFATALRGLPEQQRRHSLLPVLEVYRHPGRARAGSALPAAGFRDAVRAAG
LPGAPDIPHLDPALITKYVTDLRELGLL
>Mycobacterium_kyorinense
MSIEINEERLERRIADLTATDQQFAAARPSQAVAAAVEQPGLTLPQVIQTVLEGYADRPALGQRAVEFVADPATGRTSAQLLPRFDTITYRELGERVGALASAWADGVVSP
GDRVAVLGFTSVDYTTIDMALAQLGAVSVPLQTSAAIAQLQPIVAETEPTVLAASVDYLSDAVELIQSGRQPARLVVFDFHPEVDDHREALDAARARLANSPVAVETLADV
LDRGKSLPPVPAVDTGNDALALLIYTSGSTGAPKGAMYPQRNVAKMWRRSARNWFGPSAASITLNFMPMSHVMGRGILYGTLGNGGTAYFGAKSDLSTLLEDLALVRPTEL
NFVPRIWETLYQEYQRELDRGRGEAEVLADMRQNLLGGRFIFAMTGSAPISPELNAWVESLLEMHLMDGYGSTEAGMVLFDGQVQRPPVIDYKLADVPDLGYFATDKPYPR
GELLLKTENMFPGYYKRPEITADVLDADGYYHTGDVVAEVAPDRLVYVDRRNNVLKLAQGEFVTLAKLEAVFGTSPLVRQIYIYGNSAHPYLLAVVVPTDPNASKSAIAES
LQTVAKEAGLQSYEVPRDFIIETTPFTLENGLLTGIRKLAWPKLKQHYGERLEQLYAELEESQANELRTLRQSGADKPVLETVSRAAAALLGASTSDVSPDAHFTDLGGDS
LSALTFGNLLREIFDVDVPVGVIVSPASDLQSIAGYIEGERQGSKRPTFASVHGRDAVEVHAKDLTLDKFLDADTLAAAPNLPRANHEVRTVLLTGATGFLGRYLALEWLE
RMDLVDGKVIALVRAKDDAAARERLDKTFDSGEPKLLAHYQELAADHLEVIAGDKGEANLGLSQDVWQRLADTVDLIVDPAALVNHVLPYSELFGPNALGTAELIRIALTT

KIKPYTYVSTIGVGDQIAPGKFTEDADVRVMSPTRAINDGYANGYGNSKWAGEVLLREANDLAGLPVAVFRCDMILADTTYAGQLNLPDMFTRMMLSLVATGIAPKSFYEL
DADGNRQRAHYDGLPVEFIAEAISTLGAQNVETFQTYHVMNPYDDGIGMDEYVDWLIEAGYPIQRIDDYREWVQRFETSLRALPEKQRQASLLPLLHNYQKPEKPIRGSIA
PTDRFRAAVQEAKIGPDKDIPHVTAPVIVKYITDLQLLGLL
>Mycobacterium_haemophilum
MATRDERLARRVDDLTANDPQFAAARPDPAVAAALEQPELRLPQVVAAVLEGYADRPALGQRAVEFVKDARTGRTGLQLLPRFDTITYRELGDRVGALARALTTDSVQAGD
RVCVLGFTSIDFTTIDMALGQISAVAVPLHVSATTSALRPIVTETEPSVIAASVNQLADAVELILSGPAPAKLIVFDYHPEVDDQREAVATARARLADTAVVIDTLADMLD
RGKALPAAPVTVPDSDHQLAVLIYTSGSTGAPKGAMYPQSNVGKMWRRSSRSWFGPTAASITLNFMPMSHIAGRGILYGTLGNGGTAYFAARSDLSTLLEDLKLVRPTELN
FVPRIWETLFGEFQRQVDRRLTDSATREAVESEVMDEQRQYLLGGRYIFAMTGSAPTSPELKHWVESLLEMHLLDGYGSTEAGMVLFDGEIQRPPVIDYKLVDVAELGYFR
TDRPYPRGELLLKTQNMFPGYYKRPEVTAGVFDADGYYRTGDVVAEVGPDRLVYVDRRNNVLKLAQGEFVTVAKLEAVFGNSPLVRQIYVYGNSAHPYLLAVVVPTEDALA
SNDIEVLKPLIAGSLQDVAKEAGLQSYEVPRDFIVETTPFSLENGLLTDIRKLAWPKLKQHYGARLEQLYTDLTEGQANELRALRQCGADAPVLQTVSRAAAAMLSAATTD
LSPDAYFTDLGGDSLSALTFGNLLREIFDIDVPVGVIVSPANDLAAIADYIEGERQGSKRPSFASVHGRDAVEVHASDLTLDKFIDASTLATAPTLPQPSAEVRTVLLTGA
TGFLGRYLALEWLERMDLVGGKVIALVRAKSDEDARARLDKTFDSGDPELLAHYQELAADHLEVIAGDKGEADLGMDRQTWQRLADTVDLIVDPAALVNHVLPYSELFGPN
ALGTAELIRIALTSKQKPYIYVSTIGVGDQIAPAKFTEDADIRVISPTRNINDSYANGYGNSKWAGEVLLREAHDLCGLPVAVFRCDMILADTSYAGQLNVPDMFTRMMLS
LAATGIAPGSFYELDAEGNRQRAHYDGLPVEFIAEAISTLGAQRVAFRTYHVMNPHDDGIGMDDFVDWLNDAGCLIQRISDYGEWLRRFETSLRALPERQRHSSLLPLLHN
YQKPEKPLHGSLAPTDRFRTAVQDAKIGQDKDIPHISPAIIAKYVSDLRLLGLL
>Mycobacterium_mageritense
MSTETREERLARRITELSATDPQFAAAIPDEAVTAAVDEPGLPLPQIVQTVLQGYSDRPALGERAVEFVADPATGRTMARLLPRFDTITYGQLWDRVRATAAAWHEAGVTA
GDRVAILGFTSADYTALDTALGQLGAVSVPLQTSSSPAALAPIVVETQPRVIAASVDHLPDAVELALTAHAPAQLVVFDHHPEVDDHRDALVAARERLDAAGLVVPLVTLA
ELVEQGRELPQAPAPQVDESDPLALLIYTSGSTGAPKGAMYQQSAVAKFWRRNSKAWFGPTAASINLSFMPMSHVMGRGILYASLAAGGTAYFAARSDLSTLLEDLALARP
TELNFVPRVWEMIYGEFQSRVDRQAADGADRADVEAAVIADVRDHLLGGRYISAMTGSAPISAELKAWVEQSLDIHLLEGYGSTEAGMVLFDDQIQRPPVIDYKLVDVPDL
GYFGTDKPHPRGELLVKTDNMFPGYYKRPEVTAGVFDEDGFYKTGDIVAEVAPDHLKYVDRRNNVLKLAQGEFVTVAKLEAVFGNSPLVRQIYIYGNSARPYLLAVVVPTE
DALDRHEPSELKALIAESLQDVAKAADLQSYEIPRDFLVETTPFTLENGLLTGIRKLAWPKLKAHYGDRLEQLYAELADVQANELRALRANGADAPVLETVSRAAGALLGA
SAGDVQPDAHFTDLGGDSLSALTFGNLLQEIFDVEVPVGVIVSPANDLAAIAAHIESARHGSKRPTFASVHGRHAAEVSAADLTLDKFIDAETLAAAPSLPKAGSEVRTVL
LTGATGFLGRYLALEWLERMDLVDGKVITLVRAKSDADARARLDATFDSGDPKLLAHYRELAADHLEVLAGDKGEADLGLDRETWQRLADTVDLIVDPAALVNHVLPYGEL
FGPNALGTAELLRIALTTKIKPYTYVSTIGVGDQVSPGTFVESGDIRAISPIRRIDDSYANGYGNSKWAGEVLLREANDLCGLPVAVFRCDMILADTTYSGQLNLPDMFTR
MMFSLVASGIAPGSFYQLGADGKRQRAHYDGLPVEFIAEAISTLGAQSVDSFETYHVMNPYDDGIGMDEFVDWLVEAGYPIQRIADYREWVQRFESTLRALPDRQRQASLL
PLLHNYQQPAVPLNGAMAPTDVFRAAVQEAKIGPDKDIPHVTREVIVQYITNLEQLGLL
>Mycobacterium_sp._IS-3022
MTSDIRETRLARRISHLFASDPQFAAAQPNEHIAHAIEASDLTLRRVLHTIFDAYADRPALGQRAVEFVTDADTGRTSAQLLPRFDTITYRELSDRVDAVAAALTHNGVQP
GDRVAILGFTSIDYTTIDMALLRVGAVSVPLQTSAPVGQLRPVAAETEPVAIASSVDLLDDAVEVMLTGHLPQRLMVFDYHAELDDHRDTLTAARSRLAETDVVVETLGDT
LARGRALPTPTPFDFGDDDLALLIYTSGSTGTPKGAMYLAKMVAGSWRRSSMATWGAGEALPSITLNFMPMSHMMGRGILYATLGAGGIGYFVARSDLSTLLNDLALVRPT
QLNFVPRVWDMIFQQFQSEVDRRTATGVDRGTAETEVLTDLRQNLLGGRFVSAMTGSAPISAEMTAFVESLLDLHLTDGYGSTEAGAVFVDGQVARPPVIDYKLADVPDLG
YFRTDRPHPRGELLIKSETMFPGYYKRPETTAEVFDADGYYRTGDVVAELGPDRLAYLDRRNNVLKLSQGEFVTVSKLEAAFDTSPLVRQIFVYGNSARSYLLAVIVPTED
ALARHGDASLKSAITESLQDVARTAGLQSYEIPRDFIVETTPFTLENGLLTGIRKFARPKLKERYGEQLEQLYTQLADSQAGELRELRLHGSDRPVIETVSRAAAALLGAA
AADVQPDAHFTDLGGDSLSALTFGNLLTEIYDIDVPVGVIVSPANDLTALAEYVEAARRGSVRPTFASVHGRDTVEAHAGDLTLDKFVDAETLAAAPSLPGPASEVRTVLL
TGATGFLGRYLALEWLERMEMVDGKVICLVRAKDDASARARLDDTFDSGDPELLRHYRELAAEHLEVVAGDKGEADLGLDRHTWQRLADTVDLIVDPAALVNHVLPYSQLF
GPNVVGIAELIRLALTTKIKPFVYVSTIGVGAGITPGRFTEDGDIRVISATRKVDDSYANGYSNSKWAGEVLLREAHDLSGLPVSVFRCDMILADTTYAGQLNLPDMFTRL
ILSLVATGIAPFSFYEVDGDGNRQRAHYDGLPVEFIAEAISTLGEQVGHAPAAAFETYHVMNPYDDGLGLDEFVDWLIDAGYRIERVGDYATWLHRFDTAVRALPERQRQA
SLLPLLHNYQRPETPIRGSIAPTERFRTAVQDAKIGPDKDIPHVTRGVIVKYVTDLELLGLV
>Segniliparus_rotundus
MGSGADRAKLFFQKIEELTAADPQFAAAVPDQEVVAAVSDPTLSFTRYLDTLMRGYADRPALAHRVGDGYATISYGELWSRVGAIAAAWSADGLEPGDFVATIGFTSPDYT
ALDLAATRSGLVSVPLQAGASVAQLSAILEETAPKVFAASAESLEGAVDCVLRTPSVQRLVIFDLRDDSPEHRAALAAAKAKLAQPQNPEQARGPVAVETLDELVARGAAL
PEPPVFEPAEGEDPLALLIYTSGSTGTPKGAMYSQRLVSRFWPRTPVVAQLPSISLHYMPLSHSYGRAVLCGTLAAGGTAHFTAHSDLSTLFEDIALARPTFLALVPRVCE
MLLHESRRARDLAELRERVLGERLLVAVCGSAPLAPETRAFMEELLGFPLLDGYGSTEALSLMRDGVIQRPPVIDYKLVDVPELGYFTTDKPHPRGELLIRSESLVSGYYK
RPELTAEMFDEQGYYKTGDVMAEIAPDRLVYVDRSKNVLKLSQGEFVAVAKLEAAFGASPYVKQIFVYGNSERSFLLAVVVPNAELVGRLDTVQALAEVKPLIADSLAAIA
KESGLQSYEVPRDFIVETEPFTTGNGLLSEVGKLLRPKLKERYGERLEALYDQIAQGQADELRALREQAGERPVIDTVRKAAAAVVGSSGADFRPDANFADLGGDSLSALG
FANLLQDVFGVETPVRIIIGPTASLAGIAEHIERALGGRPGEAAPNSASVHGAGAEVIRASDLTLDKFLDAQALEAAQSLPRPTGSHRTVLLTGANGWLGRFLALEQLQRL
EATGGKLICLVRGKDAASARARVEEALGTDPALAARFAELAADRLEVVPGDVGEPKFGLDDRTWDRLAGEVDAVVHSGALVNHVLPYHQLFGSNVVGVAEIIRFAVASKLK
PVAYLSTVAVAAGADPAAFDEDGDIREVVPQRPVDDSYANGYGNSKWAGEVLLREAHERTGLPVRVFRSDMILAHRQHTGQLNATDQFTRLILSLLATGLAPKSFYQLDPQ
GRRQRAHYDGIPVDFTAEAIVALAAEGNNGHRSYNVFNPHHDGVGLDEFVDWLIEAGHPITRIEDHATWFARFTTALRALPEKQRQLSLLPLAQVYSFPHPAVDGSPFRNA
VFRADVQRARIGKDHDIPHLTRELILKYAADLAALGLL
>Segniliparus_rugosus
MGDGEERAKRFFQRIGELSATDPQFAAAAPDPAVVEAVSDPSLSFTRYLDTLMRGYAERPALAHRVGAGYETISYGELWARVGAIAAAWQADGLAPGDFVATVGFTSPDYV
AVDLAAARSGLVSVPLQAGASLAQLVGILEETEPKVLAASASSLEGAVACALAAPSVQRLVVFDLRGPDASESAADERRGALADAEEQLARAGRAVVVETLADLAARGEAL
PEAPLFEPAEGEDPLALLIYTSGSTGAPKGAMYSQRLVSQLWGRTPVVPGMPNISLHYMPLSHSYGRAVLAGALSAGGTAHFTANSDLSTLFEDIALARPTFLALVPRVCE
MLFQESQRGQDVAELRERVLGGRLLVAVCGSAPLSPEMRAFMEEVLGFPLLDGYGSTEALGVMRNGIIQRPPVIDYKLVDVPELGYRTTDKPYPRGELCIRSTSLISGYYK
RPEITAEVFDAQGYYKTGDVMAEIAPDHLVYVDRSKNVLKLSQGEFVAVAKLEAAYGTSPYVKQIFVYGNSERSFLLAVVVPNAEVLGARDQEEAKPLIAASLQKIAKEAG
LQSYEVPRDFLIETEPFTTQNGLLSEVGKLLRPKLKARYGEALEARYDEIAHGQADELRALRDGAGQRPVVETVVRAAVAISGSEGAEVGPEANFADLGGDSLSALSLANL
LHDVFEVEVPVRIIIGPTASLAGIAKHIEAERAGASAPTAASVHGAGATRIRASELTLEKFLPEDLLAAAKGLPAADQVRTVLLTGANGWLGRFLALEQLERLARSGQDGG
KLICLVRGKDAAAARRRIEETLGTDPALAARFAELAEGRLEVVPGDVGEPKFGLDDAAWDRLAEEVDVIVHPAALVNHVLPYHQLFGPNVVGTAEIIRLAITAKRKPVTYL
STVAVAAGVEPSSFEEDGDIRAVVPERPLGDGYANGYGNSKWAGEVLLREAHELVGLPVAVFRSDMILAHTRYTGQLNVPDQFTRLVLSLLATGIAPKSFYQQGAAGERQR
AHYDGIPVDFTAEAITTLGAEPSWFDGGAGFRSFDVFNPHHDGVGLDEFVDWLIEAGHPISRIDDHKEWFARFETAVRGLPEAQRQHSLLPLLRAYSFPHPPVDGSVYPTG
KFQGAVKAAQVGSDHDVPHLGKALIVKYADDLKALGLL
>Nocardia_brasiliensis
MFAEDEQVKAAVPDQEVVEAIRAPGLRLAQIMATVMERYADRPAVGQRASEPVTESGRTTFRLLPEFETLTYRELWARVRAVAAAWHGDAERPLRAGDFVALLGFAGIDYG
TLDLANIHLGLVTVPLQSGATAPQLAAILAETTPRVLAATPDHLDIAVELLTGGASPERLVVFDYRPADDDHRAALESARRRLSDAGSAVVVETLDAVRARGSELPAAPLF
VPAADEDPLALLIYTSGSTGTPKGAMYTERLNRTTWLSGAKGVGLTLGYMPMSHIAGRASFAGVLARGGTVYFTARSDMSTLFEDLALVRPTEMFFVPRVCDMIFQRYQAE
LSRRAPAAAASPELEQELKTELRLSAVGDRLLGAIAGSAPLSAEMREFMESLLDLELHDGYGSTEAGIGVLQDNIVQRPPVIDYKLVDVPELGYFRTDQPHPRGELLLKTE
GMIPGYFRRPEVTAEIFDEDGFYRTGDIVAELEPDRLIYLDRRNNVLKLAQGEFVTVAHLEAVFATSPLIRQIYIYGNSERSFLLAVIVPTADALADGVTDALNTALTESL
RQLAKEAGLQSYELPREFLVETEPFTVENGLLSGIAKLLRPKLKEHYGERLEQLYRDIEANRNDELIELRRTAAELPVLETVTRAARSMLGLAASELRPDAHFTDLGGDSL
SALSFSTLLQDMLEVEVPVGVIVSPANSLADLAKYIEAERHSGVRRPSLISVHGPGTEIRAADLTLDKFIDERTLAAAKAVPAAPAQAQTVLLTGANGYLGRFLCLEWLQR
LDQTGGTLVCIVRGTDAAAARKRLDAVFDSGDPELLDHYRKLAAEHLEVLAGDIGDPNLGLDEATWQRLAATVDLIVHPAALVNHVLPYSQLFGPNVVGTAEIIRLAITER
RKPVTYLSTVAVAAQVDPAGFDEERDIREMSAVRSIDAGYANGYGNSKWAGEVLLREAHDLCGLPVAVFRSDMILAHSKYVGQLNVPDVFTRLILSLALTGIAPYSFYGTD
SAGQRRRAHYDGLPADFVAEAITTLGARAESGFHTYDVWNPYDDGISLDEFVDWLGDFGVPIQRIDDYDEWFRRFETAIRALPEKQRDASLLPLLDAHRRPLRAVRGSLLP
AKNFQAAVQSARIGPDQDIPHLSPQLIDKYVTDLRHLGLL
>Mycobacterium_smegmatis3

MSTVSREERLARRISDLYATDQQFADARPSEAVAHAIESPALRLPQIIQTVIDGYAERPALGQRAVEFVTDPTTGRTSAALLPRFDTITYRELSERVDAVATALTQNPVRP
GDRVAILGFTSIDYTTVDMALLRAGAVSVPLQTSAPVAQLRPIAAETEPVAILSSVDFLDDAVELMLTGHLPERLVVFDYHAEVDDHREALASATARLAETPVVVETLAEV
LARGNALPAHPAFDAGDDNLALLIYTSGSTGTPKGAMYLAKAVANSWRRSSMAMWGNEGATPSITLNFMPMSHMMGRGILYATLGAGGTAYFVARSDLSTFFDDLSLVRPT
QLSFVPRIWDMVFAEYQSEVDRRSADGGDRWAVEADVLADLRQNLLGGRFISAMTGSAPISSEMRTFVESLLDIHLTDGYGSTEAGAVFVDGQVQRPPVIDYKLVDVPDLG
YFSTDRPYPRGELLVKSETMFPGYYKRPEITADVFDADGYYRTGDVVAELGPDQLQYLDRRNNVLKLSQGEFVTVAKLEAVFVDSPLIRQIFVYGNSARSYLLAVIVPTEE
ALARHDAAELKQLITESLQDVAKATGLQSYEIPRDFIIETTPFTLENGLLTGIRKLARPKLKEFYGERLEQLYTDLADSQANELRELRQHGADRPVLETISRAAGALLGAA
ASELQPDAHFTDLGGDSLSALTFANLLHEIFEIDVPVGVIVSPANDLAALAAYIEAERQPGSKRPTFASVHGRDATEVYANDLTLDKFIDAKTLAAASSLPGPSSEVRTVL
LTGATGFLGRYLALEWLERMDMVDGKVIALVRAKDDDAARERLDKTFDSGDPELLRHYHELAADHLEVIAGDKGEANLGLDQQTWQRLADTVDLIVDPAALVNHVLPYSQL
FGPNALGTAELIRIALTTKQKPFVYVSTIGVGAGIEPGQFTEDGDVRVYSATRKVDDTYANGYGNSKWAGEVLLREAHDLGLPVSVFRCDMILADTTYAGQLNLPDMFTRL
MLSLVATGIAPDSFYEVDANGNRQRAHYDGLPVEFIAEAISTLGAQVVDGFETYHVMNPYDDGIGLDEYVDWLIEAGFPVERVGDYTTWLQRFDTAVRALPERQRQASLLP
LLHNYQRPETPIRGSIAPTDRFRAAVQDAKIGPDKDIPHVTRDVIVKYITDLQLLGLL
>Mycobacterium_brisbanense
MTDDARQQRLIQRIQQLHATDPQFRAAAPDPDVSARIQDPDLRLWQAIDACLTGYADRPALGLRSREVIRDPATGRTTTSLLAGFDTITYGRLRDNVVALANTWHGSGFEP
GDFVAVLGFTSIGYTVIQLACARLGAVFVPLQTSSSARQLAPIIAETTPRIFAASVESLDTAAELIIDAPSVQRLVVFDYSEADDDQRERFEAATARLTAAGRTVEVVSLV
ADMDAGRSMAETPPFIPAPDENPLATLVYTSGSTGAPKGAMYTTDLMTRLWQRPRSPALDIGIEIPAIHLQYMPLSHVYGMAWLISTLTSGGIGYFAAKSDMSTLFDDITL
VRPTALNLVPRVCDMFFRRYRKELDQRTAAEPTAADLEETVKAELREDFLGGRVVSAMCGSAPLSKDMHAFVESLLDIAVSDGYGSTENGGGIMRNGVVQRPPVTEYKLVD
VPELGYSTTDKPHPRGELYIKTTTLIPGYYKHPELSADIFDADGFYKTGDVMAEIGPDHLVYLDRRNNVIKLSQGEFVAVSNLEATYSTSPYIRQIFIYGSSDQPFLLAVI
VPNADGVGAGDARSLIADSLQQIAAENHLNPYEIPRDFLLESEPFTRDNGLLSGVGKLLRPALKRHYQDRLDAMYAEIAAGQDNQLDALRTASRHQPTIETVRGAAAATLG
LDAGGSDLPADAKFIELGGDSLSAFSFATLLEGIFNIDVPVQTIVSPTATLTTVAEYVDGERQSASTRPTFASVHGRDAEVAKATDLTLDKFIDAATLAAAPTLPAPSATV
NTVLMTGANGYLGRFLCLDWLERLAPTGGRLICLARGADPIAARQRIEEAIDSGDETLSKRFRELADKHLEVLVGDVGAANLGVDTVTWNRLADSVDLIVHSAALVNHVLP
YSQLFGPNAVGTAEIIKLAITNRLKPVNYISTVAVTALPDGTYVGEDVDVRTASPARSLGAAYAGGYATSKWAGEVLLREANDLCGLPVTVFRSDMILAHSTYSGQVNVTD
MFTRLILSIVATGRAPKSFYTLDAAGNRQRAHYDGLPADFTSAAITALGAASTTGYHTYNVLNTHDDGISLDEFVDWLIAQGLDIERIDDYEEWRERFEEAMKALPEDQRK
NSVLPLMSAYAHPAPPTGGVAMPAEKFRAAVQSAGIGAGRDVPHLSEELIAKYIADLRQLGLLDAG
>Nocardia_gamkensis
MRRITRLIAENDDIRNATPRAEIAEKLREPGAGLAQMVETVMVGYADRPALGTRATELRTGESGRTTLSLLPEFDTITYGELWERVRAVAAAWHGDTREPLRAGDFVGILG
FTSSDYTTLDLACIHLGLVAVPLQSNAPVAQLSAIVAETAPRVLAATPELLDAAVTCALSQAGPQRLIVFDYHPGDDDQRSAFEAARARLAEAGSSVLVEPLDEVLARGRS
LPPAPLFSAEQGVDPLALLIYTSGSTGTPKGAMYSQRLVARGWVNRRDVAAINLNYMPMSHVAGRGSLTGVLARGGTAYFTAESDMSTLFDDIALARPTELFFVPRVCDMI
FQRFRGEVDRRDGPGVDRAALEAQVKTALREHFLGGRMVMAICGSAPVSAEMKSFVESVLELELHDGYGSTEAGGVVVIDERVQRPPVLDYKLADVPELGYFRTDKPHPRG
ELLLKSTTLISGYYKRPELTAEIFDEEGFYKTGDIVAELAPDRLVYVDRRNNVLKLSQGEFVAVSHLEAVYATSPLIQQIFVYGSSERAYLLAVIVPTEDVLGWGDLEKMK
TALSESLRQLAKDAELQSYEIPRDFLIEPEPFTTANGLLSGIGKLLRPKLRERYEPRLQQLYADLDAGQANELLDLRRSAVDLPVLQTVGRAAKAVLGCADADLRPDAHFT
DLGGDSLSALSLSNLLHEVFDVEVPVSVVISPANNLREVADYIAAVRVSGGRGATYTTVHGAGPQVRAADLTLDKFLDARTLAAAPSLPRPTETARTVLLTGANGYLGRFL
CLEWLRRMHDTAGTLVCLVRGSDAAAARQRLDSAFDTGDPTLVREFRELAAAHLEVIPGDIGEPNLGLDDRTWDRLADRVDLIVHPAALVNHVLPYRQLFGPNVVGTAELI
RLALTTRLKPFTYLSTVAVAAQIDPAVFAEDGDIREISPVRAVDDSYANGYGNSKWAGEVLLREANDLCGLPVAVFRSDMILADRRYAGQLNVPDIFTRLLLSLIVTGIAP
FSFYRTDADGNRSRAHYDGLPADFTADAITALGATAGTGFQSFDVLNPHDDGRSLDEFVDWLIAAGHRIERIADYREWFTRFETALRALPEWQRQASVLPLLHAYRRPAPA
VRGSALPAERFRSAVRAAKIGAEHDIPQISRELIEKYATDLQLRGLL
>Nocardia_mikamii
MPDDVQKAERKRRLAAAMADDEVRAAWPDADVSAALARPGLRLAELIDTVVTAYADRPAVGQRAGEIVVDAEGRRVRRLLPRFETLSYRQLWSRAGALASAWQAAGVRAGD
FVCTVGFVSSDYVTVDLAGVRLGAVAVPLQATAAVAQWHSIIAETEARVLACSAELLDAAVEAALTSATIGQLVVFDSADDDAEREAVAAARTRLAESGRDITIDSLHDLV
ERGLALPAVPLASAPGDDPLSLLVYTSGSTGTPKGAMYTDRLAAAMWLYTAKAPVPAITLNYLPLSHVAGRLQLGGTLARGGTAYFTARSDMSTLFEDLELSRPTELVFVP
RVCEMLLQHHQGEVEARIAAGGERAVVEDEVKTELRERLLGGRFLGAMCASAPLAPEMRAFMESVLGIGLHDGYGSTEAGGSVIVDNKVRRPPVLDYKLVDVPELGYFGTD
QPHPRGELLLKTTTMFPGYFKRPEITAEMFDEDGFYRTGDVVAELGPDHLVYVDRRNNVLKLSQGEFVTIAKLESVFSTSALIRQIFVYGSSERAYLLAVIVPSEEALTLP
DPRAALAESLQELAKEAGLDSYEIPRDFLLETEPFTQDDGLLSGIGKLLRPKLKQRYGKRLEQLYAELSQEQADELAALRHGAADRPVLETVGRAARALLGCASTDIRPEA
HFTDLGGDSLSALSLSNLLTDLFGVQVPVGTIVHPANTLRRLAEYIDGERNEGGRRPTLASVHGPGTRVRAADLTLDAFIDHETLTAAQSLPPASTPPRTVLLTGANGYLG
RFLCLEWLDRLDTCDGTLICVIRGRDAEAARARLDEVFDSGDPVLLQRYRELAQRRLTVLPGDIAEPNFGLDEDRWRELAETVDLVVHPAALVNHVLPYDQLFGPNVVGTA
EVVRLAITARRKPVTYLSTVAVAAQVDHFTEDGDIREVSPERVIDGGYANGYGNSKWAGEVLLREAHDLCGLPVAVFRSDMILAHSDFAGQFNLPDMFTRLLLSVLATGLA
PESFYRPAPGGGRARAHYDGLPADFTAAAITALGPGADSGFETFDVLNPHDDGIGLDTFVDWLVDDGHRITRIADYADWLARFETALRALPERQRRHSVLPLLHAFRRPAP
AVAGSALPAERFRAAVRATRTGPDGGIPHLSRELIGKYVRDLTAAGLL
>Streptomyces_aureofaciens
MYATDPQFRDAAPLDSVTEAIRRPGLPLADLVATVMEGYADRPALGERATEPVTDPDTGRTTLRLLERFDTITYGELWERVGAVASEWRHHPGHAVDRGDFVALLGPTSAE
YAMVDLACLRCGAVSVPLQAGASAEHLAPIIAQTGPRLLAVDMAHLDVALQLAADAPSLDRIVVLGHRSEITAHQEGLDSARDRLAAQGRGVTVDTLASVIERGRALPPLP
RCPEGATPDALSSLIYTSGSTGTPKGAMYTERLVRQFWVDFVPGQGVRPSIVLNYMPLSHMMGRGVLFGTLAKGGIAYFVASSDLSTLFEDLSLTRPTEFIMVPRISDMLF
QRYQAELARRSDTGAEAPGTTADRAEHVQEDVQAELQAELREELREKTLGGRLLWALSASAPLSAEMTAFVEKCLHVRLLNGYGSTEAGIVSLDGRVVRPPVTDHKLADVP
ELGYFRTDSPHPRGELLIKSDRLFSGYFQRPDATAQVFDEDGFYRTGDIMARTGPDTLVYVDRRSNVLKLSQGEFVATSRLEALFIGSPFVRQVFVYGNSTRAYLLAVIVP
TQDALDRSGEDTQRLRSILRESLQRLAAEAGLNAYEIPRDFLIETEPFSQQNGLLSGVRKLLRPALTKRYGERLEALYTELAERGTDELQALRQAGPSQPVPETVLRAARA
LLGHRQGDVKPDTHFLELGGDSLSALSFSQLLKEIFHVDVPVDVLINPVNTLRQVADHIENALAGHQRPTADSVHGPGAKRLLASDLKLGAFLDTGTLTKTGRPAGPLPEA
RTVLLTGANGYLGRFLCLEWLERVAERGGTLVCVVRGSTDEAARARLDAAFDSGDAELLRHYREVAAEHLEVIAGDIGEADLGLDKETWQRLADTVDLIVHPAALVNHVLP
YDQQFGPNVLGTAELIRLALTSRVKQFTYLSTVAVVFGAEAAADETADIRTACAVRDLGGGYADGYAASKWAGEVLLREAHETYGLPVAVFRSNMILAHRRYRGQLNIPDV
FTRLLLSLLATGIAPGSFYAGGAGAHTGSGHYDGLPVDFTARAVAALGDGTREGYRTFNVVNPHEDGISLDTFVDWLTAAGHPLTRIHDYDAWLDRFETALRGPPDRQRQH
SLLPLLHAFTKPEEPLPGSALPAQRFRAAVRAAALDGKADIPHLSQDLITKYVADLRAQHLL
>Streptomyces_roseoverticillatus
MASRVEALHAHDPGFARAAPSPSVAEAVHRAGGSLIGTISAAMSGYADRPALGRRAERIVEDPATGRRTTELLPHFETVTYAELWQEVGSLAAAWADASGGLRAGDFIGVL
GFTSTDYATVDLACMYLGAVSVPLPTGWSAARLAPILAETEPRILAADLGSIDAAVEAVLATDSVERLVVFDYDPRVDDHREALQTAVGKLSGRAPVVPLQEELGRGRRLP
ALEPRDDGDPDRLAGLIYTSGSTGAPKGAMYTASMITRMWQNSRTGMPNAAPGSDRPVPTIVLHYMPMSHVNGRARLISGLASGGIGFFTARSDMSTLFDDIGLARPTVLS
LVPRICDMVHQRYLLEADRLSRTGTPRITRAEAEEVALTRVRDDMLGGRIVSALCGSAPLSSQMHTFMAEVLGTKVVDCYGSTETTRPVVVDQQVRRPPVIDYRLVDVPEL
GYFATDKPHPRGELRLKSVGLVQGYYKQPEVTARAFDEVGYYKTGDVVAEIAPDRLVYVDRINNVVKLSQGEFVAVSRLEALYSTSPYISRVYVYGSSEQAFLLAVVVADR
EQLDQERLGREQLDGEKSQSDGADGADGVDGAEGADGAEGADDDAATRAKILDSMRELARDAGLNPYEVPYDVVLEPHPFTIENGLLSGVGKLLRPALKDRYGAQLEQLYA
DIAGGRAGRIAALRAAGRSAEPLEAVLAAVQITLGYPASMVVPEAAFTDLGGDSLSALTFSTVLEQIFDTEVPVQAVLSPTSTLAGIADHLAAAPATAAAPRPTFASVHGR
GATEVRAGDLTLDRFVDEQVLVRAPKAPAPTDPPTSGNVLLTGATGYLGRFLAVEWLERVAASGGRLTVLARGADDAAAGQRVLECLRSASGDRTDWFDAVAARHLEVLAA
DVSAPKLGLGRTTWERLAAETDRIVHSAALVNHVLPYSRLFEPNVAATAELIELALTHRMKKFAYVSTIAAAMMPGGTFLDETADVRTASPARQLNDSDANGYATSKWAGE
VLLRDAHDRTGLPVAVFRPDMILAHSRLTGQLNLPDRFTRLLLSVVATGMAPRSFHRLDADGNRRRAHYSGLPVDFTADAIASLSARSQDGYVTYHSVNANDDDVSLDEIV
DWLIRAGHPITRIDDYEQWRVRFEAALRGLPEHQRRLSMLPLMRAYARPTEPRTSSMVPAGRFTTAVAESGAGGGVIPSLSADFIDKCVADLRTLGIL
>Streptomyces_griseofuscus
MHPSRIPAGELDARTARRGAHLYATDTQFRDTAPLDTVTAAVRRPGLPLAALVATVMEAYADRPALGERATEPFTDPETGRTTLRLLNRFDTLTYGELWERAGAVAAEWRH
QRHHPEHAVRPGDFVALLGRPGTEYTMVELACVRSGAVSVPLPAGASAEQLAPLVEQTGPRLLAVDTDQLEVALRIAANTPSPPRIVVLGHRPEVTAHQADLAAARDRLAA
HGASLDTLASVIDRGRTLPPLPRVPETSAADALSTLIYTSGSTGTPKGAMYTERVVRQFWVDFVPGQAARPSIVLNYLPLSHMVGRGVLFGTLAKGGLACFAASGDLSTLF
EDLSLVRPTEFIMVPRIADLLLRHYRDELSRRGGTGTDLAEIEARVKEELREEVMGGRLLWAVSASAPLSAETTAFVESCLQVRLLDGYGSTEAGIVLLDGRVLRPPVTDH

KLADVPELGYFGTDSPYPRGELLVRSERLVPGYFRRPDATSEVFDEHGFYRTGDIMARVGPDELRYVDRRANVLKLSQGEFVAVSRLEALFGGSPAVRQIFLYGNSARAYL
LAVVVPTRDALDRADGDTRRLRTALRESLRSLAAEAGLNSYEVPREFLVETEPFSQENGLLSGVRKPLRPALTKRYGERLEALYAELSDREATELEALRRVGADEPVLDTV
LRAVRAHLGQEDAAVEPGTRFLELGGDSLTALSFSRTLKEIFQVDVPVDVVISPVNTLRQVAEHIERALAPDHRRPTADRVHGPDATRLDAADLRLDAFLDARSAARRPAG
PPPEARTVLLTGANGYLGRFLCLEWLERLAERDGTLVCLVRGSSADEARARLDAAFDSGDPELLKRYHEVADRHLHVIPGDIGEPDLGLDGETWRRLADTVDLIVHPAAQV
NHLLPYGQLFGPNVLGTAELIRLAITSRIKRFTYLSTVAVVFGDEAAADESADIRTACPTRDLKGEYADGYAAAKWAGEVLLREAHDAFGLPVAVFRSNLILAHPRYAGQL
NVSDVFTRLLLSLLATGIAPGSFYAPGTGAGGGHYDALPVDFTARAVTALGDDARAGFRTYNVVNPHEDGISLDTFVDWLAAAGHPLTRVPDHTEWFGRFETALRALPDRL
RQHSLLPLLHAFAAPEPPLSGSALPAERFRSAVRAASLGGDNDIPHLSPELITKYVTDLRALRLI
>Tsukamurella_pseudospumae_hyp
MLLMSVEIVESTEPNTAAGAASGAGGSPGHLADVITSAFTRFADRPAFATRDGGPDGPYRGTTYGQVWHRVTALTAAWREELQPGDFVAVLGFTSPDFVVVDLATTLLGAP
NVPLQAGAPAARIAAILDETAPKILAVSAAQLPLARAAVAESGTDPRLVVFDGAAPGATTLDDEISRGAALPVPEPYRAAEPDGDRLVTLIYTSGSTGTPKGAMYTEHLVV
HAWLKVESIIDDDIPTGVLLHFLPMSHMYGRNWLIAGLAAGGVGYFASAADMSSLFDDLAEARPTAIALVPRVCELVHQRFLALEAETDAESARTYLRDEVLGGNIAAAVC
GSAALSADLQAFMEQLLGVSIQIGYGSTEAGGVIVDGVVSRPPVIDYKLIDVPELGYLTTDLPHPRGELLVKSSQLIPGYYRSDKQVLDEDGYYRTGDVMAELGPDRLEYV
DRRSNVIKLAQGEFVPIAQLEATYSANPAVHQIFLHGTSERSYLLAVVVPAPGPADETDEQARGRVLDALATVARENELAGYELPRDVIIEREPFSAENGLRSGIGKLVRP
ALTTRYGDRLADLYAAAEERRRDGLRGLDADGPVADTVVRAAALTLGVLPEELDGGTRFVDLGGDSLSALSLATTLEGLYDLPVPVQTIVGPTATLAGVVAHIEAARAGAV
QAPTAASIHGTGASIARAADLRLDRFIDADLLAAAPSKPGPHGEPKTVLLTGATGYLGRFLLLEWLRRVALSDGTVVALVRGTDADDARRRVFDAIGTADAALTEEFARLA
AGHLEVVPGDFGSPSLGLDDGAWERLTHTVDHVVHCGALVNHVLPYDQLFGPNVVATAEIVRLAVTDRRKSVDYVSTVAVVPQDDGRLLVEDDDVRVLGAERRIGAEAYAN
GYAVSKWAGEVLLREASDLAGLPVRVFRSDMILAHSRFRGQYNPVDQFTRLLLSIAETGLAPQSFYEPDGSGRSPHYDGLPVDFTAESIVTLGSAAREGFRTFHVLNVNDD
AVSLDSFVDWIAQERTVERIADYDEWLERFERALRALPDADRQRSVLPLLHSFARRSPAGAGGALTADRFREAVHAENVGPGDIPHLDRALVDRYLRGFEDAGWLSSEGSV
>Tsukamurella_carboxydivorans_hyp
MSIETVWGDDPAITGAAAAGTADGRAERLPQVIARIFARYADRPAFATRDGGPRAPYATVTYGEVWRRVTALAAAWRSELEPGDFVAILGFTSADFVTVDLATTLLGAPNV
PLQAGAPAARIAAILDETRPKVFAVSADQVGLAEQALAESAATPRVVVFDGEHAGYEGIEADVLAGGVLPAPEFYAPEPGTDPLVTLIYTSGSTGTPKGAMYTEQLVSDAW
LKVDSIVDIDLPSESLLHFLPMSHMYGRNWLIAGLASGGTGYFAGASDMSTLFDDLAAARPTALGLVPRVCELIHQRFLAVEAETDTETARAELRDRVLGGRLQAAMCGSA
ALSAELQTFMEWLLGVEIQIGYGSTEAGGVLRDGAIVRPPVTEYKLVDVPELGYFVTDSPHPRGELLVKSTQLIPGYYNSDKRIRDEEGFYHTGDVMAELAPDRLEYVDRR
SNVIKLAQGEFVPIAQLEATYAAGPEVHQIFLYGTSERSYLLGVVVPAPGPDGESDAAARVRVLDGLAAIARENGLASYEVPRDVIIEREPFSQENGLRSGIGKLVRPALT
ARYGAELEALYAAAEERRRDGLRALDADGSVTETVVRAAALTLGVLPEELDEGTRFLDLGGDSLSALSLATTLEGIYDLPVPVQAIVGPTATLRGVIAHIEEARAGGVQAP
TSASIHGPDAEVARASDLRLDRFIDPALLAAAPSLPAPHGEPSTVLLTGGTGYLGRFLLLEWMRRVAAHGGTVVALVRGADADDARRRVFGAIGTADPALTEEFTALAEGH
LEVVVGDFGAPSLGLDTATWEALAERVDHVVHCGAMVNHVLPYDQLFGPNVVGTAEIARLALTVRRKSIDYVSTVAVVPQDDGRLLVEDDDVRVAGAERRIGADAYANGYA
VSKWAGEVLLREAADLADLPVRVFRSNMILAHSRFRGQYNPVDQFTRLLLSIAETGLAPASFSADPTGPRAHYDGLPVDFTAEAITRLGAAGRAGFRTFHVLNVAEGGAGL
DDFVDWIAEDRPIERIADYAEWLARFEAALRALPAEDRHRSVLPLLHSFARPAETGAGAALTADRFREAVREENVGPGDVPHLDRALIERYLDGFAATGWLA
>Tsukamurella_spongiae_hyp
MQSSDPVVTGSATGEAGAPAERLPQVIARVFERFADRPAFATRDGGPRAPYVTVSYGEIWQRVTALAAAWQSELAPGDFVAILGFTSADFVTVDLATTLLGAPNVPLQAGA
PAARIAAILDETRPKIFAVSADQAALAEQALAESSATPRVVVFDGEHAGYEGIEADVLAGRALPDPEFFAPEPDADPLVTLIYTSGSTGTPKGAMYTEKLVTDAWLKVDSI
VDYDLPSESLLHFLPMSHMYGRNWLIAGLASGGTGYFAGASDMSTLFDDLAAARPTAIGLVPRVCELVHQRFLTLEAETDTETARVELREHVLGGRLQAAMCGSAALSAEL
QTFMEWLLGIEIQIGYGSTEAGGVLRDGEIVRPPVTEYKLIDVPELGYFVTDSPHPRGELLVKSTQLIPGYYKSDKRIRDDEGFYRTGDVMAELAPDRLEYVDRRSNVIKL
AQGEFVPIAQLEATYAAGPDVHQIFLYGTSERSYLLAVVVPAPGPDGETDAQARTRVLDGLAAIARDQDLAGYELPRDVIIEREPFSQENGLRSGIGKLVRPALNARYGDE
LAALYAAAEDRRRAGLRDLDADGSVTETVVRAAALTLGALPEELDAATRFADLGGDSLSALSLATTLEGLYEVPVPVQTIVGPTATLGGIVEHIEAARAGALSAPTAASIH
GADAQVARASDLHLDRFVDPELLAAAPSIPAPHGEPSTVLVTGATGYLGRFLLLEWLRRVAPHEGAVVALVRGADADDARRRVLDAIGTSDDALTAEFAELAERHLEVVVG
DFGAPSLGLDAPTWDRLAERVDHVVHCGAMVNHVLPYDQLFGPNVVGTAEIARLAITVRRKSIDYVSTVAVVPQDDGRLLVEDDDVRVAGAERRIGADAYANGYAVSKWAG
EVLLHEASELAGLPVRVFRSDMILAHSRFRGQYNPVDQFTRLLLSIAETGLAPASFAADPTGPRPHYDGLPVDFTAEAIVTLGAAGREGFRTFHVLNVDSDGAGLDDFVDW
IAEDRPIERIADYGEWFARFEAALQALPAEDRQRSVLPLLHSFAHPTPNGGGVALTADRFREAVREANVGPGDIPGLDRALIERYLDGFTAAGWLA
>Streptomyces_sp._W007
MAEPLDATPASAHDPGQGLAEALASVDPGLALAEVMASVLESHGDRPALGERARDPETGCLLPRFDTISYRELWSRVRALAGRWHHDPEYPLGPGDRICTLGFTSTDYATL
DLACIHLGAVPVPLQSNAPLSQLAPIVEESGPTVLAASVDRLDTAVDVAIASSTIRRLVVFDHRPGTAGPREALAAARQRLAGAGSPVAVDTLAELIARGRSLPPPPLYTA
APGEDPLSLLIYTSGSTGAPKGAMYTQSLLGTAWYGFSYGAADTPAISVLYLPQSHLAGRYAVMGSLVKGGTGYFTAAGDLSTLFEDIALVRPTELTMVPRLCDMLLQHYR
GELDRRADEPGDIEAAVRKELREVFLGGRIAKAFVGTAPLSDELAAFVESVLGFHLYTGYGSTEAGGVLLDTVVQRPPVIDYKLVDVPELGYYATDLPYPRGELLLKSHTL
IPGYYRRPDLTATIFDADGYYRTGDVFAETGPDRLVYVDRTKDTLKLSQGEFVAVSRLETVLLGSPLVQHLYLYGNSERAHLLAVVVPTPAALAGCGGDTEALEPLLMESL
RNVARKAGRNSYEIPRGILVETEPFSPENGLFTESHKLLRPRLKERYGPALEQLYDQLADGQDRRLRELRRTGTDGPVEETVTRAAQALLGTPGADVRPGTRFTDLGGDSL
SALSFSELMEEVFHVDVPVGVILSPANDLAGVARYIGTARGPAGTRRPTFASVHGEHRTEVRARDLTLEKFLDAPALAAAPPRPRPDGDVRTVLLTGATGYLGRFLCLEWL
ERLAPSGGRLVCLVRGSDATVAARRLEEAFDSGDAALLRRYRKAAGKTLEVLAGDIGEPRLGLAEDTWRELAGTVDLIVHPAALVNHLLPYSELFGPNVVGTAEVIRLALT
TRLTPVNHVSTVAVCLGTPAETADENADIRATSPVRAIGQGYADGYATSKWAAEVLLREAHERFGLPVAVFRSDMVLAHSTYSGQVNVPDVLTRLLLSLVATGIAPGSFYR
AGARAHYDGLPVDFTAEAVVALGAPITEGHRTFNVLNPHDDGVSLDTFVDWLIEAGHPIRRIDDHRAWITRFTAALRDLPENHRKHSLLPLLGAYAEPGGGAPGALLPAGR
FHAAVREARVGPEQDIPRVSPDLIRKYVTDLRALGLLSSP
>Nocardia_higoensis
MATRTPLSAEAQEARLERRIADLYRFDEQCAAARPDPAVAARTEAPGLRPLELAQIVMEGYAHRPALGQRAIEFVTDRRTGRTSAKLLPTFETITYREVWDRAGAIAAALA
GDPVRSGDRVCVLGFTSVDYTLVDVALMRLAAVAVPLQASAVAAQLLPIVDETEPTVIAVSVDHLAIAVELALTGHTPQRLIVFDHHEQIDDEREALAAASMRLAEAGSPV
TIETLPAVIARGRALPPAPVPTRADADPLALLIYTSGSTGAPKGAMITERSVADNWRASTSERWGQRGAESVIVLAFMPMSHIMGRAVLYMALARGGTAYFVARSDLSTLL
EDLATVRPTHLTFVPRIWEMLFQRFQSEMQQRAAEGGDRAALERRVLADMRENLLGGRFLSATTGSAPMSAEMRAWAESLLELHLVDGYGATETGSIAVDGRICRPPVLDY
ELVDVPELGYFHTDRPYPRGELVVRSETLVPGYYKRADVTAEVFDSDGRYHTGDIFAEIGVDHLAFLDRRSFVLKLSQGEFVTVSKVEAVFSESPLVRQIFVYGNSTRSYL
LAVVVPTADAIEYTGGDIDALKQLVRESLQQVARSAGLQAYEIPRDVLIETQPFTPENGLLTGIRKLARPKLKEYYGPRLEQLYSELAVAEVEELRALRVGAGDRPTVETV
VRAAAAVLGAATRDVRPDAHFTDLGGDSLSALTFAQLLRDIFAVEVPVGFLIGPATDLHALAGYIDRQRRGADRPTFAAVHGADATEARATDLTLDEFIDGNTLAEAPRLP
GPSPEVRTVLLTGATGFLGRYLALEWLERLARTGGTLICLVRAHDDVDARARLDKTFDAGDPKLSDHYRELAAAHLEVIAGDKAEPELGLEHATWQRLADTVDVIVDPAAL
VNHLLPYSELFGPNVVGTAELIRLAVTTKQKPYTYVSTIGVNDQIDRSAFTEEADIRVISPRRTIDGSYANGYANSKWAGEVLLREANDLCGLPVTVFRCDMILADTGYAG
QLNVPDMFTRWVLSVEAVGTAPGSFYELDSDGHRQRAHYDGLPVGFIAEAIAALGARADTGFRTYHVMNPYDDGISLDTYVDWLIDAGHPIERVPDYELWLQRFETAVRAL
PDRQRRYSLLPMLQNYRKPQRAVRGSIAPTERFRAAVRHEEIGSTKDIPHITPEIIVKYTTDLQLLGLL
>Nocardia_sienata
MSTDTRESRLEDRIAELYRTDEQFAAARPDPAVTARADKPDARPVEAAQIVMQGYARRPAVGRRAVEFVTDPQTGRTSMKLLPRFDTLTYGEVWDRAGAVAAALSGDPVRP
GDRVCVLGFSSVDYTLVDLALVRLAAVAVPLQSGGPLNQLRSVVAETEPTVLATSIDHLDDAVDLVRTGHSPDRLIVFDHHPQDDDQRATFEAATARLSAAGSRVRVDDLT
EVIARGAELPPPQVPPPAEPDPLALLIYTSGSTGAPKGAMITEHRLAGQWRAASSEHWGQRATEPAIALGFMPMSHIMGRAILYMTLARGGTVYFAAKSDLSTLLEDLALV
RPTQLAFVPRIWEMLFQRFRGEVQRRAGGSADTATLEQEVLTDLRENLLGGRFFSATTGSAPISRELRTWVETLLDFHLVDGYGSTEAGTIAVDGRVRRPPVLDYELIDVP
ELGYFHTDRPHPRGELVIRSQALIPGYYERPEATAEVFDAEGWHHTGDIFAEVGVDELVYLERRSFVLKLSQGEFVTVSTVEAVLAESPLVRQVYVYGNSTRSYPLAVVVP
TEEALARAGGDIAVLRPMMTESLRQVARTAGLQSYEIPRDLLIETRPFTPENGLLTGIRKLARPALKQQYGPRLEQLYTDLAAAEADELRALHRGGDRPTVETVVRAAGAI
LGSPADELRPDAHFTDLGGDSLSALTFAQLLREIFDIEVPVGSIIGPATDLRALADYIDGQRRGGGRPTFASVHGANATEVHARDLTLDKFLDTETLAAAPALPGPSAPAR
TVLLTGATGFLGRYLVLEWLQRLNRCGGTLICPVRAQDDAAARARLDEKFETGDPVLSARYRELAADRLEVIAGDKSAPGLGTTRATWERLADTVDAIVDPAALVNHMLPY
RELFGPNVVGTAELIRLALTTRQKPYTYTSTIGVSEQIDRAAFTEDADIRVISPDRRIDGGYANGYSNSKWAGEVLLREAADLCGLPVTVFRCDMILADTGYAGQLNLPDM
FTRLVLSVEATGTAPGSFYELDAEGRRPRAHYDGLPVGFIAEAVATLGAGAGTGFRTYHVMNPYDDGISLDTYVDWLIDAGHPIERISGYDTWLQRMETAIRALPERQRRY
SLLPLLDNYRKPQRAIRGSIAPTERFRAAVRHAEIGADKDIPHVTPEMIVKYTTDLELLGLL

Supporting information

>Nocardia_fusca
MSTDTREARLEHRIAELYRTDPQFAAARPDPAVTARAEKPGARPFEVARTVMQGYAGRPAVGQRAVEFVTDPQTGRTAAKLLPRFDTLTYGEVWDRAGALAAALSGDPVRP
GDRIGVLGFSSVDYTLVDMAVVRLAAVAVPLQTSAPVDRLRSVVAETEPTVLATSIDHLDDAVDLVRTGHSPARLIVFDYHPQADDQRATFDAAAARLSAAGSPVRIDHLA
ELMARGAALPPPWVPPPTEPDPPALLIYTSGSTGEPKGAVITERLVADHWRAATSERWGQRATEPAIALGFMPMSHIMGRAILYMTLARGGTVYFAATSDLSTLLDDLALA
RPTQLAFVPRIWEMLFQRFRGEMQRRAAAGADPATLEQQVLTDLRQNLLGGRFLSATTGSAPISGELKTWVETLLDFHLVDGYGSTEAGSIAVDGRVRRPPVLDYELVDVP
ELGYFHTDRPYPRGELVIRSEALIPGYYKRPDATAEVFDADGRYHTGDIFAEVGADELVYLERRSFVLKLSQGEFVPVSTVEAALAESPLVRQVYVYGNSTRSYPLAVVVP
TEDAIAQTSGDLEALRPLLAESVRQVAKTAGLQSYEIPRDLIVETRPFTPENGLLTGIRKLARPALKQHYGPRLEQLYTELAAAEADELRALHRSGDRPTVETVVRAAGAL
LGSATGDLRPDAHFTDLGGDSLSALTFAQLLREIFDVEVPVGTIIGPATDLRALADYIDDRRRGGHRPTFASVHGAGATEIHARDLTLDKFLDTGTLADAPEPPAPNAPAR
TVLLTGATGFLGRYLVLEWLRRLNRSGGTLICPVRAQDDAAARARLDEVFETGDPALSARYRELATGHLEVIAGDKSAPRLGTSRATWEWLAETVDVIVDPAALVNHVLPY
RELFGPNVVGTAELLRLALTTRQKPYTYISTIGVGDQIDRAAFTEDADIRDISPDRAIDGGYANGYSNSKWAGEVLLREAADLCGLPVTVFRCDMILADTGYSGQLNLPDM
FTRLVLSVEATGTAPGSFYELDAEGRRPRAHYDGLPVGFIAEAVATLGARAGSGFRTYHVMNPYDDGISLDTYIDWLIDAGHPIERISGYDTWLQRMETAVRALPDRQRRY
SLLPLLDNYRKPQQAIRGSIAPTERFRAAVRHAGIGADKDIPHVTPEMIVKYTTDLELLGLL
>Nocardia_terpenica
MVDERVVERIAELEATDAQYAGARSDRAVAARVNDPELRLPEVVRAALDGYADRPALGARAVEVVAEPGTGRRVARLTRRLETVTYRELGERIAAVAGGLRRDPDGNSLVS
PGDRVVILGFTSIDYTVVDLALTQLGAVSVPLQASSSAQQLRPIVVETEPRVAAASIDHLLDAVELALTGFAPARLVVFDYLPEVDAQRELFDTAAERLAGSGVVLESLAA
VADRGAGGFTPEPPVTDDPLSLLIYTSGSTGAPKGAMYPESLAIRLWRGGFGLEDEDRAPGAWITLNFMPMSHVMGRATVFGTLGRGGIAYFGGSSDMSTFLEDLALIRPT
QLQFVPRVWELLFQEYQRALDRYTPDEALTVLRRDVLGGRALAGLTGSAPISAEVYEFADRLLGTHVIELYGSTEAGGIFVDGRVLRPPVRDYKLADVPDLGYYSTDRPYP
RGELLLLTETLFPGYFRRPEVTAEVIDEQGYYHTGDVVAEVAPDELRYLDRRNNVLKLSQGEFVTVSKLEAAFAASPVIEQIYVYGNSSRPYLLAVVVPTEKALAGDEAAV
KSRVTEGLRAIAAEVGLQSYEVPRDFLIETTPFTVENGLLTGIRKLARPKLKQHYGERLEQLYHDLAQGQAAELRALHEGAADRPTLETVTRAAAALLGVAVADVAADARF
TDLGGDSLSALTFGNTLNSIFEVEVPVGVIVGPATDLRALAEYIEHESATRGARPTFASVHGATATEVRASDLTLDRFIDPAVLAAAPSLPRATGPVRTVLLTGATGFLGR
YLALEWLERVAQQDGTVICLVRATDDAAARERLDRVFDSGDPALWERYRGLAQRHLRVLAGDKGSANLGLDEQTWRELADTVDLIVDPAALVNHVLPYSELFGPNVIGTAE
LIRLALTGRIKSYDYISTVGVGDQIEPAAFTEDADVRAISAVRRIDDSYANGYGNSKWAGEVLLREAHDLVGLPVAVFRCDMILADGVSIGQLNLPDMFTRLMLSLVATGI
APESFYELDADGHRQRAHYDGLPVDFIAAAVAELGVGEGFSTYHVMNPHDDGIGLDQYVDWLVEAGYRIDRIADYRSWLEQFETKLRALPERQRKASLLPLLHNYQHPTPP
LNGAIAVTDRFRAAVQEAKIGPDKDIPHVGPAVVVGYVTNLEHLGLL
>Nocardia_anaemiae
MLSEMRMERLMSRIADLYATYSDIRDARPIPETFAAIREPGMRPAQIAATVMAAYADRPALGHRVRELTTEASGRRTLRLRPEFATITYGELWARVGAIAAAWHHDPENPV
NAGDFIATLGFTSSDYTTLDLACLYLGAVAVPLQAGAAPKQLSAIIAETEPRILAATPENLTTAVECVRTGPTPERLVVFDYHPDDDDHREAFESARDCLANTPIVLESLE
QVLERGRTLPPAPLFVPENDALELLIYTSGSTGTPKGAMYTSRLVAAMWLAQPEVGGIGLNYMPMSHMAGHMSLYGVLARGGTAYFTAKSDISTLFEDIGLVRPTEMFFVP
RVCDMVFQRYQSEMERRAEPDIDRVELDRAVKTELRENFLGGRFLSPIVGSAPLAAEMKTFMESVIEMDLHDGYGSTEAGGAIMVDNVIRRPPVLDYKLVDVPELGYFGTD
RPHPRGELLLKTTTMIPGYYKRPEVTAEIFDADGFYRTGDIVAELGPDELVYLDRRNNVLKLSQGEFVAVAHLESVYASSPLLEQIFVYGSSERAYLLAVIVPTEETLRGA
DPKAALSDSLQRIAKEADLESYEVPRDFIIETERFTTENGLLSGIGKLLRPKLIERYGERLQQLYAELAAGQANELLALRREAADLPVLETVSRAARALLGCAATDLRPDA
HFTDLGGDSLAALSFSNLLRDIFAVEVPVGIIVSPANGLRELAEYVESARGSGAIRPTFAAVHTHGDLARADELTLDKFIDSQTLSIATTLPHTTDTQAVLLTGANGYLGR
FLCLEWLERLDKSDGRLICIIRGKDAAAARKRLDEAFDSGDPELLRHYRELAARRLEVLPGDIGEPNLGLNDVVWHRLADTVDLIVHPAALVNHVLPYDQLFGPNVVGTAE
LIRLAITSRIKPVTYLSTVAVAAQINPSSFVEDGDIREISPTRAIDDSYANGYGNSKWAGEVLLREAHDLCGLPVAVFRSDMILAHSRFGGQLNIPDMFTRLLLSLVATGI
APYSFYETDADGKRQRAHYDGLPADFTAEAITTIGAEVRTDFHTFDVLNPHDDGVSLDEFVDWLIESGHPIRRIDDYQEWLSRFETALRSLPEKQRQASLLPLLHAYSRPG
RPVRGAAIPTQGFTAAVQAAKVGAEQGIPHLTPALIDKYVSDLRLHGLL
>Nocardia_niwae
MRRVTRLYAEYDDIRNATPRAEVTEKVREPGAGLARIVETVMTGYADRPALGQRATELRTGETGRTTLSLLPEFDTITYGELWERVRAVAAAWHGDALDPLEAGDFVAILG
FTSSDYTTLDLACIHLGLVAVPLQSSAPVAQLTAIVGETAPRVLAATPALLDAAVTCALAESGTRRLVVFDYHPGDDDQRAAFDAARARLADAGSSVLLETFGEVLARGRS
LPQAPLFTAEPGEDPLALLIYTSGSTGTPKGAMYSQRLVARGWVNRPDVAAINLNYMPMSHVAGRGSLTGVLARGGTAYFAAESDMSTLFDDIALARPTEVFFVPRVCDMI
FQRFRSEVDRRDAPGVDRAALDAEVKTELREDFLGGRIVVAICGSAPVSAEMKSFIESVLDLELHDGYGSTEAGGVVVIDGLVQRPPVLEYKLADVPELGYFRTDKPHPRG
ELLLKSTTLISGYYKRPDITAEIFDAEGFYKTGDIVAELAPDHLVYVDRRNNVLKLSQGEFVAISHLEAVYATSPLIRQIFVYGSSERAYLLAVIVPTEDVLGWGDTEKMK
TALSESLRRLAKDAELQPYEIPRDFLIEPEPFSTVDGLLSGIGKLLRPKLKERYEPRLQELYAELAAGQANELLDLRRTALELPVLETVSRAAKAVLGCADADLRPDAHFT
DLGGDSLSALSLSNLLQEIFAVEVPVSVVISPANGLREVADYVAAERASGGRGVTYTAVHGDGAQVRAADLTLHKFLDARTIAEAPTLPRPSATARTVLLTGANGYLGRFL
CLEWLRRMHDGGGTLVCLVRGSDAAAARKRLDSAFDSGDPALISDFQTLAAEHLEVIPGDIGEPNLGLDDRMWQRLAERVDVIVHPAALVNHVLPYRHLFGPNVVGTAELI
RLALTTRLKPFTYLSTVAVAAQIDPAVFAEDGDIRRISPVRAVDDSYANGYGNSKWAGEVLLREANDLCGLPVAVFRSDMILADRRYAGQLNVPDIFTRLLLSLIVTGIAP
FSFYRTDAGGNRSRAHYDGLPVDFTAEAITALGASATAGFESFDVLNPHDDGRSLDEFVDWLIEAGHRIERIPDYDEWVTRFETALRALPERQRHASVLPLLHAYRRPAPA
VRGSALPAEKFRSAVRAAKIGADHDIPQISRELIEKYATDLTVRGLV
>Nocardia_arthritidis
MYAENDDIRNAIPRAEVAEKIREPGAGLAQIVETIMVGYADRPALGTRATELRTGETGRTTLSLLPAFDTITYGELWERVRAVAAAWHGDSREPLRAGDFVGILGFTSSDY
TTVDLACIHLGLVAVPLQSSAPVAQLSAIVEETAPRVLAATPELLDAAVTCALSQAGPQRLIVFDYHPGDDDQRSAFEAARARLAEAGSSVFVEPLGEVLARGRSLPPAPL
FAAEPGADPLALLIYTSGSTGTPKGAMYSQRLVARGWVNQRDVAAINLNYMPMSHVAGRGSLTGVLARGGTAYFTAESDMSTLFDDIALARPTELFLVPRVCDMIFQRFRG
EVDRRDGPGVDRAALEAQVKTELREHFLGGRMVMAICGSAPVSAEMKSFVESVLELELHDGYGSTEAGGVVVIDERVQRPPVLDYKLADVPELGYFRTDKPHPRGELLLKS
TTLISGYYKRPELTAEIFDAEGFYKTGDIVAELAPDRLVYVDRRNNVLKLSQGEFVAVSHLEAVYATSPLIQQIFVYGNSERAYLLAVIVPTEDVLGWGDPEKMKTALSES
LRGLAKDAELQSYEIPRDFLIEPEPFTTADGLLSGIGKLLRPKLKERYEPRLQQLYADLDAGQANELLDLRRTAVDLPVLETVSRAAKAVLGCADADLRPDAQFTDLGGDS
LSALSLSSLLHEIFEVEVPVSVVISPANNLREVADYIAAVRVSGGRSVTYTTVHGAGPQVRAADLTLDKFLDARTLAAAPSLPRPTETARTVLLTGANGYLGRFLCLEWLR
RMHDTAGTLVCLVRGSDAAAARQRLDSAFDSGDPTLIREFRELAAAHLEVIPGDIGEPNLGLDDRTWDRLAERVDLIVHPAALVNHVLPYRQLFGPNVVGTAELIRLALTT
RLKPFSYLSTVAVAAQIDPAVFAEDGDIREISPVRAVDDSYANGYGNSKWAGEVLLREANDLCGLPVAVFRSDMILADRRYAGQLNVPDIFTRLLLSLIVTGIAPFSFYRT
DADGNRSRAHYDGLPVDFTADAITALGATAGTGFQSFDVLNPHDDGRSLDEFVDWLIAAGHRIERIADYQEWFIRFETALRALPEWQRQASVLPLLHAYRRPAPAVRGSAL
PAERFRSAVRAAKIGAEHDIPRISRELIEKYATDLRLRGLL
>Nocardia_niigatensis
MADDQVRALVPLPEVAAAAQQCGQGLAGVAAILMDGYADRPAIADRATEIVTDADGRRTRRLLPAYRTTTYGELWARSGAIAAAWYSDPSHPLRAGDFLCSLGFASGDYTA
VELAGIRLGLVSVPLQSSSPAAQWHSIITETGARVLAVSLELLTPALECVLDGTPVESIVVFDFEPADDHHAEIYAAAQRRIAESGAAIGLESLAAVADRGRDLPAVSLHV
PADPDELALLIYTSGSTGTPKGAMYPQRLVTGMWLGPTVIPAPVLNFCYMPMSHVAGRMVLTGTFARGGTAYFAAASDMSTLFEDFGLARPTEAFFVPRVCDMVFQRYQSD
VQRRIAAGDAAEQADKAAKAALRQDFLGGRLARVMVGSAPISADMKEFMNSVLGQPMIDGYGSTEAGGGLLIDNEVRRPPVIDYKLADVPELGYFSTDKPYPRGEFLVKSM
QQIPGYFKRPEVTAEIFDEDGYYRTGDIVAEVRPDHLVYVDRRNNVLKLSQGEFVAVAKLEAVYATSPLIAQIFVHGSSERAHLLAVIVPSPAARALEPAEMTAAITESMQ
QIAKDAELESYEIPREFLLETEPFTLDNGLLSGIGKLLRPKLRERYGAQLEELYRAQARQQEDELLALRREAGDRPVIETVCRGARAILGGEAPQPDAHFTDLGGDSLSAL
SFSTLLREIFAVEVPVGVIVSPANDLARLAAHIEFERTGDGTRPTASTVHGGTEIRADELTLDKFIDAATLATAPGLPLAPHPPRTVLLTGANGYLGRFLCLEWLERLEES
DGTLVCVVRGHDAAAARRRLDEVFDSGDPALLRRYRELAERRLRVLAGDIGAPHFGLDEEVWRELAATVDLIVHPAALVNHVLPYDQLFGPNVVGTAEVIRLALTTIRKPV
TYLSTVAVAAQVEPAVFTEDGDIRAISAVRAVDDSYANGYGNSKWAGEVLLREAHDLCELPVAVFRSDMILAHSVFAGQFNLPDMFTRLLLSVLATGLAPKSFYALDSQGG
RRRAHYDGLPADFTAAAITTLGARVTAGFATYDVLNPHDDGISLDEFVDWLIESGHPIDRIESYPDWIARFETALRALPEHQRTHSVLPLLHAYRRPGLPLPGAALPAKRF
QEAVQQAKIGPNGDIPHLTRELIDKYVTDLRLRGLL
>Nocardia_violaceofusca
MPDDVQKAERKRRLAAAMADDEVRAAWPDADVSAALARPGLRLAELIDTVMTAYADRPAVGQRSGEIVVDAEGRRVWRLLPRFETLSYRQLWSRAGALASAWQAAGLRAGD
FVCTVGFVSSDYVTVDLAGVRLGTVAVPLQATAAVAQWHSIIAETEARVLACSAELLDAAVEAALASVTIGQLVVFDSADDDAERAAVAAARARLAESGREITLESLHDLV
EHGLALPAVPLAGVPGDDPLSLLVYTSGSTGTPKGAMYTDRLAAAMWLYTGKAPVPAITLNYLPLSHVAGRLQLGGTLARGGTAYFTARSDMSTLFEDLELTRPTELVFVP

RVCEMLLQHHQGEVEARVAAGRERVVVEDEVKTALRERLLGGRFLGAMCASAPLAPEMRAFMESVLGIGLHDGYGSTEAGGSVIVDNVVRRPPVLDYKLVDVPELGYFGTD
RPHPRGELLLKTTTMFPGYFKRPEITAEMFDEDGFYRTGDVVAELGPDHLVYVDRRNNVLKLSQGEFVTVAKLESVFSTSALIRQIFVYGSSERAYLLAVIVPSEEALTLP
DPRAALAESLQQLAKEAGLDSYEIPRDFLLETEPFTQDDGLLSGIGKLLRPKLKQRYGERLEQLYAELSREQADELAALRHGAADRPVLETVGRAARALLGCASTDIRPEA
HFTDLGGDSLSALSLSNLLTELFGVEVPVGTIVHPANTLRRLAEYIDDERHEGGRRPTLATVHGPGTRVRAADLTLGAFIDHETLTAAKSLPPASSPPRTVLLTGANGYLG
RFLCLEWLDRLDACDGTLICVIRGRDAEAARARLDEAFDSGDPVLLQRYRELAQRRLTVLPGDIAEPNFGLDDDRWRELTETVDLVVHSAALVNHVLPYDQLFGPNVVGTA
EVVRLAITARRKPVTYLSTVAVASQVDHFTEDGDIRGTSPERVIDGGYANGYGNSKWAGEVLLREAHDLCGLPVAVFRSDMILAHSDFAGQFNLPDMFTRLLLSVLATGLA
PESFYRPAADGSRARAHYDGLPADFTAAAITALGPGADGGFETFDVLNPHDDGIGLDTFVDWLVDDGHRITRITDYADWLGRFETALRALPERQRRHSVLPLLHAFRRPAP
AIAGSALPAERFRAAVRAAGTGPDGDIPHLSRELIGKYVRDLTAAGLL
>Nocardia_flavorosea
MSTGQPEARRNRMSTADANQANDSQPGQGLAQAIDAVMTRYADRPALARRATELVEDSATGRRSRHLLPEFTTLTYLELWSRARAIATAWSEQGVKPGDRVVAIGFASVDY
VTVHLAVAALGGVFVPLQAGAPVEPLRAIVAETEPRVIAASTEHLAKAAALVPAAGTRASVVLFDDDPDVDDDRESIEATSREFGDIPLESLREIESFGRTLPTAPLYVPD
AGADPLTLLIYTSGSTGAPKGAMYLENMLLKYWTGKVPQISQGKAPVILSYMPMGHMFGLATLWRTFQRGGTSHFTAHSNLSTLFEDIALAKPTEFYAVPRILDQIYQIYQ
SELANRRGEFSDPEHLDAAAKTHVRETVLGGRIQEASVGSAPISAEMKEFVHSCLGVPLRDAYGMTETGNVLRDGKISSPPVIAYKLADTPELGYHRTDKPYPRGELLVKT
ENIIAGYYRRPELTESMFDADGFYRTGDIMAEVGEGELVYVDRRNNVQKLSQGEFVTISQLEETYTDDPAIRQIYVYGNSAQAYLLAVIVPTEEVARTSGSAEALRSRLSE
ALQSSAKRAELESYEIPRDFLIEEEPFSTANGLLSEVGKLLRPRLRERYGERLEELYATLARGEADELQALRTGGPDQPVYETVGRAAQALLGCPGADLRPDAHFTDLGGD
SLSALTFSNLLADIFEIDVPVSVVISPAVTLETLSDYIRAKRTETDTRPTFASVHGAGATEIRAGDLTLDRFIDTATLDAAARLPAAASGAPETVLLTGATGYLGRFLCID
WLEILAARGGKLICLVRATDEAAARRRLEETFDTGDEYLLRHYRELAANHLEVLAGDLGEYRLGLGEDTWRRLVDEVDAIVHPGALVNHMLPYDQLFGPNVIGTAELIRLA
ITGRIKPFTNLSTIAVSHDIEPARFDEVADIRVVSPVRRCHDGYANGYGNSKWAGEVLLREAYERFGLPVTVFRSDMILTHSRYTGQLNVPDMFTRLMLSIVATGLAPRSF
YSGAVDGARPRAHYDGLPADFSSAAVTTLGGNTAGYHTYHLINPHDDGISLDTFVDWLIEAGHGIQRLDYGDWLKRVEAVMRNLPEKQRQRSLLPLLKAYERPLPPANGAA
VNAPRLQDAVAATPVAGHTEIPHLTRELILKYVADLRALGLL
>Nocardia_paucivorans
MSVDTRESRLERRIAELYATDEQFAAARPDVAVTAEVERAGRRSARVVHAVAKGYAHRPALGQRAVDYITDPRTGRTSMELLPRYETLTYREVWERAGAIASALAGNPVRA
GDRVCILGFASVDYTTIDMALTRLAAVSVPLQTGAPAERLRPIIAETEPTVLAASIDHLDDAVTAVLAGHLPARLIVFDYHPRVDDQREAFAAATEKLAEAGGPVVIETLA
EVIDRGTALPPVPLAPAEEPDSLALLIYTSGSTGAPKGAMITDRMVAEHWRAATAERWGQRGTEPSIVLGFMPMSHILGRAICWMALGSGGTVYFAAKSDLSTLLDDLALV
RPTQLTFVPRVWEMLFQRFQSEMYRRAADGREQAEVAAEVTAELRRNLLGGRFLSATTGSAPMSAEMKVWVEDLLDLPLRDGYGSTEAGSITLDGRVRRPPVIDYKLVDVP
ELGYFRTDRPYPRGELAVRSESLTPGYYRRPDVTAQVFDADGYYHTGDIFAEVAPDELVYVDRRSFVLKLSQGEFVTVSKLEAVFARSPLVRQIYVYGNSTRSYLLAVVVP
TEEAQARAGGDVEALKPLIGRSLRQVAKTAGLQSYEIPRDLIVDTTPFTLRNGLLTGSRKLARPKLEEHYGPRLEQLYTELAEAGTDELRALRRDGADRPVLETVRRAVSA
LLGTTDGAPPPEAHFTDLGGDSLSALTFANLLRDIFDVDVPVGVIVGPATDLRALAEYIDRQRGGTERPTFAAVHGAGATELRARDLTLDKFLDPATLAAAPSLPGPSSTA
RTVLLTGATGFLGRYLALEWLRRMSFVDGKLICLVRAEDDATARQRLDRTFSAGDPRLFTHYREAAAGHLEVLAGDKSEPELGLDHTTWQRLADTVDLIVDPAALVNHVLP
YSQLFGPNVVGTAELIRLALTTKQKPYTYVSTIGVSAQIDPSAFTEDADIRTIGPIRALDDSYANGYSNSKWAGEVLLREAHDLCGLSVAVFRCDLILADTEYAGQLNVPD
MFTRLMLSLLATGIAPGSFYELDSAGRRQRAHYDGLPVGFVSEAIAALGAQPAAEFRTYHVMNPYDDDISLDACVDWLIDAGHPIERIPDYETWLRRFETALRALPDRQRR
HSVLPLLHSYRQPQRPVRGSIAPTERFHAAVREAKIGPDKDIPHVTREIIGKYATDLGLLGLL
>Tsukamurella_sp._1534
MVRVDAEGADGPPERLPEVISRVFAEFADRPAFATRRGGPHGPYETLTYAQVWERVTALRAAWAREVRPGDFVAILGFTSPDFAAVDLASTLLGAPTVPLQAGAPASRIAA
ILDETAPVLLAVSAAQLDFAREAVGLSGADPRIVVFDSAVPGGTAPSRGVADLDEEVERGRALPVPAPFAPGPGTDPLVTLIYTSGSTGTPKGAMYTERIVRDAWLKVDSI
VDDDLPTGALLHFLPMSHMYGRNWLIAGLASGGTGYFAAAADMSSLFDDLADARPTAVALVPRVCELVHQRFLALEAESDAESARVYLRDEVLGGRIEAAMCGSAALSADL
QAFMEWMLGVSIRIGYGSTEAGGVIVDGVVARPPVTEYKLIDVPELGYLTTDLPHPRGELLVKSTQLIPGYYRAPQPLLDDDGFYRTGDVMAELGPDRLEYVDRRSNVIKL
AQGEFVPVAQLEAAYAASPAVHQAFLYGTSERSYLLAVVVPAPGPDGETDIEARARVLDALAQVARENGLASYEVPRDVIIEHEPFSQENGLRSGIGKLVRPALTAQYGDR
LAALYDAAEERRRDGLRGLDTDGPTVDVVIRAAALTLGVGTESLGAETRFADFGGDSLSALSLATTLEGLYDVPVPVQTIVGPTATLGGLAAHIDGALSGVGAAPTAASVH
GEGALIARAADLTLEKFIDPELLAAAPALPAATGEPHVVLLTGATGYLGRFLLLEWLRRVSRYGGTVVALVRGEDASDARRRVGAAIGDADPALAQEFSRLAADHLDVVAG
DFGSPSLGLDEATWDRLARHVDHVVHCGALVNHVLPYDQLFGPNVAATAEVVRLAVTAKRKSIDYVSTVAVVPQDDGRLLVEDDDVRVEGAERRIGADAYANGYAVSKWAG
EVLLREASDLAALPVRVFRSDMILAHSRFRGQFNDADQFTRILFSVAETGLAPRSFYLPDDAGRRPHYDGLPVDFTAEAIATLGSAGREGFRTFHVLNVNDDGVSLDSFVD
WIGERREIERVADYDEWFSRFEAALRALPDGARRRSVLPLLHSLEHPAPAGGTQALTADRFRAAVRDAGVGPGDIPHLDRALIEKYLHDFEAAGWLAR
>Mycobacterium_malmoense
MSTINHDERLERRIEELTANDPQFAAAHPDPAIEAALEEPGLRLPQVIRTVLDGYADRPALAHRAVEFVADPASGRTTLELLPRFETLTYRELGDRVGALGRAWAHDEVRV
GDRVCILGFNSVDYATIDMALATISAVSVPLQTSASLTSLQPIVAETEPTVIAASANQLPDAVELILTGQRPAKLVVFDYHPEVDDEREAVEAARTQLADTGVVVETLAEV
LERGKALPDTELPAGDEPDPLALLIYTSGSTGAPKGAMYPQSNVGKIWRRGSRNWFGESAASITLNFMPMSHVMGRGILYGTLGNGGTAYFAAKSDLSTLLEDLELVRPTE
LNFVPRIWETLFGEFQRQVARRLSEGGDRAAVEAEVLAEQREYLLGGRFIFAMTGSAPTSPELRNWVESLLQMHLMDGYGSTEAGMVLFDGEIQRPPVIDYKLVDVPDLGY
FGTDRPHPRGELLLRTENMFPGYYKRAEITANVFDEDGYYRTGDVFAEVAPDKLVYVDRRNNVLKLAQGEFVTLAKLEAEFGNSPLVRQIYVYGNSSQPYLLAVVVPTQEA
LGRWDPEALKGKIADSLQNVARQAGLQSYEVPRDFLIETTPFSLENGLLTGIRKLAWPKLKQHYGERLEQLYADLAEGQANELAELRRSGADAPVLQTVSRAAAAMLGTAA
ADLSPDAHFTDLGGDSLSALTFGNLLREIFDIDVPVGVIVSPASDLQAIANYIEGERQGSKRPTFAAVHGRDATEVHAGDLTLDKFLEAETLAAAPSLPKPTAEVRTVLLT
GATGFLGRYLALEWLERMDLVDGKVIALVRAKSDAEARARLDRTFDSGDEKLLAHYRQLAADHLEVIAGDKGEPNLGLDQQTWQRLADTVDLIVDPAALVNHVLPYSELFG
PNALGTAELIRIALTTKLKPYTYVSTIGVGDQIKPGQFVEDADIRQVSATRAVNDTYANGYGNSKWAGEVLLREAHDLCGLPVAVFRCDMILADTTYAGQLNLPDMFTRLM
LSLVATGVAPGSFYELDADGNRQRSHYDGLPVEFIAAAISTLGTQVLDNAPNGDGFQTYHVMNPYDDGIGLDEYVDWLIDAGYGIQRIADYGEWLRRFEGTMRGLPERQRQ
YSLLPLLHNYQQPEKPINGSMAPTDRFRAAVQEAKIGPDKDIPHVSPPIIVKYATDLQLLGLL
>Mycobacterium_europaeum
MSTMNHDERLERRIEVLTATDPQFAAARPDPAVEAAVGNPELRLPQVIRTVLDGYADRPALAHRVVEFVADPATGRTRLELLPRFETITYRQLGERVAALGRAWAGNEVQV
GDRVCVLGFNSVDYATIDMALATISAVSVPLQTSASLTSLQPIVTETEPTVIAASANQLPEAVELILSGHRPAKLVAFDYHPEVDDEREALETARARLADAGVAGGTLTEV
LERGEALPDAPLPTSEEADPLALLIYTSGSTGAPKGAMYPQSNVAKIWRRGSRNWFGESAASITLNFMPMSHVMGRGILYGTLGNGGTAYFAAKSDLSTLLEDLELVRPTE
LNFVPRIWETLFGEFQRRVERHMTGGADREAVAAEVLAEQRQYLLGGRFIFAMTGSAPTSPDLRTWVESLLEMHLMDGYGSTEAGMVLFDGEIQRPPVIDYKLVDVPDLGY
FSTDRPHPRGELLLRTENMFPGYYKRAETTANVFDEDGYYRTGDVFAEVAPDKLVYVDRRNNVLKLAQGEFVTLARLEAEFGNSPLVRQIYVYGNSAQPYLLAVVVPTEEA
LARWDLDTLKGKIADSLQNVARQAGLQSYEVPRDFIIETTPFSLENGLLTGIRKLAWPKLKQHYGERLEQLYAELAEGQANELAELRRNGADAPVLQTVSRAAAAMLGTAS
TELTPDAHFTDLGGDSLSALTFGNLLREIFDIDVPVGVIVSPASDLQAIARYIEGEREGTKRPTFAAVHGRDATEVHAGDLTLDKFLEAETLAAAPGLPKPAAEVRTVLLT
GATGFLGRYLALEWLERMDLVDGRVIALVRAKSDTEARARLDKTFDSGDPKLLAHYRELAADHLEVIAGDKGEANLGLDQQTWQRLADTVDLIVDPAALVNHVLPYSELFG
PNALGTAELIRIALTTKLKPYAYVSTIGVGDQIKPGQFVEDADIRQVSATRAINDNYANGYGNSKWAGEVLLREAHDLCGLPVAVFRCDMILADTTYAGQLNLPDMFTRLM
LSLVATGVAPGSFYELDAEGNRQRSHYDGLPVEFIAAAISTLGTQVLDNGEGFRTYHVMNPYDDGIGLDEYVDWLIDAGYGIQRVADYGDWLRRFEQAMRTMPERQRQYSL
LPLLHNYQQPEKPINGSMAPTDRFRAAVQEAKIGPDKDIPHVSAPIIVKYVTDLQLLGLL
>Mycobacterium_novocastrense
MTTLTREARLERRISDLFETDPQFAAARPSQGVAHAIEAAGLPLPQLVRTIFDGYAERPALGQRAVEFVTDPASGRTAAQLLPRFDTVTYRELSDRVEAAAAALTRVPVRP
GDRVAILGFTSIDYATVDMALMHLGAVSVPLQTSAPVSQLRPILAETEPVAMASSVDFLDDALELIVDGHVPQHLIVFDYHHEDDDHRATIDAAAARLDGTGVRIETLTDV
LTRGRALPVPVPADRSHDDLALLIYTSGSTGAPKGAMYLREMVANFWRRSSMAMWGGGPALPSITLNFMPMSHVMGRGILYATLGAGGTAYFVARSDLSTLLEDLSLVRPT
QLSFVPRIWDMMFQHFQTEVDRRTGDGVDRWVAETDVIADLRQNLLGGRFISAMTGSAPISPEMKAFVESLLDLHLTDGYGSTEAGSIFVDGRVSRPPVIDYRLVDVPDLG
YYRTDRPLPRGELLVKSDTMFPGYYKRPEITASVFDADGYYKTGDIVAEVGPDQLEYLDRRNNVLKLSQGEFVTVAKLEATFGTSPLIRQIFVYGNSARSYLLAVIVPTGD
ALARHDADELKTAITQSLQDVARTAGLQTYEIPREFIMETTPFTLENGLLTGIRKLARPALKERYGERLEALYTELADSQAAELRALRRHGSEGPVIETVSRAAGALLGAT
AADLRPRAHFTDLGGDSLSALTFGNLLNEIYDIEVPVGVIVSPANDLAALAAYIEFAREGGSTRATFASVHGHQATEVHASDLTLNKFIDDGTLAGAPALPGPSTEVRTVL
LTGATGFLGRYLALEWLERMAMVGGRVICLVRAKDDTSARDRLDRTFDSGDPELLRHYRELAADHLEVIAGDKGEADLGLDRATWQRLADTVDLIVDPAALVNHVLPYNQL

FGPNAVGTAELIRIALTTKIKPFVYVSTIGVGAGIAPGRFTEDGDIRLISPTRKIDGSYANGYANSKWAGEVLLREAHDLCGLPVAVFRCDMILADTTYAGQLNVPDMFTR
LILSLVATGIAPFSFYEVDDEGRRQRAHYDGLPVEFIAEAVSTLGAQVGHDAEPGFETYHVMNPYDDGIGLDEFVDWLIDAGHRIERVGDYATWLQRFDTAIRGLPERQRQ
ASLLPLLHNYQRPATPIRGSIAPTERFRTAVQNAKIGPDKDIPHVTREVIVKYATDLELLGLL
>Mycobacterium_szulgai
MSTPTLDERLERRIEQLIANDPQFAAAAPDPAVGAALEQPGMRLPQIIQTVLDSYADRPALAQRVVEYVKDPATGRTSMELLSRFETLTYGELSERVGALGRALLNESVRA
GDRVCALGFNSVDYATIDMALAQIGAVSVPLQTSAAITQLQPIVAETEPTLIASSVAQLRDAVALILSGEHKPAKLIVFDYHPQVDDEREAVEDARAQLSDAGVTAETLSD
VVERGKGLPAAPAPASDDDDPLALLIYTSGSTGAPKGAMYPQSNVGKMWRRSSRNWFGESAASITLNFMPMSHVMGRGILYGTLGNGGTAYFAAKSDLSTLLEDLELVRPT
ELNFVPRIWETLFGEFQRQVERRLAEAGDAADRTAIEAEVLAEQRQYQLGGRYIFAMTGSAPTSPELRAWVEALLEMHLMDGYGSTEAGMVLFDGEIQRPPVIDYKLVDVP
DLGYFGTDRPYPRGELLLRTENMFPGYYKRPETTASVLDEDGWYRTGDVFAEVEPDHLVYVDRRNNVLKLAQGEFVTLAKLEAEFGNSPLIRQIYVYGNSAQPYLLAVVVP
TEEALARWDLETLKPTIADSLQTVARQANLQSYEVPRDFLIETTPFSLENGLLTGIRKLAWPKLKQHYGERLEALYAELAQGQANELAELRRNGADAPVLQTVSRAAAAML
GTAASDLSPDAHFTDLGGDSLSALTFGNLLREIFDIDVPVGVIVSPANDLEAIAAYIEAERQGTKRPSFAAVHGRGATEVHASDLTLDKFLDEATLAGARDLPKPTAEVRT
VLLTGATGFLGRYLALEWLERMDLVDGKVIALVRAKSDEEARARLDRTFDSGDPKLLAHYQELAADHLEVIAGDKGEANLGLEPQVWQRLADTVDLIVDPAALVNHVLPYS
ELFGPNALGTAELIRIALTSKQKPYTYVSTIGVGDQIQPGQFTEDADIRQISATRQVNDNYANGYGNSKWAGEVLLREAHDLCGLPVAVFRCDMILADTTYAGQLNVPDMF
TRMMLSLVATGIAPASFYELDADGNRQRAHYDGLPVEFIAEAISTLGAQNVGEGFQTYHVMNPYDDGIGMDEFVDWLIEDGNSIQRIADYGQWLQRFETSLRGLPEKQRQA
SLLPLLHNYQQPEKPLRGSLAPTDRFRAAVQDAKVGPDKDIPHISPAIIAKYVSDLRLLGLL
>Mycobacterium_wolinskyi
MSTDTREERLERRIAELTATDPQFAAAIPSESVTAAVDKPGLRLPQIVRTVLKGYSDRPALGERAVKFVVDPASGRTTARLLPRFDTITYGELWSRVSAVAAALHASGVTP
GDRVAILGFTSADYTAIDTALGQVGAVSVPLQTSSSAAALAPIVVETEPSVIAASVDYVADAVELALTAHAPAQLIVFDHHPEVDDHRDALAAAKARLAAAGSAVAVDLLS
DLEERGRALPEAPAPQSDEADPLALLIYTSGSTGAPKGAMYLQSAVAKFWRRNSKAWFGPTTASINLSFMPMSHVMGRGILYASLAAGGTCYFAARSDLSTFLEDLALVRP
TELNFVPRVWEMIHGEFQSRVDSRLHDDGDRAAVETEVMDDLRHNLLGGRFISAMTGSAPISPELKAWLETFLDIHVLEGYGSTEAGMVFFDDQVQRPPVIDYKLVDVPDL
GYFGTDQPYPRGELLVKTDNMFPGYYKRPEITAGVFDEDGFYRTGDIVAEIGPDQLKYVDRRNNVLKLAQGEFVTLAKLEAVFGNSPLVRQIYIYGNSARPYLLAVVVPTE
TAVNRYEPAELKSQIAESLQQVAREADLQSYEIPRDFIVETTPFTLENGLLTGIRKLAWPKLKAHYADRLEQLYADLADGQASELRALRANGADAPVLETVSRAAGALLGA
AASDLQPDAHFTDLGGDSLSALTFGNLLREIFDVDVPVGVIVSPANDLAAIATFIESARQGTKRPSFASVHGRHTAEVAAADLTLDKFIDDQTLAAAPHLPKAGSEVRTVL
LTGATGFLGRYLALEWLERMDLVDGKVICLVRAKSDADARARLDATFDVGDAKLLAHYRDLAADHLEVIAGDKGEADLGLDKQTWQRLADTVDLIVDPAALVNHVLPYSEL
FGPNALGTAELIRIALTTKIKPYIYVSTIGVGDQIEPGRFVEDADVRVMSPVRKIDDSYANGYGNSKWAGEVLLREANDLCGLPVSVFRCDMILADTTYSGQLNVPDMFTR
MMLSLVATGIAPKSFYELDTDGNRQRAHYDGLPVEFIAEAISTLGSHVTTGFETYHVMNPYDDGIGLDEFVDWLIDAGYHIERIADYREWLQRFESTLRALPDKQRQASLL
PLLHNYQKPEHPVLGALAPTDRFRAAVQEAKIGPDKDIPHVTAPVIVQYATNLKQLGLL
>Nocardia_pseudovaccinii
MIIDTRMEQVMNRIADLYATYPDIRNARPKPETFAAIREPGMRPSQIVATVAAAYADRPALGHRVLEPTADESGRRTLRPQPEFATITYGELWARVGAIAAAWHHNPDNPI
KAGDFIATLGFTSGDYTTLDLACLYLGAVAVPLQAGAAPKQVSAIIAETEPRILAATPENLTTAVECVLAGPTPERLVVFDYHPEDDDQRDAFESARDRLANTPIVVESLE
SVLERGRTLPPAPLFVPDDDALELLIYTSGSTGTPKGAMYTSRLVTAMWLAQPEVGGIGLNYMPMSHMAGRMSLYGVLARGGTAYFTAKSDISTLFEDIGLVRPTEMFFVP
RVCDMVFQRYQSEMERRAEPGIDRAELDRAVKTELREDFLGGRFLSPIVGSAPLAAEMKTFMESVIDMDLHDGYGSTEAGGAIMVDNVIHRPPVLDYKLVDVPELGYFGTD
QPHPRGELLLKTSTMIPGYYKRPEVTAEIFDADGFYRTGDVVAELGADELVYLDRRNNVLKLSQGEFVAVAHLEAVYASSPLLEQIFVYGSSERAYLLAVIVPTDETLRSA
DPKAALSDSLQRIANEADLQSYEIPRDFLIETERFTTENGLLSGIGKLLRPKLIERYGRRLQQLYTELAEGQANELLALRREAADLPVLETVSRAAQALLGCATTDLRPDA
HFTDLGGDSLSALSFSNLLRDIFAVEVPVGVIVSPANGLRELAEYVEAAQGSGAIRPTFAAVHTHGGVARADELTLDKFIDAQTLSAATTLPHTTETQTVLLTGANGYLGR
FLCLEWLERLEKTDGTLVCIIRGKDAAAARKRLDDAFDSGDPDLLRHYRELAARRLEVLPGDIGEPNLGLDDATWHRLADTVDLIVHPAALVNHVLPYDQLFGPNVVGTAE
LIRLAITTRIKPVTYLSTVAVADRIQPSSFEEDGDIREISPTRAIDDSYANGYGNSKWAGEVLLREAHDLCGLPVAVFRSDMILAHSRYAGQLNVPDMFTRLLLSLVATGI
APYSFYETDDDGKRRRAHYDGLPVDFTAEAITAVGVAARTDFHSFDVLNPHDDGISLDEFVDWLIESGHPIRRIDDYQEWLRRFETALRGLPEKQRQASLLPLLHAYSRPG
RPVRGAVIPAKGFTAAVQGAKVGVEHDIPHLTRALIDKYVSDLRLRGLL
>Nocardia_salmonicida
MTVELAAGQLADRIKALYAEDEQIRAARPVEDVHAIVTTPGLATARIVETVMTAYADRPALGTRRTQLVENSGRSTRKLLPEFELLTYGQVWERTRALAAAWHRDGVVAGD
FVAVLGFTSADYTVLDLATIHLGGVAVPLQAGAGLEQLRSILDETEPTVFAVDTAHLDIAVDAVLAGVTPRSLIVFDHHADDDDHRDTLDAARARLRAGGSGLVLRTLDDT
IEQGRRADAAPLSVPAAGEDPLALLIYTSGSTGTPKGAMYTQRLVAVGWQPARPLTAINVNFLPMCHIAARLTLNSVLARGGTAYFTAAADLSTLFTDISLVRPTEIFLVP
RVCDMILQRFQRAVAERGDAAIDTVKTELREQFLGGRLLSVLCGSAPIAPDLRAFVESVLQLRLHDGYGSTETGGGVIFDTKVMRPPVLDYKLVDVPELGYFGTDKPYPRG
ELLLKTTTMIAGYYRRPEVTAEVFDADGFCRTGDVVVELAPDRLAYVDRRNNVLKLSQGEFVTVSRLEAVFAGADLVRQIYVYGSSERSYLLAVVVPTEAALAGPADELRA
AVSASLHRAAASAELEPYEIPRDFIIESEPFSTADGMLSGVGKLLRPKLKQRYGQRLEQLYTDLARGQEDELQRLRHDAPGLPVYDTVARAARAVLGGDPADLRPDARFGD
LGGDSLAALSYSTLLRELLAVDVPVNVLLGPDSDLAHIADYVRREREPGTHRTDSDAVHGKGATEIRAADLTLEKFVDVSTAARVPAATPAKTVLITGANGYLGRFLLLSW
LERLAPEGGKVICVVRGGDAVAARERLDAAFDTGDSELVMHFQVLAKDTLEVLPGDIGEPNLGLPQPTWDRLAGEVDLIVHSAALVNHVLPYAQLFGPNVVGTAEIIRLAL
TTTRKPVSYLSTVAVAAQGESFAEDGDVRTMSPVRRLDGSYANGYGNSKWAGEVLLREAAERFGLPVAVFRSDMILAHSHFLGQLNVPDVFTRLLLSVLVTGLAPTSFYRT
DSHGRPQRAHYDGLPADFVATAITELGAAASSGYRTYDVLNPHDDGISLDVFVDWLIEAGHRIDRIDGYDDWFARFETALRALPEPERKHTLLPLLDAYRKPGRPLRGSAL
PADGFRAAVRAAQIGPDHDIPHLPTDLIAKYVRDLKHLGILE
>Nocardia_africana
MPDDVQKAERKRRLAAAMADDEVRAAWPDADVTAALARPGLRPAELIDTVMTAYADRPAVGQRAGEIVVDASGRRVRRLLPRFETLTYRQLWSRAGALASAWQGAGVRAGD
FVCTVGFVSTDYLTVDLAGVRLRTVAVPLQATAAVAQWNSIIAETEARVLACSAELLGAAVEAALASVTIGHLVVFDSAGDDDERAAVAAARTRVAESGREIAIESLHDLV
ERGFALPAVPLASAPGDDPLSLLIYTSGSTGTPKGAMYTDRLAAAMWLYTGKTPVPAITLNYLPLSHVAGRLQLGGTLARGGTAYFTARSDMSTLFEDLELIRPTELVFVP
RVCEMLLQHHQGEVEARVAAGGERGVVEDEVKTELRERLLGGRFLAAMCASAPLAPEMRAFMESVLGIGLHDGYGSTEAGGSIIIDNKIRRPPVLDYKLADVPELGYFGTD
RPHPRGELLLKTTTMFPGYFKRPEITAEMFDEDGFYRTGDVVAELGPDHLVYVDRRNNVLKLSQGEFVTVAKLESVFSTSALIRQIFVYGSSERAYLLAVIVPTDEALALP
DPRAALAESLQELAKEAGLDSYEIPRDFLLETEPFTHDNGLLSGIGKLLRPKLKQRYGERLERLYAELSREQADELAALRHGAADRPVLETVGRAARALLGCASTDVRPEA
HFTDLGGDSLSALSLSNLLTELFGVEVPVGTIVHPANTLRRLAEYIGTERNEGGRRPTLTSVHGHGTRVRAADLTLDAFIDHETLTAAKSLPSVSTPPRTVLLTGANGYLG
RFLCLEWLHRLDARDGRLICVIRGRDADAARARLDEAFDSGDPALLQRYRELAERRLTVLPGDIAEPNFGLDEDRWRELTHTVDLVVHPAALVNHVLPYDQLFGPNVVGTA
EVVRFAITARRKPVTYLSTVAVASQVGHFTENGDIREISPERVIDGGYANGYGNSKWAGEVLLREAHDLCGLPVAVFRSDMILAHSEFAGQFNLPDMFTRLLLSVLATGLA
PESFYRPPADGGRARGHYDGLPADFTAASITALGPGADSGFETFDVLNPHDDGIGLDTFVDWLVADGHRITRIADYADWLARFETALRALPERQRRHSVLPLLHAFRRPAP
AIAGSALPAERFRAAVRAAGTGPDGDIPHLSRALIGKYVRDLTAAGLL
>Segniliparus_rotundus
MTQSHTQGPQASAAHSRLARRAAELLATDPQAAATLPDPEVVRQATRPGLRLAERVDAILSGYADRPALGQRSFQTVKDPITGRSSVELLPTFDTITYRELRERATAIASD
LAHHPQAPAKPGDFLASIGFISVDYVAIDIAGVFAGLTAVPLQTGATLATLTAITAETAPTLFAASIEHLPTAVDAVLATPSVRRLLVFDYRAGSDEDREAVEAAKRKIAD
AGSSVLVDVLDEVIARGKSAPKAPLPPATDAGDDSLSLLIYTSGSTGTPKGAMYPERNVAHFWGGVWAAAFDEDAAPPVPAINITFLPLSHVASRLSLMPTLARGGLMHFV
AKSDLSTLFEDLKLARPTNLFLVPRVVEMLYQHYQSELDRRGVQDGTREAEAVKDDLRTGLLGGRILTAGFGSAPLSAELAGFIESLLQIHLVDGYGSTEAGPVWRDGYLV
KPPVTDYKLIDVPELGYFSTDSPHPRGELAIKTQTILPGYYKRPETTAEVFDEDGFYLTGDVVAQIGPEQFAYVDRRKNVLKLSQGEFVTLAKLEAAYSSSPLVRQLFVYG
SSERSYLLAVIVPTPDALKKFGVGEAAKAALGESLQKIARDEGLQSYEVPRDFIIETDPFTVENGLLSDARKSLRPKLKEHYGERLEAMYKELADGQANELRDIRRGVQQR
PTLETVRRAAAAMLGASAAEIKPDAHFTDLGGDSLSALTFSNFLHDLFEVDVPVGVIVSAANTLGSVAEHIDAQLAGGRARPTFATVHGKGSTTIKASDLTLDKFIDEQTL
EAAKHLPKPADPPRTVLLTGANGWLGRFLALEWLERLAPAGGKLITIVRGKDAAQAKARLDAAYESGDPKLAGHYQDLAATTLEVLAGDFSEPRLGLDEATWNRLADEVDF
ISHPGALVNHVLPYNQLFGPNVAGVAEIIKLAITTRIKPVTYLSTVAVAAGVEPSALDEDGDIRTVSAERSVDEGYANGYGNSKWGGEVLLREAHDRTGLPVRVFRSDMIL
AHQKYTGQVNATDQFTRLVQSLLATGLAPKSFYELDAQGNRQRAHYDGIPVDFTAESITTLGGDGLEGYRSYNVFNPHRDGVGLDEFVDWLIEAGHPITRIDDYDQWLSRF
ETSLRGLPESKRQASVLPLLHAFARPGPAVDGSPFRNTVFRTDVQKAKIGAEHDIPHLGKALVLKYADDIKQLGLL
>Segniliparus_rugosus

MTESQSYETRQARPAGQSLAERVARLVAIDPQAAAAVPDKAVAERATQQGLRLAQRIEAFLSGYGDRPALAQRAFEITKDPITGRAVATLLPKFETVSYRELLERSHAIAS
ELANHAEAPVKAGEFIATIGFTSTDYTSLDIAGVLLGLTSVPLQTGATTDTLKAIAEETAPAVFGASVEHLDNAVTTALATPSVRRLLVFDYRQGVDEDREAVEAARSRLA
EAGSAVLVDTLDEVIARGRALPRVALPPATDAGDDSLSLLIYTSGSTGTPKGAMYPERNVAQFWGGIWHNAFDDGDSAPDVPDIMVNFMPLSHVAGRIGLMGTLSSGGTTY
FIAKSDLSTFFEDYSLARPTKLFFVPRICEMIYQHYQSELDRIGAADGSPQAEAIKTELREKLLGGRVLTAGSGSAPMSPELTAFIESVLQVHLVDGYGSTEAGPVWRDRK
LVKPPVTEHKLIDVPELGYFSTDSPYPRGELAIKTQTILPGYYKRPETTAEVFDEDGFYLTGDVVAEVAPEEFVYVDRRKNVLKLSQGEFVALSKLEAAYGTSPLVRQISV
YGSSQRSYLLAVVVPTPEALAKYGDGEAVKSALGDSLQKIAREEGLQSYEVPRDFIIETDPFTIENGILSDAGKTLRPKVKARYGERLEALYAQLAETQAGELRSIRVGAG
ERPVIETVQRAAAALLGASAAEVDPEAHFSDLGGDSLSALTYSNFLHEIFQVEVPVSVIVSAANNLRSVAAHIEKERSSGSDRPTFASVHGAGATTIRASDLKLEKFLDAQ
TLAAAPSLPRPASEVRTVLLTGSNGWLGRFLALAWLERLVPQGGKVVVIVRGKDDKAAKARLDSVFESGDPALLAHYEDLADKGLEVLAGDFSDADLGLRKADWDRLADEV
DLIVHSGALVNHVLPYSQLFGPNVVGTAEVAKLALTKRLKPVTYLSTVAVAVGVEPSAFEEDGDIRDVSAVRSIDEGYANGYGNSKWAGEVLLREAYEHAGLPVRVFRSDM
ILAHRKYTGQLNVPDQFTRLILSLLATGIAPKSFYQLDATGGRQRAHYDGIPVDFTAEAITTLGLAGSDGYHSFDVFNPHHDGVGLDEFVDWLVEAGHPISRVDDYAEWLS
RFETSLRGLPEAQRQHSVLPLLHAFAQPAPAIDGSPFQTKNFQSSVQEAKVGAEHDIPHLDKALIVKYAEDIKQLGLL
>Tsukamurella_sunchonensis
MSVEIVESTEPNTAAGAACGAGGSPGHLADVITSAFTRFADRPAFATRDGGPDGPYRGTTYGQVWHRVTALTAAWREELQPGDFVAVLGFTSPDFVVVDLATTLLGAPNVP
LQAGAPAARIAAILDETAPKILAVSAAQLPLARAAVAESGTDPRLVVFDGAAPGATTLDDEISRGAALPVPEPYRAAEPDGDRLVTLIYTSGSTGTPKGAMYTEHLVVHAW
LKVESIIDDDIPTGVLLHFLPMSHMYGRNWLIAGLAAGGVGYFASAADMSSLFDDLAEARPTAIALVPRVCELVHQRFLALEAETDAESARTYLRDEVLGGNIAAAVCGSA
ALSADLQAFMEQLLGVSIQIGYGSTEAGGVIVDGVVSRPPVIDYKLIDVPELGYLTTDQPHPRGELLVKSSQLIPGYYRSDKQVLDEDGYYRTGDVMAELGPDRLEYVDRR
SNVIKLAQGEFVPIAQLEATYSANPAVHQIFLHGTSERSYLVAVVVPAPGPADETDEQARGRVLDALATVARENELAGYELPRDVIIEREPFSAENGLRSGIGKLVRPALT
AHYGDRLAALYAAAEERRRDGLRGLDADGPVADTVVRAAALTLGVLPAELDGGTRFVDLGGDSLSALSLATTLEGLYDLPVPVQTIVGPTATLAGVVAHIEAARAGAVQAP
TAASIHGTGASIARAADLRLDRFIDADLLAAAPSKPAPHGEPKTVLLTGATGYLGRFLLLEWLRRVARNDGTVVALVRGTDADDARRRVFDAIGTADPALTEEFARLAAGH
LEVVPGDFGSPSLGLDDGAWERLTHTVDHVVHCGALVNHVLPYDQLFGPNVVATAEIVRLAVTDRRKSVDYVSTVAVVPQDDGRLLVEDDDVRVLGAERRIGADAYANGYA
VSKWAGEVLLREASDLAGLPVRVFRSDMILAHSRFRGQYNPVDQFTRLLLSIAETGLAPQSFYEPDGSGRSPHYDGLPVDFTAESIVTLGSAAREGFRTFHVLNVNDDAVS
LDSFVDWIAQERTVERIADYDEWLERFERALRALPDADRQRSVLPLLHSFARPTPAGAGGALTADRFREAVRAENVGPGDIPHLDRALVDRYLRGFEDAGWLSPEGAV
>Tsukamurella_tyrosinosolvens
MSIETVWGDDPAITGAAAAGTADGRAERLPQVIARIFARYADRPAFATRDGGPRAPYATVTYGEVWRRVTALVAAWRSELEPGDFVAILGFTSADFVTVDLATTLLGAPNV
PLQAGAPAARIAAILDETRPKVFAVSADQVGLAEQALAESAATPRVVVFDGEHAGYEGIEADVLAGGVLPAPEFYAPEPGNDPLVTLIYTSGSTGTPKGAMYTEQLVSDAW
LKVDSIVDIDLPSESLLHFLPMSHMYGRNWLIAGLASGGTGYFAGASDMSTLFDDLAAARPTALGLVPRVCELIHQRFLAAEAETDTETARAELRDRVLGGRLQAAMCGSA
ALSAELQTFMEWLLGVEIQIGYGSTEAGGVLRDGAIVRPPVTEYKLVDVPELGYFVTDSPHPRGELLVKSTQLIPGYYNSDKRIRDEEGFYHTGDVMAELAPDRLEYVDRR
SNVIKLAQGEFVPIAQLEATYAAGPDVHQIFLYGTSERSYLLGVVVPAPGPDGESDAAARVRVLDGLSAIARENDLASYEVPRDVIIEREPFSQENGLRSGIGKLVRPALT
ARYGAELEALYVAAEERRRDGLRALDADGSVTETVVRAAALTLGVLPEELDEDTRFLDLGGDSLSALSLATTLEGIYDLPVPVQAIVGPTATLRGVIAHIEEARAGGVQAP
TAASIHGPDAEVARASDLRLDRFIAPALLAAAPSLPAPHGEPSTVLLTGGTGYLGRFLLLEWMRRVAAHGGTVVALVRGADADDARRRVFAAIGTADPALTEEFTALAERH
LEVVVGDFGAPSLGLDTATWEALAERVDHVVHCGAMVNHVLPYDQLFGPNVVGTAEIARLALTVRRKSIDYVSTVAVVPQDDGRLLAEDDDVRVAGAERRIGADAYANGYA
VSKWAGEVLLREAADLADLPVRVFRSNMILAHSRFRGQYNPVDQFTRLLLSIAETGLAPASFSADPTGPRAHYDGLPVDFSAEAITRLGAAGRVGFRTFHVLNVAEGGAGL
DDFVDWIAEDRPIERIADYAEWLARFEAALRALPAEDRHRSVLPLLHSFARPAETGAGAALTADRFREAVREENVGPGDVPHLDRALIERYLDGFAATGWLA
>Tsukamurella_pulmonis
MQSSDPVVTGSATGEAGAPAERLPQVIARVFERFADRPAFATRDGGPRAPYVTVSYGEIWQRVTALAAAWQSELAPGDFVAILGFTSADFVTVDLATTLLGAPNVPLQAGA
PAARIAAILDETRPKIFAVSADQAALAEQALAESSATPRVVVFDGEHAGYEGIEADVLAGRALPDPEFFAPEPDADPLVTLIYTSGSTGTPKGAMYTEKLVTDAWLKVDSI
VDYDLPSESLLHFLPMSHMYGRNWLIAGLASGGTGYFAGASDMSTLFDDLAAARPTAIGLVPRVCELVHQRFLTLEAETDTETARVELREHVLGGRLQAAMCGSAALSAEL
QTFMEWLLGIEIQIGYGSTEAGGVLRDGEIVRPPVTEYKLIDVPELGYFVTDSPHPRGELLVKSTQLIPGYYKSDKRILDDEGFYRTGDVMAELAPDRLEYVDRRSNVIKL
AQGEFVPIAQLEATYAAGPDVHQIFLYGTSERSYLLAVVVPAPGPDGETDAQARTRVLDGLAAIARDQDLAGYELPRDVIIEREPFSQENGLRSGIGKLVRPALNARYGDE
LAALYAAAEDRRRAGLRDLDADGSVTETVVRAAALTLGALPEELDAATRFADLGGDSLSALSLATTLEGLYEVPVPVQTIVGPTATLGGIVEHIEAARAGALSAPTAASIH
GADAQVARASDLHLDRFVDPELLAAAPSIPAPHGEPSTVLVTGATGYLGRFLLLEWLRRVAPHEGTVVALVRGADADDARRRVLDAIGTSDPELTVEFAELAERHLEVVVG
DFGAPSLGLDAPTWDRLAERVDHVVHCGAMVNHVLPYDQLFGPNVVGTAEIARLAITVRRKSIDYVSTVAVVPQDDGRLLVEDDDVRVAGAERRIGADAYANGYAVSKWAG
EVLLHEASDLAGLPVRVFRSDMILAHSRFRGQYNPVDQFTRLLLSIAETGLAPASFAADPTGPRPHYDGLPVDFTAEAIVTLGAAGREGFRTFHVLNVDSDGAGLDDFVDW
IAEDRPIERIADYGEWFARFEAALQALPAEDRQRSVLPLLHSFAHPTPNGGGVALTADRFREAVREANVGPGDIPGLDRALIERYLDGFTAAGWLA

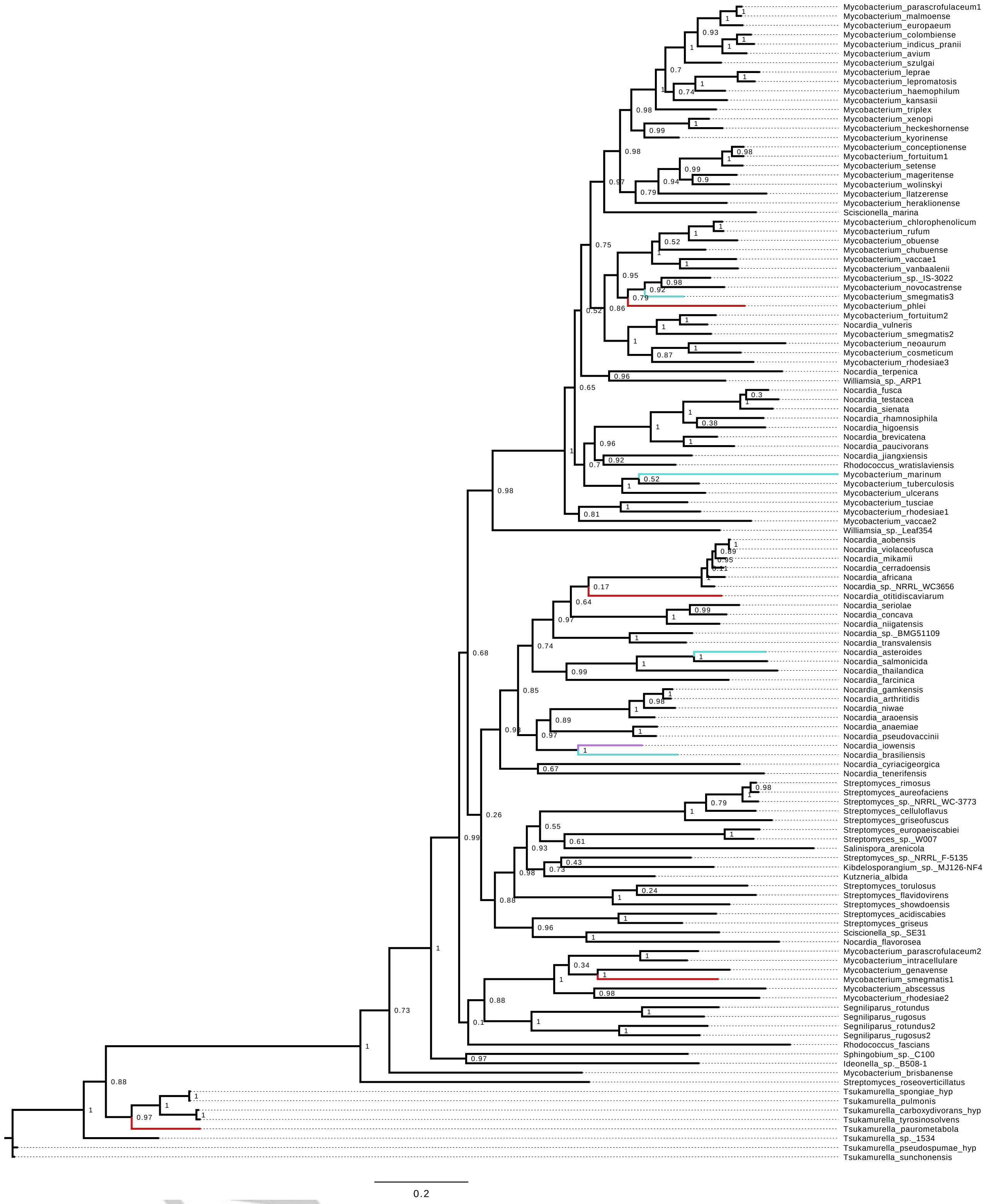

Supplementary Figure 18 - Unrooted maximum likelihood phylogeny of the 124 CAR homologues. CARs were aligned with MUSCLE, in Geneious, masked by eye, and had their phylogeny constructed in the PhyML plug-in for Geneious under the WAG+I+G model of amino acid substitution with 8 gamma rate categories. Node confidence scores are SH-like statistics computed by PhyML, with 1 being unequivocal. Coloured branches represent CARs that have been studied: Blue – in previous research, Red – in this paper, Purple – in both this paper and previous research.

	Molecular Weight	Extinction Coefficient
mpCAR	129534.6	116800
msCAR	131391.8	102235
tpCAR	122207.3	108430
niCAR	129519.6	102485
noCAR	129087.2	114850

Supplementary Figure 19 – Table of molecular weights and extinction coefficients calculated using ExPASY ProtParam.

WILEY-VCH
